# Isoxazole‐Derived Amino Acids are Bromodomain‐Binding Acetyl‐Lysine Mimics: Incorporation into Histone H4 Peptides and Histone H3

**DOI:** 10.1002/anie.201602908

**Published:** 2016-06-06

**Authors:** Angelina R. Sekirnik (née Measures), David S. Hewings, Natalie H. Theodoulou, Lukass Jursins, Katie R. Lewendon, Laura E. Jennings, Timothy P. C. Rooney, Tom D. Heightman, Stuart J. Conway

**Affiliations:** ^1^ Department of Chemistry, Chemistry Research LaboratoryUniversity of OxfordMansfield RoadOxfordOX1 3TAUK; ^2^ Nuffield Department of Clinical Medicine, Structural Genomics ConsortiumUniversity of Oxford, Old Road Campus Research BuildingRoosevelt DriveOxfordOX3 7DQUK

**Keywords:** alkylation, amino acids, bromodomain, electrostatic interactions, mass spectrometry, protein modifications

## Abstract

A range of isoxazole‐containing amino acids was synthesized that displaced acetyl‐lysine‐containing peptides from the BAZ2A, BRD4(1), and BRD9 bromodomains. Three of these amino acids were incorporated into a histone H4‐mimicking peptide and their affinity for BRD4(1) was assessed. Affinities of the isoxazole‐containing peptides are comparable to those of a hyperacetylated histone H4‐mimicking cognate peptide, and demonstrated a dependence on the position at which the unnatural residue was incorporated. An isoxazole‐based alkylating agent was developed to selectively alkylate cysteine residues in situ. Selective monoalkylation of a histone H4‐mimicking peptide, containing a lysine to cysteine residue substitution (K12C), resulted in acetyl‐lysine mimic incorporation, with high affinity for the BRD4 bromodomain. The same technology was used to alkylate a K18C mutant of histone H3.

Lysine acetylation is a prevalent post‐translational modification (PTM) which plays a fundamental role in regulating protein function.^1^, ^2^ Although acetylated lysine (KAc) residues are found throughout the cellular environment,^3^, ^4^, ^5^ it is the role of this PTM in chromatin function which has garnered most recent interest.^6^ KAc is viewed as one of the “marks” that comprises the epigenetic code,^7^, ^8^ and enzymes that write (histone acetyl transferases) and erase (histone deacetylases) this modification are well characterized.^9^ Neutralization of lysine's positive charge by acetylation weakens the interaction of histone with DNA, thus resulting in the relaxed, transcriptionally active form of DNA—euchromatin. In addition, transcriptional machinery is recruited to chromatin through the interaction of KAc with reader protein modules: bromodomains.^10^, ^11^, ^12^ The study of lysine acetylation is therefore of high interest and the generation of homogeneously modified proteins and peptides is essential to achieve this. To this end, elegant chemical strategies have been developed to synthesize single homogeneous forms of proteins, containing either KAc or an analogue which functions similarly.^13^, ^14^, ^15^, ^16^, ^17^, ^18^ The value of these modifications in uncovering the mechanistic roles of histone modifications has been demonstrated,^19^, ^20^ particularly in the study of sirtuins (see Figure S1 in the Supporting Information).^21^, ^22^, ^23^, ^24^, ^25^, ^26^, ^27^ However, only two of these KAc mimics have been employed in the study of bromodomains: *N*‐methanesulfonyl‐lysine (**6**; see Figure 2) has been reported as a stable KAc mimic which binds to bromodomains when incorporated into a p53‐mimicking peptide;^28^ KAc mimics containing methylthiocarbonylthialysine (MTCTK; **7**; Figure 2) interact two‐ to fourfold less efficiently with the bromodomain of BRDT compared to KAc.^18^

During the development of ligands for the bromodomain and extra‐terminal domain (BET) family of bromodomain‐containing proteins (BCPs), we identified the 3,5‐dimethylisoxazole (DMI) moiety as an effective KAc mimic.^29^, ^30^, ^31^ Sharp et al. subsequently showed that the DMI moiety is most potent based on a comparison of KAc mimics.^32^ Our analysis of the ChEMBL data base (https://www.ebi.ac.uk/chembl/) revealed that 4‐phenyl‐3,5‐dimethylisoxazole (**1**; Figure 1 A) possesses the highest binding efficiency index and surface efficiency index^33^ for the BET BCPs, BRD2 and BRD4. We therefore decided to investigate whether the DMI motif is a general KAc mimic which can be incorporated into unnatural amino acids, and whether peptides in which KAc has been substituted for these amino acids would retain affinity for bromodomains. If successful, these amino acids would be of high utility in generating homogenous peptides and proteins in which the DMI functions as a stable replacement for a KAc mark.

**Figure 1:**
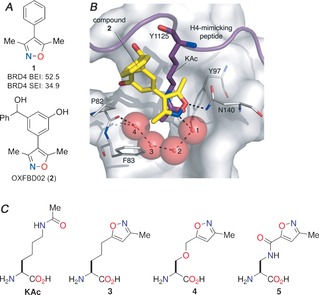
A) Structures of 4‐phenyl‐3,5‐dimethylisoxazole (**1**), which has a high binding efficiency index (BEI) and surface efficiency index (SEI) for the BRD4 bromodomains, and OXFBD02 (**2**). B) An overlay of the X‐ray crystal structure of the BET bromodomain ligand **2** bound to BRD4(1) (PDB code: 4J0S, carbon: yellow) with the X‐ray crystal structure of the histone H4‐mimicking peptide H4_1–12_KAc5KAc8 (PDB code: 3UVW, carbon: purple) bound to BRD4(1). Some residues are omitted for clarity. C) The structures of KAc and the isoxazole‐containing amino‐acids **3**–**5** incorporated into peptides in this study.

Analysis of the X‐ray crystal structure of OXFBD02 (**2**; Figure 1 A)^31^ bound to BRD4(1) (PDB code: 4J0S, carbon: yellow) and the X‐ray crystal structure of the histone H4‐mimicking peptide H4_1‐12_KAc5KAc8 (PDB code: 3UVW, carbon: purple) in complex with BRD4(1) (Figure 1 B) informed the design of ten isoxazole‐containing amino acids (**3**–**5** and **8**–**14**; Figure 2). Varying chain lengths and locations of hydrogen‐bond donors and acceptors were incorporated (see the Supporting Information for syntheses). Substitution of the isoxazole core at either the 3‐, 4‐, or 5‐position was intended to probe the optimum orientation for KAc pocket binding (**12**–**14**). Inclusion of an amide on the linkage might allow formation of further hydrogen bonds with the bromodomain (**5** and **10**–**14**). The phenyl ring of the phenylalanine derivatives **8** and **9** was predicted to interact with the hydrophobic residues in the ZA channel region of the binding pocket. As the aim was to identify general KAc mimics, the structures of these compounds were kept flexible to facilitate binding to the maximum number of bromodomains. Although some isoxazole‐containing amino acids have been reported previously,^34^, ^35^ in these compounds the isoxazole is attached directly to the α‐carbon atom, and so is unlikely to penetrate the KAc binding pocket effectively.

**Figure 2:**
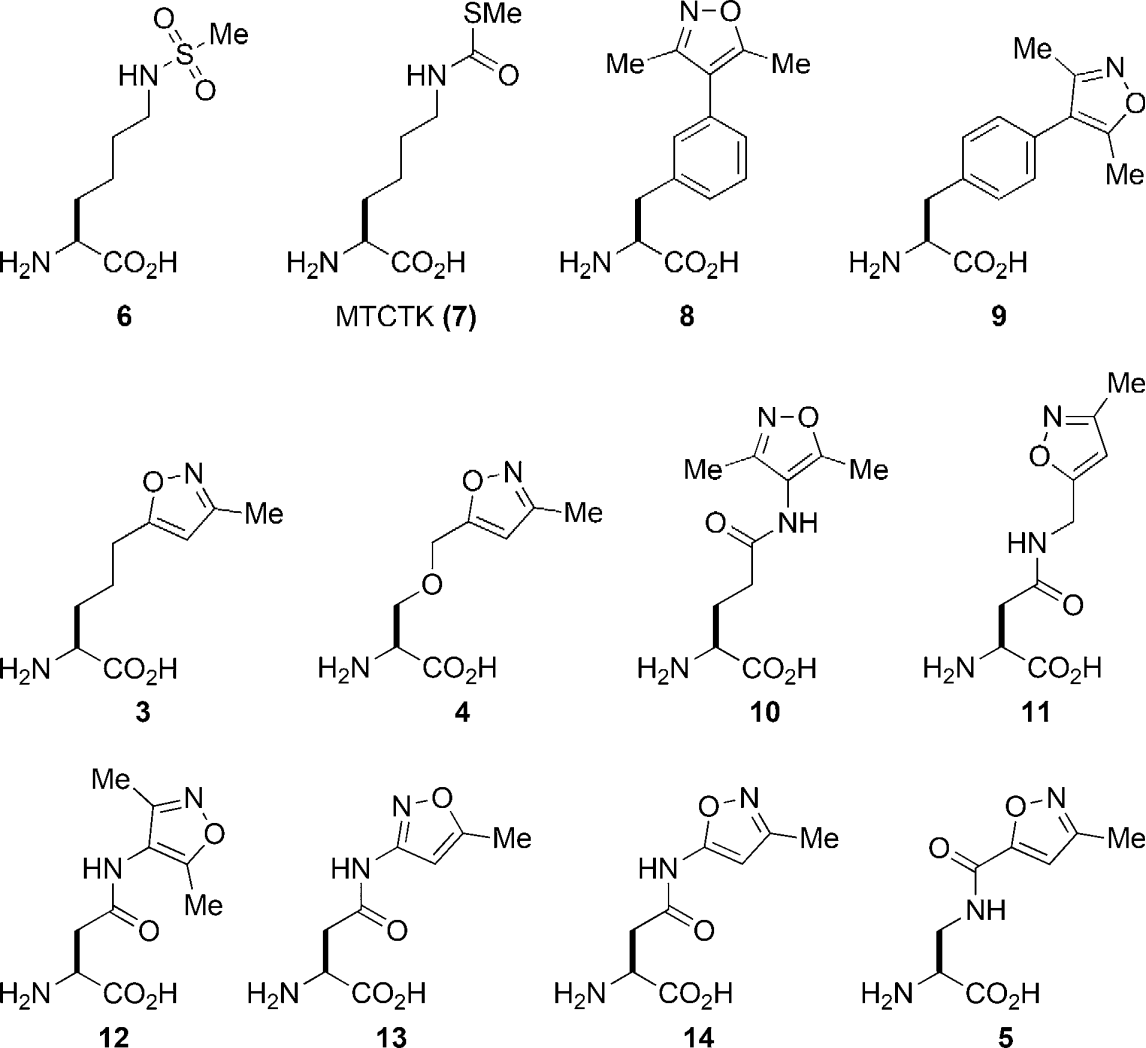
The structures of previously reported acetyl‐lysine mimicking amino acids **6** and **7**, and the isoxazole‐containing amino‐acids **3**–**5** and **10**–**14**.

KAc has a low affinity for bromodomains.^36^ In histone peptides the surrounding residues are thought to provide much of the bromodomain affinity, which complicates the assessment of the bromodomain‐binding abilities of individual amino acids. Nonetheless, the percentage inhibition AlphaScreen data, obtained at amino‐acid concentrations of 50 μm or 250 μm, were used to assess the binding of the individual amino acids to the phylogenetically diverse BAZ2A, BRD4(1), and BRD9 bromodomains (Table 1). All of the amino acids showed some concentration‐dependent binding to at least one bromodomain. Interestingly, some of the amino acids showed selectivity for one or two of the bromodomains, with the phenyl‐containing amino‐acid **8** showing the highest affinity for BRD4(1), but no binding to BRD9. The compound **5** showed the highest affinity for both BAZ2A and BRD9. These data indicate that isoxazole‐containing amino acids can act as KAc mimics in the context of bromodomain recognition. The data also suggest that ligand selectivity between bromodomains can be achieved by altering interactions in the KAc binding pocket, in addition to the peptide‐binding region (see the Supporting Information).

**Table 1:** Percentage inhibition of bromodomain‐KAc recognition by isoxazole‐containing amino acids.^[a]^

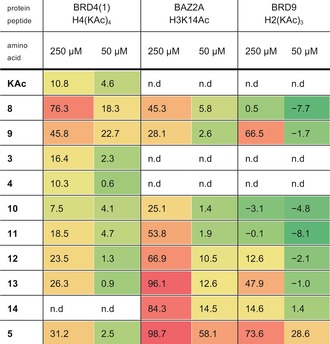

[a] n.d=not determined.

Having established that isoxazole‐containing amino acids can bind to diverse bromodomains, we next sought to determine how effectively these amino acids could emulate KAc when incorporated into a histone‐mimicking peptide. For these studies we selected amino‐acids **3**–**5**, based on a combination of their bromodomain affinity and structural flexibility. We decided to focus on BRD4(1) and an H4‐mimicking peptide as a model system, as the binding of variously acetylated histone H4‐mimicking peptides, and a large number of small‐molecule ligands, to BRD4(1) have been well characterized.^37^

The histone H4 tail possesses four lysine residues which can be acetylated, and are located at positions 5, 8, 12, and 16. The tetra‐acetylated histone H4‐mimicking peptide H4_1–20_KAc5KAc8KAc12KAc16 [H4(KAc)_4_] shows the highest affinity for BRD4(1), with reported *K*_D_ values of 3.1 μm (ITC)^36^ and 4.8 μm (ITC).^38^ BRD4(1) can recognize two adjacent KAc residues on the same peptide at any one time.^39^ X‐ray crystal structures reveal the N‐terminal KAc forms a hydrogen‐bonding interaction with the conserved asparagine residue, while flexible glycine residues allow a peptide conformation where the C‐terminal KAc fits into the grooves created by the ZA and BC loops (PDB: 3UVW, 3UVX, 3UVY). It was not clear whether an isoxazole‐containing amino acid could effectively mimic a KAc residue in either of these binding modes, or whether two adjacent isoxazole‐containing amino acids would be accommodated by BRD4(1). Therefore, the amino‐acids **3**–**5** were sequentially introduced into H4(KAc)_4_ in place of KAc at either position 5, 8, 12, or 16. IC_50_ values for the resulting peptides were determined using an AlphaScreen assay (Figure 3) [inhibiting the interaction of BRD4(1) with biotinylated H4(KAc)_4_], with inclusion of unbiotinylated H4(KAc)_4_ to determine whether the KAc mimics could interact more strongly than KAc with bromodomains.

**Figure 3:**
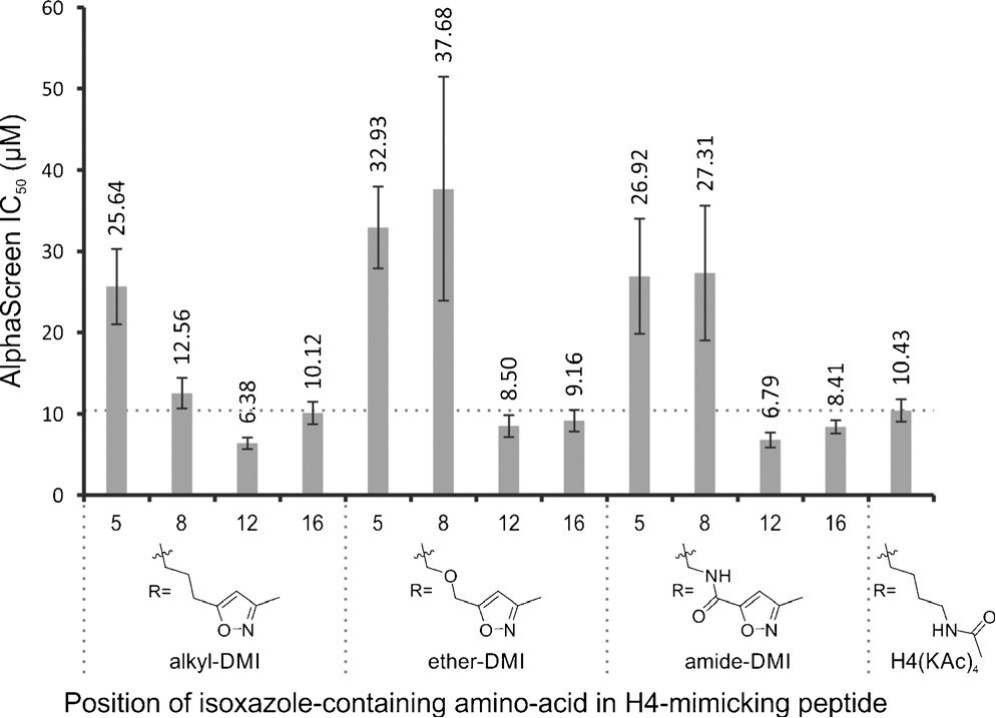
IC_50_ values for H4(KAc)_4_‐mimicking peptides bearing isoxazole‐containing amino acids (substitution position indicated numerically), obtained using an AlphaScreen assay inhibiting the interaction of BRD4(1) with biotinylated H4(KAc)_4_. The guideline shows the IC_50_ value of control H4(KAc)_4_ peptide for comparison. IC_50_ values (in μm above bars) are geometric means, from 4‐parameter fittings^40^ to data obtained in triplicate. Error bars represent the error of the fit.

Affinities of the modified peptides for BRD4(1) were dependent on the position at which the unnatural residue was incorporated. Substitution at either K5 or K8 reduced the affinity compared to H4(KAc)_4_, with these peptides showing IC_50_ values in the 12.6–37.8 μm range. The alkyl‐based amino‐acid **3** had the least detrimental effect on affinity when incorporated at these positions. However, substitution at either K12 or K16 resulted in peptides with similar affinities compared to that of the reference peptide (IC_50_=6.4–10.1 μm). When incorporated at these positions, the amide‐linked isoxazole **5** bound more strongly than the control peptide, with IC_50_ values of (6.8±0.9) and (8.4±0.8) μm for the K12 and K16 position, respectively. Positional sensitivity was consistent with studies on a histone H4‐mimicking peptide, which demonstrated that dual acetylation at K5 and K8 was almost as potent as H4(KAc)_4_.^39^ Given the apparent high contribution of KAc at these positions to the overall peptide affinity, it seems reasonable that their substitution is less well tolerated. Overall, these data indicate that amino‐acids **3**–**5** are able to mimic KAc when incorporated into a peptide.

We next sought to develop a complementary, tag‐and‐modify, approach in which a cysteine residue could be alkylated by an isoxazole‐containing electrophile. Based on the alkylation procedure used in the synthesis of the serine derivative **4** (see Scheme S8), we investigated whether 5‐(chloromethyl)‐3‐methylisoxazole (Cl‐DMI) could be used to modify a cysteine within a peptide in situ. Trials were first performed with this alkylating agent on a model tripeptide (YCK), thus indicating that selective alkylation on a cysteine residue could be achieved (see Figure S2). The reaction was then carried out using an H4‐mimicking peptide with a cysteine residue replacing lysine 12, H4_1‐20_KAc5KAc8K12CKAc16 [H4(KAc)_3_K12C; see Figure S3], as our data suggested that an isoxazole‐containing amino acid would be well tolerated at this position.

The degree of alkylation was determined using analytical HPLC, which allowed separation of the reactant and product, and identification of by‐products. In addition, the reaction could be followed using MALDI‐TOF‐MS (Figure 4). HPLC integral ratios from reaction mixtures correlate well with peak ratios in MALDI‐TOF‐MS spectra (see Figure S7). The site of alkylation was confirmed as the cysteine residue by targeted nano‐LC tandem MS/MS (see Figure S8) and MALDI‐TOF‐MS/MS, with laser‐induced fragmentation (see Figure S9). Importantly, the characteristic peak resulting from amide‐bond cleavage adjacent to the alkylated cysteine was observed at *m*/*z* 1148.75 (expected *m*/*z* 1148.58), but was not present in the starting peptide.

**Figure 4:**
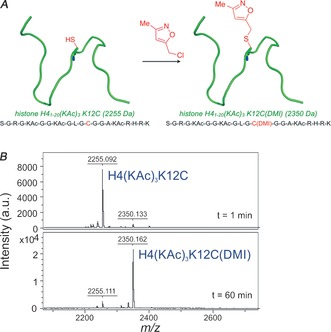
A) A cartoon representing H4(KAc)_3_K12C alkylation. B) Regions of MALDI‐TOF‐MS spectra of samples taken at the time indicated during the alkylation of H4(KAc)_3_K12C under optimized conditions: 1 mm H4(KAc)_3_K12C, 4 m guanidine hydrochloride, 10 mm methionine, 5 mm TCEP, 1 m CHES (pH 9), 5 mm Cl‐DMI, showing conversion from starting peptide (observed *m*/*z* 2255.09; expected *m*/*z* 2255.21) to alkylated H4(KAc)_3_K12C(DMI) peptide (observed *m*/*z* 2350.07; expected *m*/*z* 2350.24).

Only one equivalent of Cl‐DMI was required for almost complete alkylation after 1 hour, whereas excess (≥5 equiv) reagent gave rise to unidentified by‐products (see Figure S10). Once the alkylation had been optimized (see Table S2) the desired product was purified using HPLC. Isolated H4(KAc)_3_K12C(DMI) was lyophilized and its affinity to BRD4(1) assessed in an AlphaScreen assay, and compared to the affinities of H4(KAc)_3_ peptides containing the amino‐acids **3**–**5** at position 12 (Figure 5). The thioether‐linked DMI showed an IC_50_ value of (2.87±0.3) μm, which is the lowest observed for any of the reported peptides in this assay. The decreased affinity of the H4(KAc)_3_K12C starting peptide, relative to H4(KAc)_4_, was confirmed by ITC [*K*_D_=(32.4±6.7) μm; see Figure S12]. This result corroborates the ability of this isoxazole‐containing amino acid to mimic the interactions of KAc with bromodomains.

**Figure 5:**
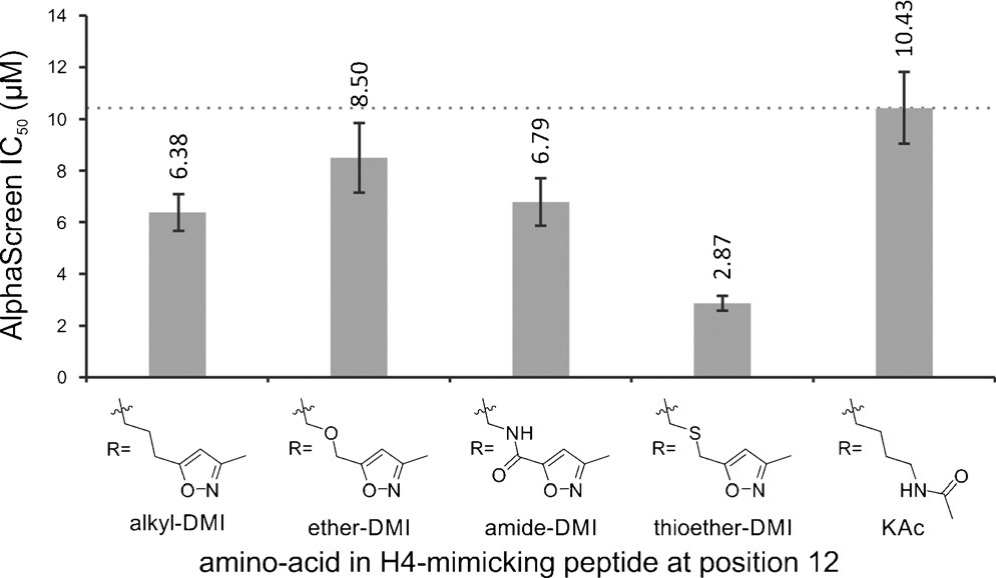
IC_50_ values for H4(KAc)_4_ analogue peptides bearing isoxazole‐containing amino‐acid substitutions at K12 (identity of substitution is shown below bars). Data were obtained from serial dilutions in an AlphaScreen assay inhibiting the interaction of BRD4(1) with biotinylated H4(KAc)_4_. Guideline shows IC_50_ value of the control H4(KAc)_4_ peptide for comparison. IC_50_ values (in μm above bars) are geometric means, from 4‐parameter fittings^40^ to data obtained in triplicate. Error bars represent the error of the fit. See Figure S11.

To determine whether an isoxazole‐derived KAc mimic could be incorporated into a protein, a K18C mutant of human histone H3, a position that is endogenously acetylated, was treated with Cl‐DMI under the optimized alkylation conditions (Figure 6). After 3 hours, a mass corresponding to the monoalkylated protein was the sole species observable by LCMS (see Figure S13: observed mass 15 309 Da). These data indicate selective incorporation of the DMI into the histone H3 protein.

**Figure 6:**
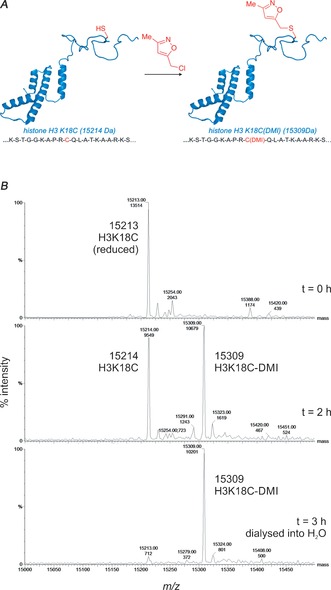
A) A cartoon representing histone H3 alkylation. B) LCMS monitoring H3K18C protein alkylation with Cl‐DMI. Deconvoluted masses (range 10.0 kDa–20.0 kDa, resolution 1.0 Da, zoomed region 15.0 kDa‐15.5 kDa) observed at time indicated bottom right. Spectra show progression from starting protein [H3K18C expected mass 15214] to monoalkylated product [H3K18C(DMI) expected mass 15309]. See the Supporting Information for full spectra (see Figure S12).

In conclusion, ten structurally diverse isoxazole‐containing amino acids were synthesized. They are the first unprotected monomeric amino acids able to prevent a histone‐mimicking peptide binding to the bromodomains of BAZ2A, BRD4(1), and BRD9. Incorporation into histone H4‐mimicking peptides confirmed that these isoxazole‐containing amino acids have the ability to mimic the interactions of KAc with bromodomains. Peptides bearing these modifications demonstrated increased bromodomain affinity in a positionally dependent manner. Furthermore, an isoxazole‐containing amino acid has been introduced into histone H4‐mimicking peptides using selective cysteine alkylation. Notably, a high‐yielding, specific alkylation of H4(KAc)_3_K12C gave an isoxazole‐containing peptide with an IC_50_ value, against BRD4(1), which was approximately a third of the cognate H4(KAc)_4_ peptide. Mild conditions enabled this method to be extended to modify a mutant of human histone H3 containing a single cysteine, thus demonstrating that this strategy is applicable to whole proteins. The complementary approaches that we report, which enable incorporation of effective and stable KAc mimics in a site‐selective manner into both peptides and proteins, will allow precise activation of KAc‐mediated protein–protein interactions, thus facilitating study of their downstream effects in an unprecedented manner.

## Supporting information

As a service to our authors and readers, this journal provides supporting information supplied by the authors. Such materials are peer reviewed and may be re‐organized for online delivery, but are not copy‐edited or typeset. Technical support issues arising from supporting information (other than missing files) should be addressed to the authors.

Supplementary
