# Supplementary material for: Isoxazole‐Derived Amino Acids are Bromodomain‐Binding Acetyl‐Lysine Mimics: Incorporation into Histone H4 Peptides and Histone H3
Source: Angew Chem Int Ed Engl. 2016 Jun 6;55(29):8353–7. doi: 10.1002/anie.201602908 (PMC5089653; doi:10.1002/anie.201602908)

Angewandte Chemie
Eine Zeitschrift der Gesellschaft Deutscher Chemiker

Supporting Information

Isoxazole-Derived Amino Acids are Bromodomain-Binding Acetyl-Lysine Mimics: Incorporation into Histone H4 Peptides and Histone H3

*Angelina R. Sekirnik (née Measures)[+], David S. Hewings[+], Natalie H. Theodoulou, Lukass Jursins, Katie R. Lewendon, Laura E. Jennings, Timothy P. C. Rooney, Tom D. Heightman, and Stuart J. Conway**

anie_201602908_sm_miscellaneous_information.pdf

Table of Contents

Supporting Figure S1: Acetyl-lysine-mimicking amino acids. 2
Supporting Figure S2: Alkylation of tripeptide (YCK). 3
Supporting Figure S3: Chemical structure and predicted masses of starting peptide [$H4_{1-20}(KAc)_3K12C$] and alkylation products. 4
Supporting Figure S4: Regions of MALDI-TOF-MS spectra of samples taken during the alkylation of $H4_{1-20}(KAc)_3K12C$. 5
Supporting Figure S5: Selected regions of MALDI-TOF mass spectra obtained from reaction of $H4(KAc)_3K12C$ with various alkylating agents. 6
Supporting Figure S6: HPLC comparison of products obtained from reaction of $H4(KAc)_3K12C$ with various alkylating agents. 7
Supporting Figure S7: Region of interest in HPLC trace of partial alkylation conditions. 8
Supporting Figure S8: Fragmentation ions identified by targeted LC/MS/MS to identify the location of alkylation. 9
Supporting Figure S9: MALDI-TOF-TOF fragmentation species observed after alkylation. 10
Supporting Figure S10: Full elution profile from analytical-HPLC of excess alkylation reaction. 11
Supporting Figure S11: AlphaScreen emission readings from serial dilutions of $H4(KAc)_3K12X$ peptides. 12
Supporting Figure S12: Isothermal titration calorimetry (ITC) data for peptides against BRD4(1). 13
Supporting Figure S13: Deconvoluted masses from LCMS of H3K18C alkylation 14
Supporting Table S1: Percentage inhibition of bromodomain-KAc recognition by isoxazole-containing amino acids 15
Supporting Table S2: Results from reaction condition optimization. 16
Supporting Scheme S1: Synthesis of dimethylisoxazole trifluoroborate salt. 17
Supporting Scheme S2: Synthesis of *meta*-phenylalanine derived amino acid. 17
Supporting Scheme S3: Synthesis of *para*-phenylalanine derived amino acid. 17
Supporting Scheme S4: Synthesis of isoxazole-containing extended electrophile. 18
Supporting Scheme S5: Synthesis of alkyl coupled amino acid. 18
Supporting Scheme S6: Synthesis of isoxazole-containing bromine electrophile. 19
Supporting Scheme S7: Synthesis of isoxazole-containing chlorine electrophile. 19
Supporting Scheme S8: Synthesis of ether coupled amino acid. 19
Supporting Scheme S9: Synthesis of glutamic acid derived amino acid. 20
Supporting Scheme S10: Synthesis of aspartic acid derived amino acids. 20
Supporting Scheme S11: Synthesis of *iso*-amide coupled amino acid. 20
Supporting Scheme S12: Optimised alkylation of tripeptide. 21
Biochemical Methods 21
Synthetic Methods 23
General Procedures 25
Synthesis and Characterization of Compounds 26
References 44
^{1}H and ^{13}C spectra 45

Supporting Figure S1: Acetyl-lysine-mimicking amino acids.

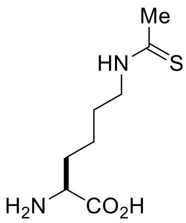
15

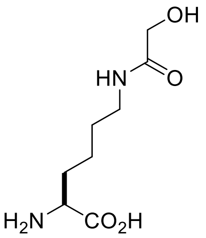
16

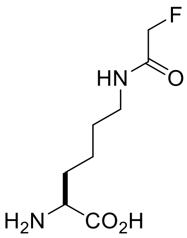
17

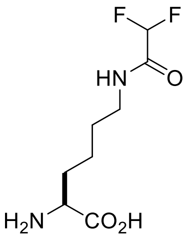
18

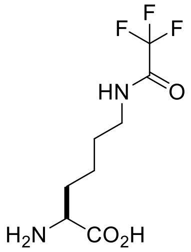
19

Fatkins *et al.*[1]

Smith *et al.*[2]

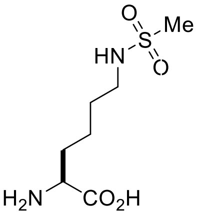
6

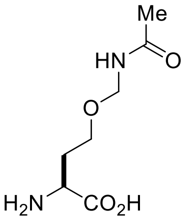
20

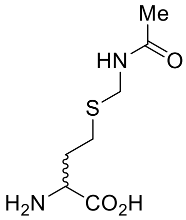
21

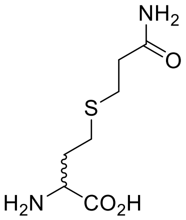
22

Jamonnak *et al.*[3]

Jamonnak *et al.*[4]

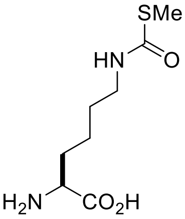
7

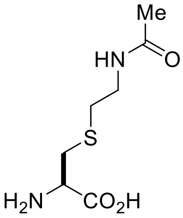
23

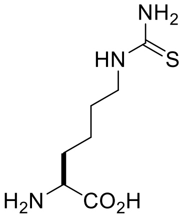
24

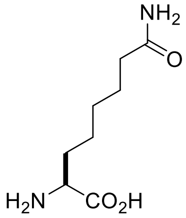
25

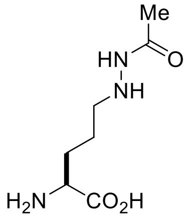
26

Huang *et al.*[5]

Hirsch *et al.*[6]

Hirsch *et al.*[7]

Dancy *et al.*[8]

Previously developed acetyl-lysine-mimicking amino acids have predominantly been utilised as chemical probes of histone deacetylases, particularly sirtuins.

Supporting Figure S2: Alkylation of tripeptide (YCK).

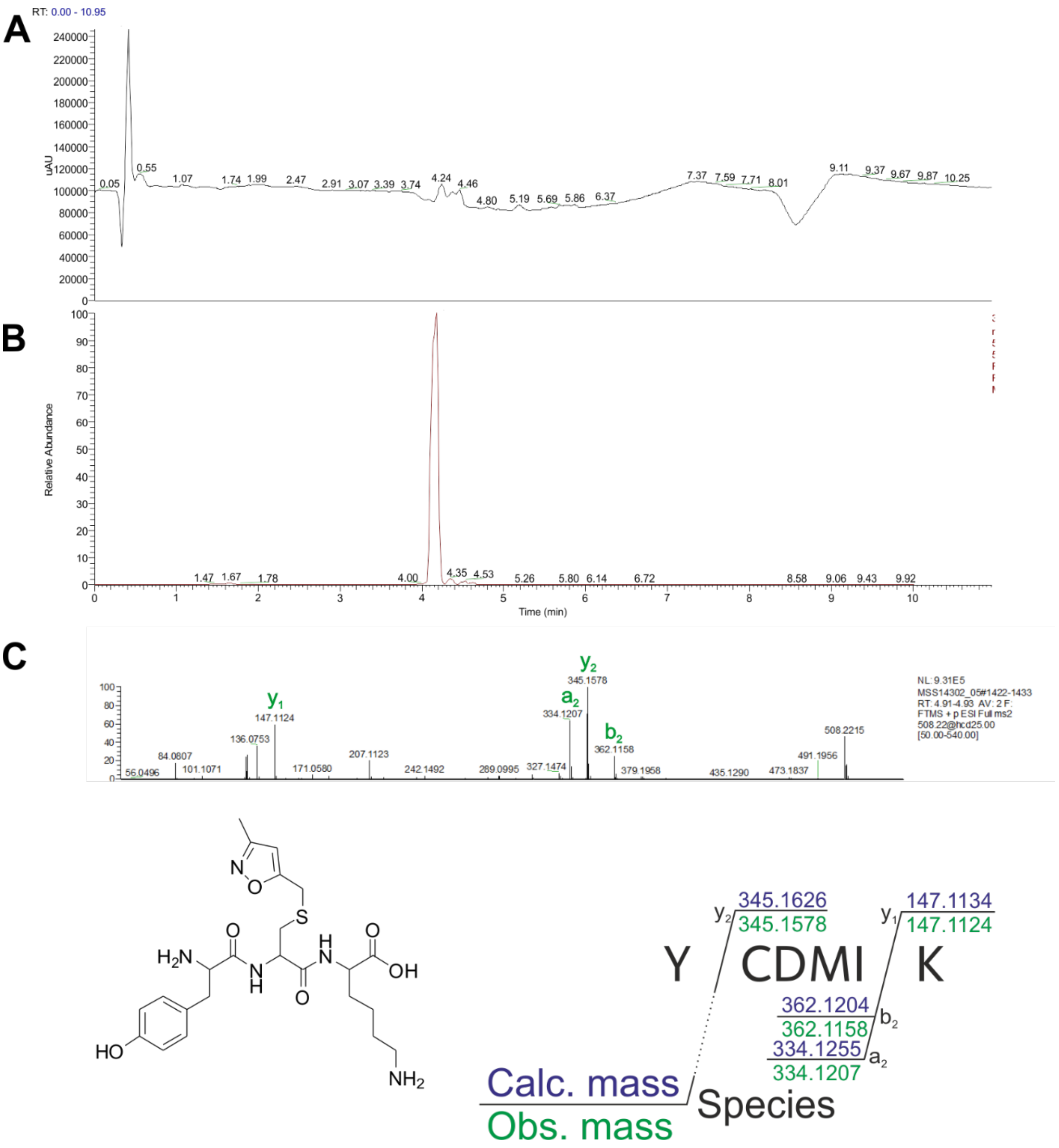

A LCMS chromatogram of optimized alkylation of tripeptide, reaction conditions: 1 M HEPES, 10 mM L-methionine, 4 M guanidinium chloride, 12 mM YCK, 12 mM Cl-DMI, 85 mM TCEP, pH 7.8, 20 °C, argon atm., 1.5 h. **B** Displayed mass chromatogram of *m/z* 508.22 (corresponding to monoalkylation) shows a single peak. **C** Targeted LCMS/MS fragmentation spectra (CID) of peak at retention time 4.18 min (parent ion *m/z* 508.22). Desired monoalkylated tripeptide species shown, with diagnostic a-, b-, and y-type ions observed, confirming specific alkylation of the cysteine residue (CDMI). Calculated mass (Da) – blue; observed mass (Da) – green.

Supporting Figure S3: Chemical structure and predicted masses of starting peptide [H4$_{1\text{-}20}$(KAc)$_3$K12C] and alkylation products.

Tyr-Ser-Gly-Arg-Gly-Lys(Ac)-Gly-Gly-Lys(Ac)-Gly-Leu-Gly-Cys-Gly-Gly-Ala-Lys(Ac)-Arg-His-Arg-Lys-NH_2

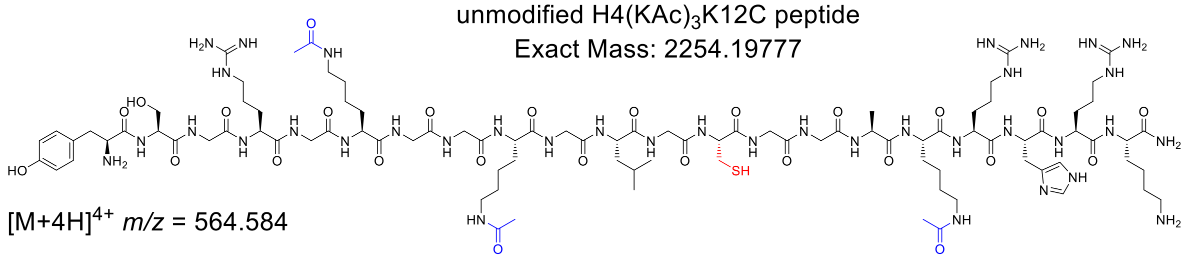

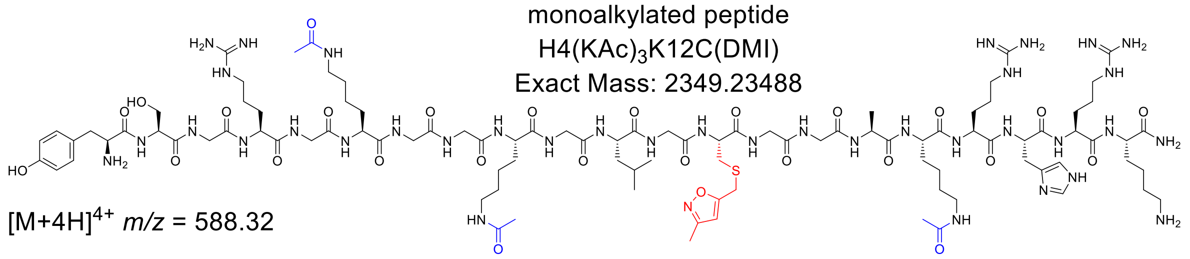

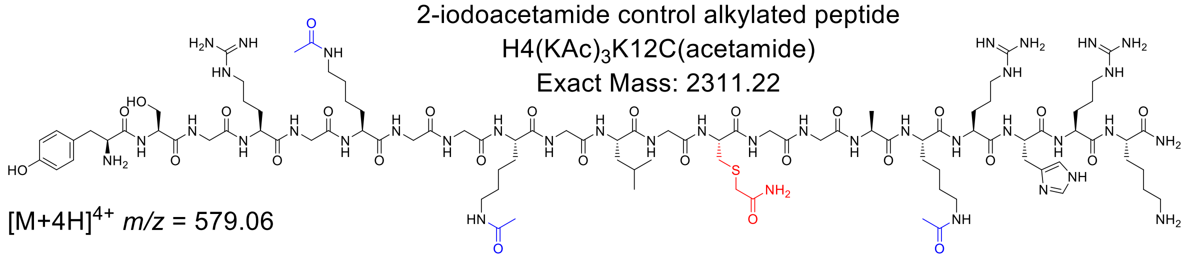

Sequence and modifications of starting peptide displayed. Modifications highlighted in blue. Cysteine residue and corresponding alkylated residues highlighted in red. Monoalkylation at K12C with 5-(chloromethyl)-3-methylisoxazole (Cl-DMI) or 2-iodoacetamide (2IA). Mass of $[M+4H]^{4+}$ parent ion shown as used in targeted MS.

Supporting Figure S4: Regions of MALDI-TOF-MS spectra of samples taken during the alkylation of $H4_{1\text{-}20}(KAc)_3K12C$.

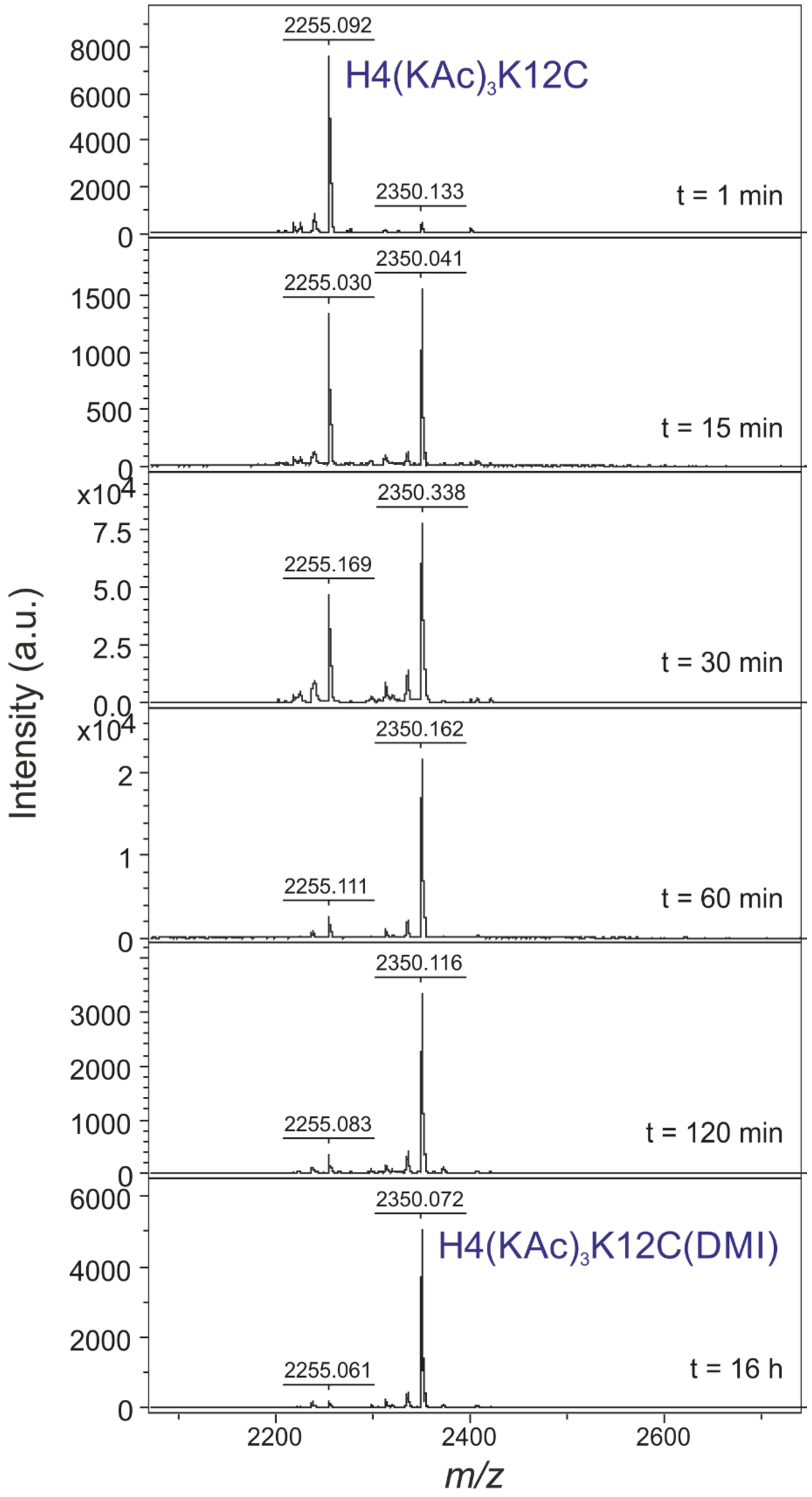

Samples were taken at the time indicated, from the reaction under optimized conditions: 1 mM $H4(KAc)_3K12C$, 4 M guanidine hydrochloride, 10 mM L-methionine, 5 mM TCEP, 1 M CHES (pH 9), 5 mM Cl-DMI. This extended time course shows further details (see Figure 4) of conversion from starting peptide (observed *m/z* 2255.09; expected *m/z* 2255.21) to alkylated $H4(KAc)_3K12C(DMI)$ peptide (observed *m/z* 2350.07; expected *m/z* 2350.24).

Supporting Figure S5: Selected regions of MALDI-TOF mass spectra obtained from reaction of $H4(KAc)_3K12C$ with various alkylating agents.

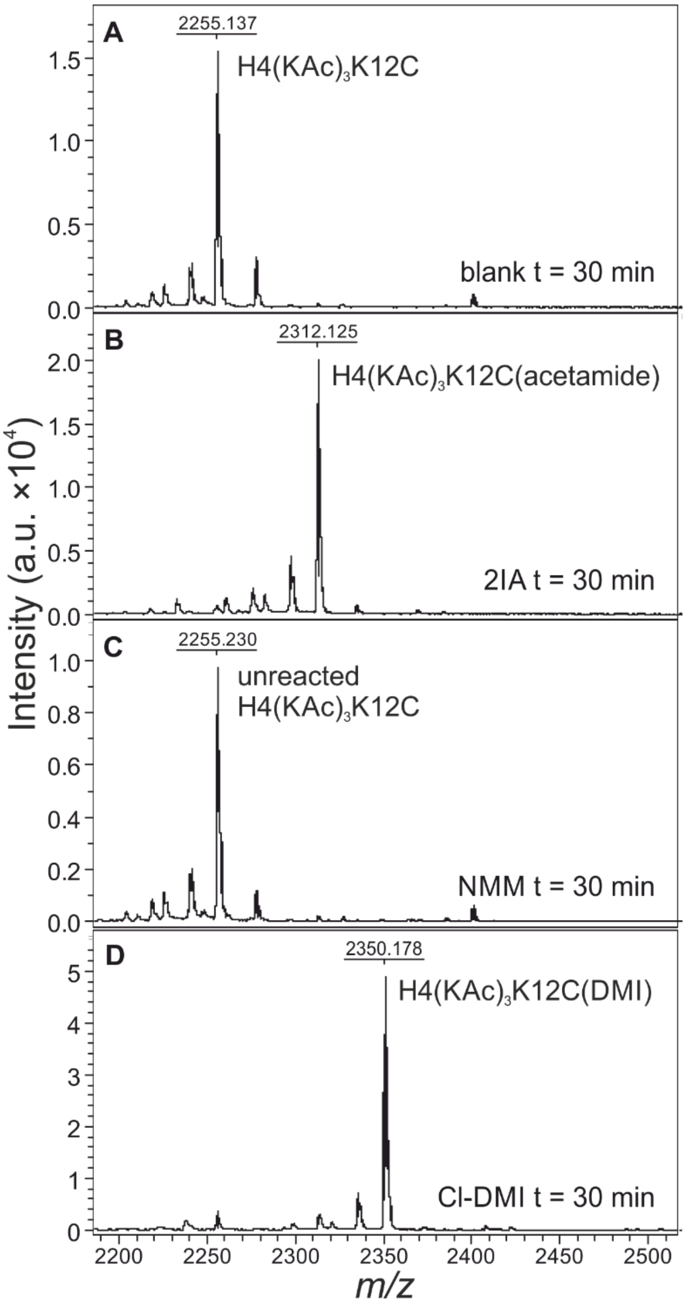

Samples were taken from reaction mixtures after 30 minutes, comparing alkylation with: **A**, buffer (blank); **B**, 2-iodoacetamide (2IA); **C**, *N*-methylmaleimide (NMM); **D**, 5-(chloromethyl)-3-methylisoxazole (Cl-DMI). 2IA caused almost complete conversion of the $H4(KAc)_3K12C$ peptide into the alkylated product within the first sampling interval (**B**): a mass shift of +57 Da was observed, corresponding to addition of the acetamide, with the percentage of product peak integrals (2312.19 Da) compared to the starting material (2254.66 Da) of 93.9% after 5 min. NMM, however, formed negligible amounts of alkylated product (**C**), with no peak observed at the expected mass of 2366.24 Da within the duration of experiment (2 h). There was no mass shift for the peptide under the reaction conditions alone (**A**), confirming that alkylation observed was indeed specific to the Cl-DMI agent.

Supporting Figure S6: HPLC comparison of products obtained from reaction of H4(KAc)$_3$K12C with various alkylating agents.

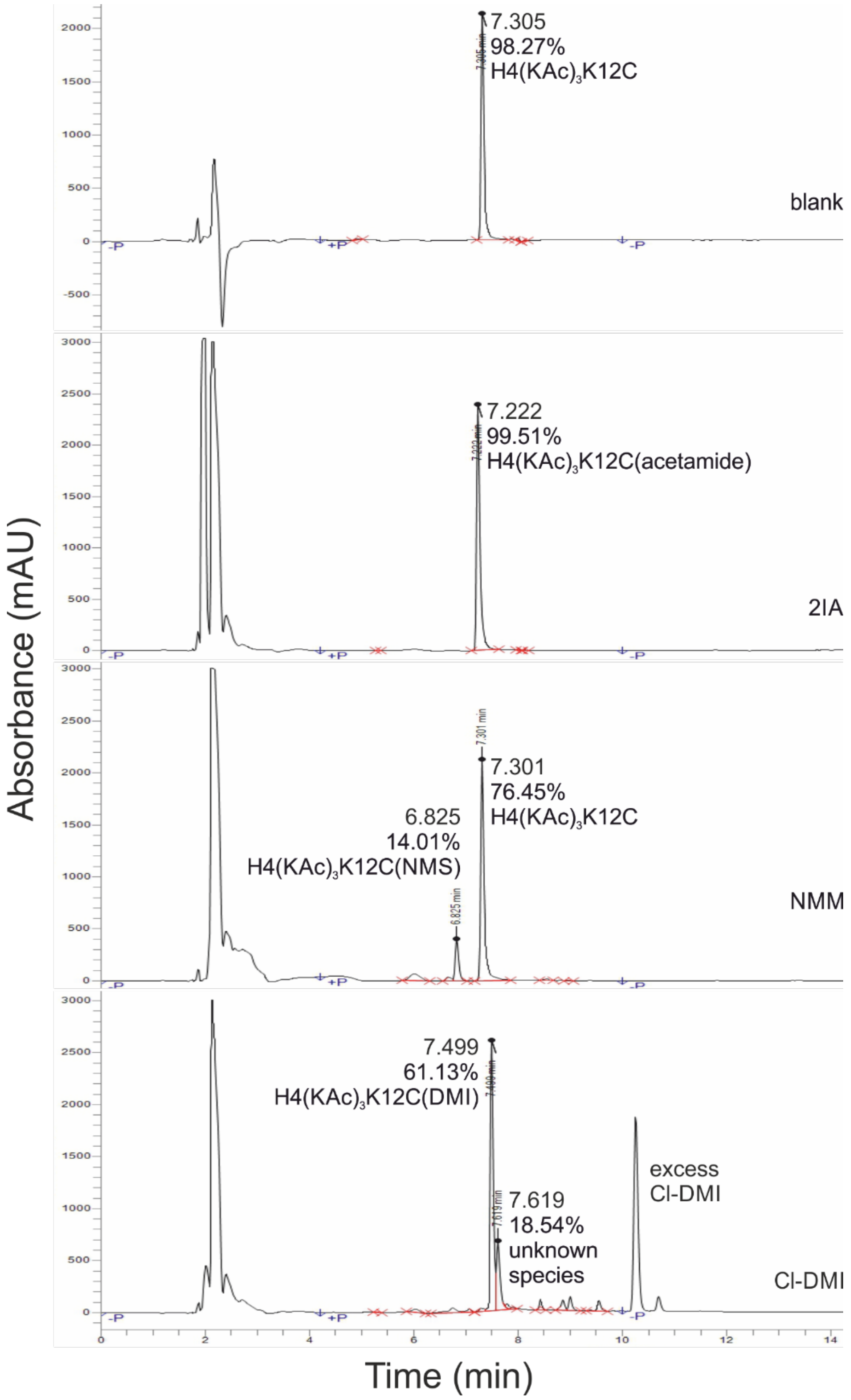

Peaks corresponding to contrasting alkylation products (Cl-DMI, 2IA and NMM) were observed at different retention times. Reaction mixtures after 2 h: 1 mM H4(KAc)3K12C, 4 M guanidine·hydrochloride, 10 mM L-methionine, 5 mM TCEP, 1 M CHES (pH 9). Alkylating agent (5 mM) shown bottom right: buffer; 2-iodoacetamide (2IA); *N*-methylmaleimide (NMM); 5-(chloromethyl)-3-methylisoxazole (Cl-DMI)

Supporting Figure S7: Region of interest in HPLC trace of partial alkylation conditions.

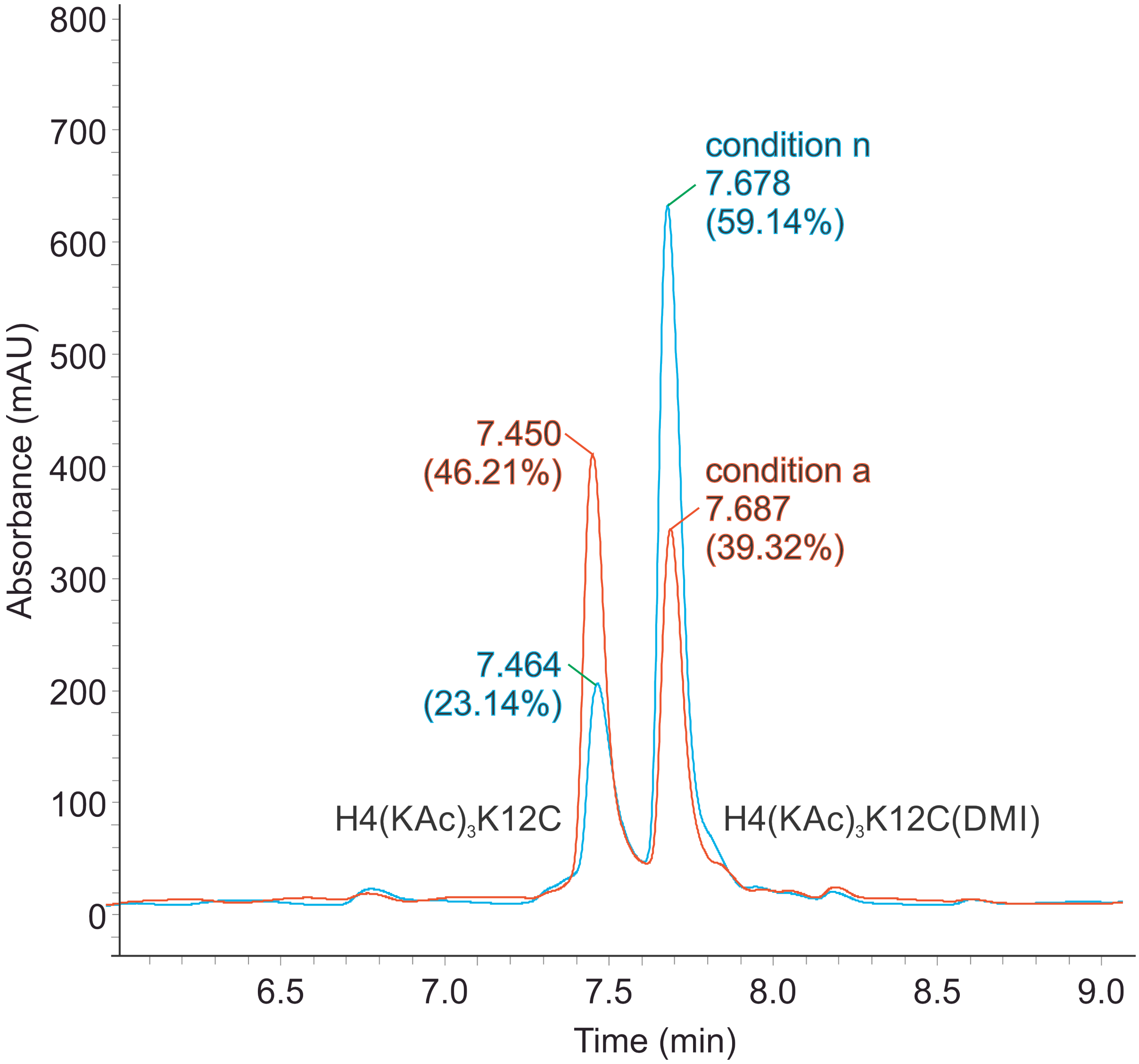

UV traces show conversion from H4(KAc)$_3$K12C (7.5 min) to H4(KAc)$_3$K12C(DMI) (7.7 min). Reaction conditions as stated in Supporting Table S2: 1 mM H4(KAc)3K12C, 4 M guanidine·hydrochloride, 10 mM L-methionine, 5 mM TCEP, 1 M CHES (pH 9). Condition a: 1 mM Cl DMI; product peak integral ratio 45.97% (41.04% by MALDI). Condition n: 2 mM Cl-DMI; product peak integral ratio 71.88% (74.42% by MALDI). Samples injected after 16 h.

Supporting Figure S8: Fragmentation ions identified by targeted LC/MS/MS to identify the position of alkylation.

m/z tolerance	Intensity threshold	Precursor ion	Predicted sequence	Calculated MW	Observed MW
0.30	104 (0.750%)	4+	YSGRGKGGKGLGCGGAKRHRK	2254.1978	2254.3687

	Tyr	Ser	Gly	Arg	Gly	KAc	Gly	Gly	KAc	Gly	Leu	Gly	Cys	Gly	Gly	Ala	KAc	Arg	His	Arg	KAc
a	136.08<br>-0.00	223.11<br>-0.01	280.13<br>---	436.23<br>-0.03	493.25<br>0.01	663.36<br>-0.03	720.38<br>-0.02	777.40<br>-0.03	947.51<br>-0.03	1004.53<br>-0.02	1117.61<br>0.05	1174.63<br>---	1277.64<br>---	1334.66<br>---	1391.69<br>---	1462.72<br>---	1632.83<br>---	1788.93<br>---	1925.99<br>---	2082.09<br>---	2252.19<br>---
b	164.07<br>-0.01	251.10<br>-0.06	308.12<br>-0.01	464.23<br>-0.02	521.25<br>0.00	691.35<br>-0.05	748.37<br>-0.10	805.40<br>-0.00	975.50<br>-0.05	1032.52<br>-0.03	1145.61<br>-0.23	1202.63<br>---	1305.64<br>---	1362.66<br>---	1419.68<br>-0.10	1490.72<br>---	1660.82<br>---	1816.92<br>---	1953.98<br>---	2110.08<br>---	2280.19<br>---
y	2255.21<br>---	2092.14<br>-0.06	2005.11<br>-0.08	1948.09<br>---	1791.99<br>-0.07	1734.97<br>---	1564.86<br>0.01	1507.84<br>-0.08	1450.82<br>-0.12	1280.71<br>-0.06	1223.69<br>0.01	1110.61<br>-0.04	1053.59<br>0.05	950.58<br>-0.04	893.55<br>0.09	836.53<br>-0.05	765.50<br>0.12	595.39<br>0.00	439.29<br>-0.01	302.23<br>-0.01	146.13<br>-0.00
z	2238.18<br>---	2075.11<br>-0.19	1988.08<br>---	1931.06<br>-0.07	1774.96<br>-0.12	1717.94<br>0.01	1547.83<br>0.02	1490.81<br>---	1433.79<br>-0.15	1263.68<br>0.01	1206.66<br>-0.08	1093.58<br>-0.06	1036.55<br>-0.03	933.55<br>-0.05	876.52<br>-0.04	819.50<br>-0.03	748.47<br>-0.01	578.36<br>-0.02	422.26<br>-0.01	285.20<br>0.02	129.10<br>-0.01

Modifications: amidation deacetylation (+) lysine acetylation (+)

m/z tolerance	Intensity threshold	Precursor ion	Predicted sequence	Calculated MW	Observed MW
0.30	127 (0.750%)	4+	YSGRGKGGKGLGCGGAKRHRK	2349.2349	2349.2488

	Tyr	Ser	Gly	Arg	Gly	KAc	Gly	Gly	KAc	Gly	Leu	Gly	CDMI	Gly	Gly	Ala	KAc	Arg	His	Arg	KAc
a	136.08<br>-0.01	223.11<br>-0.03	280.13<br>---	436.23<br>-0.05	493.25<br>-0.09	663.36<br>-0.08	720.38<br>-0.08	777.40<br>-0.07	947.51<br>-0.12	1004.53<br>-0.09	1117.61<br>-0.10	1174.63<br>---	1372.68<br>0.06	1429.70<br>---	1486.72<br>---	1557.76<br>---	1727.87<br>---	1883.97<br>---	2021.03<br>---	2177.13<br>---	2347.23<br>---
b	164.07<br>-0.02	251.10<br>-0.03	308.12<br>-0.03	464.23<br>-0.07	521.25<br>-0.05	691.35<br>-0.09	748.37<br>-0.16	805.40<br>-0.07	975.50<br>-0.07	1032.52<br>-0.11	1145.61<br>-0.05	1202.63<br>---	1400.67<br>-0.03	1457.70<br>---	1514.72<br>---	1585.75<br>-0.24	1755.86<br>0.06	1911.96<br>---	2049.02<br>---	2205.12<br>---	2375.23<br>---
y	2350.24<br>---	2187.18<br>-0.24	2100.15<br>-0.23	2043.13<br>-0.20	1887.02<br>-0.18	1830.00<br>-0.12	1659.90<br>-0.09	1602.88<br>-0.12	1545.85<br>-0.03	1375.75<br>-0.19	1318.73<br>-0.18	1205.64<br>-0.13	1148.62<br>-0.12	950.58<br>-0.08	893.55<br>-0.10	836.53<br>-0.09	765.50<br>-0.07	595.39<br>-0.04	439.29<br>-0.05	302.23<br>-0.03	146.13<br>-0.02
z	2333.21<br>---	2170.15<br>-0.27	2083.12<br>-0.18	2026.10<br>-0.20	1869.99<br>-0.21	1812.97<br>---	1642.87<br>0.03	1585.85<br>-0.15	1528.82<br>-0.15	1358.72<br>-0.10	1301.70<br>-0.17	1188.61<br>-0.12	1131.59<br>-0.14	933.55<br>-0.10	876.52<br>-0.08	819.50<br>-0.10	748.47<br>-0.07	578.36<br>-0.07	422.26<br>-0.05	285.20<br>-0.01	129.10<br>-0.02

Modifications: amidation deacetylation (+) lysine acetylation (+) cysteine isoxazole (+)

Calculated and observed ions corresponding to a-, b-, y- and z-type ions from fragmentation across peptide bonds and subsequent decarboxylation or deamidation. Observed ions (coloured) shown with deviation from calculated values (Da). Isolation of precursor ions in the 4+ charge state, followed by collision-induced dissociation (CID), allowed observation of fragmentation ions corresponding to those calculated for peptide bond cleavage of the starting peptide (from precursor *m/z* 564.6) and desired product (from precursor *m/z* 588.3). For the alkylated product these included a-, b-, y- and z-type fragment ions from cleavage adjacent to the alkylated cysteine [K12C(DMI): black box].

Supporting Figure S9: MALDI-TOF-TOF fragmentation species observed after alkylation.

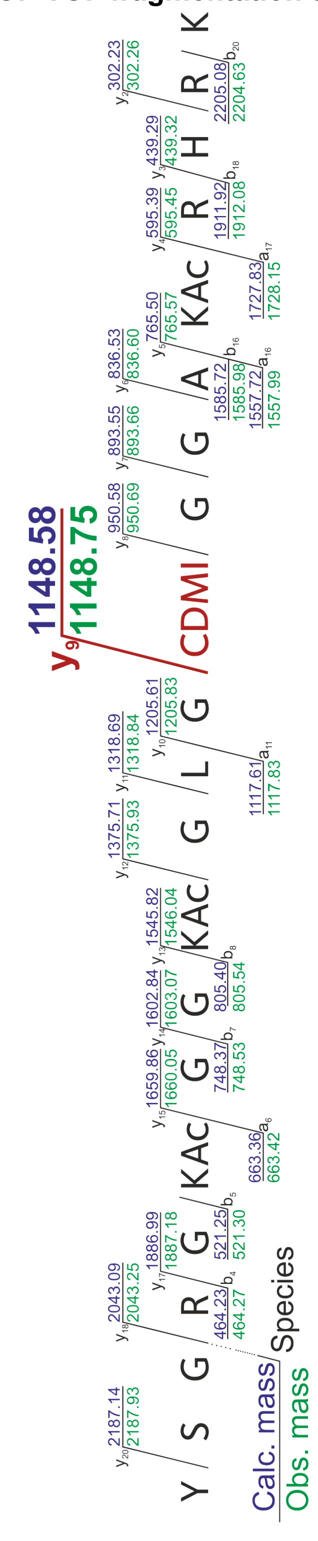

Fragmentation ions observed confirmed the site of alkylation as that of the cysteine residue (CDMI). Labels indicate a-, b-, or y-type ion and position. Calculated mass (Da) – blue; observed mass (Da) – green. Good coverage of y-type ions were detected along the full length of the peptide, with a- and b-type ions towards both the N- and C-terminus. Crucially, the characteristic peak resulting from amide bond cleavage adjacent to the alkylated cysteine was observed at 1148.75 m/z (expected m/z 1148.58) and was not present in the starting peptide.

Supporting Figure S10: Full elution profile from analytical-HPLC of excess alkylation reaction.

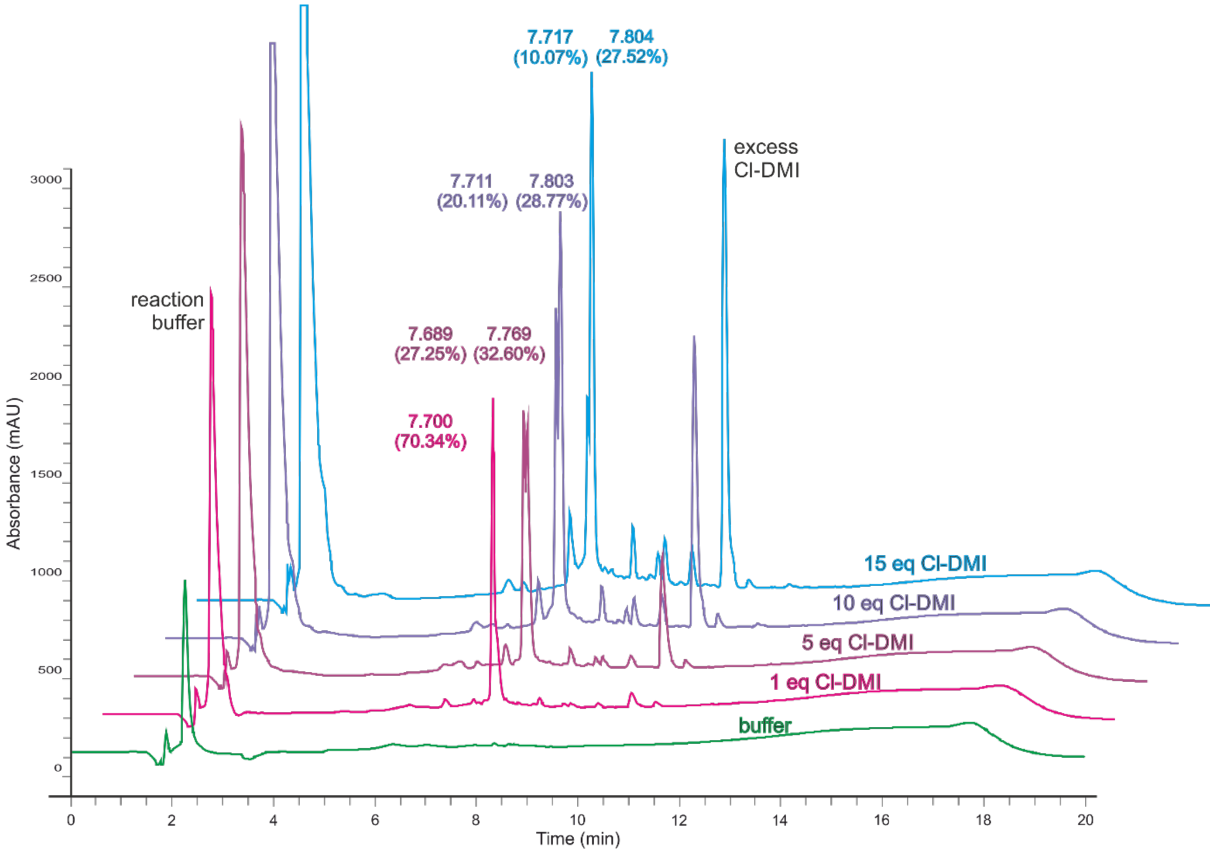

Gradient elution 0-100% B, 2-10 min (eluent A: 95% H_2O, 5% MeCN, 0.1% TFA; eluent B: 5% H_2O, 95% MeCN, 0.1% TFA). Large peaks at the start of elution are caused by high concentrations of reaction buffer; unreacted, excess Cl-DMI eluted >10 min at 100% B.

To investigate whether over alkylation was occurring, the starting peptide was subjected to high concentrations of Cl-DMI and analyzed by HPLC. Spectra demonstrate selective alkylation to give a single peak at 7.7 min [H4(KAc)$_3$K12C(DMI) peptide] with the use of 1 eq Cl-DMI. With increasing equivalents, several unknown products were formed with retention times of >6 min; the most significant new product eluted with a retention time of 7.8 min. Observation of these species at higher equivalents, reinforces the correct, mono-alkylated product as the isolated species from 1 eq.

Supporting Figure S11: AlphaScreen emission readings from serial dilutions of $H4(KAc)_3K12X$ peptides.

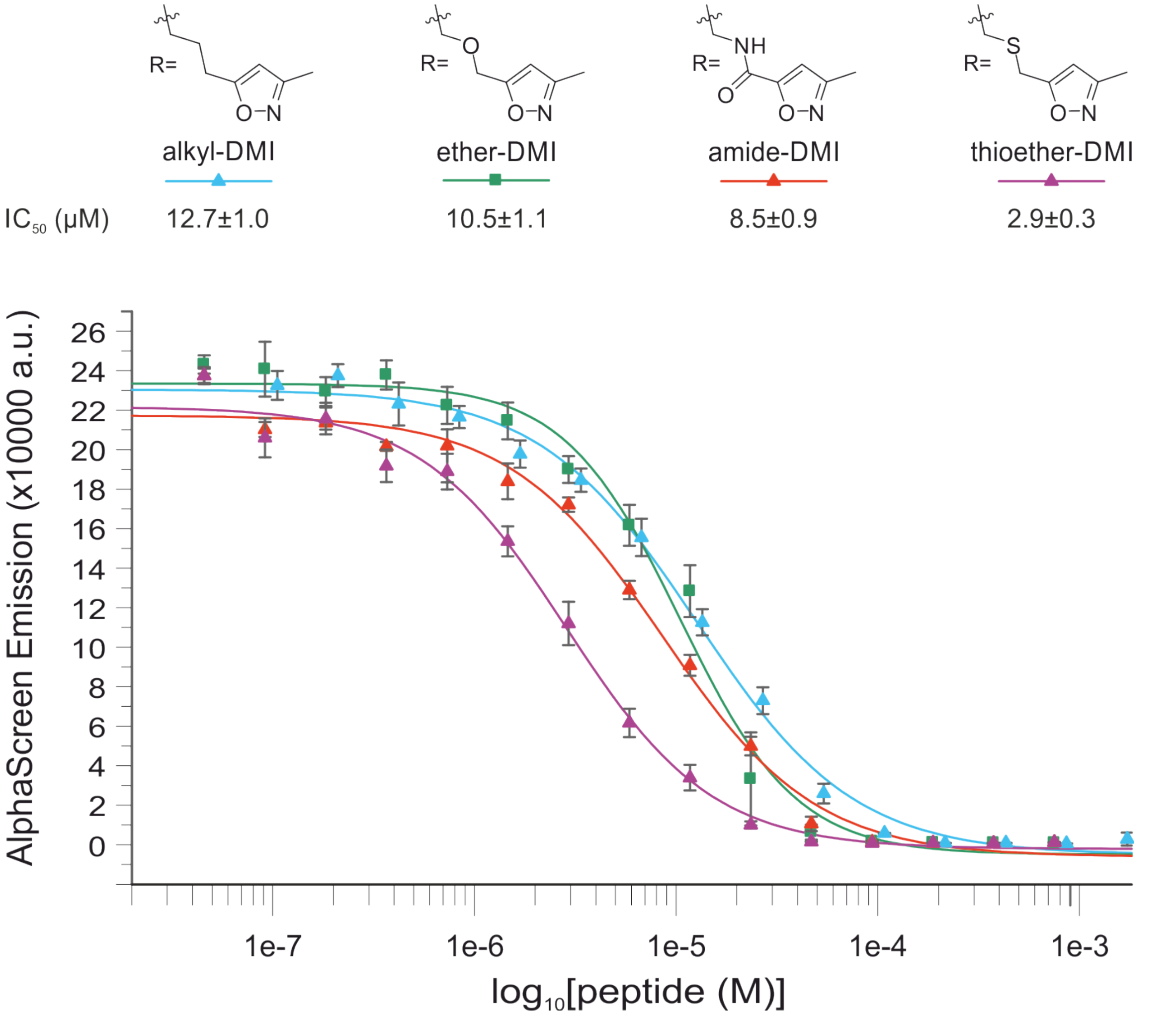

AlphaScreen assay inhibiting the interaction of BRD4 with biotinylated $H4(KAc)_4$. IC_{50} values for peptides based on the $H4_{1\text{-}20}(KAc)_4$ sequence, bearing amino-acid substitutions at the K12 position, shown in μM. Results are the average of readings taken in triplicate; error bars represent the standard deviation. Data fitting was performed according to a full 4-parameter equation, where the x axis represents the (logarithmically scaled) concentration of analyte, range is the maximum y range (response minus background), and s is a slope factor:

$$y = \frac{range}{1 + \left(\frac{x}{IC_{50}}\right)^{s}} + background$$

Values reported in the manuscript are geometric means of multiple results obtained from fitting to data in triplicate: $H4(KAc)_4$ control peptide n=6, $H4(KAc)_3$K5/8/16>**3**/**4**/**5** n=2, $H4(KAc)_3$K12>**3**/**4**/**5** n=4, $H4(KAc)_3$K12CDMI n=1. Errors stated are geometric means of the errors of the fitting.

Supporting Figure S12: Isothermal titration calorimetry (ITC) data for peptides against BRD4(1).

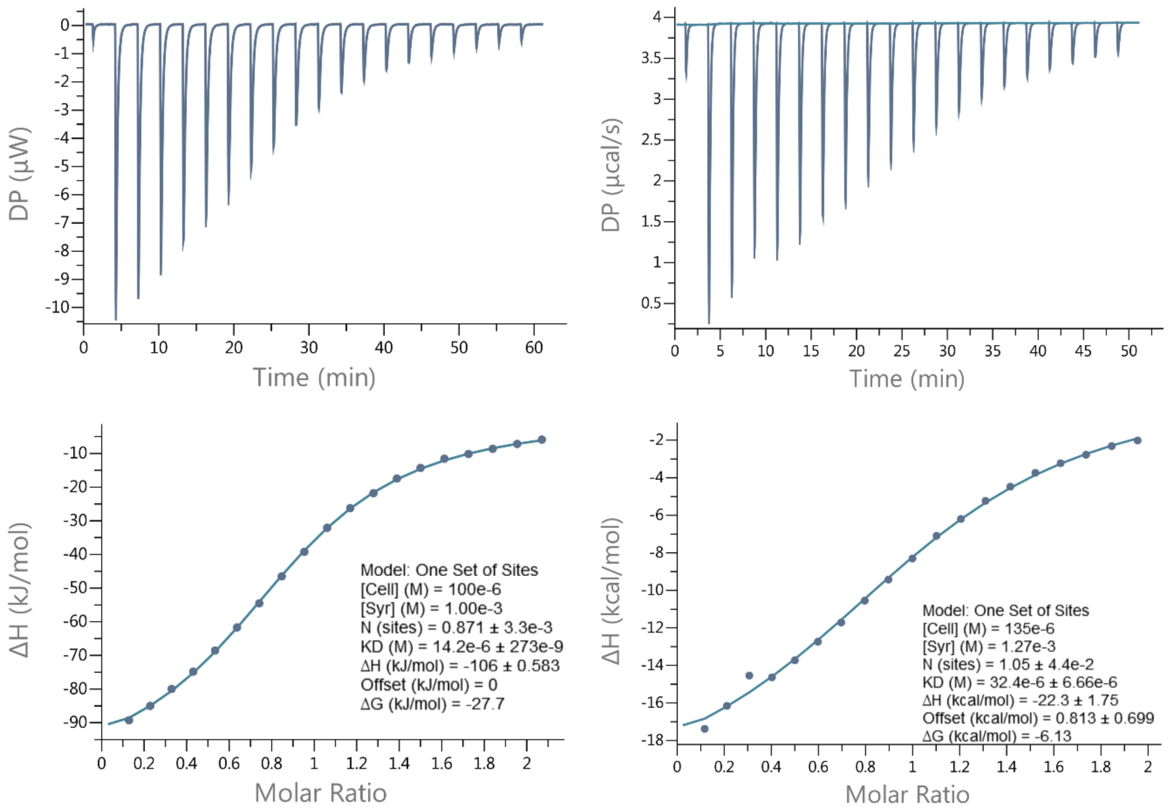

$H4_{1\text{-}20}(KAc)_4$	$H4_{1\text{-}20}(KAc)_3K12C$
K_D = 14.2 ± 0.3 μM	K_D = 32.4 ± 6.7 μM
ΔH = −106 ± 0.6 kJ/mol	ΔH = −93.3 ± 7.3 kJ/mol
$-T\Delta S$ = 78.3 kJ/mol	$-T\Delta S$ = 67.7 kJ/mol

* Errors in K_D and ΔH represent error in fitting of data.

For comparison, isotheral titration calorimetry (ITC) data for binding to BRD4(1) were obtained for the tetra-acetylated control peptide [$H4_{1\text{-}20}(KAc)_4$], and the K12C peptide modified for alkylation [$H4_{1\text{-}20}(KAc)_3K12C$]. A K_D value of 14.2 ± 0.3 μM was obtained for $H4_{1\text{-}20}(KAc)_4$, in good agreement with AlphaScreen IC_{50} values of 15.1 ± 1.7 μM. Titration of $H4_{1\text{-}20}(KAc)_3K12C$ showed the expected reduction in affinity to BRD4(1).

Supporting Figure S13: Deconvoluted masses from LCMS of H3K18C alkylation.

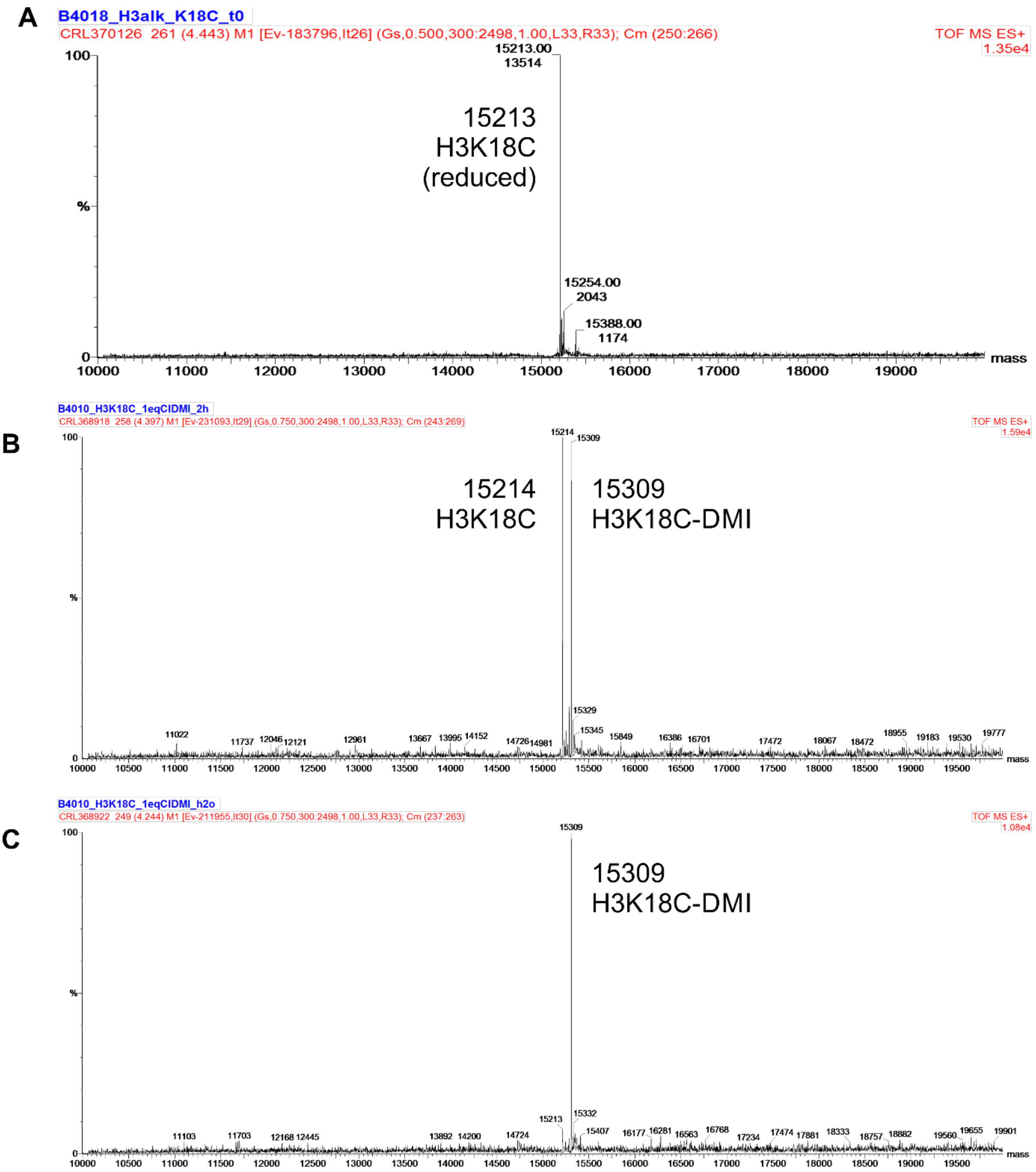

Deconvoluted masses (1.0 Da resolution, 10,000-20,000 Da range) from combined spectra across the protein region of LCMS chromatograms (around 4.3 min retention time). **A** H3K18C starting protein showing single species as β-ME adduct: calculated MW of reduced H3K18C 15213 Da + 76 Da for SC_2H_4OH. **B** Alkylation reaction after 1 h: reaction progression evident through observation of H3K18C (15214 Da) and H3K18C-DMI (15309 Da) protein species. **C** Alkylation reaction after 3 h and buffer exchange into H_2O: complete monoalkylation of protein resulted in single protein species corresponding to H3K18C-DMI (15309 Da).

Reaction conditions (final concentrations in 50 µL): 1 M CHES, 4 M guanidinium hydrochloride, 10 mM L-methionine, 5 mM TCEP, 330 µM H3K18C (from 8.5 mg/mL, 557 µM), 330 µM Cl-DMI (1 eq. solubilized in 20% DMSO in alkylating buffer to 10 mM for addition).

Supporting Table S1: Percentage inhibition of bromodomain-KAc recognition by isoxazole-containing amino acids.

Protein<br>Competitor peptide<br>L-amino acid	BRD4(1)<br>$H4(KAc)_4$<br>250 µM	 <br>50 µM	BAZ2A<br>H3K14Ac<br>250 µM	 <br>25 µM	BRD9<br>$H2(KAc)_3$<br>250 µM	 <br>25 µM
R = 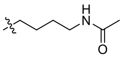 KAc	10.8	4.6	n.d	n.d	n.d	n.d
R = 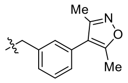 8	76.3	18.3	45.3	5.8	0.5	−7.7
R = 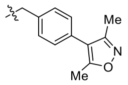 9	45.8	22.7	28.1	2.6	66.5	−1.7
R = 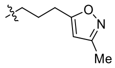 3	16.4	2.3	n.d	n.d	n.d	n.d
R = 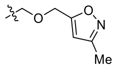 4	10.3	0.6	n.d	n.d	n.d	n.d
R = 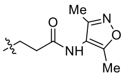 10	7.5	4.1	25.1	1.4	−3.1	−4.8
R = 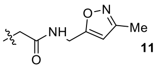 11	18.5	4.7	53.8	1.9	−0.1	−8.1
R = 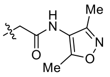 12	23.5	1.3	66.9	10.5	12.6	−2.1
R = 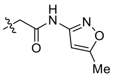 13	26.3	0.9	96.1	12.6	47.9	−1.0
R = 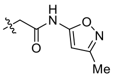 14	n.d	n.d	84.3	14.5	14.6	1.4
R = 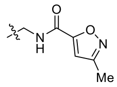 5	31.2	2.5	98.7	58.1	73.6	28.6

Inhibitory values of unprotected amino acids given as average percentage inhibition (buffer blank – 0% inhibition; 25% DMSO – 100% inhibition) against each bromodomain, from measurements in triplicate (AlphaScreen). n.d. value not determined. Inhibition values highlighted as a heatmap from green (low inhibition) to red (high inhibition). The phenol derivative (**2**) bearing a 3,5-dimethylisoxazole moiety, with an IC_{50} of 382 nM,[9] was included on each plate as a positive control, which in every case demonstrated 100% inhibition. Data for BAZ2A and BRD9 were obtained by Dr. Oleg Fedorov at the SGC. Several amino acids resulted in >50% inhibition at 250 µM, with significant inhibitory effect detected at lower concentrations of 25/50 µM. These findings represent an increase in binding affinity relative to that of KAc, which displayed negligible inhibition at 250 µM, and are the first unnatural free amino acids known to inhibit bromodomain recognition of an KAc-containing peptide. The data suggest that a three-atom linker (**3-5** & **12-14**) is long enough to allow the isoxazole to access the KAc pocket, and extended lengths are less well tolerated. (23.5% inhibition at 250 µM for three atom linker **12** against BRD4(1); 7.5% inhibition at 250 µM for four atom linker **10**). Amide connections demonstrated a higher percentage of BRD4(1) inhibition than alkyl or ether, with a preferred orientation of the carbonyl adjacent to the isoxazole (31.2% inhibition at 250 µM for **5**). The phenyl-linked isoxazole (**8**) showed the strongest inhibitory activity against BRD4(1) (>70% inhibition at 250 µM). Apparent selectivity over BRD9, might be due to increased rigidity, or the ability of the aromatic linker to interact with the hydrophobic region of the ZA channel and displace multiple, proximal KAc residues more effectively.

Supporting Table S2: Results from reaction condition optimization.

Variable	Condition	TCEP (eq)	Buffer (pH)	T (°C)	Cl-DMI (eq)	% ratio (120 min)
eq. of reducing agent	a	10	CHES (10)	25	1	41[b]
	b	5	CHES (10)	25	1	79
	c	2.5	CHES (10)	25	1	71
	d	0	CHES (10)	25	1	26
pH of reaction and buffer dependence	e	10	HEPES (7)	25	1	3
	f	10	HEPES (8)	25	1	n.d.
	g	10	CHES (8)	25	1	38
	h	10	CHES (9)	25	1	58
	a	10	CHES (10)	25	1	41[b]
	i	10	CAPS (10)	25	1	47[a]
	j	10	CAPS (11)	25	1	59
temperature	k	10	CHES (10)	4	1	17[b]
	a	10	CHES (10)	25	1	41[b]
	l	10	CHES (10)	42	1	78
eq of alkylating agent	m	10	CHES (10)	25	0.5	34
	a	10	CHES (10)	25	1	41[b]
	n	10	CHES (10)	25	2	74
	o	10	CHES (10)	25	5	92

n.d. value not determined; [a] ratio after 60 min; [b] ratio after 16 h.

Reaction mixture (20 µL) containing 1 mM H4(KAc)$_3$K12C, 1 M buffer, 4 M guanidine·hydrochloride, 10 mM L-methionine was prepared in buffer with TCEP and Cl-DMI as shown in the table. Percentage ratio of integrals of peaks corresponding to H4(KAc)$_3$K12C and H4(KAc)$_3$K12C(DMI): 100%×∫2350/(∫2350+∫2255). In every case, these were the highest intensity peaks in the spectra. Note that condition a) was selected as the locus for exhaustive local optimization, and is listed several times in the table for ease of comparison, with variations highlighted in grey.

Where no temperatures is stated, reactions were carried out at room temperature (rt).

Supporting Scheme S1: Synthesis of dimethylisoxazole trifluoroborate salt.

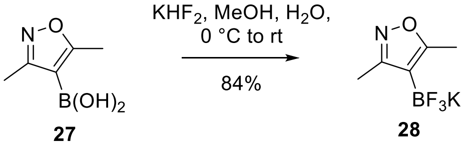

Supporting Scheme S2: Synthesis of *meta*-phenylalanine derived amino acid.

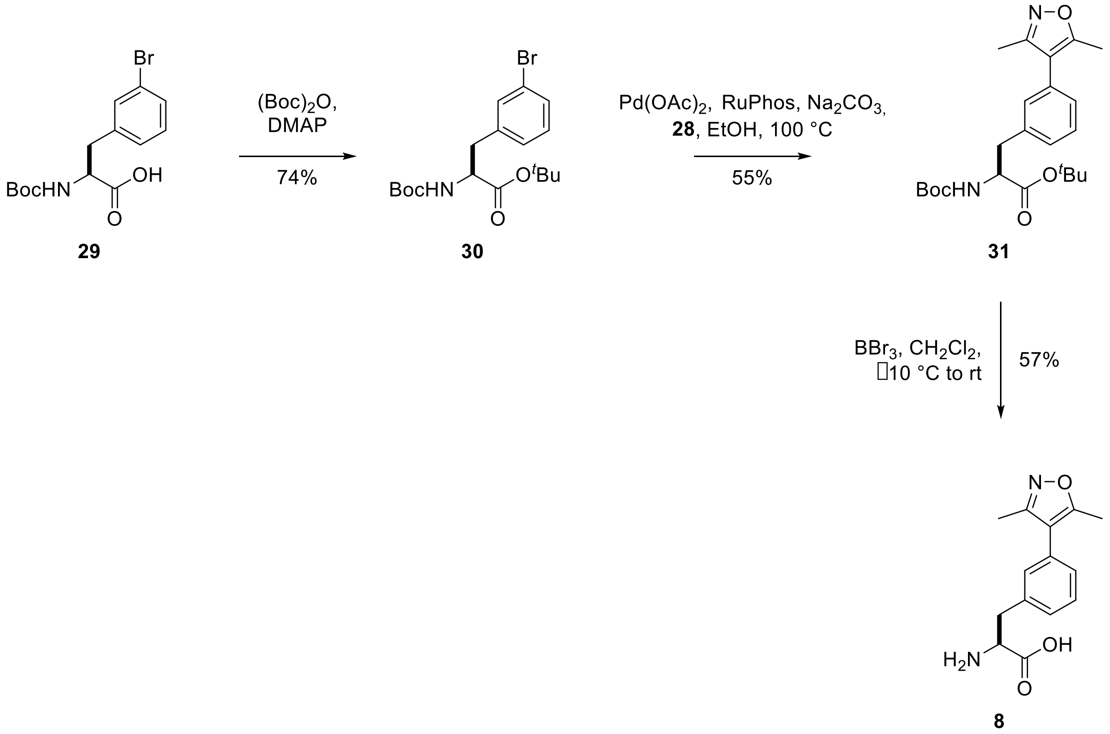

Supporting Scheme S3: Synthesis of *para*-phenylalanine derived amino acid.

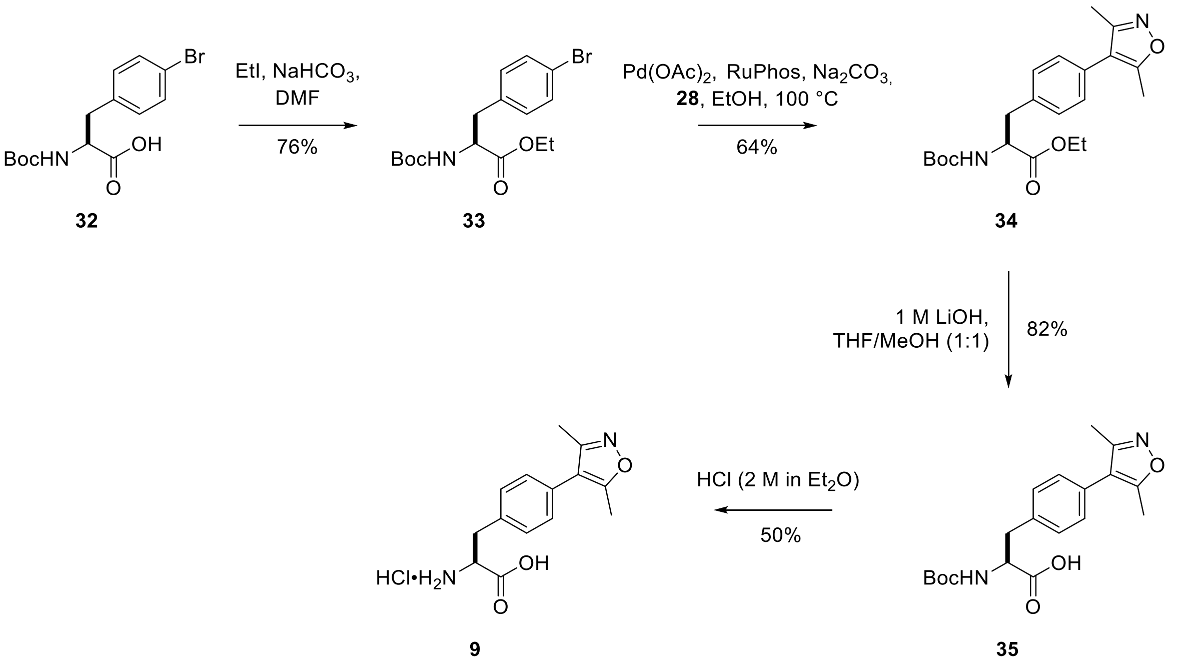

Supporting Scheme S4: Synthesis of isoxazole-containing extended electrophile.

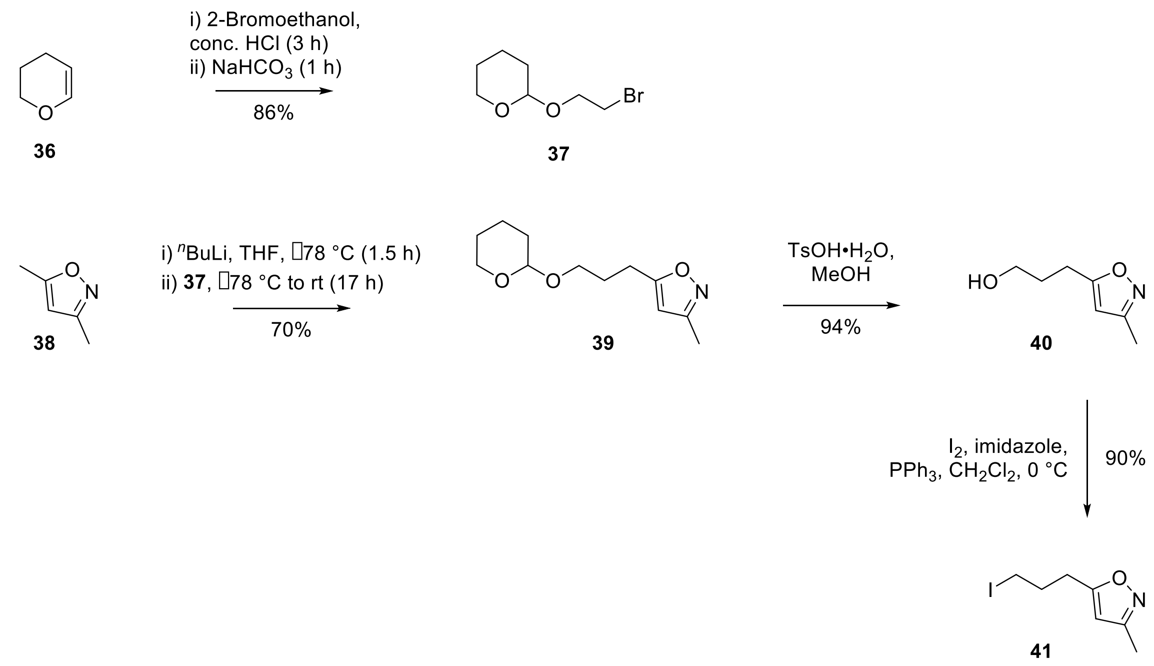

Supporting Scheme S5: Synthesis of alkyl coupled amino acid.

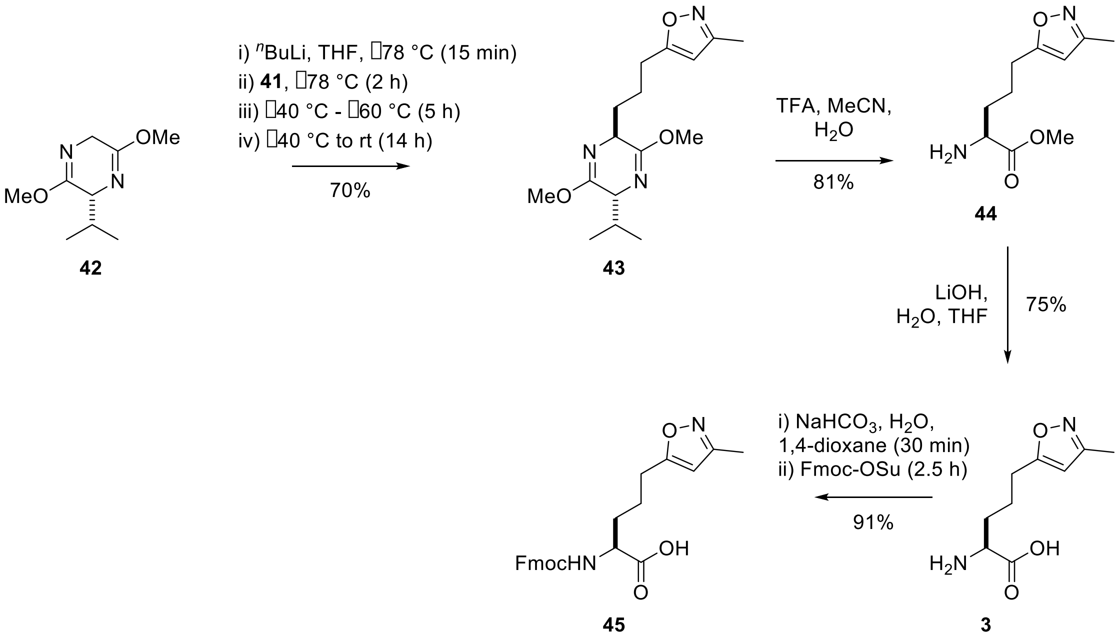

Supporting Scheme S6: Synthesis of isoxazole-containing bromine electrophile.

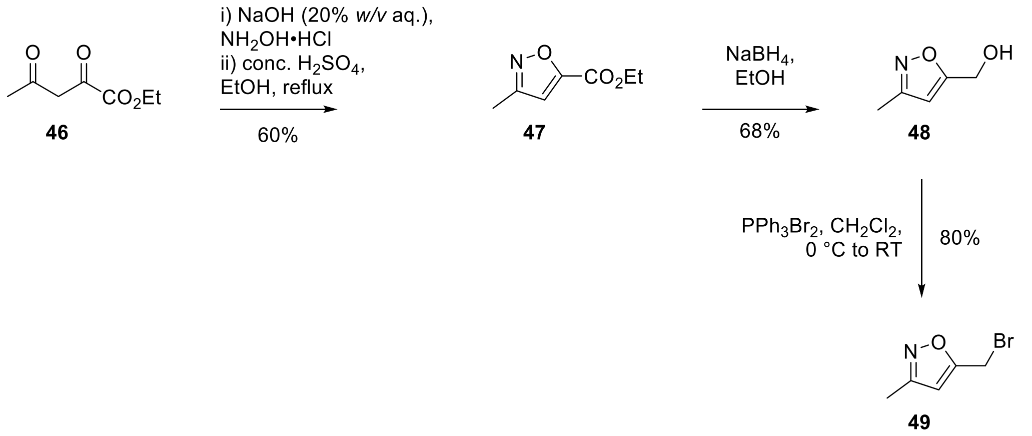

Supporting Scheme S7: Synthesis of isoxazole-containing chlorine electrophile.

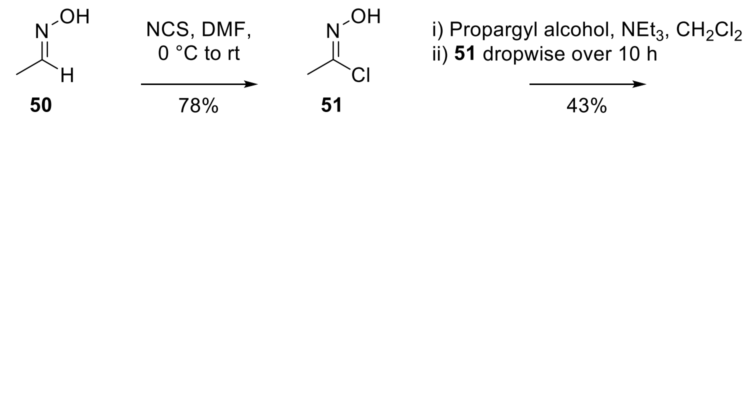

Supporting Scheme S8: Synthesis of ether coupled amino acid.

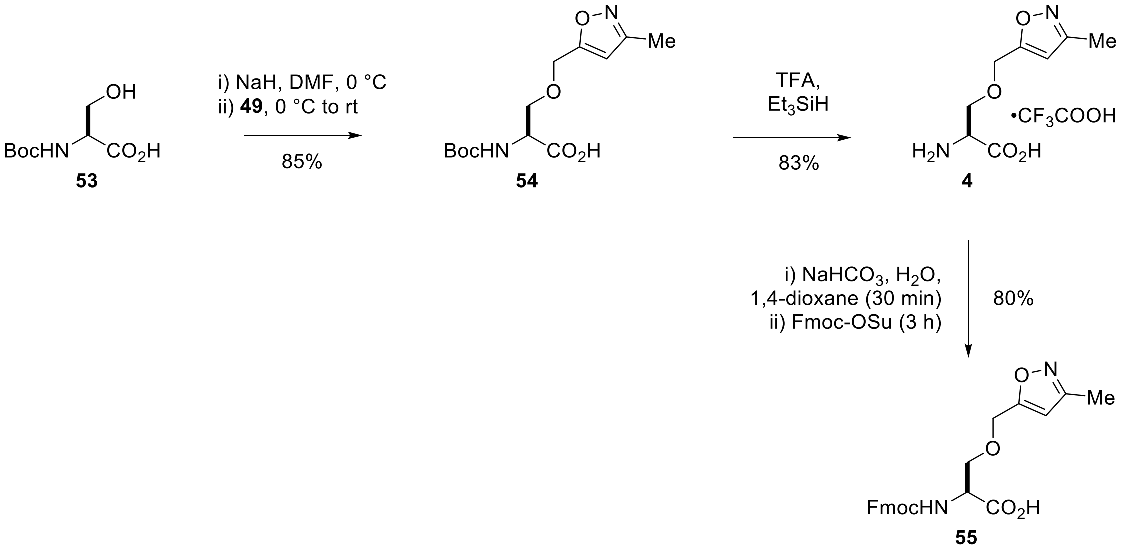

Supporting Scheme S9: Synthesis of glutamic acid derived amino acid.

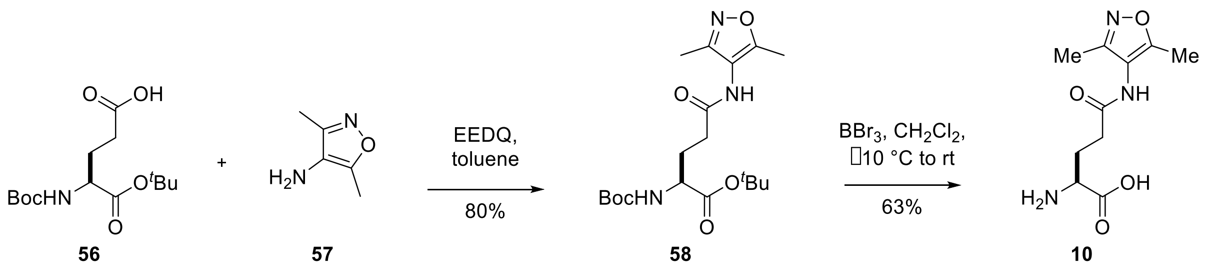

Supporting Scheme S10: Synthesis of aspartic acid derived amino acids.

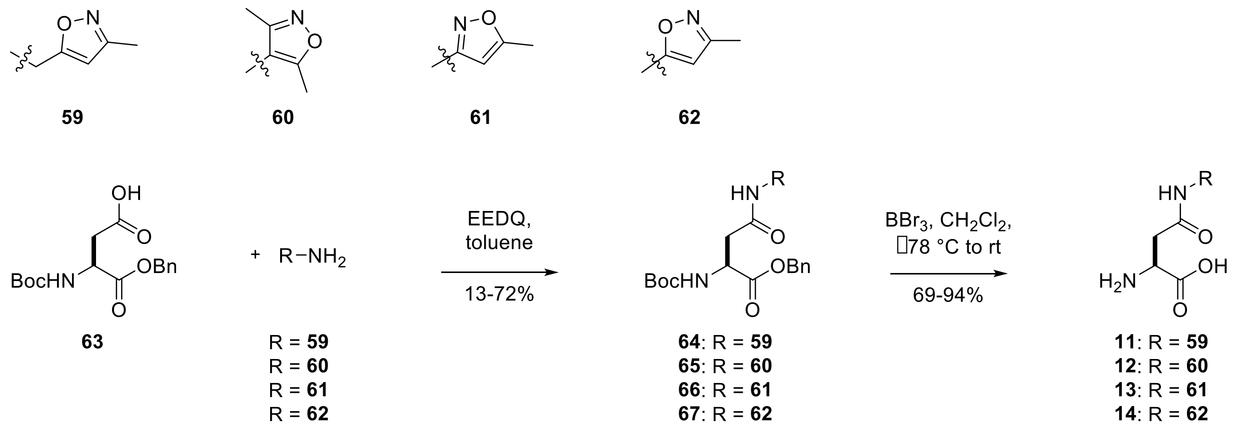

Supporting Scheme S11: Synthesis of *iso*-amide coupled amino acid.

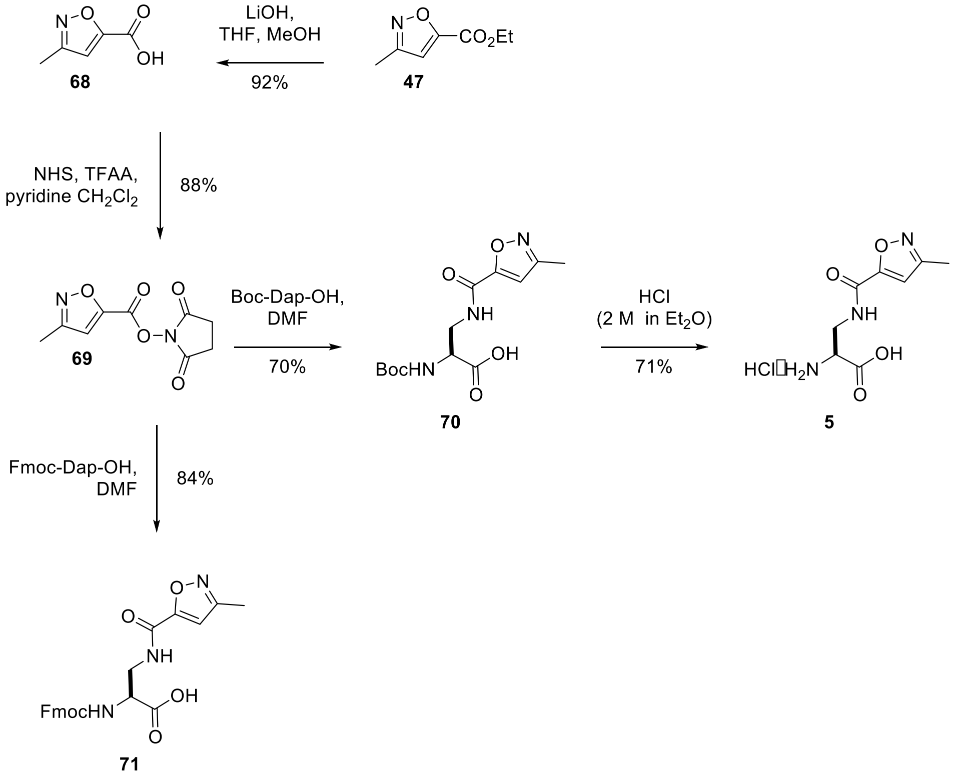

Supporting Scheme S12: Optimised alkylation of tripeptide.

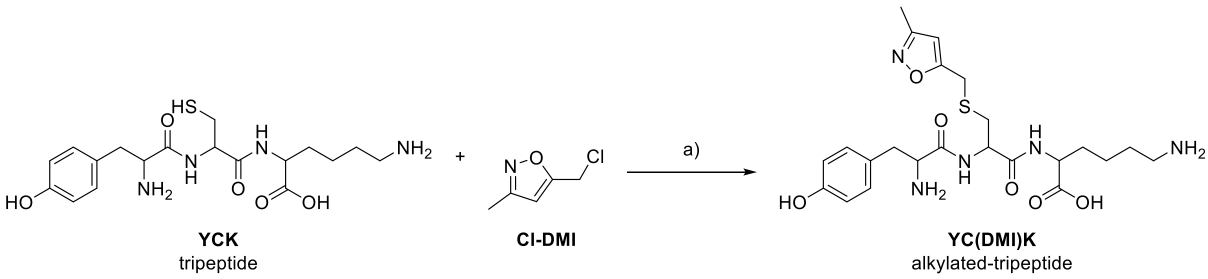

Reaction conditions: a) 1 M HEPES (pH 7.8), 10 mM L-methionine, 4 M guanidinium chloride, 12 mM YCK, 12 mM Cl-DMI, 85 mM TCEP, 20 °C, 1.5 h

Biochemical Methods

Peptide synthesis: in order to accurately quantify peptide concentrations by UV absorbance, an N-terminal tyrosine residue was included in all sequences (ε_{280} = 1280 $M^{-1}cm^{-1}$) This additional residue was also present in the $H4_{1\text{-}20}(KAc)_4$ sequence employed by Filippakopoulos *et al.* in ITC studies,[10] and had little apparent effect on affinity. $H4_{1\text{-}20}(KAc)_4$ [YSGRGK(Ac)GGK(Ac)GLGK(Ac)GGAK(Ac)RHRK-NH_2] was prepared on a CS Bio CS336X synthesiser using standard Fmoc SPSS techniques with double coupling, then purified by semi-preparative reverse phase HPLC to >95% purity (analytical HPLC at 220 nm). For validation of controls, this peptide was also synthesised by GL Biochem and purified to >95 % purity. Amino acids were incorporated into the $H4_{1\text{-}20}(KAc)_4$ sequence at each acetylation position: K5, K8, K12, and K16. $H4_{1\text{-}20}(KAc)_3$K5>**5** and $H4_{1\text{-}20}(KAc)_3$K8>**5** were prepared on a CS Bio CS336X synthesiser as above. All other isoxazole-containing peptide sequences were synthesised by ProteoGenix and purified to >95 % purity. $H4_{1\text{-}20}$(KAc)K12C [YSGRGK(Ac)GGK(Ac)GLGCGGAK(Ac)RHRK-NH_2] was synthesised by Activiotec and purified to >95 % purity.

Alkylation: reactions were performed in thin walled 500 µL PCR tubes, with a reaction volume of 20 µL. Buffers were degassed by sonication under argon immediately prior to use. TCEP was freshly prepared as a 100 mM stock in the corresponding buffer. Alkylating agents (Cl-DMI, *N*-methylmaleimide and 2-iodoacetomide) were accurately weighed and prepared as 20 mM stocks, by dissolving in DMSO (20% of final volume) before dilution into alkylation buffer. Time series commenced upon addition of the alkylating agent to a PCR tube containing all other reagents, pre-equilibrated to the temperature of interest. Optimised alkylation reaction conditions were: 1 M CHES (pH 9.0), 4 M guanidine hydrochloride, 10 mM L-methionine, 5 mM TCEP, 1 mM Y-$H4_{1\text{-}20}(KAc)_3$K12C peptide, 5 mM Cl DMI, 25 °C, 2 h. Under these conditions product was observed with 96% product/starting peptide integral ratio by MALDI-MS; analytical HPLC (C-18, 220 nm) retention time = 7.50 min, 77%.

High-performance liquid chromatography: samples from alkylation reactions were monitored by HPLC on a PerkinElmer Flexar system with a Binary LC Pump and UV/VIS LC Detector. 20 µL samples were loaded onto a pre-equilibrated reversed phase Dionex Acclaim® 120 column (C18, 5 µm, 120 Å, 4.6 × 150 mm), and eluted with a standard gradient elution of 0-100% B (A: 95% H_2O, 5% MeCN, 0.1% TFA; B: 5% H_2O, 95% MeCN, 0.1% TFA), at 1 mL/min over 10 min, monitoring absorption at 220 nm (absorption of peptide bonds).

Peaks corresponding to mono-alkylation were collected separately, and lyophilized, before resuspension for use in AlphaScreen assays. Collected samples were run on the same gradient to confirm isolated peptide was a single species.

Mass spectrometry: LCMS/MS was performed on a nano-QCT. With a standard elution gradient of 0-100% B (A: 95% H_2O, 5% MeCN, 0.1% TFA; B: 5% H_2O, 95% MeCN, 0.1% TFA). The extent of substrate alkylation was analysed by MALDI-TOF-MS using a Bruker Ultraflex instrument. Peptide samples were prepared in a 1:4 ratio from quenched reaction mixture (1:3 dilution of reaction into 95% H_2O, 4.5% acetonitrile, 0.5% formic acid) with CHCA matrix (20 mg/mL α-cyano-4-hydroxycinnamic adcid in 50% acetonitrile) and spotted onto an MTP 384 Massive target plate using the dried droplet method. The instrument was calibrated directly prior to data acquisition using mono-isotopic peptide masses with Peptide Calibration Standard II (Bruker Daltonics, Coventry, UK). Sample ionization was achieved with a N_2 laser (337 nm) at 35-50% laser energy and MS spectra were acquired by manual operation in reflectron mode: 2000-3000 data points were collected to make a total intensity of approximately 8×10^4 a.u. Alkylation sites were assigned unambiguously by MALDI-TOF/TOF mass spectrometry: MS/MS spectra were acquired by Laser-induced fragmentation (LIFT).[11] Predicted fragmentation masses were calculated using the Fragment Ion Calculator, as part of the Proteomics Toolkit from the Institute for Systems Biology, and compared manually to spectrum generated.

Protein expression and purification: bromodomain-containing constructs in pNIC28-Bsa4 vector backbones were transformed into *E. coli* BL21 (DE3) Gold cells. Flasks containing 600 mL of 2·TY media with the appropriate antibiotic were inoculated with 1% of the bacterial starter culture. Flasks (2000 mL PYREX® narrow-mouth graduated Erlenmeyer flasks or plastic baffled flasks) were incubated at 37 °C and 180 rpm in an Innova® 44 shaker (New Brunswick Scientific). When OD_{600} reached 0.8-1.0, the temperature was lowered to 18 °C and IPTG was added to a final concentration of 0.25 mM to induce protein expression. Cultures were incubated at 18 °C overnight, after which time cultures were harvested by centrifugation in a JA10 rotor in an Avanti J25 centrifuge (Beckman CoulterTM) at 8000 rpm for 15 min at 4 °C. The resulting pellet was weighed and stored in a sealable plastic bag at -80 °C. Proteins were purified with a 3 mL HisTrapTM chromatography column (GE Healthcare), and Superdex 75 XK 16/600 gel filtration column (GE Healthcare) using standard procedures. H3 construct contained cysteine substitutions (C96S, C110A), in addition to a K18C substitution, resulting in a single cysteine residue: amino-acid sequence ARTKQTARKSTGGKAPRCQLATKAARKSAPATGGVKKPHRYRPGTVALREIRRYQKSTELL IRKLPFQRLVREIAQDFKTDLRFQSSAVMALQEASEAYLVALFEDTNLAAIHAKRVTIMPKDI QLARRIRGERA.

AlphaScreen assay: AlphaScreen buffer (25 mM HEPES, 100 mM NaCl, 0.05% *w/v* CHAPS, 0.1% *w/v* BSA; pH 7.6) was prepared fresh each day, filter sterilization through a 0.22 μm filter, and stored at 4 °C, with equilibration to room temperature before use. Single shot screening against BAZ2A and BRD9A was carried out by the Structural Genomics Consortium (Oxford) as described previously.[12] Biotinylated peptides employed and final assay concentrations were: BRD9 40 nM, biotin-$H2A_{1\text{-}21}$KAc9KAc13KAc15 25 nM; BAZ2A 25 nM, biotin-$H3_{5\text{-}24}$KAc14 50 nM. Amino acids were tested at 25 μM and 250 μM with <0.5% DMSO. Inhibition is reported as a reduction in signal arising from peptide-bromodomain interaction, normalised to a DMSO control. Concentration response curves against BRD4(1) were performed in triplicate on a ProxiPlate-384 Plus (Perkin Elmer). The 3,5-dimethylisoxazole-

containing bromodomain ligand (**2**) was used as a positive control on each individual assay plate. This compound afforded IC_{50} values of 280 nM and 257 nM, which is in line with published values (IC_{50} 382 nM[9]). For incubation steps, the plate was sealed, shaken for 10 sec at 600 rpm, and incubated at room temperature in the dark for 1 h. Plates were read using a Synergy™ 2 MultiMode Microplate Reader using the built-in AlphaScreen 384 ProxiPlate function: excitation 680 nm, 0.18 sec; emission 570 nm, 0.37 sec. Final assay concentrations: BRD4(1) 10 nM; biotin-$H4_{1\text{-}20}$KAc5KAc8KAc12KAc16 4 nM; donor beads 5 µg/mL; acceptor beads 5 µg/mL.

Isothermal titration calorimetry: all calorimetric experiments were performed on a MicroCal iTC200 or MicroCal PEAQ-ITC (Malvern) and analysed with the MicroCal ORIGIN software package using a single binding site model. The first data point was excluded from the analysis. Proteins and peptides containing an *N*-terminal tyrosine were dialysed at room temperature overnight in a Slide-A-Lyzer™ MINI Dialysis Device (2000 MWCO; Thermo Scientific Life Technologies) into 50 mM HEPES, 500 mM NaCl; pH 7.5. Samples were centrifuged to remove aggregates (20 min, 14,000 rpm, 25 °C), and concentrations estimated by measuring the absorbance at 280 nm using a Nanodrop® ND-1000 spectrophotometer (Nanodrop® Technologies Inc.) with the 'Protein A280' program module according to the manufacturer's instructions (tyrosine Ɛ280 = 1280 $M^{-1}cm^{-1}$). Samples were diluted to the required concentration using dialysis buffer, and lyophilized histone peptides were dissolved in the same buffer. The cell was stirred at 750 rpm, with reference power set to 4 µcal/sec and temperature held at 298 K. After an initial delay of 60 sec, 19×2 µL injections (first injection 0.4 µL) were performed with a spacing of 180 sec. Heats of dilution were measured under the same conditions and subtracted for analysis. Protein solutions in the calorimetric cell (250 µL) were titrated with the peptide solutions in the syringe (60 µL) at the following concentrations: 100 µM BRD4(1) and 1.0 mM $H4_{1\text{-}20}(KAc)_4$; 135 µM BRD4(1) and 1.27 mM $H4_{1\text{-}20}(KAc)_3$K12C.

Synthetic Methods

Compound names are those generated by ChemBioDraw™ (CambridgeSoft) following IUPAC nomenclature.

Reagents and solvents used, unless otherwise stated, were of commercially available reagent grade quality and were used without further purification. Where appropriate and if not stated otherwise, all non-aqueous reactions were carried out in a flame dried flask under an inert atmosphere of nitrogen or argon. Anhydrous solvent was purchased from SigmaAldrich UK in SureSeal™ bottles, or dried according to the procedure outlined by Pangborn *et al.*,[13] and used without purification unless otherwise indicated. Solvents for use in organometallic reactions were degassed by three freeze-thaw cycles under vacuum and were stored under an argon atmosphere over 3 Å molecular sieves. Triethylamine for use in organometallic reactions was dried with KOH and distilled onto KOH pellets. *In vacuo* refers to solvent removal under reduced pressure using a Buchi™ rotary evaporator. Brine refers to a saturated aqueous solution of sodium chloride. Petroleum ether refers to the fraction of light petroleum ether boiling in the range 40-60 °C.

Analytical thin layer chromatography (TLC) was carried out on Merck silica gel 60 F_{254} aluminium supported thin layer chromatography sheets. Visualization was by absorption of UV light (λ_{max} 254 or 365 nm), or thermal development after dipping in one of: **a** ethanolic

solution of phosphomolybdic acid; **b** aqueous solution of potassium permanganate, potassium carbonate and sodium hydroxide; **c** ethanolic solution of ninhydrin.

Flash column chromatography was carried out either on Merck silica gel 60 (240-400 mesh), eluting with solvents as supplied under a positive pressure of compressed air, or on a Biotage SP1 system using KP-Sil™ cartridges.

Melting points were determined using a Kofler hot stage microscope and are uncorrected.

Specific optical rotations were measured using a Perkin-Elmer 241 or 341 polarimeter with a water-jacketed 1 dm path-length cell maintained at 20 °C. The light source was maintained at 589 nm. The concentration (c) is expressed in g/100 mL and specific rotations are denoted $[\alpha]_D$ with implied units of 10^{-1} deg cm^2 g^{-1}.

Infrared spectra were obtained as a thin film on sodium chloride discs or from neat samples using a diamond ATR module. The spectra were recorded on a Bruker Tensor 27 spectrometer and a representative number of absorption maxima are reported in wavenumbers (cm^{-1}). The intensity of each signal is indicated by: (w) weak; (m) medium; (s) strong; (br) broad.

^{1}H NMR spectra were recorded on Bruker DPX400, AVII400 or AVIII400 (400 MHz) and Bruker DRX500 or AVII500 with cryoprobe (126 MHz) spectrometers using deuterochloroform (unless indicated otherwise) as a reference for internal deuterium lock. The chemical shift data for each signal are given as δH in units of parts per million (ppm) relative to tetramethylsilane (TMS) where δH (TMS) = 0.00 ppm. The multiplicity of each signal is indicated by: s (singlet); br s (broad singlet); d (doublet); t (triplet); q (quartet); dd (doublet of doublets); dq (doublet of quartets); tt (triplet of triplets); qd (quartet of doublets); ddd (doublet of doublet of doublets); or m (multiplet); app (apparent). The number of protons (n) for a given resonance signal is indicated by nH. Coupling constants (*J*) are expressed in Hz and are recorded to the nearest 0.1 Hz. Identical proton coupling constants (*J*) are averaged in each spectrum and reported to the nearest 0.1 Hz. The coupling constants are determined using Bruker TopSpin software.

^{13}C NMR spectra were recorded on Bruker AVII400 or AVIII400 (101 MHz) and Bruker DRX500 or AVII500 with cryoprobe (126 MHz) spectrometers using broadband proton decoupling and an internal deuterium lock. The chemical shift data for each signal are given as δC in units of parts per million (ppm) relative to tetramethylsilane (TMS) where δC (TMS) = 0.0 ppm. Where appropriate, coupling constants (*J*) are expressed in Hz and are recorded to the nearest 0.1 Hz. ^{1}H and ^{13}C spectra were assigned using 2D NMR experiments including COSY, HSQC and HMBC.

Mass spectra were acquired on either a Micromass LCT Premier spectrometer, Agilent 6120 Quadrapole spectrometer or Bruker MicroTOF spectrometer using electrospray ionization, operating in positive or negative mode, from solutions of methanol. m/z values are reported in Daltons and followed by their percentage abundance in parentheses.

Elemental analyses were obtained by the microanalysis service of the London Metropolitan University, UK.

High-Performance Liquid Chromatography was carried out using a PerkinElmer Flexar system with a Binary LC pump (flow rate 0.6 mL/min) and UV/VIS LC detector. For determination of compound purity a Dionex Acclaim 120 column (C18, 5 μm, 120 Å, 4.6 × 150 mm) was used with the method described below (Table A); samples were injected in

$(CH_3)_2SO$ or $(CH_3)_2CHOH$. For determination of enantiomeric excess, a Daicel ChiralPak® (AD-H/AS-H, 5 µm 4.6 × 250 mm) column was used with an isocratic elution of 20:80 EtOH:H_2O or 30:70 IPA:Heptane; samples were injected in $(CH_3)_2CHOH$. Alternatively, an Astec CHIROBIOTIC™ T (TS, 5 µm 4.6 × 250 mm) column was used with an isocratic elution of 50:50 MeOH/0.1% NEt_3/0.1% AcOH and 0.1 M aqueous triethylammonium acetate buffer (pH 4.1); samples were injected in CH_3OH. Chromera software was used to determine purity and enantiomeric excess from relative peak areas of UV/VIS absorbance at 254 nm.

Table A Solvents: A = 95% H_2O/5% MeCN + 0.1% TFA; B = 95% MeCN/5% H_2O + 0.1% TFA.

Step length (min)	Elapsed time (min)	%A	%B
1	1	100	0
10	11	0	100
3	14	0	100
1	15	100	0
5	20	100	0

General Procedures

General Procedure 1 for coupling of isoxazole amines to aspartic acid

EEDQ (1.38 eq.) was added to a solution of Boc-Asp-*O*-Bn (1.0 eq.), and the corresponding amine (1.0 eq.) in toluene (0.43 mM) at rt and left to stir for the time indicated. The volatile components were removed *in vacuo*, and the resulting residue was redissolved in EtOAc (20 mL) and washed with citric acid (10% *w/v* aq., 3 × 20 mL), sat. aq. $NaHCO_3$ (20 mL), H_2O (20 mL), and brine (20 mL). The organic layer was dried ($MgSO_4$), filtered and concentrated *in vacuo* to give crude products which were subsequently purified.

General Procedure 2 for the deprotection of aspartic acid derived amino acids

To a solution of the corresponding Boc-, *O*-Bn protected amino acid (1.0 eq.) in anhydrous CH_2Cl_2 (0.07 mM) at –10 °C, BBr_3 (5.0 eq.) was added. The reaction mixture was left to stir at –10 °C for 1 h and then for a further duration at rt as indicated. The solution was quenched with H_2O (10-20 mL) and washed with EtOAc (5 × 10-20 mL). Purification by ion exchange chromatography (Dowex 50WX8, 100-200 mesh), washing with H_2O then eluting with 1 M aq. NH_4OH, followed by lyophilization, yielded unprotected isoxazole-containing amino acids.

Synthesis and Characterization of Compounds

3,5-Dimethyl-4-(trifluoroboranyl)isoxazole, potassium salt (28)

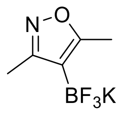

Following the procedure of Molander *et al.*,[14] to a suspension of 3,5-dimethylisoxazol-4-ylboronic acid **27** (2.50 g, 17.7 mmol, 1.0 eq) in MeOH (5 mL) at 0 °C was added KHF_2 (4.16 g, 53.2 mmol, 3.0 eq). H_2O (11.8 mL) was then added dropwise. The solution was allowed to warm to rt and stirred for 10 min, then concentrated and dried overnight *in vacuo*. The crude solid was purified by Soxhlet extraction (16 h) with acetone (100 mL). The collected solvent was concentrated *in vacuo*, and the residues were redissolved in the minimum amount of boiling acetone (400 mL). The product was precipitated by the addition of Et_2O (600 mL) and collected by filtration. The filtrate was concentrated *in vacuo*, redissolved in acetone (100 mL) and further product was precipitated by the addition of Et_2O (200 mL) and collected by filtration. The filtrate was again concentrated *in vacuo*, redissolved in acetone (30 mL) and further product was precipitated by the addition of Et_2O (60 mL) and collected by filtration. The combined solids were dried *in vacuo* to give **28** (3.02 g, 84%) as a powdery colorless solid. mp >275 °C (from acetone; lit. >200 °C); ^{1}H NMR (400 MHz, DMSO-D_6): δ 2.20 (s, 3H), 2.05 (s, 3H); ^{19}F NMR (470 MHz; DMSO-d_6): δ −134.8 - −134.2; ^{11}B NMR (160 MHz, DMSO-d_6): δ 2.33 (q, *J* 49 Hz); LRMS *m/z* (ES+) 164 ($[M-K]^-$, 100%); Anal. calcd for $C_5H_6BF_3KNO$: C 29.6%; H 3.0%; N 6.9%; Found: C 29.7%, H 2.9%, N 6.8%.

tert-Butyl (*S*)-3-(3-bromophenyl)-2-((*tert*-butoxycarbonyl)amino)propanoate (30)

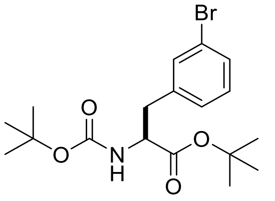

(2*S*)-3-(3-Bromophenyl)-2-[(*t*-butoxycarbonyl)amino]propanoic acid **29** (750 mg, 2.18 mmol, 1.0 eq), DMAP (73 mg, 0.65 mmol, 0.3 eq), $(Boc)_2O$ (523 mg, 2.40 mmol, 1.1 eq) and dry ^{t}BuOH (6 mL) were added to a dry flask under a N_2 atmosphere. The reaction mixture was stirred at rt for 1 h, then concentrated and dried overnight *in vacuo*. The mixture was dissolved in EtOAc (50 mL) and washed with citric acid (10% *w/v* aq., 3 × 50 mL), Na_2CO_3 (10% *w/v* aq., 3 × 50 mL), H_2O (50 mL), and brine (50 mL), dried ($MgSO_4$), filtered, and concentrated *in vacuo.* Purification *via* silica gel chromatography (gradient elution 2 to 20% Et_2O in petroleum ether) yielded **30** (646 mg, 74%) as a colorless solid. R_f 0.22 (1:9 Et_2O:petroleum ether); $[\alpha]_D$= +4.66 (*c* 1.0 in $CDCl_3$); mp 51-57 °C (from $CDCl_3$); ν_{max} (solid)/cm^{-1} 3390 (w), 3344 (w), 2980 (w), 2932 (w), 1738 (m), 1692 (m), 1513 (m), 1148 (s); ^{1}H NMR (500 MHz, $CDCl_3$) δ 7.37 (d, *J* 7.8 Hz, 1H), 7.33 (s, 1H), 7.17 (dd, *J* 7.8 7.8 Hz, 1H), 7.12 (d, *J* 7.7 Hz, 1H), 5.03 (d, *J* 6.8 Hz, 1H), 4.43 (dd, *J* 6.8 6.8 Hz, 1H), 3.10–2.98 (m, 2H), 1.44 (s, 9H), 1.42 (s, 9H); ^{13}C NMR (126 MHz, $CDCl_3$) δ 170.5, 155.0, 138.8, 132.6, 129.9, 129.9, 129.8, 122.3, 82.4, 79.8, 54.7, 38.1, 28.3, 27.9; LRMS *m/z* (ES+) 400 ($[M(^{79}Br)+H]^+$, 65%), 402 ($[M(^{81}Br)+H]^+$, 60%), 422 ($[M(^{79}Br)+Na]^+$, 95%), 424 ($[M(^{81}Br)+Na]^+$, 100%); HRMS *m/z* (ES+) [Found $(M+Na)^+$ 422.0936, 424.0919. $C_{18}H_{26}{}^{79}BrNO_4Na^+$ requires 422.0937, $C_{18}H_{26}{}^{81}BrNO_4Na^+$ requires

424.0918]; Anal. calcd for $C_{18}H_{26}BrNO_4$: C 54.0%, H 6.6%, N 3.5%; Found: C 54.2%, H 6.6%, N 3.6%.

tert-Butyl (_S_)-2-((_tert_-butoxycarbonyl)amino)-3-(3-(3,5-dimethylisoxazol-4-yl)phenyl)propanoate (31)

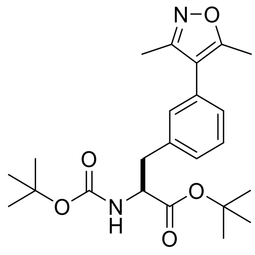

30 (212 mg, 0.53 mmol, 1.0 eq), **28** (118 mg, 0.58 mmol, 1.1 eq), palladium acetate (6 mg, 0.03 mmol, 0.05 eq), RuPhos (5 mg, 0.01 mmol, 0.02 eq) and Na_2CO_3 (112 mg, 1.06 mmol, 2.0 eq) were added to a dry 5-10 mL microwave vial. The vial was sealed, evacuated and purged with argon (3 × evacuate/fill). Degassed EtOH (4 mL) was added, and the reaction was heated at 100 °C for 24 h. The reaction mixture was filtered through silica (eluent CH_2Cl_2, 100 mL) and concentrated *in vacuo*. Purification *via* silica gel chromatography (gradient elution 2 to 20% EtOAc in petroleum ether) yielded **31** (121 mg, 55%) as yellow oil. R_f 0.16 (1:4 EtOAc:petroleum ether); $[\alpha]_D$= +2.66 (*c* 1.0 in $CDCl_3$); ν_{max}/cm^{-1} 3390 (w), 3342 (w), 2980 (w), 2932 (w), 1738 (m), 1692 (s), 1513 (m), 1148 (s); ^1H NMR (500 MHz; $CDCl_3$): δ 7.36 (dd, *J* 7.6 Hz, 1H), 7.17 (d, *J* 7.6 Hz, 1H), 7.13 (d, *J* 7.6 Hz, 1H), 7.07 (s, 1H), 5.03 (d, *J* 7.4 Hz, 1H), 4.49 (dd, *J* 7.4 7.4 Hz, 1H), 3.17-3.03 (m, 2H), 2.41 (s, 3H), 2.27 (s, 3H), 1.41 (s, 9H), 1.4 (s, 9H); ^{13}C NMR (126 MHz; $CDCl_3$): δ 170.8, 165.2, 158.6, 155, 137.1, 130.4, 130.3, 128.7, 127.5, 125.5, 116.4, 82.2, 79.8, 54.7, 38.4, 28.3, 27.9, 11.5, 10.8; LRMS *m/z* (ES+) 855 ($[2M+Na]^+$, 100%); HRMS *m/z* (ES+) [Found: $(M+Na)^+$ 439.2205. $C_{23}H_{32}N_2O_5Na^+$ requires 439.220].

(_S_)-2-Amino-3-(3-(3,5-dimethylisoxazol-4-yl)phenyl)propanoic acid (8)

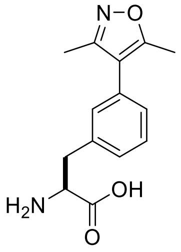

31 (377 mg, 0.91 mmol, 1.0 eq) was dissolved in anhydrous CH_2Cl_2 (25 mL) under N_2 and cooled to -10 °C. BBr_3 (1.14 g, 0.44 mL, 4.53 mmol, 5.0 eq) was added dropwise over 5 min with stirring. After 1 h the reaction was allowed to warm to rt and stirred for a further 2 h, then quenched with cold H_2O (10 mL). The layers were separated, and the organic layer was extracted with H_2O (5 × 20 mL). The combined aq. layers were lyophilized. The crude product was suspended in HCl (2 M in Et_2O, 10 mL) and concentrated under a stream of N_2 to form the HCl salt, which was purified by ion exchange chromatography and lyophilized to afford **8** (135mg, 57%) as a colorless hygroscopic powder. $[\alpha]_D$= –0.18 (*c* 0.5 in D_2O); ν_{max}/cm^{-1} 2970 (br), 1739 (m), 1626 (s), 1485 (s), 1382 (s), 1231 (s); ^1H NMR (500 MHz; D_2O): δ 7.41 (dd, *J* 7.6 7.6 Hz, 1H), 7.26-7.19 (m, 2H), 7.16 (s, 1H), 3.94-3.87 (m, 1H), 3.22-3.04 (m, 2H), 2.29 (s, 3H), 2.15 (s, 3H); ^{13}C NMR (126 MHz; $CDCl_3$): δ 173.6, 166.7, 160, 135.7, 130.5, 129.9,

129.5, 128.6, 128.3, 116.1, 55.9, 36.2, 10.7, 9.8; LRMS *m/z* (ES+) 543 ($[2M+Na]^+$, 100%); HRMS *m/z* (ES+) [Found: $(M+Na)^+$ 283.1064. $C_{14}H_{16}N_2O_3Na^+$ requires 283.1053]; HPLC (AS-H; 20:80 EtOH:H_2O, 254 nm) retention time = 7.84 min, ee 86.4%.

Ethyl (*S*)-3-(4-bromophenyl)-2-((*tert*-butoxycarbonyl)amino)propanoate (33)

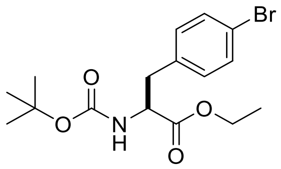

EtI (1.81 g, 11.63 mmol, 5.0 eq.) was added to a solution of *N*-Boc-4-bromo-L-phenylalanine **32** (800 mg, 2.33 mmol, 1.0 eq.), and $NaHCO_3$ (391 mg, 4.65 mmol, 2.0 eq.) in anhydrous DMF (12 mL). The reaction mixture was stirred at rt for 24 h. The volatile components were removed *in vacuo*, and the resulting residue was redissolved in EtOAc (20 mL), washed with H_2O (3 × 20 mL) and brine (20 mL), dried ($MgSO_4$), filtered and concentrated *in vacuo* to give **33** (660 mg, 76%) as a yellow solid without need for further purification. R_f 0.90 (1:9 MeOH:EtOAc); mp 83-84 °C (from EtOAc); $[\alpha]_D$ +38.0 (*c* 1.0 in $CHCl_3$); ν_{max} (thin film)/cm^{-1} 3365 (w), 2979 (w), 1593 (s), 1489 (s), 1165 (s); ^1H NMR (500 MHz; $CDCl_3$): δ 7.42 (d, *J* 8.4 Hz, 2H), 7.02 (d, *J* 8.4 Hz, 2H), 4.99 (d, *J* 7.8 Hz, 1H), 4.58-4.51 (m, 1H), 4.17 (q, *J* 7.2 Hz, 2H), 3.08 (dd, *J* 13.8 5.8 Hz, 1H), 3 (dd, *J* 13.8 6.0 Hz, 1H), 1.43 (s, 9H), 1.25 (t, *J* 7.2 Hz, 3H); ^{13}C NMR (126 MHz; $CDCl_3$): δ 171.6, 155, 135.1, 131.5, 131.1, 120.9, 80, 61.5, 54.2, 37.82, 28.3, 14.1; LRMS *m/z* (ES+) 394 ($[^{79}M+Na]^+$, 94%), 396 ($[^{81}M+Na]^+$, 97%), 767 ($[^{79}M+^{81}M+Na]^+$, 100%); HRMS *m/z* (ES+) [Found: $(M+Na)^+$ 394.0622. $C_{16}H_{22}BrNNaO_4^+$ requires 394.0624]; Anal. calcd for $C_{16}H_{22}BrNO_4$: C 51.6%, H 5.9%, N 3.8%; Found: C 51.6%, H 5.9%, N 3.7%.

Ethyl (*S*)-2-((*tert*-butoxycarbonyl)amino)-3-(4-(3,5-dimethylisoxazol-4-yl)phenyl)propanoate (34)

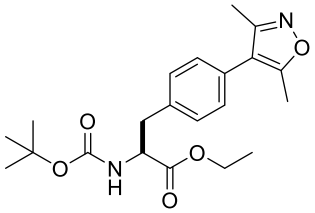

To a 10-20 mL microwave vial were added palladium acetate (4 mg, 0.015 mmol, 0.01 eq.), RuPhos (14 mg, 0.03 mmol, 0.02 eq.), anhydrous Na_2CO_3 (307 mg, 2.90 mmol, 2.0 eq.), **33** (540 mg, 1.45 mmol, 1.0 eq.), and **28** (306 mg, 1.51 mmol, 1.04 eq.). The vial was sealed, evacuated and purged with argon (3 × evacuate/fill). Degassed EtOH (8 mL) was added and the reaction was left to stir at 100 °C for 24 h. The reaction mixture was cooled to rt, filtered through a thin pad of silica (eluent: CH_2Cl_2), and concentrated *in vacuo*. Purification *via* silica gel chromatography (gradient elution 5 to 100% Et_2O in petroleum ether) yielded **34** (363 mg, 64%) as a yellow oil. R_f 0.78 (1:9 MeOH:EtOAc); $[\alpha]_D$ +10.9 (*c* 1.0 in $CHCl_3$); ν_{max}/cm^{-1} 2979 (w), 1714 (s), 1392 (s), 1166 (s); ^1H NMR (500 MHz; $CDCl_3$): δ 7.23 (d, *J* 8.1 Hz, 2H), 7.18 (d, *J* 8.1 Hz, 2H), 5.05 (d, *J* 5.1 Hz, 1H), 4.62-4.58 (m, 1H), 4.19 (q, *J* 7.1 Hz, 2H), 3.16 (dd, *J* 13.9 6.0,Hz, 1H), 3.07 (dd, *J* 13.9 6.1,Hz, 1H), 2.4 (s, 3H), 2.26 (s, 3H), 1.43 (s, 9H), 1.23 (t, *J* 7.1 Hz, 3H); ^{13}C NMR (126 MHz; $CDCl_3$): δ 171.8, 165.1, 158.6, 155, 135.6, 129.8, 116.2, 79.9, 61.4, 54.5, 38.3, 28.3, 14.1, 11.6, 10.8; LRMS *m/z* (ES+) 298 ($[M+Na]^+$, 100%); HRMS

m/z (ES+) [Found: $(M+Na)^+$ 411.1891. $C_{21}H_{28}N_2NaO_5^+$ requires 411.1890]; Anal. calcd for $C_{21}H_{28}N_2O_5$: C 64.9%, H 7.3%, N 7.2%; Found: C 64.8%, H 7.2%, N 7.2%; HPLC (AD-H; 30:70 IPA:Heptane, 254 nm) retention time = 8.77 min, ee 76.8%.

(*S*)-2-((*tert*-Butoxycarbonyl)amino)-3-(4-(3,5-dimethylisoxazol-4-yl)phenyl)propanoic acid (35)

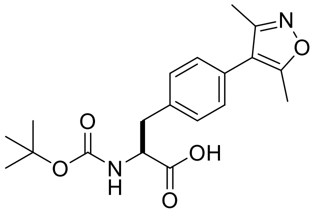

Aqueous LiOH (1 M, 1.31 mL, 1.31 mmol, 5.0 eq.) was added to a suspension of **34** (100 mg, 0.26 mmol, 1.0 eq.) in THF (12 mL) and MeOH (12 mL). After 20 h the volatile components were removed *in vacuo* and the resultant solid was redissolved in H_2O (5 mL) and acidified to pH 2 using 1 M aq. HCl. The aqueous phase was extracted with EtOAc (3 x 10 mL) and combined organic layers were washed with brine (10 mL), dried ($MgSO_4$), filtered and concentrated *in vacuo* to give **35** (77 mg, 82%) as a colorless solid without need for further purification. R_f 0.36 (1:9 MeOH:EtOAc); mp 69-70 °C (from EtOAc); $[\alpha]_D$ +9.6 (*c* 1.0 in $CHCl_3$); ν_{max}/cm^{-1} 3324 (br), 1713 (s), 1165 (s), 732 (s); ^1H NMR (400 MHz; $CDCl_3$): δ 7.28 (d, *J* 9.1 Hz, 2H), 7.21 (d, *J* 9.1 Hz, 2H), 4.99 (d, *J* 7.9 Hz, 1H), 4.69-4.61(m, 1H), 3.28 (dd, *J* 13.9 5.4 Hz, 1H), 3.11 (dd, *J* 13.9 7.2 Hz, 1H), 2.41 (s, 3H), 2.28 (s, 3H), 1.43 (s, 9H); ^{13}C NMR (126 MHz; $CDCl_3$): δ 175.7, 165.3, 158.7, 155.34, 135.4, 129.9, 129.2, 129.1, 116.3, 80.3, 54.2, 37.7, 28.3, 11.6; LRMS *m/z* (ES+) 743 ($[2M+Na]^+$, 100%); HRMS *m/z* (ES+) [Found: $(M+Na)^+$ 383.1572. $C_{19}H_{24}N_2NaO_5^+$ requires 383.1577]; Anal. calcd for $C_{19}H_{24}N_2O_5$: C 63.3%, H 6.7%, N 7.8%; Found: C 63.4%, H 6.6%, N 7.8%.

(*S*)-2-Amino-3-(4-(3,5-dimethylisoxazol-4-yl)phenyl)propanoic acid (9)

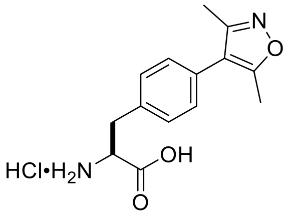

HCl (2 M in Et_2O, 1.5 mL) was added to **35** (77 mg, 0.21 mmol, 1.0 eq.) and the resulting mixture was stirred at rt. After 30 h the mixture was concentrated *in vacuo* and the resulting residue was triturated with Et_2O to give the HCl salt of **9** (31 mg, 50%) as a colorless solid. R_f 0.45 (RP, 1:1 MeOH:H_2O); mp 144-145 °C (from Et_2O); $[\alpha]_D$ –0.2 (*c* 0.5 in H_2O); ^1H NMR (400 MHz; D_2O): δ 7.29 (d, *J* 8.3 Hz, 2H), 7.25 (d, *J* 8.3 Hz, 2H), 4.17 (dd, *J* 7.7 5.7 Hz, 1H), 3.25 (dd, *J* 15.5 5.7 Hz, 1H), 3.12 (dd, *J* 15.5 7.7 Hz, 1H), 2.25 (s, 3H), 2.11 (s, 3H); LRMS *m/z* (ES+) 261 ($[M+H]^+$, 100%); HPLC (TS, 80:20 EtOH:H_2O, 254 nm) retention time = 9.59 min, ee 77.4%.

2-(2-Bromoethoxy)tetrahydro-*2H*-pyran (37)

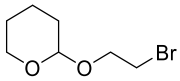

Following the procedure of Anantanaryan *et al.*,[15] to a vigorously-stirred mixture of 2-bromoethanol **36** (7.9 mL, 13.9 g, 110 mmol, 1.0 eq) and 3,4-dihydro-2*H*-pyran (10.0 mL, 9.2 g, 110 mmol, 1.0 eq) at 0 °C was added 1 drop of conc. HCl. The reaction mixture was allowed to warm to rt and stirred for 3 h. Solid $NaHCO_3$ (3 g) was added and the suspension was stirred for 1 h then filtered. The product was purified by vacuum distillation to give **37** as a colorless oil (19.8 g, 86%) which was stored under argon over 3 Å molecular sieves in the dark at –20 ˚C prior to use. R_f 0.53 (1:1 Et_2O:petroleum ether); bp 84 °C (9 mbar) [lit. 82 °C (0.5 mmHg)][15]; ^{1}H NMR (400 MHz; $CDCl_3$) δ 4.71-4.66 (m, 2H), 4.07-3.98 (m, 1H), 3.96-3.86 (m, 1H), 3.82-3.74 (m, 1H), 3.48 (m, 3H), 1.94-1.48 (m, 6H); LRMS *m/z* (ES+) 209 ($[M+H]^+$, 100%). These data are in good agreement with literature values.[15]

3-Methyl-5-(3-((tetrahydro-*2H*-pyran-2-yl)oxy)propyl)isoxazole (39)

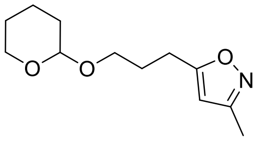

To a solution of 3,5-dimethylisoxazole **38** (5.0 mL, 5.0 g, 51 mmol, 1.0 eq) in distilled anhydrous THF (100 mL) at –78 °C under an argon atmosphere was added ^{n}BuLi (2.3 M in hexanes, 23.3 mL, 53 mmol, 1.05 eq) over 20 min. After 1.5 h, a solution of distilled **37** (7.7 mL, 10.7 g, 51 mmol, 1.0 eq) in THF (15 mL) was added over 30 min *via* cannula. The solution was allowed to warm to rt slowly over 17 h, then quenched with H_2O (100 mL) and concentrated *in vacuo* to remove THF. The residue was extracted with Et_2O (3 × 100 mL), and the combined organic layers were washed with H_2O (300 mL) and brine (300 mL), dried ($MgSO_4$), filtered and concentrated *in vacuo*. Purification by silica gel column chromatography (20% then 40% Et_2O/petroleum ether, 0.5% NEt_3) gave **39** as a pale yellow oil (8.08 g, 70%). R_f 0.27 (2:3 Et_2O:petroleum ether); ν_{max}/cm^{-1} 2871 (m), 1605 (m), 1418 (m), 1136 (m), 1120 (m), 1034 (s); ^{1}H NMR (500 MHz; acetone-d_6): δ 6.04 (s, 1H), 4.62-4.57 (m, 1H), 3.84-3.72 (m, 2H), 3.50-3.39 (m, 2H), 2.86-2.80 (m, 2H), 2.21 (s, 3H), 2.00-1.90 (m, 2H), 1.87-1.76 (m, 1H), 1.71-1.61 (m, 1H), 1.60-1.44 (m, 4H); ^{13}C NMR (126 MHz; acetone-d_6): δ 173.6, 160.1, 102.3, 99.2, 66.6, 62.2, 31.4, 28.6, 26.3, 24.0, 20.1, 11.3; LRMS *m/z* (ES+) 226 ($[M+H]^+$, 69%), 248 ($[M+Na]^+$, 100%); HRMS *m/z* (ES+) [Found: $(M+Na)^+$ 248.1265. $C_{12}H_{19}NNaO_3^+$ requires 248.1257]. These data are in good agreement with the literature values.[16]

3-(3-Methylisoxazol-5-yl)propan-1-ol (40)

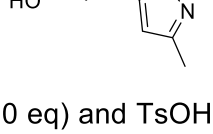

A solution of **39** (7.46 g, 33.1 mmol, 1.0 eq) and $TsOH{\cdot}H_2O$ (315 mg, 1.66 mmol, 0.05 eq) in MeOH (150 mL) was stirred for 2 h at rt. After TLC analysis indicated complete consumption of **39**, Celite® was added and the mixture was concentrated *in vacuo*. Purification *via* silica gel chromatography (gradient elution 20 to 30% acetone in petroleum ether) yielded **40** as a pale yellow oil (4.38 g, 94%). R_f 0.21 (3:7 acetone:petroleum ether); ^{1}H NMR (400 MHz; $CDCl_3$): δ 1.82-1.73 (m, 1H), 2.01-1.90 (m, 2H), 2.28 (s, 3H), 2.85 (t, *J* 7.6 Hz, 2H), 3.78-3.68 (m, 2H),

5.86 (s, 1H); LRMS m/z (ES+) 142 ([M+H]$^+$, 93%), 164 ([M+Na]$^+$, 100%). These data are in good agreement with literature values.[16–18]

5-(3-Iodopropyl)-3-methylisoxazole (41)

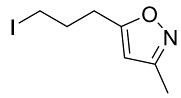

To a solution of PPh_3 (2.42 g, 9.21 mmol, 1.3 eq) and imidazole (627 mg, 9.21 mmol, 1.3 eq) in anhydrous CH_2Cl_2 (30 mL) at 0 °C under an argon atmosphere was added iodine (2.34 g, 9.21 mmol, 1.3 eq) in portions. After 15 min, a solution of **40** (1.00 g, 7.08 mmol, 1.0 eq) in CH_2Cl_2 (5 mL) was added and the reaction was stirred for 2 h at 0 °C, after which time TLC analysis indicated complete consumption of **40**. The reaction was diluted with hexane (70 mL) and filtered through Celite® (eluent hexane), then concentrated *in vacuo* at rt in the dark. The residue was then extracted with hexane and loaded onto a short silica plug, then eluted with 20% Et_2O in petroleum ether to give **41** as a pale yellow oil (1.59 g, 90%) which was stored in the dark at –20 °C prior to use. (The compound darkened if stored in the light). R_f 0.24 (1:5 Et_2O:petroleum ether); v_{max} (thin film)/cm^{-1}: 2931 (m), 1606 (s), 1447 (m), 1416 (s), 1259 (m), 1208 (m); ^{1}H NMR (500 MHz; $CDCl_3$): δ 5.88 (s, 1H), 3.21 (t, *J* 6.8 Hz, 2H,), 2.86 (t, *J* 7.2 Hz, 2H,), 2.27 (s, 3H), 2.22-2.14 (m, 2H); ^{13}C NMR (126 MHz; $CDCl_3$): δ 171.0, 159.8, 102.3, 30.8, 27.3, 11.4, 5.0; LRMS *m/z* (ES+) 252 ([M+H]$^+$, 100%); HRMS *m/z* (ES+) [Found: (M+Na)$^+$ 273.9695. $C_7H_{10}INNaO^+$ requires 273.9699].

5-(3-((2*S*,5*R*)-5-Isopropyl-3,6-dimethoxy-2,5-dihydropyrazin-2-yl)propyl)-3-methylisoxazole (43)

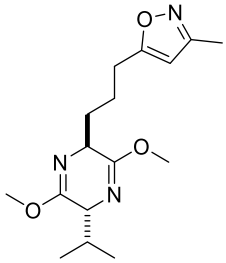

To a solution of (*R*)-2-isopropyl-3,6-dimethoxy-2,5-dihydropyrazine [(*R*)-**42**, 1.90 mL, 1.96 g, 10.6 mmol, 1.0 eq] in distilled anhydrous THF (28 mL) at –78 °C under an argon atmosphere was added ^{n}BuLi (2.3 M in hexanes, 4.8 mL, 11.1 mmol, 1.05 eq) dropwise. After 15 min, a precooled solution of **41** (2.66 g, 10.6 mmol, 1.0 eq) in THF (7.5 mL) was added dropwise *via* cannula. The reaction mixture was stirred at –78 °C for 2 h, then between –40 °C and –60 °C for 5 h, then allowed to warm to rt slowly over 14 h. The reaction was quenched with H_2O (50 mL) and phosphate buffer (pH 7, 1 M, 50 mL). The mixture was concentrated *in vacuo* to remove THF, then extracted with Et_2O (4 × 100 mL). The combined organic layers were washed with brine (400 mL), dried ($MgSO_4$), filtered and concentrated *in vacuo*. Purification *via* repeated silica gel column chromatography (consecutive elution 15%, 20%, 35% Et_2O in petroleum ether, then repurification of mixed fractions with 7% EtOAc in petroleum ether) gave (2*S*,5*R*)-**43** as a pale yellow oil (2.28 g, 70%). R_f 0.14 (3:7 Et_2O:petroleum ether); $[\alpha]_D$ –1.5 (*c* 1.0 in $CHCl_3$); v_{max} (thin film)/cm^{-1}: 2871 (m), 1692 (s), 1436 (m), 1235 (s), 1196 (m), 1007 (m); ^{1}H NMR (500 MHz; $CDCl_3$): δ 5.81 (s, 1H), 4.06-4.01 (m, 1H), 3.95 (t, *J* 3.5 Hz, 1H), 3.70 (s, 3H), 3.68 (s, 3H), 2.72 (t, *J* 7.6 Hz, 2H), 2.30-2.22 (m,[overlapping], 1H), 2.26 (s, 3H), 1.94-1.85 (m, 1H), 1.82-1.73 (m, 1H), 1.73-1.58 (m, 2H), 1.05 (d, *J* 6.8 Hz, 3H), 0.70 (d, *J* 6.8 Hz,

3H); ^{13}C NMR (126 MHz; $CDCl_3$): δ 173.1, 163.8, 163.4, 159.6, 101.4, 60.8, 55.0, 52.4, 52.4, 33.4, 31.8, 26.5, 22.7, 19.0, 16.6, 11.4; LRMS *m/z* (ES^+) 308 ($[M+H]^+$, 100%), 330 ($[M+Na]^+$, 15%); HRMS *m/z* (ES+) [Found: $(M+Na)^+$ 330.1779. $C_{16}H_{25}N_3NaO_3^+$ requires 330.1788].

Methyl (*S*)-2-amino-5-(3-methylisoxazol-5-yl)pentanoate (44)

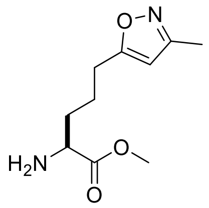

To a solution of (2*S*,5*R*)-**43** (2.23 g, 7.25 mmol) in MeCN (22 mL) and H_2O (7 mL) was added TFA (3.5 mL) and the reaction mixture was stirred at rt. After 1.5 h, TLC analysis indicated complete consumption of **43**, and the mixture was concentrated *in vacuo*. The residues were dissolved in CH_2Cl_2 (25 mL), and basified to pH 8 with sat. aq. $NaHCO_3$. The layers were separated and the aqueous layer was extracted with CH_2Cl_2 (3 × 25 mL). The combined organic layers were washed with brine (100 mL), dried (Na_2SO_4), filtered and concentrated *in vacuo*. Purification *via* silica gel chromatography (3:97 MeOH:CH_2Cl_2) followed by removal of remaining methyl valinate *in vacuo* yielded **44** as a yellow oil (1.24 g, 81%). R_f 0.23 (1:19 MeOH:CH_2Cl_2); $[\alpha]_D$ +12.3 (*c* 1.0 in $CHCl_3$); v_{max}/cm^{-1} 3379 (br), 2953 (m), 1733 (s), 1605 (m), 1437 (m), 1418 (m), 1198 (m), 1173 (m); ^1H NMR (500 MHz; $CDCl_3$): δ 1.69-1.5 (m, 3H), 1.86-1.71 (m, 3H), 2.26 (s, 3H), 2.73 (t, *J* 7.1 3H), 3.46 (dd, *J* 7.5 4.5 1H), 3.72 (s, 3H), 5.82 (s, 1H); ^{13}C NMR (126 MHz; $CDCl_3$): δ 176.2, 172.5, 159.7, 101.6, 54.1, 52.0, 34.1, 26.3, 23.7, 11.4; LRMS *m/z* (ES+) 213 ($[M+H]^+$, 100%); HRMS *m/z* (ES+) [Found: $(M+Na)^+$ 235.1046. $C_{10}H_{16}N_2NaO_3^+$ requires 235.1053]; HPLC (C-18, 220 nm) retention time = 7.84 min, 93.1%.

(*S*)-2-Amino-5-(3-methylisoxazol-5-yl)pentanoic acid (3)

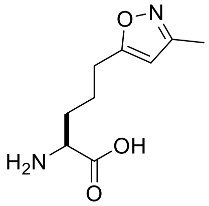

To a solution of (*S*)-**44** (1.24 g, 5.84 mmol) in THF (30 mL) was added a solution of LiOH (168 mg, 7.01 mmol, 1.2 eq) in H_2O (6 mL) and the reaction was stirred at rt. After 2 h, TLC analysis indicated complete consumption of **44**, and the mixture was acidified to pH 5 with aq. HCl (1 M). The resultant precipitate was filtered, washed with a small amount of ice-cold H_2O, ice-cold THF and Et_2O and dried in a vacuum desiccator to give (*S*)-**3** as a colorless hygroscopic solid (870 mg, 75%). $[\alpha]_D$ +4.1 (*c* 1.0 in $CHCl_3$); v_{max}/cm^{-1} 2596 (br), 1604 (s), 1580 (s), 1509 (s), 1409 (s), 1325 (m); ^1H NMR (500 MHz; D_2O): δ 6.03 (s, 1H), 3.66 (app t, *J* 6.0 Hz, 1H), 2.73 (t, *J* 7.2 Hz, 2H), 2.16 (s, 3H), 1-88-1.59 (m, 4H); ^{13}C NMR (126 MHz; D_2O): δ 174.5, 173.2, 161.7, 102.5, 54.4, 29.7, 25.3, 22.4, 10.3; LRMS *m/z* (ES+) 199 ($[M+H]^+$, 100%); HRMS *m/z* (ES+) [Found: $(M+Na)^+$ 221.0898. $C_9H_{14}N_2NaO_3^+$ requires 221.0897].

(*S*)-2-(((9*H*-Fluoren-9-yl)methoxy)carbonyl)amino)-5-(3-methylisoxazol-5-yl)pentanoic acid (45)

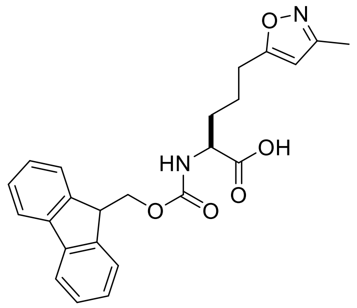

To a suspension of (*S*)-**3** (863 mg, 4.35 mmol, 1.0 eq) in H_2O (40 mL) and 1,4-dioxane (20 mL) was added $NaHCO_3$ (731 mg, 8.70 mmol, 2.0 eq). After 30 min, Fmoc-OSu (1.54 g, 4.57 mmol, 1.05 eq) in 1,4-dioxane (12 mL) was added dropwise over 15 min. After 2.5 h, TLC analysis indicated complete consumption of **3**, and the reaction was acidified to pH 2 with aq. HCl (1 M), extracted with EtOAc (4 × 50 mL), washed with citric acid (10% *w/v* aq., 200 mL), H_2O, (3 × 200 mL) and brine (200 mL), dried (Na_2SO_4), filtered and concentrated *in vacuo*. Purification *via* repeated crystallization from the minimum amount of boiling 2:1 hexane:CH_2Cl_2 gave (*S*)-**45** as a colorless solid (1.67 g, 91%). mp 92-94 °C (from 2:1 hexane:CH_2Cl_2); $[\alpha]_D$ –7.5 (*c* 1.0 in MeOH); ν_{max}/cm^{-1} 3347 (br), 2961 (w), 1682 (s), 1528 (s), 1250 (s); ^1H NMR (500 MHz; DMSO-d_6): δ 12.50 (br, 1H), 7.94-7.86 (m, 2H), 7.77-7.61 (m, 3H), 7.45-7.38 (m, 2H), 7.36-7.28 (m, 2H), 6.10 (s, 1H), 4.35-4.19 (m, 3H), 4.04-3.94 (m, 1H), 2.78-2.63 (m, 2H), 2.19 (s, 3H), 1.85-1.52 (m, 4H); ^{13}C NMR (126 MHz; DMSO-d_6): δ 173.7, 172.4, 159.3, 156.2, 143.8, 143.8, 140.7, 140.7, 127.6, 127.1, 125.3, 125.3, 120.1, 120.1, 101.9, 65.6, 53.4, 46.6, 30.1, 25.4, 23.7, 11.0; LRMS *m/z* (ES+) 443 ($[M+Na]^+$, 100%), 421 ($[M+H]^+$, 97%); HRMS *m/z* (ES+) [Found: $(M+Na)^+$ 443.1580. $C_{24}H_{24}N_2NaO_5^+$ requires 443.1577]; HPLC (C-18, 220 nm) retention time = 12.37 min, 99.2%; (TS, 254 nm) retention time = 10.0 min, ee 76.1%.

Ethyl 3-methylisoxazole-5-carboxylate (47)

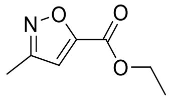

Following the procedure of Lepage *et al.*,[19] to an aq. solution of NaOH (20% *w/v*, 20 mL) was added hydroxylamine hydrochloride (9.04 g, 130 mmol, 1.0 eq). The solution was cooled to 0 °C and ethyl 2,4-dioxopentanoate **46** (20.6 g, 130 mmol, 1.0 eq) was added. The solution was allowed to warm to rt and stirred for 1 h, after which time TLC analysis indicated complete consumption of diketone. The solution was extracted with Et_2O (3 × 100 mL), and the combined organic extracts were washed with brine (200 mL), dried (Na_2SO_4), filtered and concentrated *in vacuo* at rt to give 19.4 g of yellow solid. This crude oxime was dissolved in EtOH (150 mL), and conc. aq. H_2SO_4 (2.4 mL) was added dropwise. The solution was heated under reflux for 4.5 h, then further conc. H_2SO_4 (0.5 mL) was added and the solution heated under reflux for a further 4 h. The reaction was cooled to rt and concentrated *in vacuo*. The residues were dissolved in Et_2O (250 mL), and washed with H_2O (250 mL) and brine (250 mL), dried ($MgSO_4$) and concentrated *in vacuo*. Purification by Kugelrohr distillation (90 °C, 4 mbar [lit. 122 °C, 20 mmHg][19]) gave an 87:13 mixture of desired ethyl 3-methylisoxazole-5-carboxylate (**47**) to its presumed regioisomer, ethyl 5-methylisoxazole-3-carboxylate, as a colorless solid which partially melted at rt (13.3 g, 69%). Data for ethyl 3-methylisoxazole-5-

carboxylate **47**: R_f 0.24 (1:9 Et_2O:petroleum ether); mp 26–27 °C (lit.[20] 27–28 °C); ^1H NMR (500 MHz; $CDCl_3$): δ 6.78 (s, 1H), 4.42 (q, *J* 7.1 Hz, 2H), 2.37 (s, 3H), 1.40 (t, *J* 7.1 Hz, 3H); LRMS *m/z* (ES+) 178 ($[M+Na]^+$, 100%). These data are in good agreement with the literature values.[21]

N-Hydroxyacetimidoyl chloride (50)

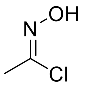

To a stirred solution of acetaldoxime (3.00 g, 50.8 mmol, 1.0 eq) in DMF (54 mL) at 0 °C, was added *N*-chlorosuccinimide (8.82 g, 66.1 mmol, 1.3 eq). The reaction mixture was allowed to warm to rt and stirred for 3 h. The pale green solution was diluted with brine (120 mL), extracted with Et_2O (2 × 100 mL), washed with H_2O (3 × 80 mL), dried ($MgSO_4$), filtered, and concentrated *in vacuo* to afford **50** as a cyan blue oil (6.53 g, 1:1 product:Et_2O, 40 mol, 78%) which was used without further purification, shortly after preparation. ^1H NMR (400 MHz, $CDCl_3$) δ_H 9.86 (br s, 1H), 2.18 (s, 3H). Further data could not be collected due to instability, however, the data presented are in good agreement with the literature values.[22]

(3-Methylisoxazol-5-yl)methanol (48)

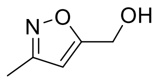

Method A

To a solution of **47** (2.00 g, 12.9 mmol, 1.0 eq) in anhydrous EtOH (25 mL) at 0 °C under an argon atmosphere was added $NaBH_4$ (585 mg, 15.5 mmol, 1.2 eq). The reaction was allowed to warm to rt and stirred for 7 h, after which time TLC analysis indicated complete consumption of **47**. The reaction was quenched with H_2O (20 mL) and aq. HCl (1 M, 10 mL), and concentrated *in vacuo* to remove EtOH. The residues were extracted with EtOAc (3 × 30 mL), and the combined organic layers were washed with brine (100 mL), dried ($MgSO_4$), filtered and concentrated *in vacuo*. Purification *via* silica gel chromatography (gradient elution 60 to 70% Et_2O in petroleum ether) yielded **48** as a pale yellow oil (989 mg, 68%).

Method B

To a stirred solution of propargyl alcohol (420 μL, 404 mg, 7.21 mmol, 1.0 eq) in CH_2Cl_2 (8.0 mL) protected from light, NEt_3 was added (1.2 mL, 871 mg, 8.61 mmol, 1.2 eq). A solution of **51** (842 mg, 9.05 mmol, 1.3 eq) in CH_2Cl_2 (8.0 mL) was added dropwise over 7 h using a syringe pump. The reaction was stirred for a further 8 h at rt. The reaction mixture was washed with H_2O (100 mL) and brine (100 mL), dried ($MgSO_4$), filtered and concentrated *in vacuo*. Purification *via* silica gel chromatography (gradient elution 30 to 70% Et_2O in petroleum ether) yielded **48** as a yellow oil (350 mg, 43%):

R_f 0.28 (2:3 Et_2O:petroleum ether); ν_{max}/cm^{-1} 3356 (br), 2934 (w), 2872 (w), 1610 (s), 1419 (s), 1279 (w), 1203 (w), 1134 (m), 1067 (s); ^1H NMR (400 MHz; $CDCl_3$): δ 6.10 (s, 1H), 4.72 (d, *J* 6.3 Hz, 2H), 3.32 (t, *J* 6.3 Hz, 1H), 2.29 (s, 3H); ^{13}C NMR (126 MHz; $CDCl_3$): δ 171.6, 159.8, 102.4, 55.8, 11.1; LRMS *m/z* (ES+) 136 ($[M+Na]^+$, 100%). These data are in good agreement with literature values.[23]

5-(Bromomethyl)-3-methylisoxazole (49)

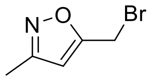

To a solution of **48** (793 mg, 7.01 mmol, 1.0 eq) in anhydrous CH_2Cl_2 (25 mL) at 0 °C under an argon atmosphere was added PPh_3Br_2 (3.55 g). The reaction was stirred in the dark for 1.5 h at 0 °C then for 1.5 h at rt, after which time TLC analysis indicated complete consumption of **48**. Most CH_2Cl_2 was removed *in vacuo*, then hexane (10 mL) was added to induce precipitation of PPh_3O. The solution was decanted off, and the residues extracted with hexane. The combined extracts were passed through a plug of silica gel (eluent 20% Et_2O in petroleum ether) and concentrated *in vacuo* to give **49** as a colorless oil (986 mg, 80%) which was stored in the dark at –20 °C prior to use (the compound darkened if stored in the light). R_f 0.21 (1:5 Et_2O:petroleum ether); ^{1}H NMR (400 MHz; $CDCl_3$): δ 6.18 (s, 1H), 4.46 (s, 2H), 2.33 (s, 3H); LRMS *m/z* (ES^+) 176 ($[^{79}M+H]^+$, 100%), 178 ($[^{81}M+H]^+$, 93%). These data are in good agreement with literature values.[17]

5-(Chloromethyl)-3-methylisoxazole (52: Cl-DMI)

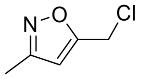

Freshly prepared (3-methylisoxazol-5-yl)methanol **48** (1.00 g, 8.85 mmol, 1.0 eq) was dissolved in thionyl chloride (12.8 mL, 21.0 g, 177 mmol, 20 eq) and the solution heated to reflux under an argon atmosphere for 20 min. The reaction mixture was allowed to cool to rt, and concentrated *in vacuo*, to yield **52** as a dark brown oil (1.11 g, 8.50 mmol, 96%) that was stored at −80 °C, and used without further purification. R_f 0.20 (1:4 MeOH:CH_2Cl_2); ν_{max} (thin film)/cm^{-1}: 1609 (s), 1411 (s); ^{1}H NMR (400 MHz $CDCl_3$) δ_H 6.09 (s, 1H), 4.51 (s, 2H), 2.24 (s, 3H); ^{13}C NMR (101 MHz, $CDCl_3$) δ_C 166.9, 159.9, 104.3, 34.8, 11.3; HRMS *m/z* (EI/FI) [Found: $(M+Cl^{35})^+$ 131.0138. $C_5H_6(^{35}Cl)NO^+$ requires 131.0138]. These data are in good agreement with the literature values.[24]

***N*-(*tert*-Butoxycarbonyl)-*O*-((3-methylisoxazol-5-yl)methyl)-L-serine (54)**

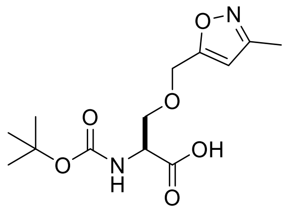

To a solution of anhydrous *N*-(*tert*-butoxycarbonyl)-L-serine **53** (1.03 g, 5.08 mmol, 1.0 eq) in anhydrous DMF (15 mL) at 0 °C under an argon atmosphere was added NaH (60% in mineral oil, 508 mg, 12.7 mmol, 2.5 eq) portionwise, and the mixture was stirred at 0 °C for 30 min. **49** (938 mg, 5.33 mmol, 1.05 eq) in anhydrous DMF (5 mL) was then added, and the reaction was allowed to warm to rt slowly over 13 h. DMF was then removed *in vacuo* and the residues were diluted with H_2O (150 mL) and washed with Et_2O (3 × 100 mL). The aqueous layer was acidified to pH 2 with aq. citric acid (10% *w/v*) and extracted with EtOAc (3 × 150 mL). The combined EtOAc extracts were washed with H_2O (450 mL) and brine (2 × 450 mL), dried (Na_2SO_4), filtered and concentrated *in vacuo* to give **54** as a pale brown gum (1.30 g, 85%)

which was used without further purification. $[\alpha]_D$ +7.8 (*c* 1.0 in $CHCl_3$); v_{max}/cm^{-1} 3331 (br), 2934 (m), 1710 (s), 1509 (m), 1368 (m), 1163 (s); ^{1}H NMR (500 MHz; $CDCl_3$): δ 4.54-4.45 (m, 1H), 7.40 (br, 1H), 6.11 (s, 1H), 5.45 (d, *J* 7.9 Hz, 1H), 4.61 (s, 2H), 4.00 (dd, *J* 9.3 2.3 Hz, 1H), 3.80 (dd, *J* 9.3 3.1 Hz, 1H), 2.31 (s, 3H), 1.47 (s, 9H); ^{13}C NMR (126 MHz; $CDCl_3$): δ 174.2, 168.1, 159.9, 155.7, 104.1, 80.5, 70.6, 64.1, 53.8, 28.3, 11.4; LRMS *m/z* (ES+) 323 ($[M+Na]^+$, 100%); HRMS *m/z* (ES+) [Found: $(M+Na)^+$ 323.1226. $C_{13}H_{20}N_2NaO_6^+$ requires 323.1214]; HPLC (C-18, 220 nm) retention time = 10.26 min, 93.1%.

***O*-((3-Methylisoxazol-5-yl)methyl)-L-serine, trifluoroacetic acid salt (4)**

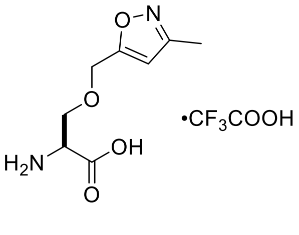

To a solution of **54** (1.18 g, 3.93 mmol, 1.0 eq) in CH_2Cl_2 (14 mL) was added Et_3SiH (1.88 mL, 1.37 g, 11.8 mmol, 3.0 eq) and TFA (7 mL). After 3.5 h at rt, TLC analysis indicated complete consumption of **54**, and the mixture was concentrated *in vacuo*, dissolved in 60 mL H_2O, washed with Et_2O (4 × 60 mL) and lyophilized to give **3** as an hygroscopic colorless solid (1.02 g, 83%) which was used without further purification. $[\alpha]_D$ –1.0 (*c* 1.0 in H_2O); v_{max}/cm^{-1} 3116 (br), 2931 (w), 1605 (s), 1556 (m), 1417 (m), 1354 (m), 1138 (m), 1092 (s); ^{1}H NMR (500 MHz; methanol-d_4): δ 6.36 (s, 1H), 4.70 (s, 2H), 4.05 (dd, *J* 4.7 4.7 Hz, 1H), 3.97 (d, *J* 4.7 Hz, 2H), 2.30 (s, 3H); ^{13}C NMR (126 MHz; methanol-d_4): δ 168.8, 168.2, 161.6, 160.1, 116.8, 104.2, 68.2, 63.2, 53.6, 9.7; ^{19}F NMR (470 MHz; methanol-d_4): δ –70.78; LRMS *m/z* (ES+) 201 ($[M–CF_3COO+H]^+$, 100%); HRMS *m/z* (ES+) [Found: $(M–CF_3COO+H)^+$ 201.0877. $C_8H_{13}N_2O_4^+$ requires 201.0875].

***N*-(((9*H*-Fluoren-9-yl)methoxy)carbonyl)-*O*-((3-methylisoxazol-5-yl)methyl)-L-serine (55)**

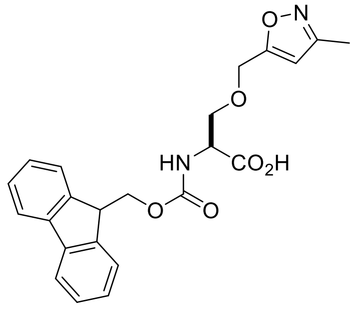

To a solution of **4** (1.00 g, 3.18 mmol, 1.0 eq) in H_2O (20 mL) and 1,4-dioxane (10 mL) was added $NaHCO_3$ (801 mg, 9.54 mmol, 3.0 eq). After 30 min, Fmoc-OSu (1.13 g, 3.34 mmol, 1.05 eq) in 1,4-dioxane (10 mL) was added dropwise over 15 min. After 3 h, TLC analysis indicated complete consumption of **4**, and the reaction was acidified to pH 2 with aq. HCl (1 M), extracted with EtOAc (4 × 80 mL), washed with citric acid (10% *w/v* aq., 250 mL), H_2O (2 × 250 mL) and brine (2 × 250 mL), dried (Na_2SO_4), filtered and concentrated *in vacuo*. Purification *via* repeated crystallization from the minimum amount of boiling 1:1 pentane:CH_2Cl_2 gave **55** as an off-white solid (1.08 g, 80%). mp 110-111 °C (1:1 pentane:CH_2Cl_2); $[\alpha]_D$ +4.1 (*c* 1.0 in MeOH); v_{max}/cm^{-1} 3312 (br), 2950 (w), 1720 (s), 1512 (m), 1450 (m), 1205 (m), 1107 (m); ^{1}H NMR (400 MHz; $CDCl_3$): δ 9.20 (br s, 1H), 7.82-7.7 (m, 2H), 7.66-7.56 (m, 2H), 7.45-7.35 (m, 2H), 7.35-7.27 (m, 2H), 6.08 (s, 1H), 5.72 (d, *J* 8.5 Hz, 1H),

4.67-4.5 (m, 3H), 4.49-4.32 (m, 2H), 4.23 (t, *J* 7.2 Hz, 1H), 4.03 (dd, *J* 9.3 2.8 Hz, 1H), 3.82 (dd, *J* 9.3 3.0 Hz, 1H), 2.27 (s, 3H); ^{13}C NMR (100 MHz; $CDCl_3$): δ 173.5, 167.9, 159.9, 156.2, 143.8, 143.7, 141.3, 127.8, 127.1, 125.1, 120.0, 104.1, 70.5, 67.3, 64.0, 54.2, 47.1, 11.3; LRMS *m/z* (ES+) 440 ([M+ NH_4]$^+$, 100%); HRMS *m/z* (ES+) [Found: (M+Na)$^+$ 445.1381. $C_{23}H_{22}N_2NaO_7^+$ requires 445.1370]; HPLC (C-18, 220 nm) retention time = 12.17 min, 100.0%; (TS, 254 nm) retention time = 9.9 min, ee >99%.

tert-Butyl _N_^{2}-(_tert_-butoxycarbonyl)-_N_^{5}-(3,5-dimethylisoxazol-4-yl)-L-glutaminate (58)

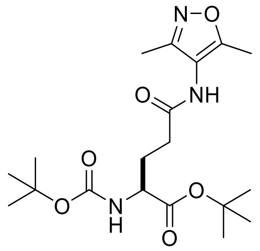

Boc-Glu-*O*^{t}Bu **56** (2 g, 6.59 mmol, 1.0 eq) and 4-amino-3,5-dimethylisoxazole **57** (739 mg, 16.59 mmol, 1.0 eq) were dissolved in dry toluene (20 mL) under N_2. EEDQ (2.25 g, 2.13 mmol, 1.4 eq) was added and the reaction was stirred at rt for 18 h. The reaction mixture was diluted with EtOAc (200 mL) then washed with citric acid (10% *w/v* aq. 3 × 100 mL), Na_2CO_3 (10% *w/v* aq. 3 × 100 mL), H_2O (100 mL) and brine (100 mL), dried ($MgSO_4$), filtered and concentrated *in vacuo*. Purification *via* silica gel chromatography (gradient elution 10 to 60% EtOAc in petroleum ether) gave **58** (2.09 g, 80%) as a yellow foam. R_f 0.74 (EtOAc); $[\alpha]_D$ −0.68 (*c* 1.0 in $CDCl_3$); ν_{max}/cm^{-1} 3278 (br), 2978 (w), 2935 (w), 1711 (m), 1694 (m), 1392 (m), 1243 (m), 1151 (s); ^{1}H NMR (500 MHz; $CDCl_3$): δ 8.27 (s, 1H), 5.38 (d, *J* 7.8 Hz, 1H), 4.29-4.19 (m, 1H), 2.51-2.40 (m, 2H), 2.35 (s, 3H), 2.28-2.19 (m, 1H), 2.21 (s, 3H), 1.91-1.80 (m, 1H), 1.47 (s, 9H), 1.46 (s, 9H); ^{13}C NMR (126 MHz; $CDCl_3$): δ 171.4, 171.1, 165.4, 157.5, 156.7, 113.8, 82.9, 80.7, 53.1, 32.7, 31.0, 28.1, 27.9, 11.3, 9.9; LRMS *m/z* (ES+) 817 ([2M+Na]$^+$, 100%); HRMS *m/z* (ES+) [Found: (M+Na)$^+$ 420.2094. $C_{19}H_{31}N_3O_6Na^+$ requires 420.2105]; Anal. calcd for $C_{19}H_{31}N_3O_6$: C 57.4%, H 7.9%, N 10.6%; Found: C 57.3%, H 7.8%, N 10.7%.

_N_^{5}-(3,5-Dimethylisoxazol-4-yl)-L-glutamine (10)

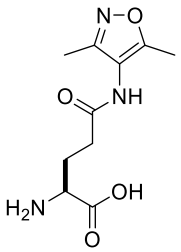

58 (500 mg, 1.20 mmol, 1.0 eq) was dissolved in anhydrous CH_2Cl_2 (30 mL) under N_2 and cooled to -10 °C. BBr_3 (1.45 g, 0.56 mL 5.80 mmol, 5.0 eq) was added dropwise over 5 min with stirring. After 1 h the reaction was allowed to warm to rt and stirred for a further 2 h, then quenched with cold H_2O (10 mL). The product was extracted with H_2O (5 × 20 mL), and the combined aq. layers lyophilized. The crude product was suspended in HCl (2 M in Et_2O) and concentrated under a stream of N_2 to form the HCl salt, which was purified by ion exchange chromatography and lyophilized to afford **10** (182 mg, 63%) as a colorless hygroscopic powder. $[\alpha]_D$ +0.42 (*c* 1.0 in D_2O) ν_{max}/cm^{-1} 2929 (br), 1645 (s), 1522 (s), 1401 (m), 1241 (m);

^{1}H NMR (500 MHz; D_2O): δ 3.72 (dd, *J* 6.2 6.2 Hz, 1H), 2.65-2.50 (m, 2H), 2.20 (s, 3H), 2.15-2.09 (m, 2H), 2.05 (s, 3H); ^{13}C NMR (126 MHz; D_2O): δ 175.1, 173.8, 165.5, 159.1, 112.9, 54.0, 31.0, 26.1, 10.0, 8.6; LRMS *m/z* (ES+) 264 ($[M+Na]^+$, 100%); HRMS *m/z* (ES+) [Found: $(M+Na)^+$ 264.0949. $C_{10}H_{15}N_3O_4Na^+$ requires 264.0955]; Anal. calcd for $C_{10}H_{15}N_3O_4$: C 49.8%, H 6.3%, N 17.4%; Found: C 49.6%, H 6.2%, N 17.3%; HPLC (AS-H; 20:80 EtOH:H_2O, 254 nm) retention time = 9.45 min, ee 89.6%.

Benzyl *N*^{2}-(*tert*-butoxycarbonyl)-*N*^{4}-((3-methylisoxazol-5-yl)methyl)-L-asparaginate (64)

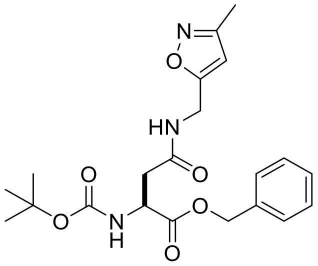

Boc-Asp-*O*-Bn **63** (200 mg, 0.62 mmol, 1.0 eq.) and 1-(3-methylisoxazol-5-yl)methanamine **59**-NH_2 (69 mg, 0.62 mmol, 1.0 eq.) were reacted according to general procedure 1 for 24 h. Purification *via* silica gel chromatography (gradient elution 30 to 70% EtOAc in petroleum ether) and crystallization (3:1 $CHCl_3$:petroleum ether) yielded **64** (130 mg, 50%) as a colorless solid. R_f 0.75 (1:9 MeOH:EtOAc); mp 125-126°C (from 3:1 $CHCl_3$:petroleum ether); $[\alpha]_D$ +4.7 (*c* 1.0 in $CHCl_3$); ν_{max}/cm^{-1} 3310 (w), 2978 (s), 1716 (s), 1499 (s), 1247 (s); ^{1}H NMR (500 MHz; $CDCl_3$): δ 7.37-7.32 (m, 5H), 6.07 (s, 1H), 6.00 (s, 1H), 6.00 (d, *J* 7.9 Hz, 1H), 5.21 (d, *J* 12.3 Hz, 1H), 5.16 (d, *J* 12.3 Hz, 1H), 4.60-4.55 (m, 1H), 4.48 (dd, *J* 15.9 5.7 Hz, 1H), 4.42 (dd, *J* 15.9 5.8 Hz, 1H), 2.92 (dd, *J* 15.4 4.4 Hz, 1H), 2.77 (dd, *J* 15.9 4.4 Hz, 1H), 2.27 (s, 3H), 1.43 (s, 9H); ^{13}C NMR (126 MHz; $CDCl_3$): δ 171.1, 169.8, 168.0, 160.0, 155.6, 135.3, 128.5, 128.4, 128.2, 102.9, 80.2, 67.4, 50.4, 38.0, 35.2, 28.2, 11.4; LRMS *m/z* (ES+) 440 ($[M+Na]^+$, 100%); HRMS *m/z* (ES+) [Found: $(M+Na)^+$ 440.1782. $C_{21}H_{27}N_3NaO_6^+$ requires 440.1792]; Anal. calcd for $C_{14}H_{10}FNO_4$: C 60.4%, H 6.5%, N 10.1%; Found: C 60.5%, H 6.5%, N 9.9%.

Benzyl *N*^{2}-(*tert*-butoxycarbonyl)-*N*^{4}-(3,5-dimethylisoxazol-4-yl)-L-asparaginate (65)

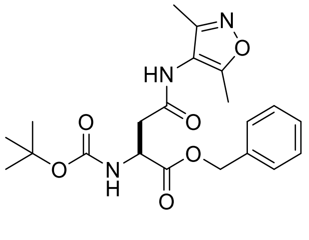

Boc-Asp-*O*-Bn **63** (500 mg, 1.55 mmol, 1.0 eq) and 4-amino-3,5-dimethylisoxazole **60**-NH_2 (173 mg, 1.55 mmol, 1.0 eq) were reacted according to general procedure 1 for 18 h. Purification *via* crystallization from the minimum amount of boiling toluene (×4) yielded **65** (465 mg, 72%) as a colorless crystalline solid. R_f 0.66 (EtOAc); mp 125-129 °C (from toluene); $[\alpha]_D$ +0.89 (*c* 1.0 in $CDCl_3$); ν_{max}/cm^{-1} 3297 (m), 2989 (w), 2938 (w), 1740 (m), 1692 (m), 1649 (m), 1527 (s), 1293 (m), 1164 (s); ^{1}H NMR (500 MHz; $CDCl_3$): δ 7.36-7.32 (m, 5H), 7.00 (s, 1H), 5.74 (d, *J* 6.4 Hz, 1H), 5.23-5.16 (m, 2H), 4.64-4.58 (m, 1H), 3.09-2.86 (m, 2H), 2.25 (s, 3H), 2.11 (s, 3H), 1.42 (s, 9H); ^{13}C NMR (126 MHz; $CDCl_3$): δ 171.0, 169.3, 163.9, 157.5, 155.7, 135.2, 128.6, 128.5, 128.2, 112.9, 80.4, 67.6, 50.7, 38.0, 28.2, 11.1, 9.6; LRMS *m/z* (ES+) 440 ($[M+Na]^+$, 100%); HRMS *m/z* (ES+) [Found: $(M+Na)^+$ 440.1799. $C_{21}H_{27}N_3O_6Na^+$

requires 440.1792]; Anal. calcd for $C_{21}H_{27}N_3O_6$: C 60.4%, H 6.5%, N 10.1%; Found: C 60.6%, H 6.4%, N 9.9%.

Benzyl *N*^{2}-(*tert*-butoxycarbonyl)-*N*^{4}-(5-methylisoxazol-3-yl)-L-asparaginate (66)

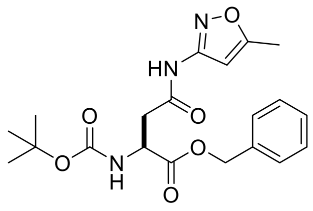

Boc-Asp-*O*-Bn **63** (711 mg, 2.20 mmol, 1.0 eq.) and 3-amino-5-methylisoxazole **61**-NH_2 (216 mg, 2.20 mmol, 1.0 eq.) were reacted according to general procedure 1 for 24 h. Purification *via* silica gel chromatography (gradient elution 7 to 60% EtOAc in petroleum ether) and crystallization (3:1 $CHCl_3$:petroleum ether) yielded **66** (372 mg, 42%) as a yellow solid. R_f 0.76 (1:9 MeOH:EtOAc); mp 107-108 °C (from 3:1 $CHCl_3$:petroleum ether); $[\alpha]_D$ +15.6 (*c* 1.0 in $CHCl_3$); ν_{max}/cm^{-1} 3284 (w), 1700 (s), 1161 (s); ^{1}H NMR (500 MHz; $CDCl_3$): δ 10.17 (s, 1H), 7.33-7.27 (m, 5H), 6.67 (s, 1H), 5.73 (d, *J* 8.9 Hz, 1H), 5.21 (d, *J* 12.4 Hz, 1H), 5.17 (d, *J* 12.4 Hz, 1H), 4.73-4.67 (m, 1H), 3.22 (dd, *J* 16.6 3.9 Hz, 1H), 2.98 (dd, *J* 16.5 4.4 Hz, 1H), 2.39 (s, 3H), 1.42 (s, 9H); ^{13}C NMR (126 MHz; $CDCl_3$): δ 171.2, 170.0, 168.7, 157.9, 155.6, 135.3, 128.5, 128.3, 128.1, 96.6, 80.1, 67.4, 50.1, 38.5, 28.2, 12.6; LRMS *m/z* (ES+) 829 ($[2M+Na]^+$, 100%); HRMS *m/z* (ES+) [Found: $(M+Na)^+$ 426.1630. $C_{20}H_{25}N_3NaO_6^+$ requires 426.1636]; Anal. calcd for $C_{20}H_{25}N_3O_6$: C 59.5%, H 6.3%, N 10.4%; Found: C 59.4%, H 6.1%, N 10.3%.

Benzyl *N*^{2}-(*tert*-butoxycarbonyl)-*N*^{4}-(3-methylisoxazol-5-yl)-L-asparaginate (67)

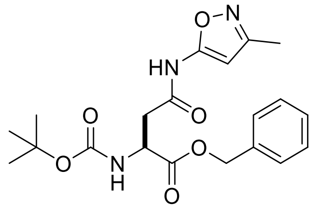

Boc-Asp-*O*-Bn **63** (100 mg, 0.31 mmol, 1.0 eq.) and 3-methyl-5-aminoisoxazole **62**-NH_2 (30 mg, 0.31 mmol, 1.0 eq) were reacted according to general procedure 1 for 24 h. Purification *via* silica gel chromatography (gradient elution 7 to 60% EtOAc in petroleum ether) and crystallization (3:1 $CHCl_3$:petroleum ether) yielded **67** (16 mg, 13%) as a yellow solid. R_f 0.76 (1:9 MeOH:EtOAc); mp 149-150 °C (from 3:1 $CHCl_3$:petroleum ether); $[\alpha]_D$ +8.7 (*c* 1.0 in $CHCl_3$); ν_{max}/cm^{-1} 3283 (w), 1716 (s), 1547 (s), 1164 (s); ^{1}H NMR (500 MHz; $CDCl_3$): δ 9.27 (s, 1H), 7.33-7.31 (m, 5H), 6.18 (s, 1H), 5.69 (d, *J* 8.9 Hz, 1H), 5.22 (d, *J* 12.4 Hz, 1H), 5.18 (d, *J* 12.4 Hz, 1H), 4.66-4.60 (m, 1H), 3.16-3.06 (m, 1H), 3.00 (dd, *J* 16.5 4.4 Hz, 1H), 2.26 (s, 3H), 1.42 (s, 9H); ^{13}C NMR (126 MHz; $CDCl_3$): δ 170.8, 170.1, 166.4, 161.5, 155.7, 135.0, 128.5, 128.4, 128.3, 89.9, 80.6, 67.7, 50.2, 38.6, 28.2, 11.8; LRMS *m/z* (ES+) 426 ($[M+Na]^+$, 100%); HRMS *m/z* (ES+) [Found: $(M+Na)^+$ 426.1637. $C_{20}H_{25}N_3NaO_6^+$ requires 426.1636]; Anal. calcd for $C_{20}H_{25}N_3O_6$: C 59.5%, H 6.3%, N 10.4%; Found: C 59.4%, H 6.2%, N 10.3%.

***N*⁴-((3-Methylisoxazol-5-yl)methyl)-L-asparagine (11)**

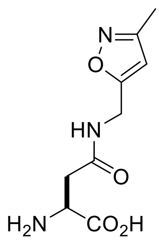

Compound **64** (69 mg, 0.17 mmol, 1.0 eq.) was reacted according to general procedure 2 at –10 °C for 1 h and then for a further 5 h at rt. Purification by ion exchange chromatography, followed by lyophilization yielded **11** (28 mg, 0.12 mmol, 73%) as a hygroscopic colorless solid. R_f 0.77 (RP, 1:1 MeOH:H_2O); $[\alpha]_D$ –4.4 (*c* 0.5 in H_2O); ν_{max}/cm^{-1} 3093 (br), 2930 (s), 1635 (s), 1613 (s), 1440 (s); ^1H NMR (500 MHz; D_2O): δ 6.15 (s, 1H), 4.40 (s, 2H), 3.94 (dd, *J* 7.3 4.6 Hz, 1H), 2.88 (dd, *J* 16.7 4.6 Hz, 1H), 2.79 (dd, *J* 16.7 7.3 Hz, 1H), 2.18 (s, 3H); ^{13}C NMR (126 MHz; D_2O): δ 173.0, 171.9, 168.9, 161.7, 103.2, 51.2, 35.0, 34.9, 10.4; LRMS *m/z* (ES+) 250 ($[M+Na]^+$, 100%); HRMS *m/z* (ES+) [Found: $(M+Na)^+$ 250.0791. $C_9H_{13}N_3NaO_4^+$ requires 250.0798].

***N*⁴-(3,5-Dimethylisoxazol-4-yl)-L-asparagine (12)**

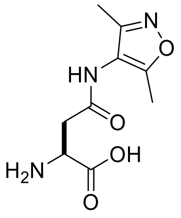

Compound **65** (50 mg, 0.12 mmol, 1.0 eq) was reacted according to general procedure 2 at –10 °C for 1 h and then for a further 2 h at RT. The crude product was suspended in 2 M HCl in Et_2O and concentrated under a stream of N_2 to form the HCl salt, which was purified by ion exchange chromatography and lyophilized to afford **12** (28 mg, 100%) as a colorless hygroscopic powder. $[\alpha]_D$ –0.03 (*c* 0.5 in D_2O); ν_{max}/cm^{-1} 2994 (br), 1630 (s), 1465 (s), 1388 (s), 1234 (m) ^1H NMR (500 MHz; $CDCl_3$): δ 3.99 (dd, *J* 5.7 5.7 Hz, 1H), 2.30 (m, 2H), 2.21 (s, 3H), 2.06 (s, 3H); ^{13}C NMR (126 MHz; $CDCl_3$): δ 172.9, 172.1, 165.7, 159.1, 112.7, 51.2, 34.8, 10.0, 8.6; LRMS *m/z* (ES+) 250 ($[M+Na]^+$, 92%), 477 ($[2M+Na]^+$, 100%); HRMS *m/z* (ES+) [Found: $(M+Na)^+$ 250.0801. $C_9H_{13}N_3O_4Na^+$ requires 250.0798]; Anal. calcd for $C_9H_{13}N_3O_4$: C 47.6%, H 5.8%, N 18.5%; Found: C 47.4%, H 5.6%, N 18.4%; HPLC (AS-H; 20:80 EtOH:H_2O, 254 nm) retention time = 10.01 min, ee 78.1%.

***N*⁴-(5-Methylisoxazol-3-yl)-L-asparagine (13)**

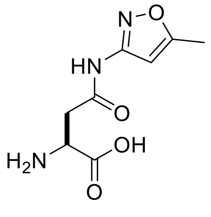

Compound **66** (50 mg, 0.12 mmol, 1.0 eq.) was reacted according to general procedure 2 at –10 °C for 1 h and then for a further 5 h at rt. Purification using an ion exchange column, followed by lyophilization gave **13** (24 mg, 0.11 mmol, 94 %) as a hygroscopic colorless solid. R_f 0.74 (RP, 1:1 MeOH:H_2O); $[\alpha]_D$ +0.6 (*c* 0.5 in H_2O); ν_{max}/cm^{-1} 3030 (br), 1679 (s), 1622 (s), 1556 (s), 1437 (s); ^1H NMR (500 MHz; D_2O): δ 6.34 (s, 1H), 4.02 (dd, *J* 7.5 4.2 Hz, 1H), 3.08

(dd, J 17.2 4.2 Hz, 1H), 2.98 (dd, J 17.2 7.5 Hz, 1H), 2.31 (s, 3H); ^{13}C NMR (126 MHz; $CDCl_3$): δ 173.0, 170.0, 171.6, 157.6, 96.1, 50.8, 35.7, 11.7; LRMS *m/z* (ES+) 236 ($[M+Na]^+$, 100%); HRMS *m/z* (ES+) [Found: $(M+Na)^+$ 236.0641. $C_8H_{11}N_3NaO_4^+$ requires 236.0642].

***N*^{4}-(3-Methylisoxazol-5-yl)-L-asparagine (14)**

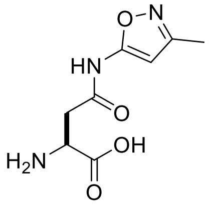

Compound **67** (85 mg, 0.21 mmol, 1.0 eq.) was reacted according to general procedure 2 at –10 °C for 1 h and then for a further 5 h at RT. Purification by ion exchange chromatography, followed by lyophilization yielded **14** (31 mg, 69%) as a hygroscopic colorless solid. R_f 0.81 (RP, 1:1 MeOH:H_2O); $[\alpha]_D$ +0.6 (*c* 0.5 in H_2O); ν_{max}/cm^{-1} 2971 (br), 1700 (s), 1503 (s), 1409 (s), 1203 (s), 785 (s); ^{1}H NMR (500 MHz; D_2O): δ 6.15 (s, 1H), 4.04 (dd, J 7.5 4.4 Hz, 1H), 3.09 (dd, J 17.4 4.4 Hz, 1H), 3.00 (dd, J 17.4 7.5 Hz, 1H), 2.18 (s, 3H); ^{13}C NMR (126 MHz; $CDCl_3$): δ 172.9, 168.5, 163.0, 160.2, 90.4, 50.7, 35.6, 10.8; LRMS *m/z* (ES+) 236 ($[M+Na]^+$, 100%); HRMS *m/z* (ES+) [Found: $(M+Na)^+$ 236.0640. $C_8H_{11}N_3NaO_4^+$ requires 236.0642].

3-Methylisoxazole-5-carboxylic acid (68)

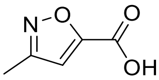

A solution of LiOH (1 M in THF, 20 mL, 20 mmol, 5.0 eq.) was added to a suspension of ethyl 3-methylisoxazole-5-carboxylate **47** (630 mg, 4.06 mmol, 1.0 eq.) in THF (30 mL) and MeOH (30 mL) at rt. After 18 h the volatile components were removed *in vacuo* and the resulting residue was redissolved in H_2O (30 mL), acidified to pH 2 (1 M HCl), extracted with EtOAc (3 × 30 mL), washed with brine (40 mL), dried ($MgSO_4$), filtered and concentrated *in vacuo*. Purification *via* crystallization from H_2O yielded **68** (473 mg, 92%) as a colorless solid. Rf 0.15 (1:9 MeOH:EtOAc); mp 170-171 °C (from H_2O; lit.[25] value: 170-170.5 °C); ^{1}H NMR (400 MHz; methanol-d_4): δ 6.92 (s, 1H), 2.36 (s, 3H); LRMS *m/z* (ES–) 126 ($[M-H]^-$, 100%). These data are in good agreement with the literature values.[25]

2,5-Dioxopyrrolidin-1-yl 3-methylisoxazole-5-carboxylate (69)

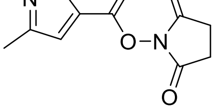

Following the procedure of Leonard and Brunckova,[26] to solution of **68** (318 mg, 2.5 mmol, 1.0 eq), *N*-hydroxysuccinimide (575 mg, 5.0 mmol, 2.0 eq) and anhydrous pyridine (810 µL, 790 mg, 10.0 mmol, 4.0 eq) in anhydrous CH_2Cl_2 (12.5 mL) at 0 °C under a N_2 atmosphere was added trifluoroacetic anhydride (700 µL, 1.05 g, 5.0 mmol, 2.0 eq) dropwise. The reaction was allowed to warm to rt and stirred for 1 h, then diluted with CH_2Cl_2 (40 mL) and aq. HCl (1 M, 50 mL). The organic layer was washed with aq. HCl (2 × 40 mL) and sat. aq. $NaHCO_3$ (2 × 40 mL), dried ($MgSO_4$), filtered and concentrated *in vacuo* to give **69** as a colorless solid (498 mg, 88%) which was used without further purification. R_f 0.43 (1:1 EtOAc:petroleum ether); mp 130-131 °C (from EtOAc); ν_{max}/cm^{-1} 2962 (w), 1738 (s), 1205 (m), 1049 (s); ^{1}H NMR

(500 MHz; $CDCl_3$): δ 7.06 (s, 1H), 2.92 (s, 4H), 2.43 (s, 3H); ^{13}C NMR (126 MHz; $CDCl_3$): δ 168.3, 160.7, 155.2, 152.1, 113.1, 25.6, 11.4; LRMS *m/z* (ES+) 247 ($[M+Na]^+$, 100%); HRMS *m/z* (ES+) [Found: $(M+Na)^+$ 247.0331. $C_9H_8N_2NaO_5^+$ requires 247.0325]; Anal. calcd for $C_9H_8N_2O_5$: C 48.2%, H 3.6%, N 12.5%; Found: C 48.3%, H 3.5%, N 12.4%.

(*S*)-2-((*tert*-Butoxycarbonyl)amino)-3-(3-methylisoxazole-5-carboxamido)propanoic acid (70)

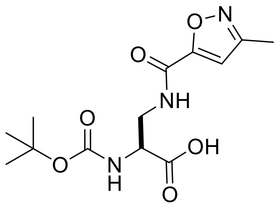

Boc-Dap-OH (426 mg, 2.08 mmol, 1.3 eq.) was added to a solution of **69** (360 mg, 1.60 mmol, 1.0 eq.) in anhydrous DMF (4.5 mL) and left to stir at rt. After 22 h the volatile components were removed *in vacuo* and the resulting residue was dissolved in H_2O (10 mL). The product was extracted with EtOAc (5 × 10 mL), washed with citric acid (10% *w/v* aq. 3 × 10 mL), H_2O (10 mL) and brine (10 mL), dried ($MgSO_4$), filtered and concentrated *in vacuo* to give **70** (350 mg, 70%) as a colorless solid without need for further purification. R_f 0.79 (RP, 1:1 MeOH:H_2O); mp 157-158 °C (from EtOAc); $[\alpha]_D$ +11.9 (*c* 1.0 in $CHCl_3$); ν_{max}/cm^{-1} 3417 (w), 2980 (s), 1671 (s), 1517 (s), 1162 (m), 732 (s); ^1H NMR (500 MHz; $CDCl_3$): δ 7.51 (s, 1H), 6.77 (s, 1H), 5.81 (d, *J* 6.0 Hz, 1H), 4.52-4.46 (m, 1H), 3.96-3.85 (m, 2H), 2.36 (s, 3H), 1.45 (s, 9H); ^{13}C NMR (126 MHz; $CDCl_3$): δ 172.2, 162.3, 161.0, 157.4, 156.4, 108.2, 81.2, 54.0, 41.4, 28.2, 11.4; LRMS *m/z* (ES+) 336 ($[M+Na]^+$, 87%), 649 ($[2M+Na]^+$, 100%); HRMS *m/z* (ES+) [Found: $(M+Na)^+$ 336.1158. $C_{13}H_{19}N_3NaO_6^+$ requires 336.1166]; Anal. calcd for $C_{13}H_{19}N_3O_6$: C 49.8%, H 6.1%, N 13.4%; Found: C 49.8%, H 6.1%, N 13.3%.

(*S*)-2-Amino-3-(3-methylisoxazole-5-carboxamido)propanoic acid (5)

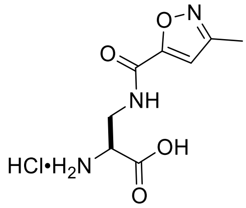

HCl (2 M in Et_2O, 3 mL) was added to **70** (50 mg, 0.16 mmol, 1.0 eq.) and the mixture stirred for 96 h. The reaction mixture was concentrated *in vacuo* and the resulting solid was triturated from Et_2O to yield **5** (28 mg, 71%) as a yellow solid. R_f 0.80 (RP, 1:1 MeOH:H_2O); mp 206-207 °C (from Et_2O); $[\alpha]_D$ –26.2 (*c* 0.5 H_2O); ν_{max}/cm^{-1} 3335 (w); 2980 (br), 1709 (s), 1657 (s), 1228 (s), 763 (s); ^1H NMR (500 MHz; $CDCl_3$): δ 6.81 (s, 1H), 4.17 (dd, *J* 6.3 4.1 Hz, 1H), 3.92 (dd, *J* 15.0 4.1 Hz, 1H), 3.81 (dd, *J* 15.0 6.3 Hz, 1H), 2.24 (s, 3H); ^{13}C NMR (126 MHz; $CDCl_3$): δ 169.9, 162.1, 161.4, 169.2, 108.5, 53.5, 39.0, 10.4; LRMS *m/z* (ES+) 236 ($[M+Na]^+$, 100%); HRMS *m/z* (ES+) [Found: $(M+Na)^+$ 236.0644. $C_8H_{11}N_3NaO_4^+$ requires 236.0642].

(*S*)-2-((((9*H*-Fluoren-9-yl)methoxy)carbonyl)amino)-3-(3-methylisoxazole-5-carboxamido)propanoic acid (71)

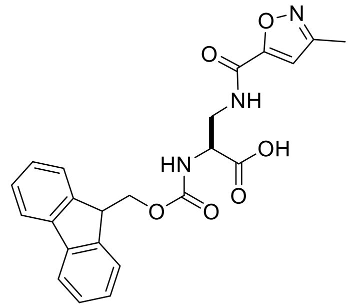

A solution of Fmoc-Dap-OH (816 mg, 2.50 mmol, 1.0 eq) and **69** (561 mg, 2.50 mmol, 1.0 eq) in anhydrous DMF (12.5 mL) was stirred for 17 h, then concentrated *in vacuo*. The residues were dissolved in EtOAc (80 mL), washed with citric acid (10% *w/v* aq., 2 × 80 mL), H_2O (4 × 80 mL), brine (80 mL), dried ($MgSO_4$), filtered and concentrated *in vacuo* to give a pale yellow solid. Purification *via* crystallization from boiling $CHCl_3$ gave **71** as a colourless solid (915 mg, 84%). R_f 0.80 (0.5% AcOH in EtOAc); $[\alpha]_D$ –14.7 (*c* 1.0 in MeOH); ν_{max}/cm^{-1} 3317 (br), 1697 (s), 1531 (s), 1449 (m), 1249 (s), 1052 (m); ^{1}H NMR (500 MHz; acetone-d_6): δ 11.50 (br s, 1H), 8.27-8.16 (m, 1H), 7.87 (d, *J* 7.5 Hz, 2H), 7.71 (d, *J* 7.5 Hz, 2H), 7.42 (dd, *J* 7.5 7.5 Hz, 2H), 7.35-7.29 (m, 1H), 6.99 (d, *J* 8.0 Hz, 1H), 6.83 (s, 1H), 4.59 (ddd, *J* 8.0 7.8 5.0 Hz, 1H), 4.37-4.31 (m, 2H), 4.28-4.22 (m, 1H), 3.99-3.83 (m, 2H), 2.32 (s, 3H); ^{13}C NMR (126 MHz; acetone-d_6): δ 171.9, 164.2, 161.6, 157.5, 157.1, 145.0, 142.1, 128.5, 128.0, 126.2, 120.8, 108.1, 67.4, 54.8, 48.0, 41.3, 11.3; LRMS *m/z* (ES+) 458 ($[M+Na]^+$, 100%); HRMS *m/z* (ES+) [Found: $(M+Na)^+$ 458.1325. $C_{23}H_{21}N_3NaO_6^+$ requires 458.1323]; HPLC (C-18, 220 nm) retention time = 11.50 min, 98.9%; (TS, 254 nm) retention time = 15.2 min, ee 98.1%.

Tripeptide H-Tyr-Cys-Lys-OH (YCK)

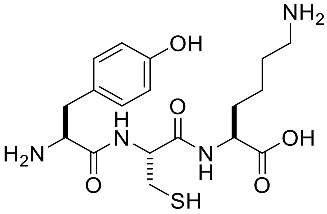

The tripetide was synthesized in a 7-step process from amino acid starting materials in various states of protection, according to standard solution-phase peptide synthesis techniques, and fully deprotected to afford **YCK** (0.57 g, 1.38 mg) as an off-white powder which was stored in the dark, under argon at –20 °C (the compound easily oxidised to form the disulfide dimer as an orange solid if stored in the light). $[\alpha]_D$ −13.8 (*c* 1.0 in $CHCl_3$); ν_{max}/cm^{-1} 3080 (br), 2964 (w), 2489 (w), 1665 (s), 1157 (m), 1460 (m), 1367 (w), 1197 (s), 1140 (s); ^{1}H NMR (500 MHz; D_2O): δ 6.80 (d, *J* 8.5 Hz, 2H), 6.52 (d, *J* 8.5 Hz, 2H), 4.47-4.32 (m, 1H), 4.12-4.01 (m, 1H), 3.88-3.73 (m, 1H), 2.93-2.86 (m, 4H), 2.65-2.59 (m, 1H), 2.59-2.48 (m, 1H), 1.78-1.67 (m, 1H), 1.67-1.53 (m, 3H), 1.34-1.26 (m, 2H); ^{13}C NMR (126 MHz; D_2O): δ 178.3, 170.8, 170.6, 154.7, 130.7, 127.3, 115.7, 55.4, 53.0, 52.8, 39.8, 38.0, 33.9, 31.1, 26.4, 22.0; LRMS *m/z* (ES^+) 413 ($[M+H]^+$, 100%); HRMS *m/z* (ES+) [Found: $(M+H)^+$ 413.18512. $C_{18}H_{29}O_5N_4{}^{32}S^+$ requires 413.18512].

References

[1] D. G. Fatkins, A. D. Monnot, W. Zheng, *Bioorg. Med. Chem. Lett.* **2006**, *16*, 3651–3656.

[2] B. C. Smith, J. M. Denu, *J. Am. Chem. Soc.* **2007**, *129*, 5802–5803.

[3] N. Jamonnak, D. G. Fatkins, L. Wei, W. Zheng, *Org. Biomol. Chem.* **2007**, *5*, 892–896.

[4] N. Jamonnak, B. M. Hirsch, Y. Pang, W. Zheng, *Bioorg. Chem.* **2010**, *38*, 17–25.

[5] R. Huang, M. A. Holbert, M. K. Tarrant, S. Curtet, D. R. Colquhoun, B. C. B. M. Dancy, B. C. B. M. Dancy, Y. Hwang, Y. Tang, K. Meeth, et al., *J. Am. Chem. Soc.* **2010**, *132*, 9986–9987.

[6] B. M. Hirsch, Y. Hao, X. Li, C. Wesdemiotis, Z. Wang, W. Zheng, *Bioorg. Med. Chem. Lett.* **2011**, *21*, 4753–4757.

[7] B. M. Hirsch, Z. Du, X. Li, J. a. Sylvester, C. Wesdemiotis, Z. Wang, W. Zheng, *Med. Chem. Commun.* **2011**, *2*, 291–299.

[8] B. C. R. Dancy, S. A. Ming, R. Papazyan, C. A. Jelinek, A. Majumdar, Y. Sun, B. M. Dancy, W. J. Drury, R. J. Cotter, S. D. Taverna, et al., *J. Am. Chem. Soc.* **2012**, *134*, 5138–5148.

[9] D. S. Hewings, O. Fedorov, P. Filippakopoulos, S. Martin, S. Picaud, A. Tumber, C. Wells, M. M. Olcina, K. Freeman, A. Gill, et al., *J. Med. Chem.* **2013**, *56*, 3217–3227.

[10] P. Filippakopoulos, S. Picaud, M. Mangos, T. Keates, J.-P. Lambert, D. Barsyte-Lovejoy, I. Felletar, R. Volkmer, S. Müller, T. Pawson, et al., *Cell* **2012**, *149*, 214–231.

[11] D. Suckau, A. Resemann, M. Schuerenberg, P. Hufnagel, J. Franzen, A. Holle, *Anal. Bioanal. Chem.* **2003**, *376*, 952–965.

[12] M. Philpott, J. Yang, T. Tumber, O. Fedorov, S. Uttarkar, P. Filippakopoulos, S. Picaud, T. Keates, I. Felletar, A. Ciulli, et al., *Mol. Biosyst.* **2011**, *7*, 2899–2908.

[13] A. B. Pangborn, M. A. Giardello, R. H. Grubbs, R. K. Rosen, F. J. Timmers, *Organometallics* **1996**, *15*, 1518–1520.

[14] G. A. Molander, B. Canturk, L. E. Kennedy, *J. Org. Chem.* **2009**, *74*, 973–980.

[15] A. Anantanarayan, P. J. Dutton, T. M. Fyles, M. J. Pitre, **1986**, *6*, 752–755.

[16] P. Ceccherelli, M. Curini, M. C. Marcotullio, O. Rosati, E. Wenkert, *Electron. Publ.* **1994**, *59*, 2882–2884.

[17] G. Dannhardt, W. Kiefer, G. Lambrecht, S. Laufer, E. Mutschler, J. Schweiger, H. G. Striegep, *Eur. J. Med. Chem.* **1995**, *30*, 839–850.

[18] W. H. Bunnelle, P. R. Singam, B. A. Narayanan, C. W. Bradshaw, J. S. Liou, *Synth. Stuttgart* **1997**, 439–442.

[19] F. Lepage, F. Tombret, G. Cuvier, A. Marivain, J. Gillardin, *Eur. J. Med. Chem.* **1992**, *27*, 581–593.

[20] K. Harada, E. Kaji, S. Zen, *Biol Pharm Bull.* **1980**, *28*, 3296–3303.

[21] G. Giacomelli, L. De Luca, A. Porcheddu, *Tetrahedron* **2003**, *59*, 5437–5440.

[22] J. Zhang, D. P. Curran, *J. Chem. Soc. Perkin Trans. 1* **1991**, 2627–2631.

[23] S. Al-Busafi, M. Al-Belushi, K. Al-Muqbali, *Synth. Commun.* **2010**, *40*, 1088–1092.

[24] R. A. Gadzhily, A. A. Aliev, **2002**, *38*, 415–418.

[25] S. Sumimoto, *Kogyo Kagaku Zasshi* **1963**, *66*, 1838–1841.

[26] N. M. Leonard, J. Brunckova, *J. Org. Chem.* **2011**, *76*, 9169–9174.

^{1}H and ^{13}C spectra

^{1}H, ^{13}C and (where appropriate) ^{19}F NMR spectra are reported for all novel compounds, i.e. those for which these data have not previously been reported. Spectra are presented in the order in which the compounds appear in Synthesis and Characterization.

tert-Butyl (*S*)-3-(3-bromophenyl)-2-((*tert*-butoxycarbonyl)amino)propanoate **30** ^{1}H NMR

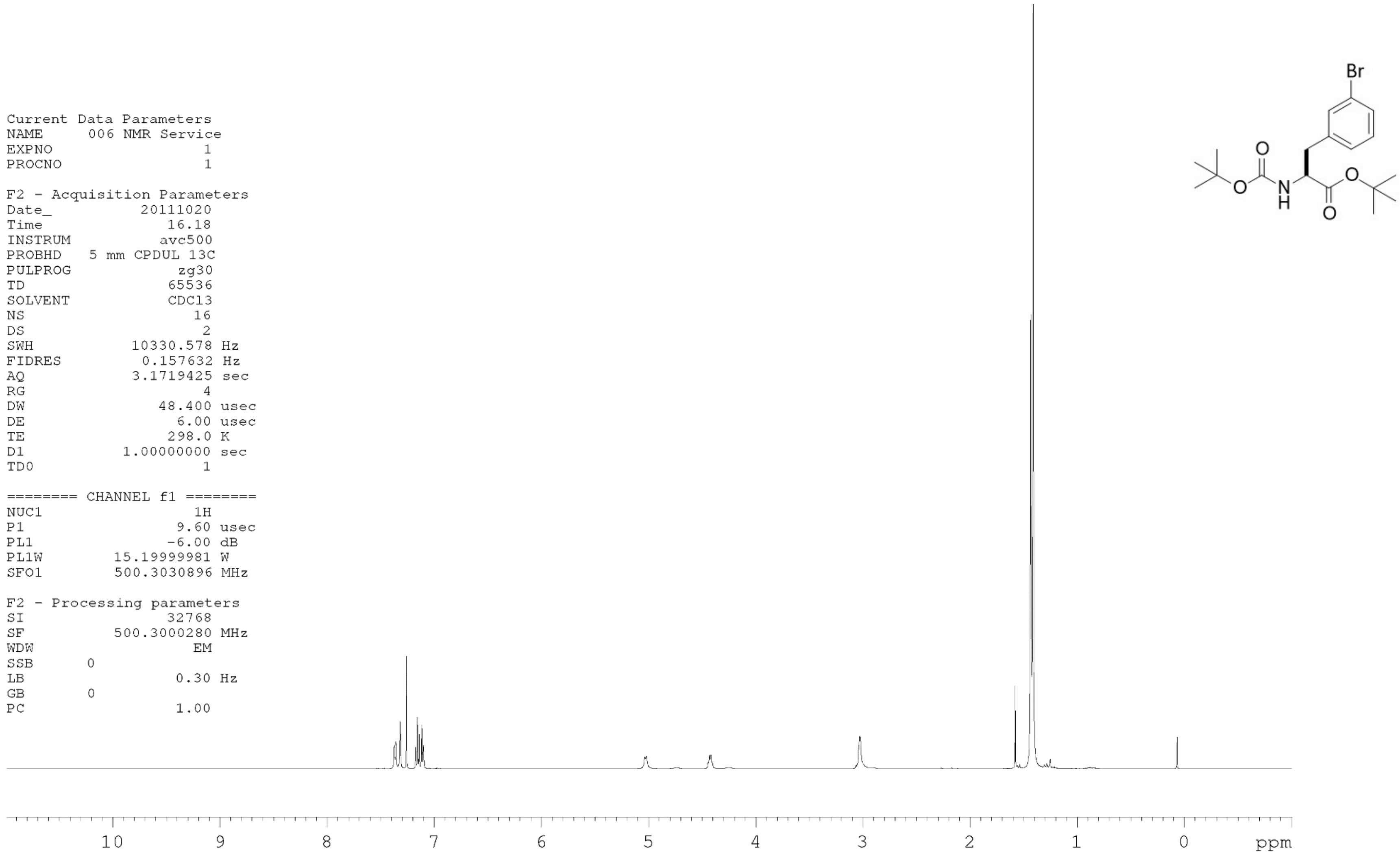

tert-Butyl (*S*)-3-(3-bromophenyl)-2-((*tert*-butoxycarbonyl)amino)propanoate **30** ^{13}C NMR

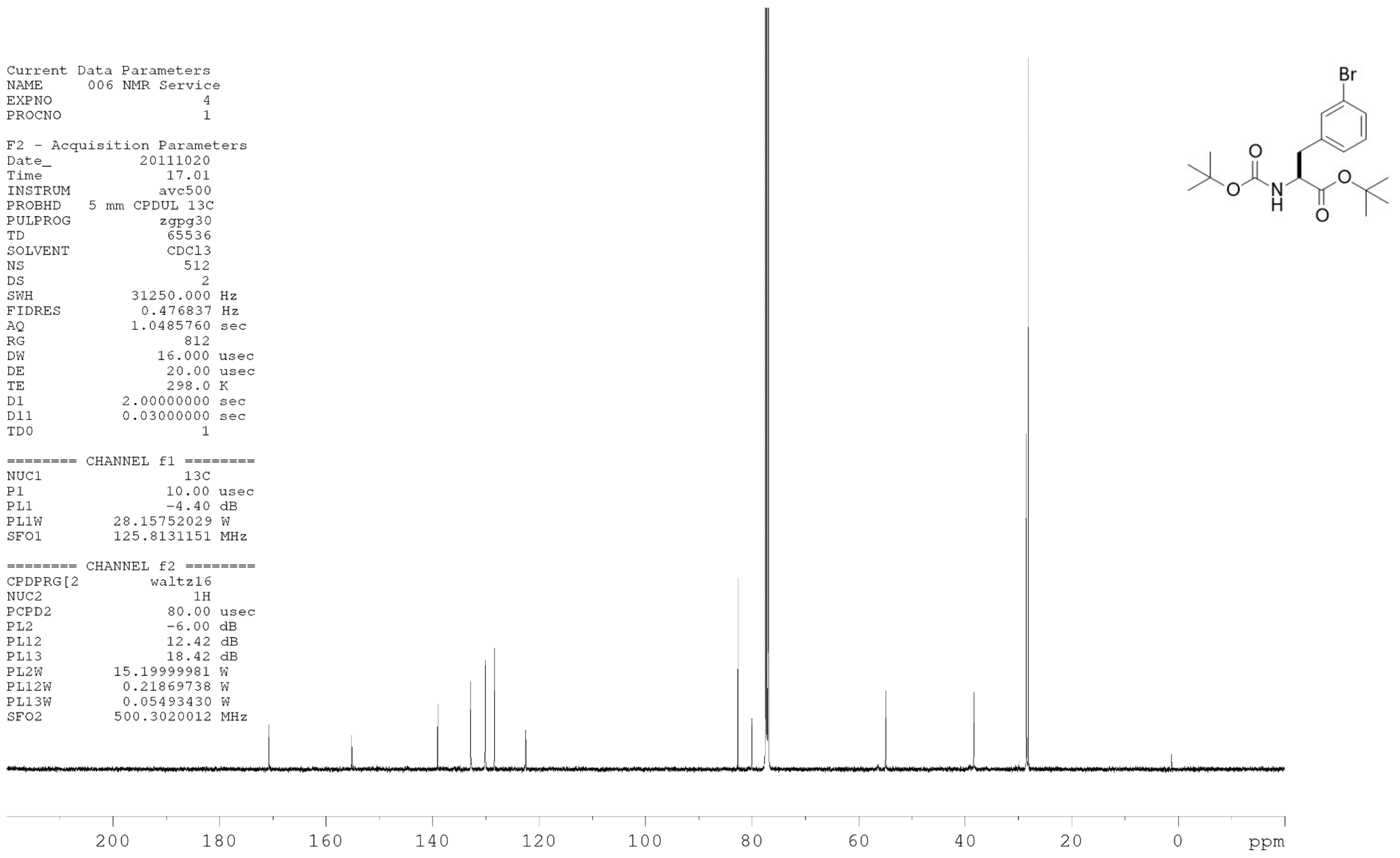

tert-Butyl (*S*)-2-((*tert*-butoxycarbonyl)amino)-3-(3-(3,5-dimethylisoxazol-4-yl)phenyl)propanoate **31** ^{1}H NMR

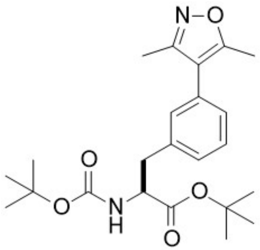

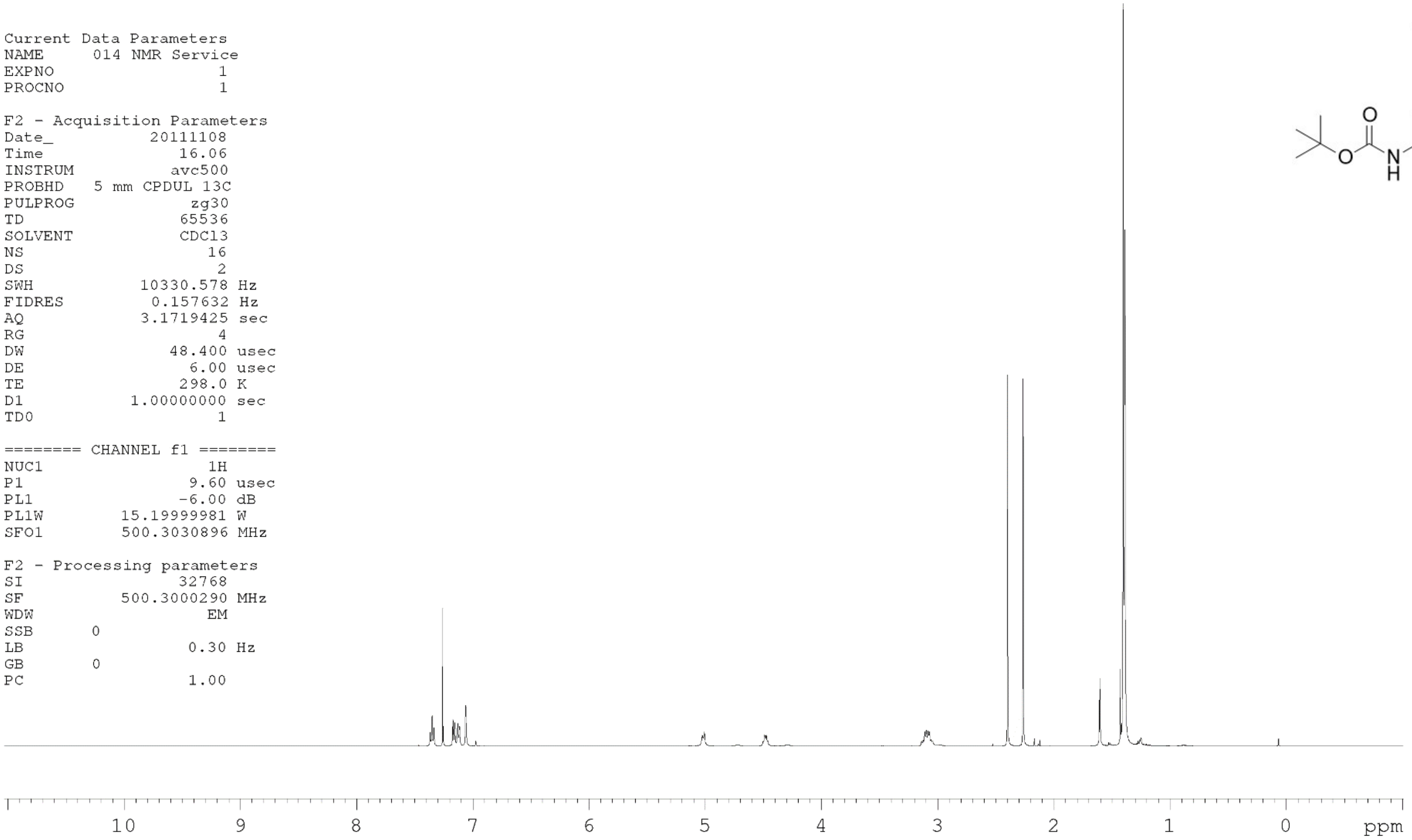

tert-Butyl (*S*)-2-((*tert*-butoxycarbonyl)amino)-3-(3-(3,5-dimethylisoxazol-4-yl)phenyl)propanoate **31** ^{13}C NMR

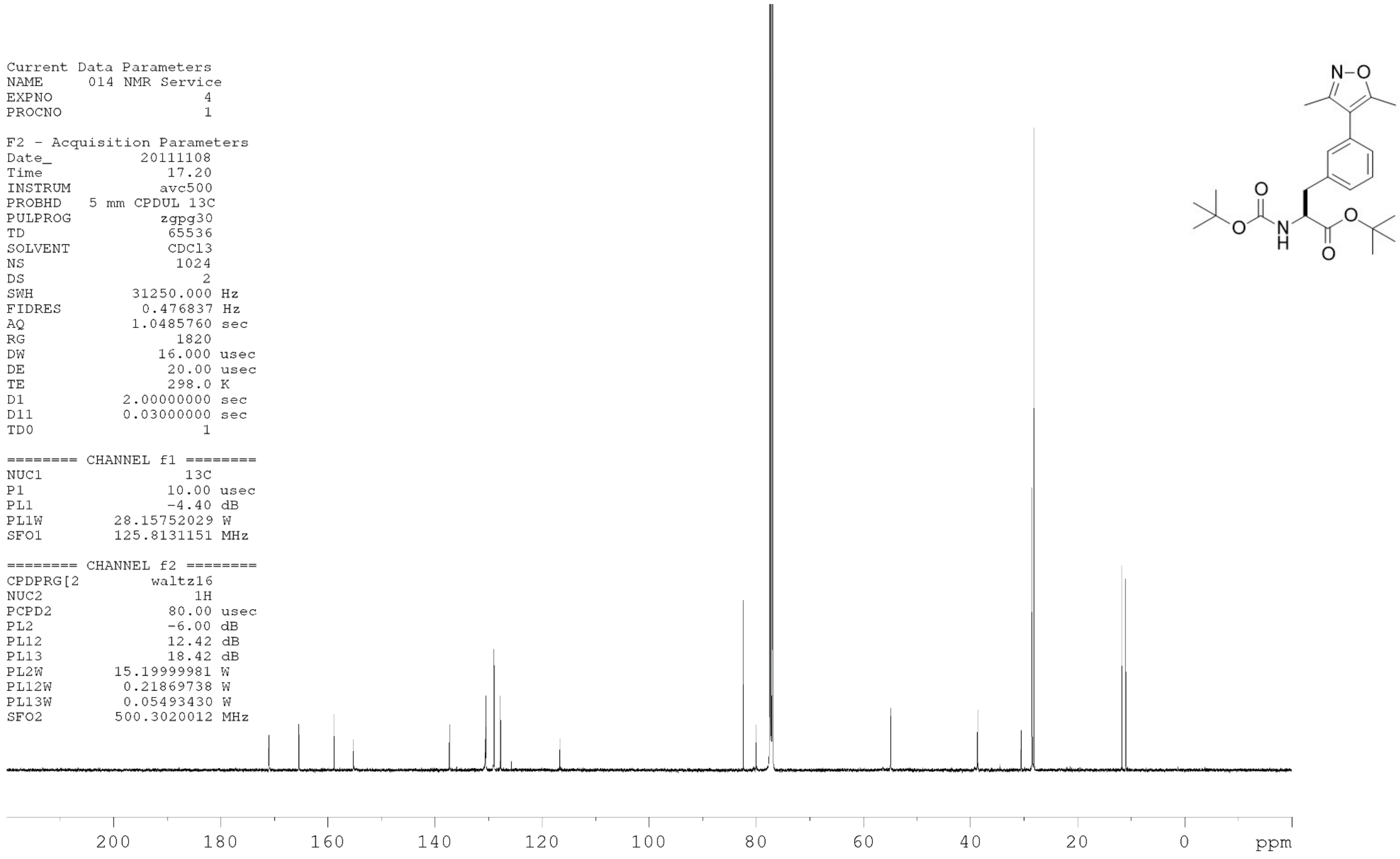

(*S*)-2-Amino-3-(3-(3,5-dimethylisoxazol-4-yl)phenyl)propanoic acid **8** ^{1}H NMR

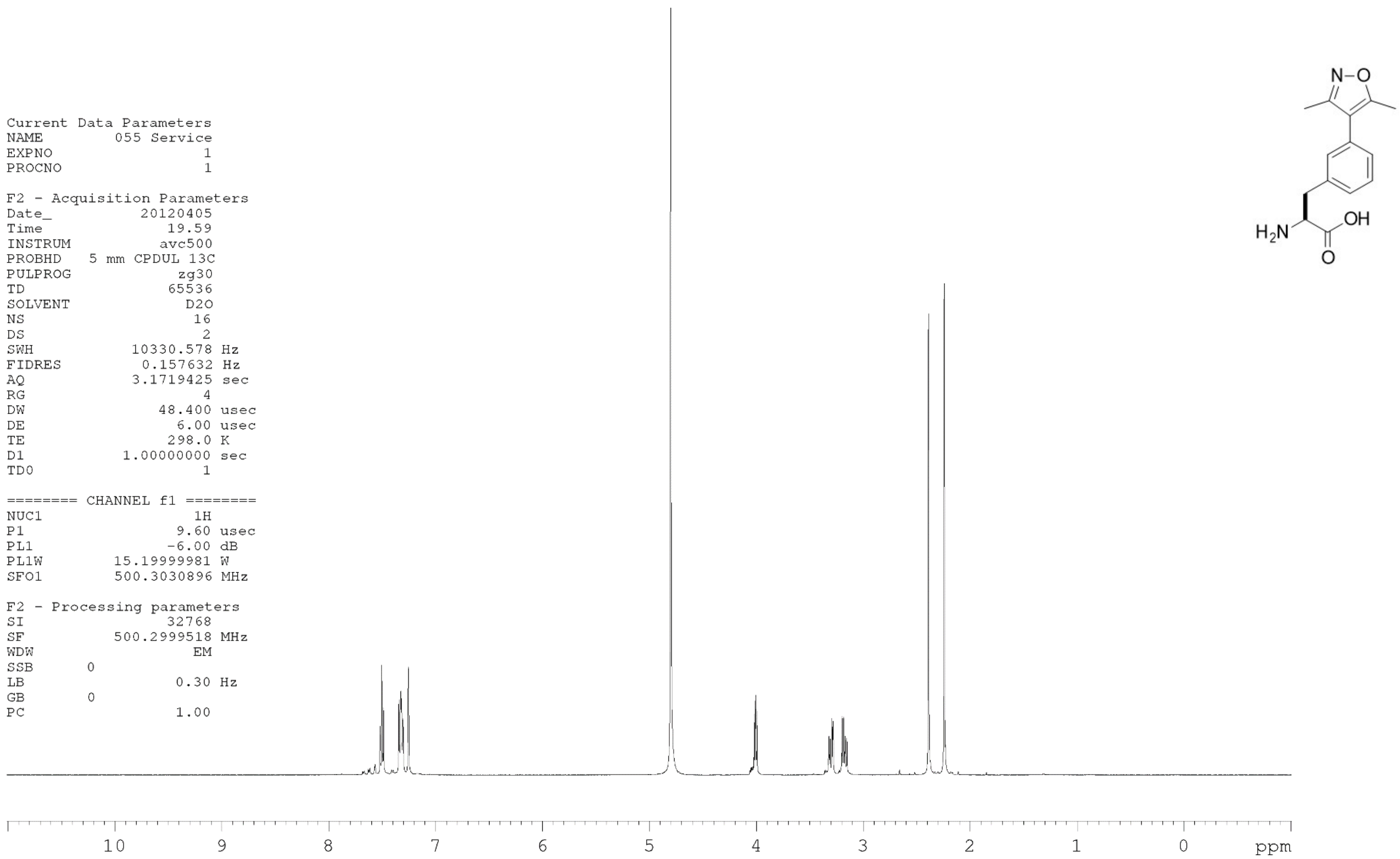

(*S*)-2-Amino-3-(3-(3,5-dimethylisoxazol-4-yl)phenyl)propanoic acid **8** ^{13}C NMR

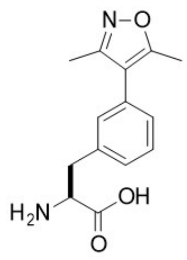


```
Current Data Parameters
NAME        055 Service
EXPNO                 4
PROCNO                1

F2 - Acquisition Parameters
Date_          20120405
Time              21.14
INSTRUM          avc500
PROBHD   5 mm CPDUL 13C
PULPROG          zgpg30
TD                65536
SOLVENT             D2O
NS                 1024
DS                    2
SWH           31250.000 Hz
FIDRES         0.476837 Hz
AQ            1.0485760 sec
RG                 1820
DW               16.000 usec
DE                20.00 usec
TE                298.0 K
D1           2.00000000 sec
D11          0.03000000 sec
TD0                   1

======== CHANNEL f1 ========
NUC1                13C
P1                10.00 usec
PL1               -4.40 dB
PL1W        28.15752029 W
SFO1        125.8131151 MHz

======== CHANNEL f2 ========
CPDPRG[2        waltz16
NUC2                 1H
PCPD2             80.00 usec
PL2               -6.00 dB
PL12              12.42 dB
PL13              18.42 dB
PL2W        15.19999981 W
PL12W        0.21869738 W
PL13W        0.05493430 W
SFO2        500.3020012 MHz
```

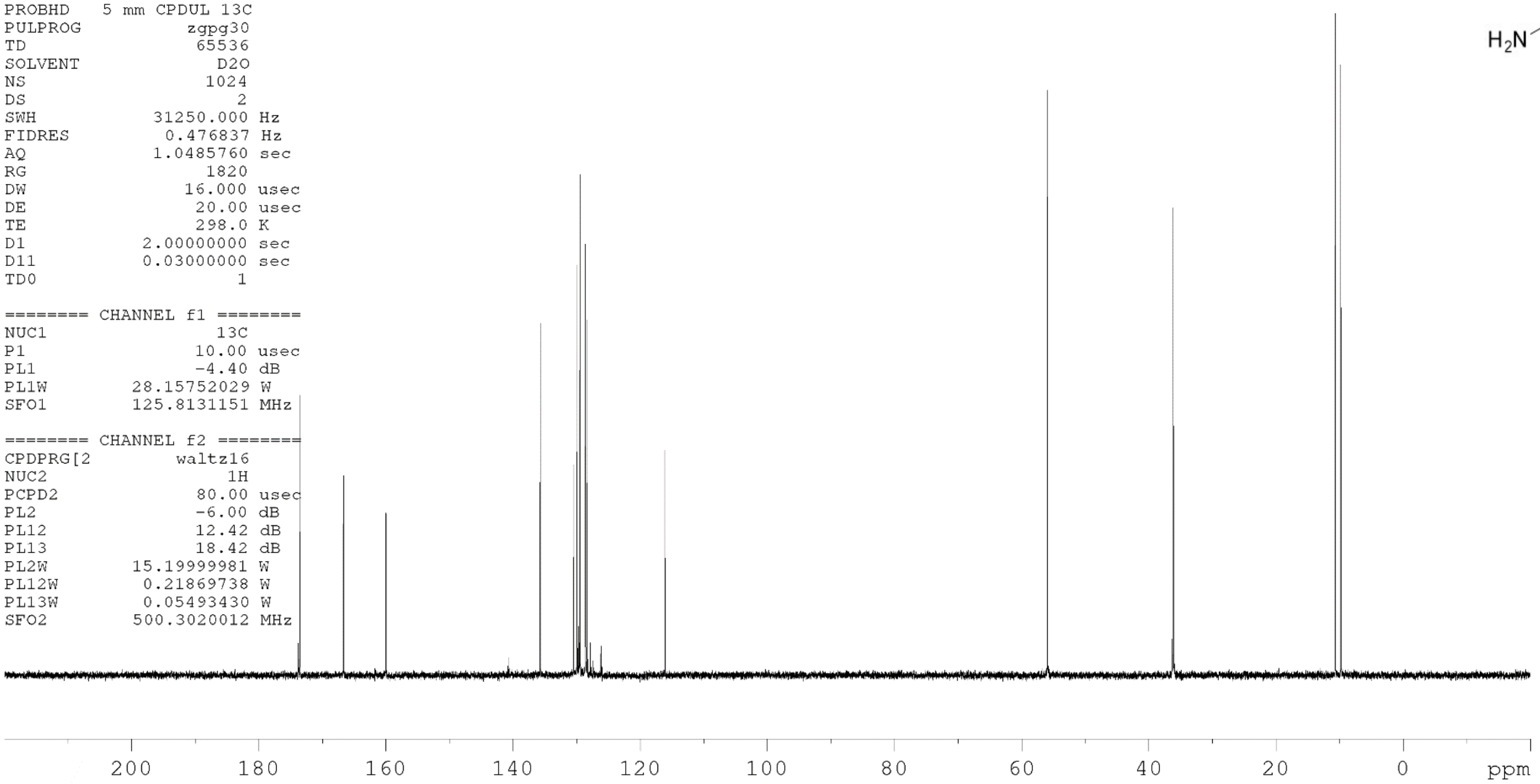

Ethyl (*S*)-3-(4-bromophenyl)-2-((*tert*-butoxycarbonyl)amino)propanoate **33** ^{1}H NMR

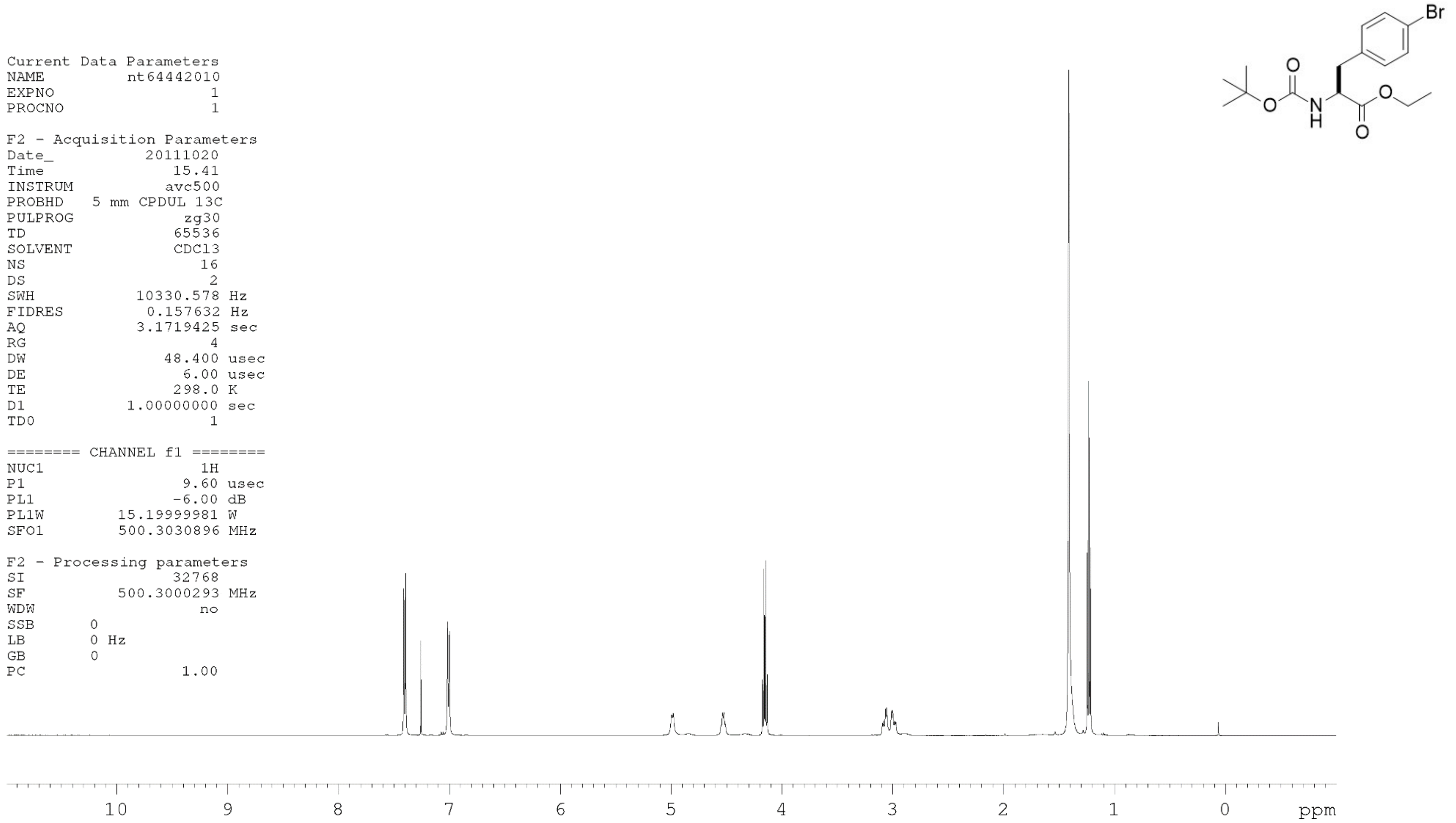

Ethyl (*S*)-3-(4-bromophenyl)-2-((*tert*-butoxycarbonyl)amino)propanoate **33** ^{13}C NMR

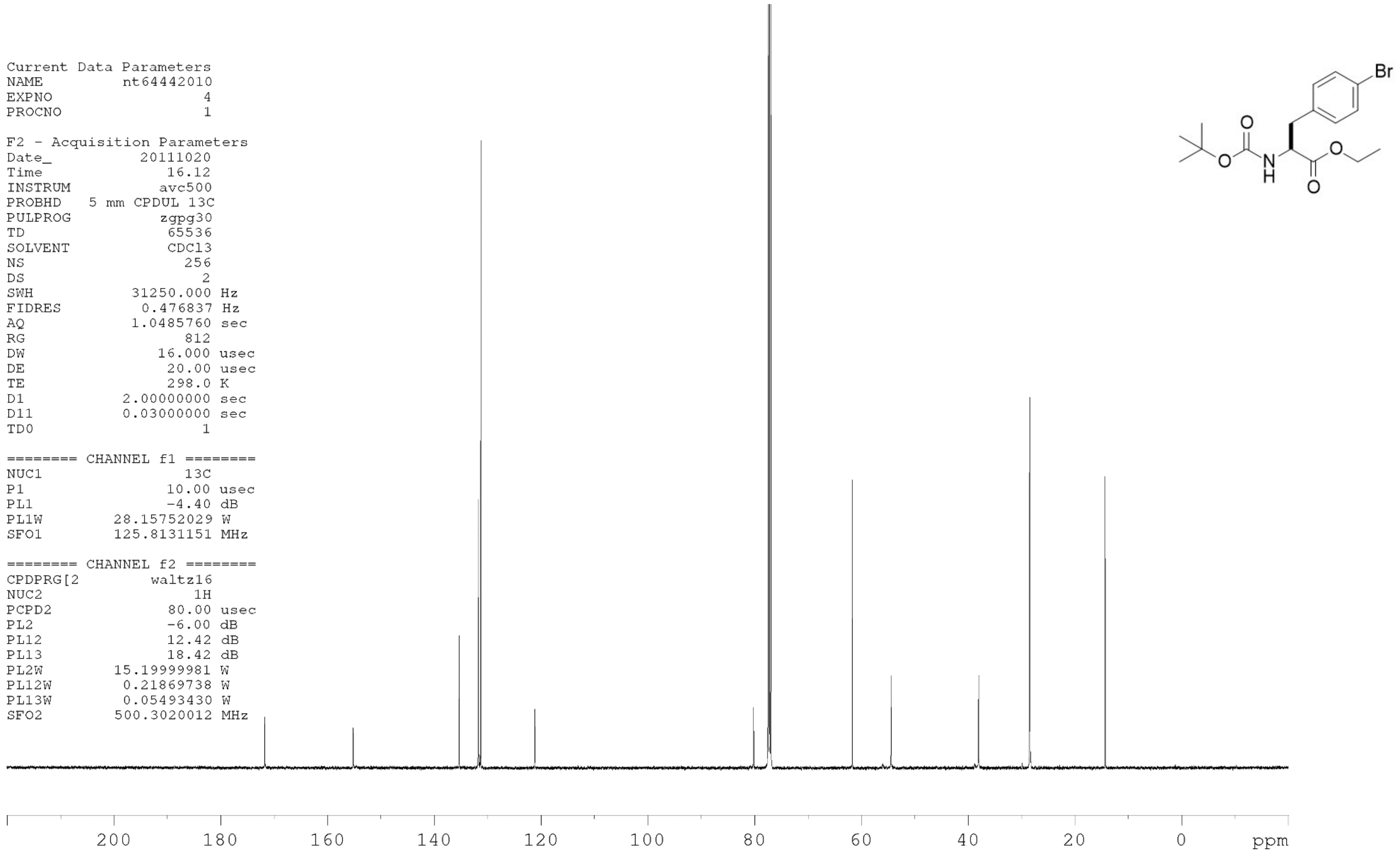

Ethyl (S)-2-((*tert*-butoxycarbonyl)amino)-3-(4-(3,5-dimethylisoxazol-4-yl)phenyl)propanoate **34** ^{1}H NMR

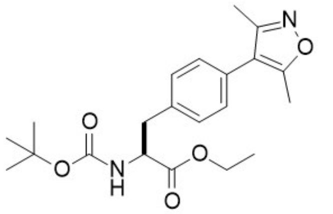

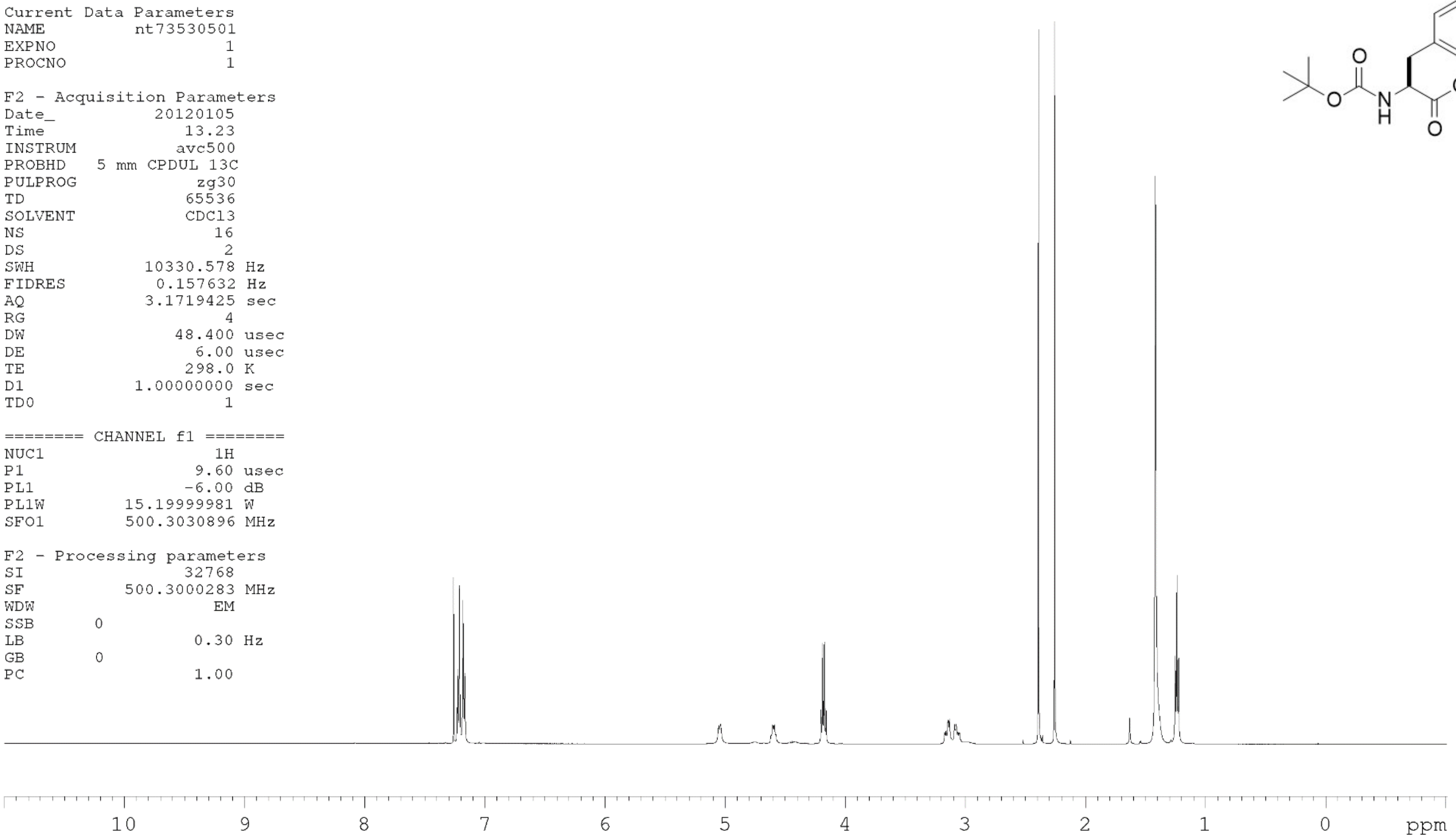

Ethyl (*S*)-2-((*tert*-butoxycarbonyl)amino)-3-(4-(3,5-dimethylisoxazol-4-yl)phenyl)propanoate **34** ^{13}C NMR

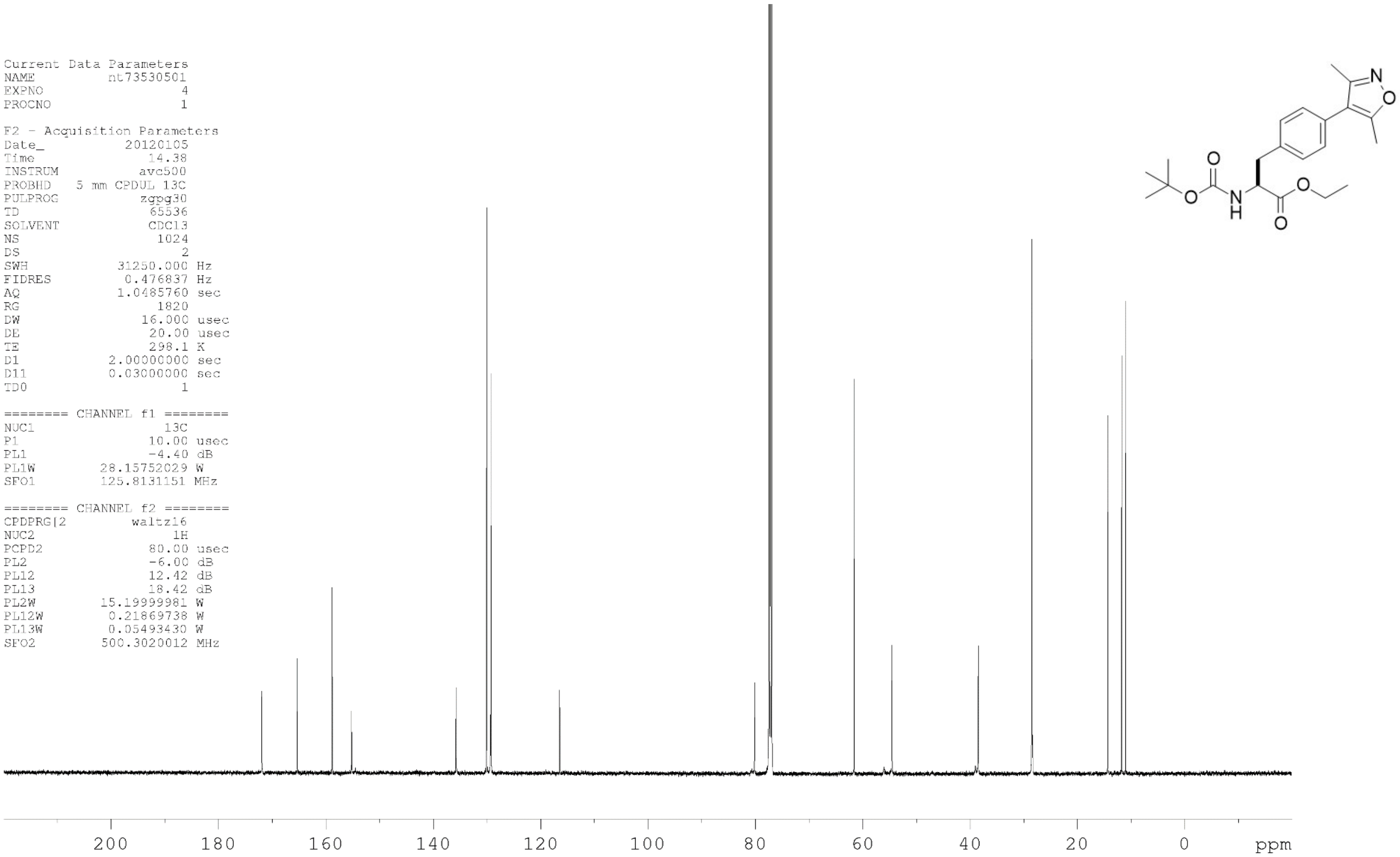

(*S*)-2-((*tert*-Butoxycarbonyl)amino)-3-(4-(3,5-dimethylisoxazol-4-yl)phenyl)propanoic acid **35** ^{1}H NMR

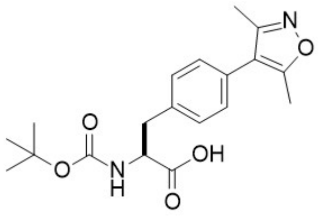


```
Current Data Parameters
NAME     NT-065 after grease wash
EXPNO                  1
PROCNO                 1

F2 - Acquisition Parameters
Date_           20120228
Time               22.26
INSTRUM           dpx400
PROBHD   5 mm Dual 1H/1
PULPROG             zg60
TD                 32768
SOLVENT            CDCl3
NS                    16
DS                     2
SWH             5592.841 Hz
FIDRES          0.170680 Hz
AQ             2.9294591 sec
RG                   256
DW                89.400 usec
DE                 17.00 usec
TE                 300.0 K
D1            1.00000000 sec

======== CHANNEL f1 ========
NUC1                  1H
P1                  7.30 usec
PL1      0 dB
SFO1         400.1320007 MHz

F2 - Processing parameters
SI                 32768
SF           400.1300222 MHz
WDW                   EM
SSB      0
LB                  0.30 Hz
GB       0
PC                  0.60
```

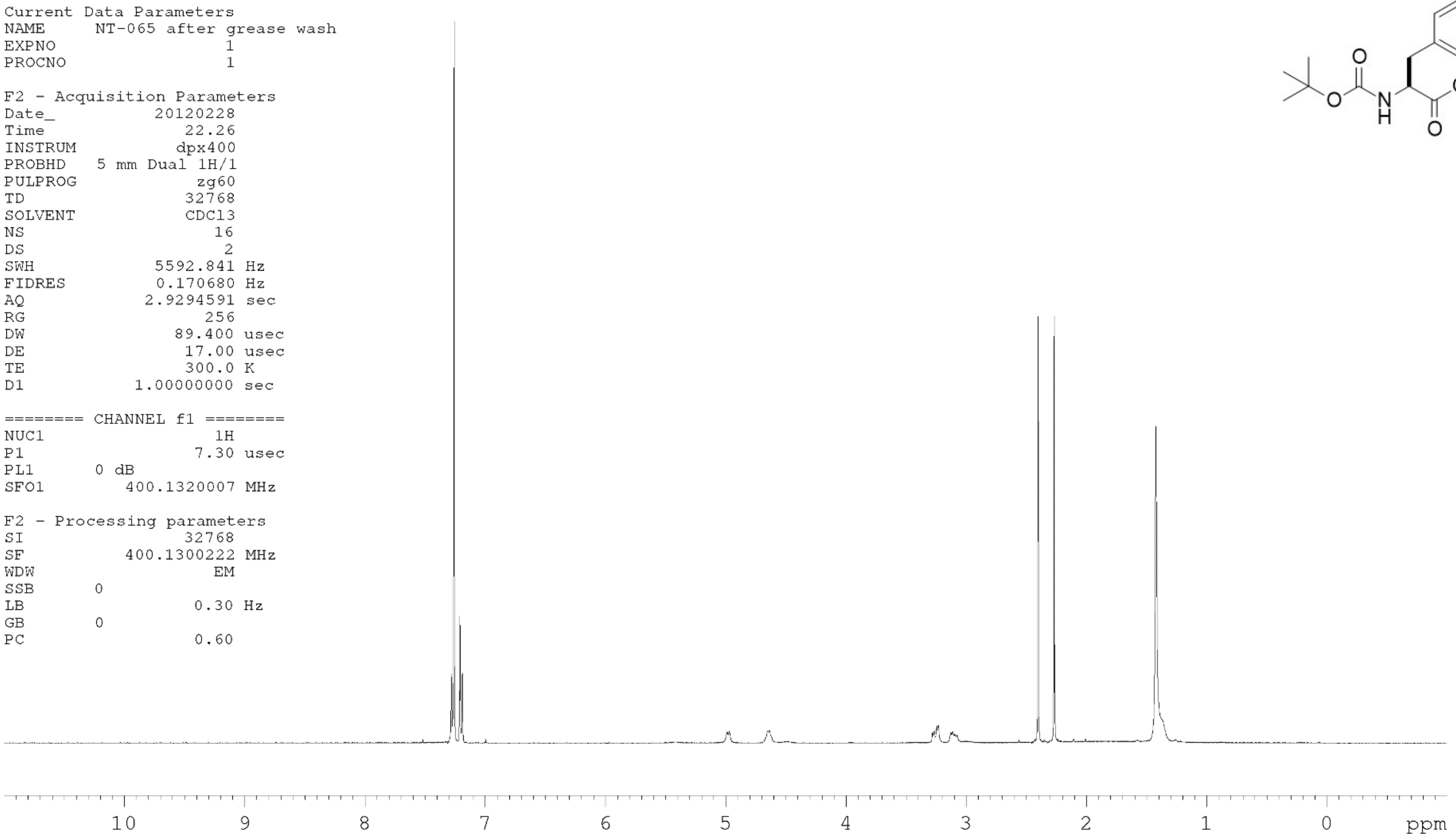

(*S*)-2-((*tert*-Butoxycarbonyl)amino)-3-(4-(3,5-dimethylisoxazol-4-yl)phenyl)propanoic acid **35** ^{13}C NMR

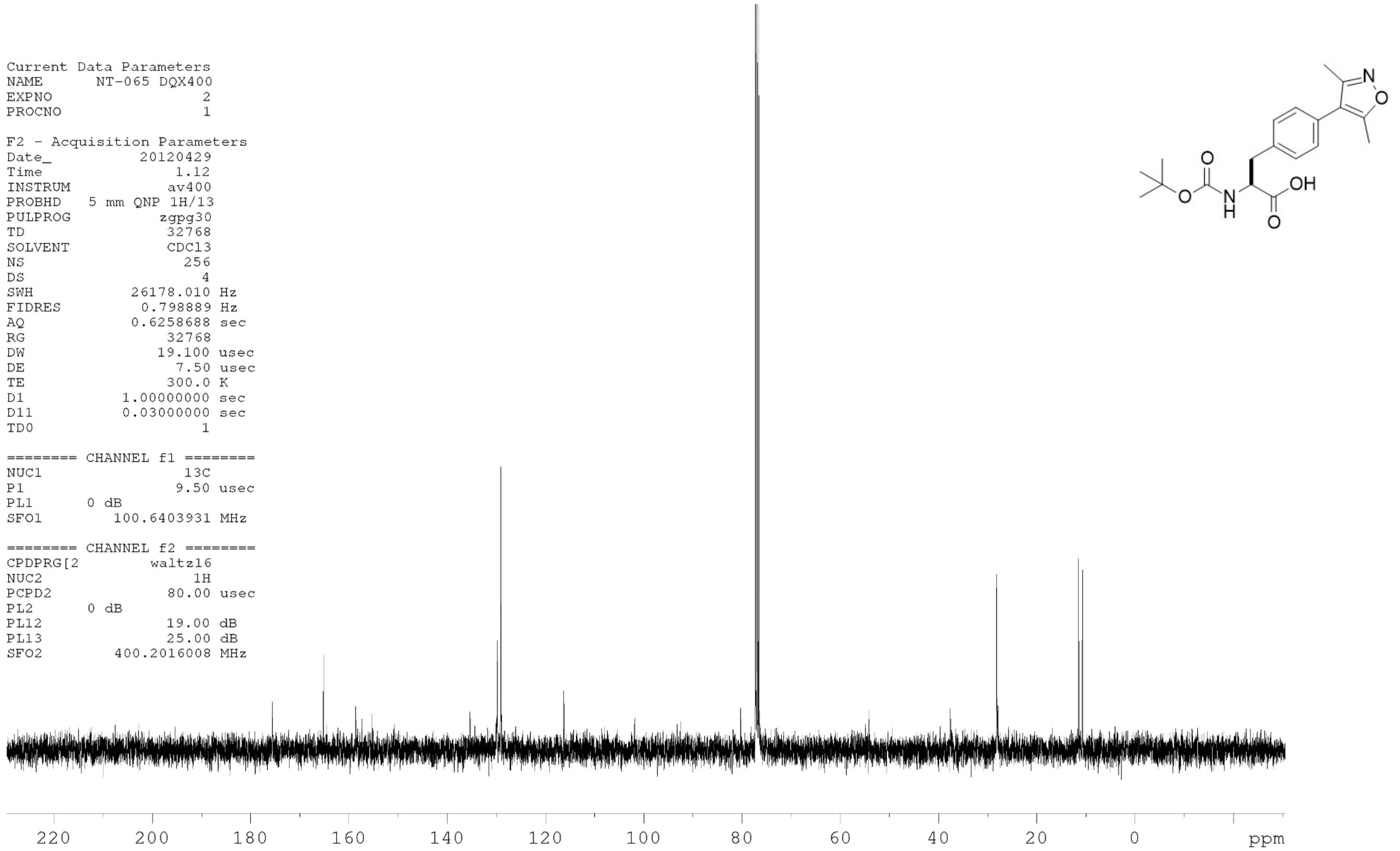

(*S*)-2-Amino-3-(4-(3,5-dimethylisoxazol-4-yl)phenyl)propanoic acid **9** ^{1}H NMR

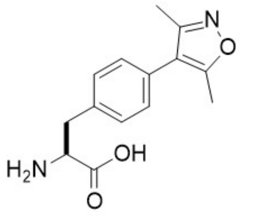

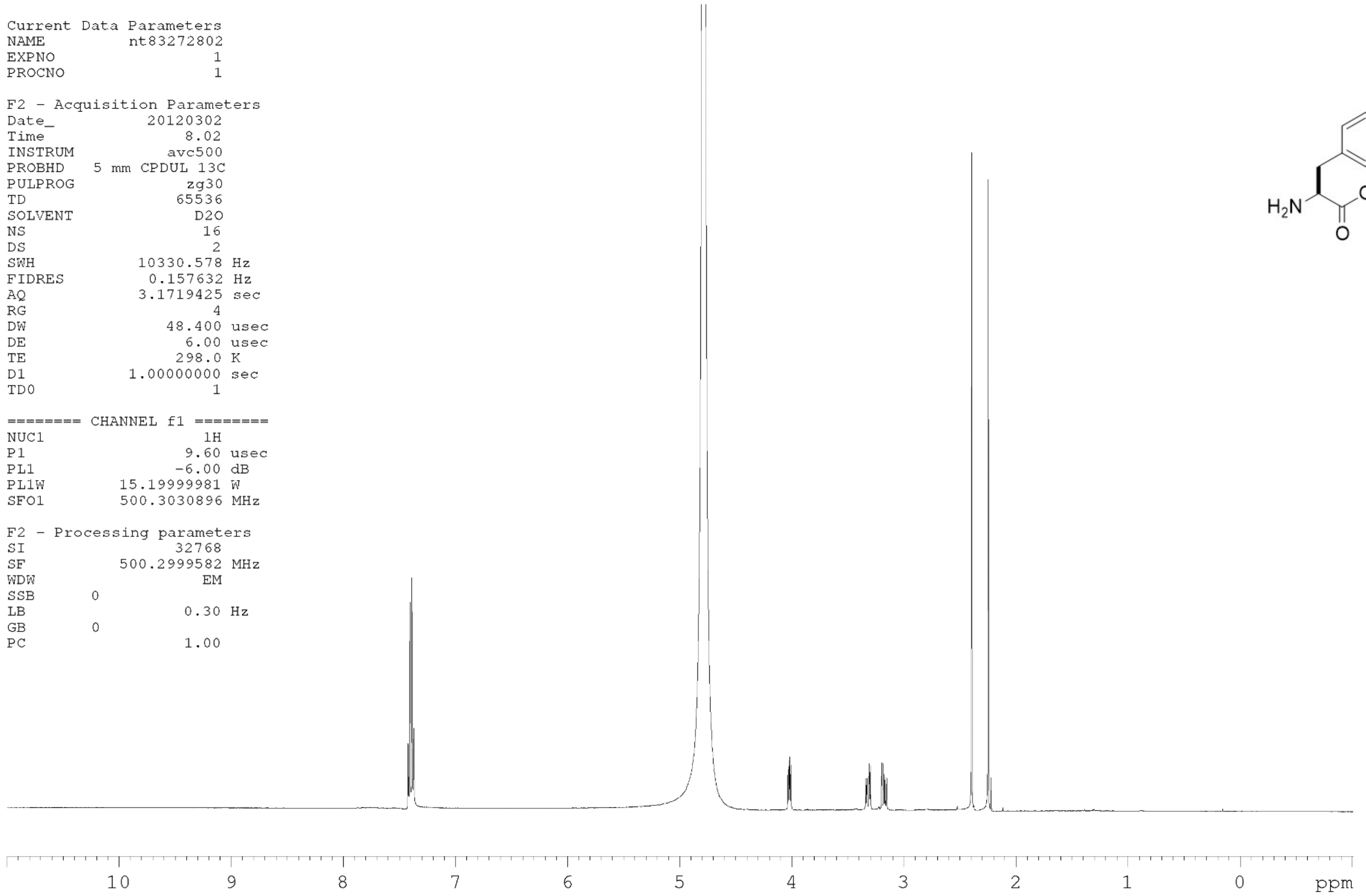

(*S*)-2-Amino-3-(4-(3,5-dimethylisoxazol-4-yl)phenyl)propanoic acid **9** ^{13}C NMR

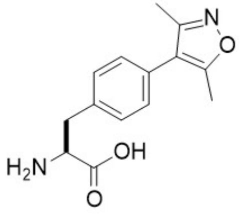

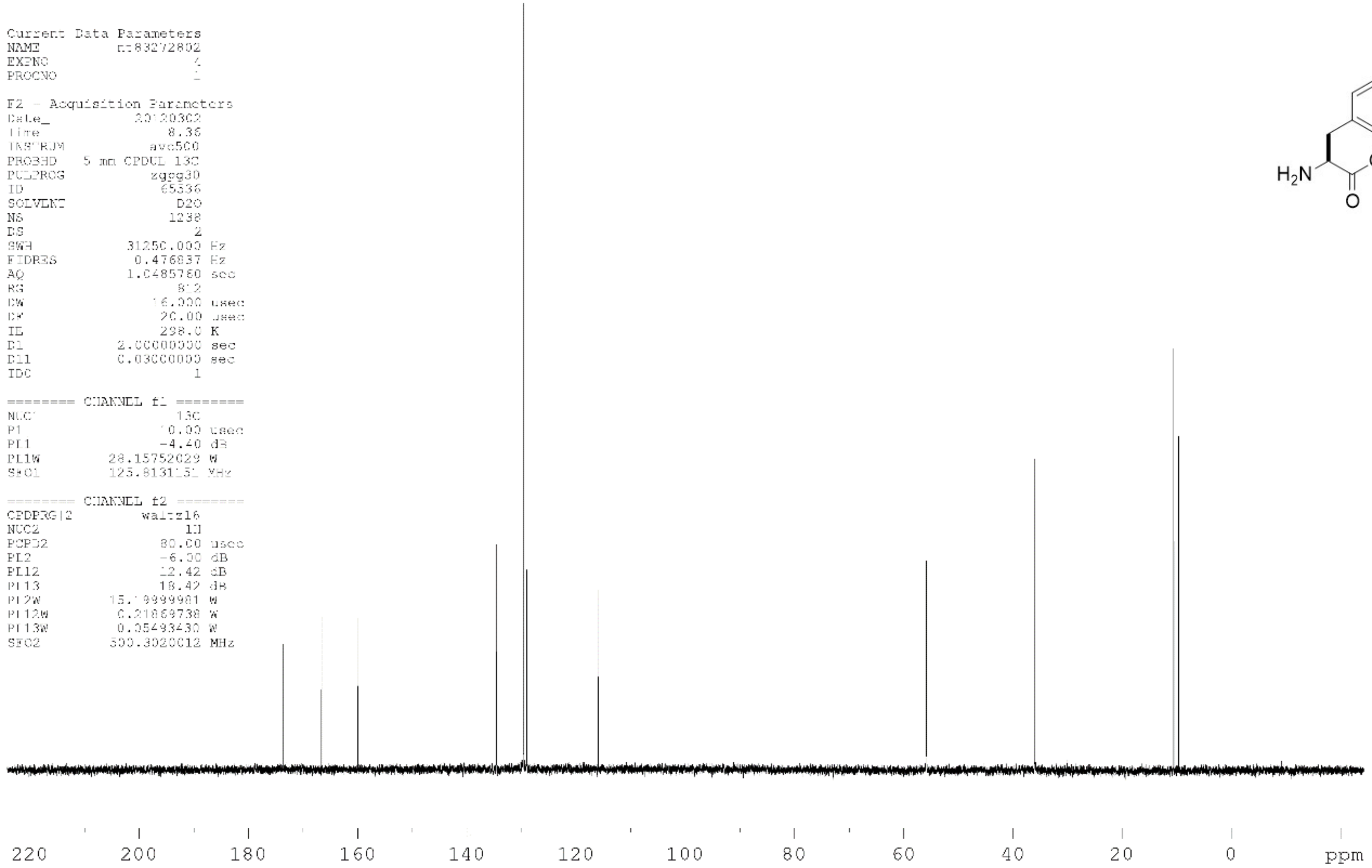

5-(3-Iodopropyl)-3-methylisoxazole **XX** ^{1}H NMR

5-(3-Iodopropyl)-3-methylisoxazole **41** ^{1}H NMR

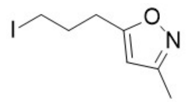

```
NAME                 340
EXPNO                  1
PROCNO                 1
Date_           20130417
Time               15.15
INSTRUM           avc500
PROBHD   5 mm CPDUL 13C
PULPROG             zg30
TD                 65536
SOLVENT            CDCl3
NS                    16
DS                     4
SWH            10330.578 Hz
FIDRES          0.157632 Hz
AQ             3.1719923 sec
RG                   2.8
DW                48.400 usec
DE                  6.00 usec
TE                 298.0 K
D1            1.00000000 sec
TD0                    1

======== CHANNEL f1 ========
NUC1                  1H
P1                 10.60 usec
PL1                 6.00 dB
PL1W          0.95905519 W
SFO1         500.3030896 MHz
SI                 32768
SF           500.3000240 MHz
WDW                   EM
SSB                    0
LB                  0.30 Hz
GB                     0
PC                  1.00
```

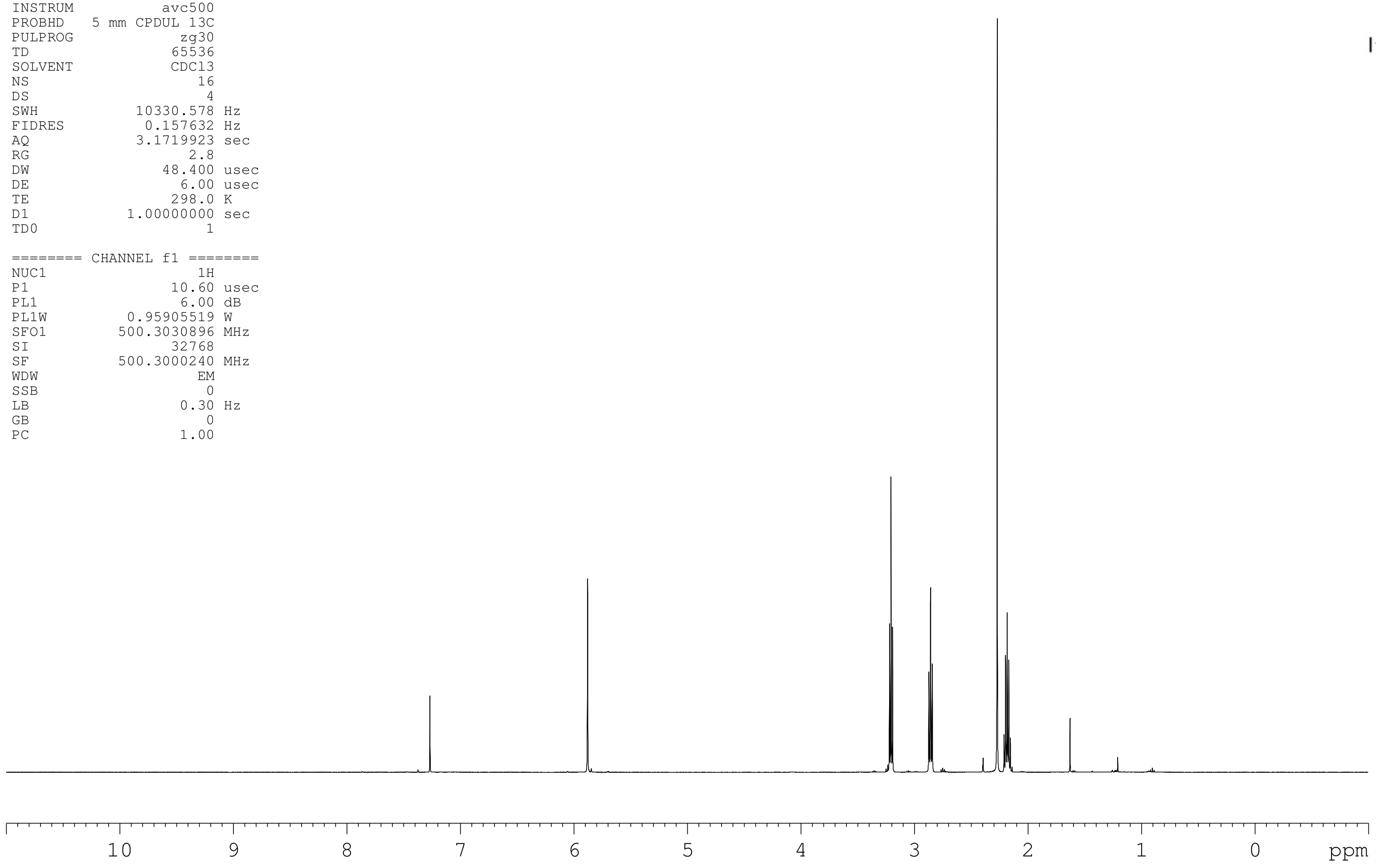

5-(3-Iodopropyl)-3-methylisoxazole **41** ^{13}C NMR

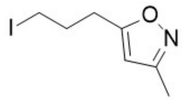

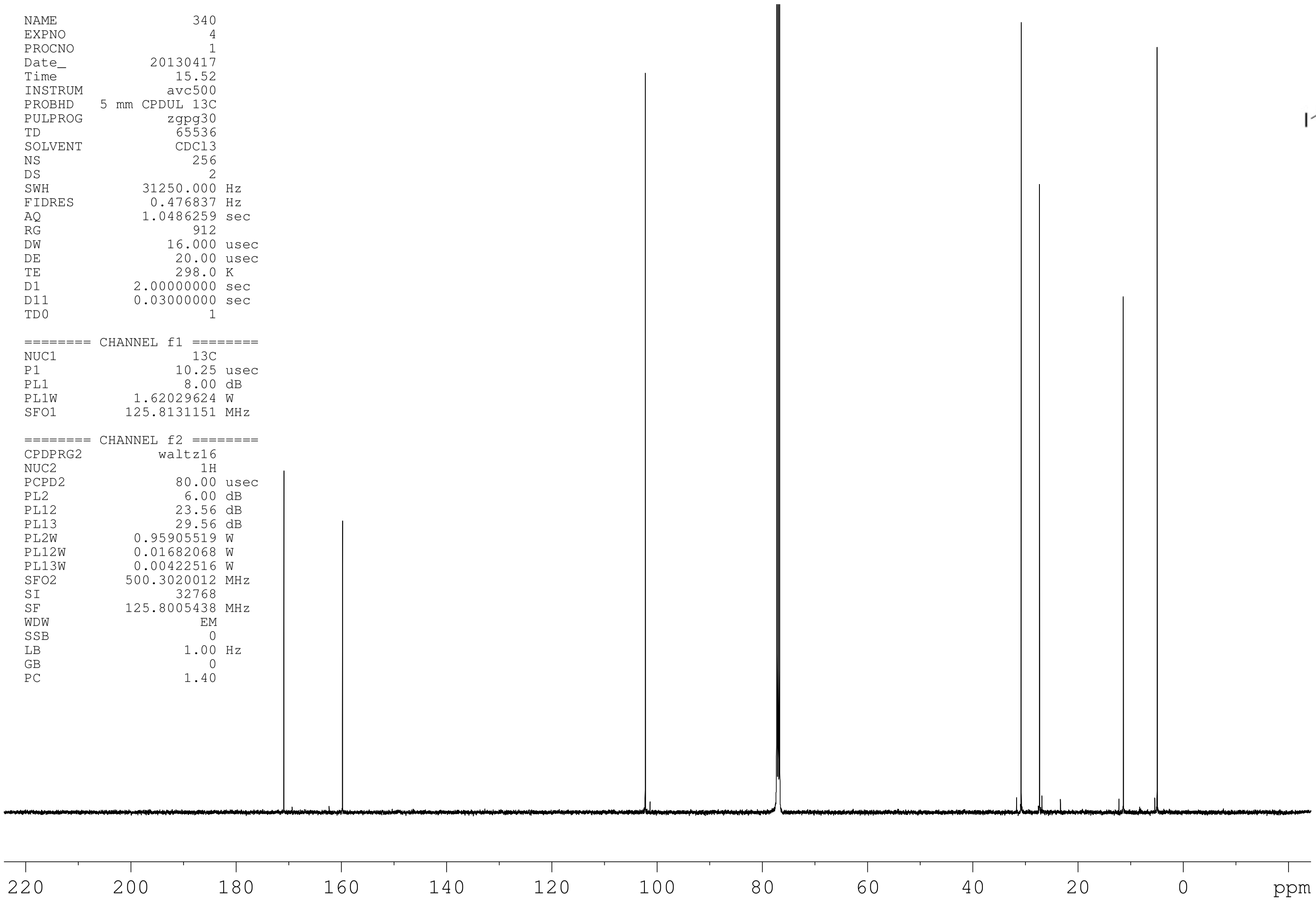

5-(3-((2*S*,5*R*)-5-Isopropyl-3,6-dimethoxy-2,5-dihydropyrazin-2-yl)propyl)-3-methylisoxazole **43** ^{1}H NMR

```
NAME                345
EXPNO                 1
PROCNO                1
Date_          20130429
Time               1.00
INSTRUM          avc500
PROBHD   5 mm CPDUL 13C
PULPROG            zg30
TD                65536
SOLVENT           CDCl3
NS                   16
DS                    4
SWH           10330.578 Hz
FIDRES         0.157632 Hz
AQ            3.1719923 sec
RG                 2.56
DW               48.400 usec
DE                 6.00 usec
TE                298.0 K
D1           1.00000000 sec
TD0                   1

======== CHANNEL f1 ========
NUC1                 1H
P1                10.60 usec
PL1                6.00 dB
PL1W         0.95905519 W
SFO1        500.3030896 MHz
SI                32768
SF          500.3000240 MHz
WDW                  EM
SSB                   0
LB                 0.30 Hz
GB                    0
PC                 1.00
```

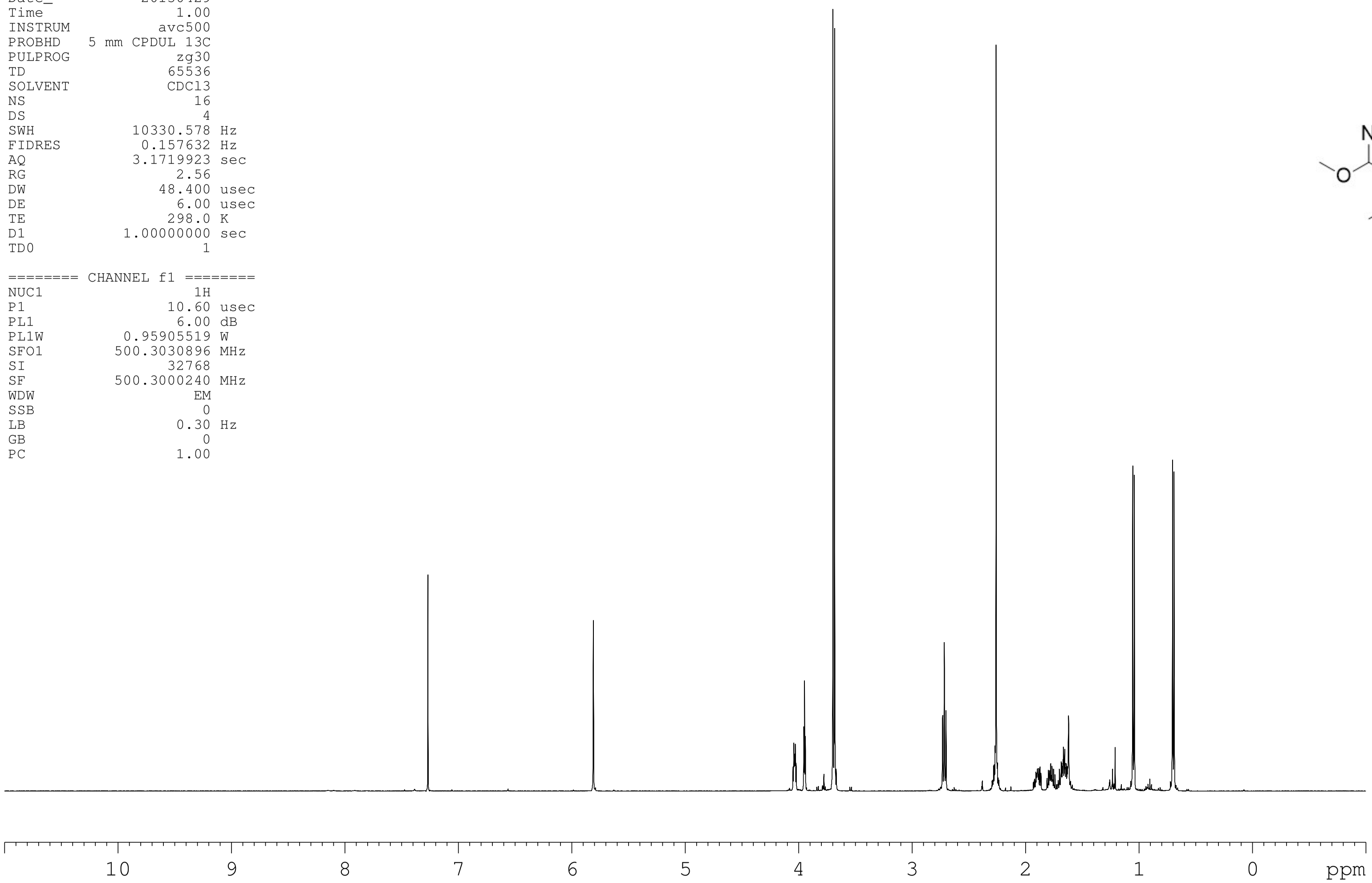

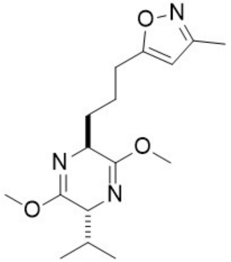

5-(3-((2*S*,5*R*)-5-Isopropyl-3,6-dimethoxy-2,5-dihydropyrazin-2-yl)propyl)-3-methylisoxazole **43** ^{13}C NMR

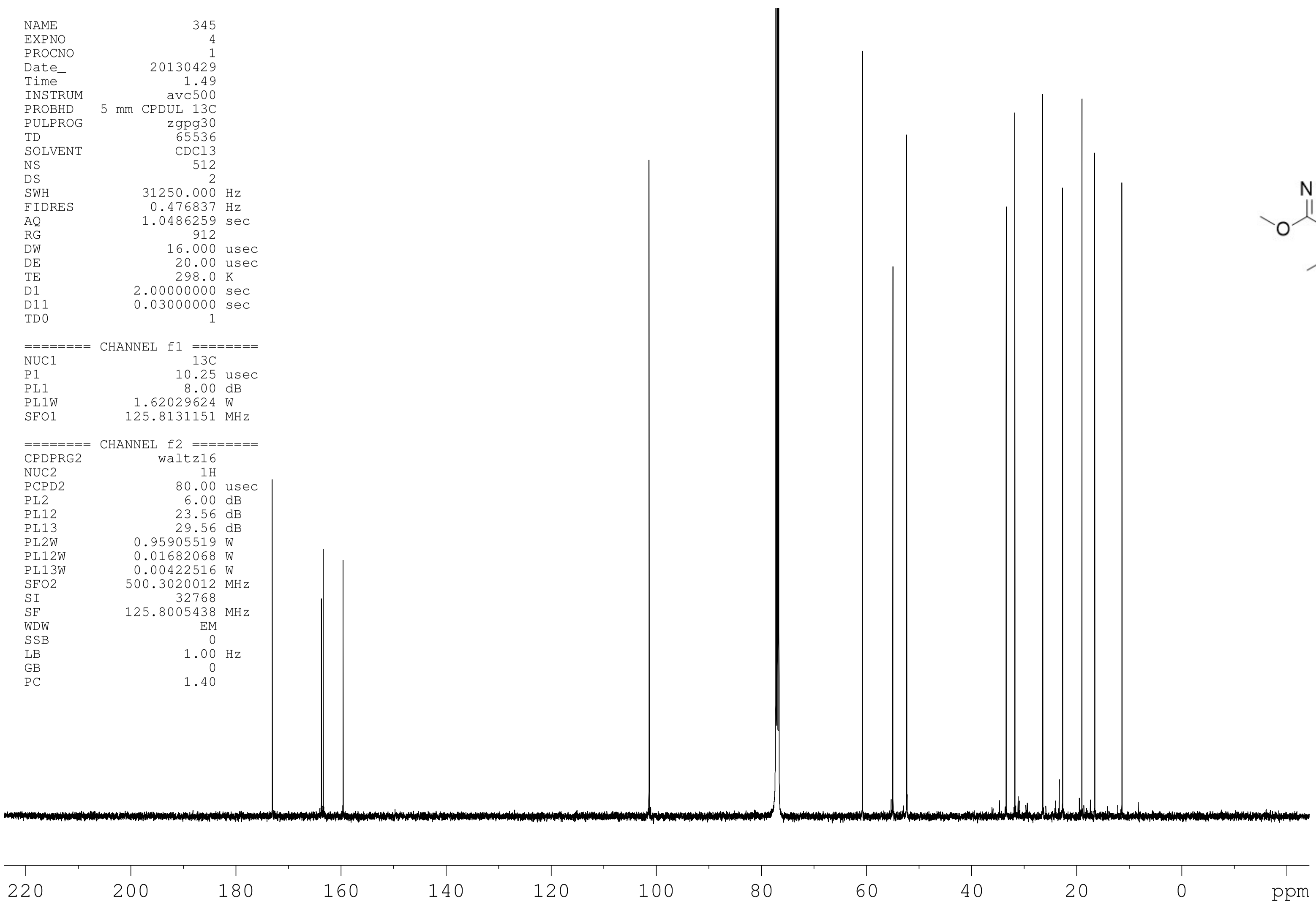

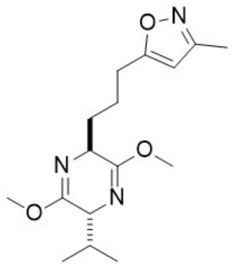

Methyl (*S*)-2-amino-5-(3-methylisoxazol-5-yl)pentanoate **44** ^{1}H NMR

```
NAME                348
EXPNO                 1
PROCNO                1
Date_          20130501
Time               8.43
INSTRUM          avc500
PROBHD   5 mm CPDUL 13C
PULPROG            zg30
TD                65536
SOLVENT           CDCl3
NS                   16
DS                    4
SWH          10330.578 Hz
FIDRES        0.157632 Hz
AQ           3.1719923 sec
RG                  2.8
DW               48.400 usec
DE                 6.00 usec
TE                298.0 K
D1           1.00000000 sec
TD0                   1

======== CHANNEL f1 ========
NUC1                 1H
P1                10.60 usec
PL1                6.00 dB
PL1W         0.95905519 W
SFO1        500.3030896 MHz
SI                32768
SF          500.3000240 MHz
WDW                  EM
SSB                   0
LB                 0.30 Hz
GB                    0
PC                 1.00
```

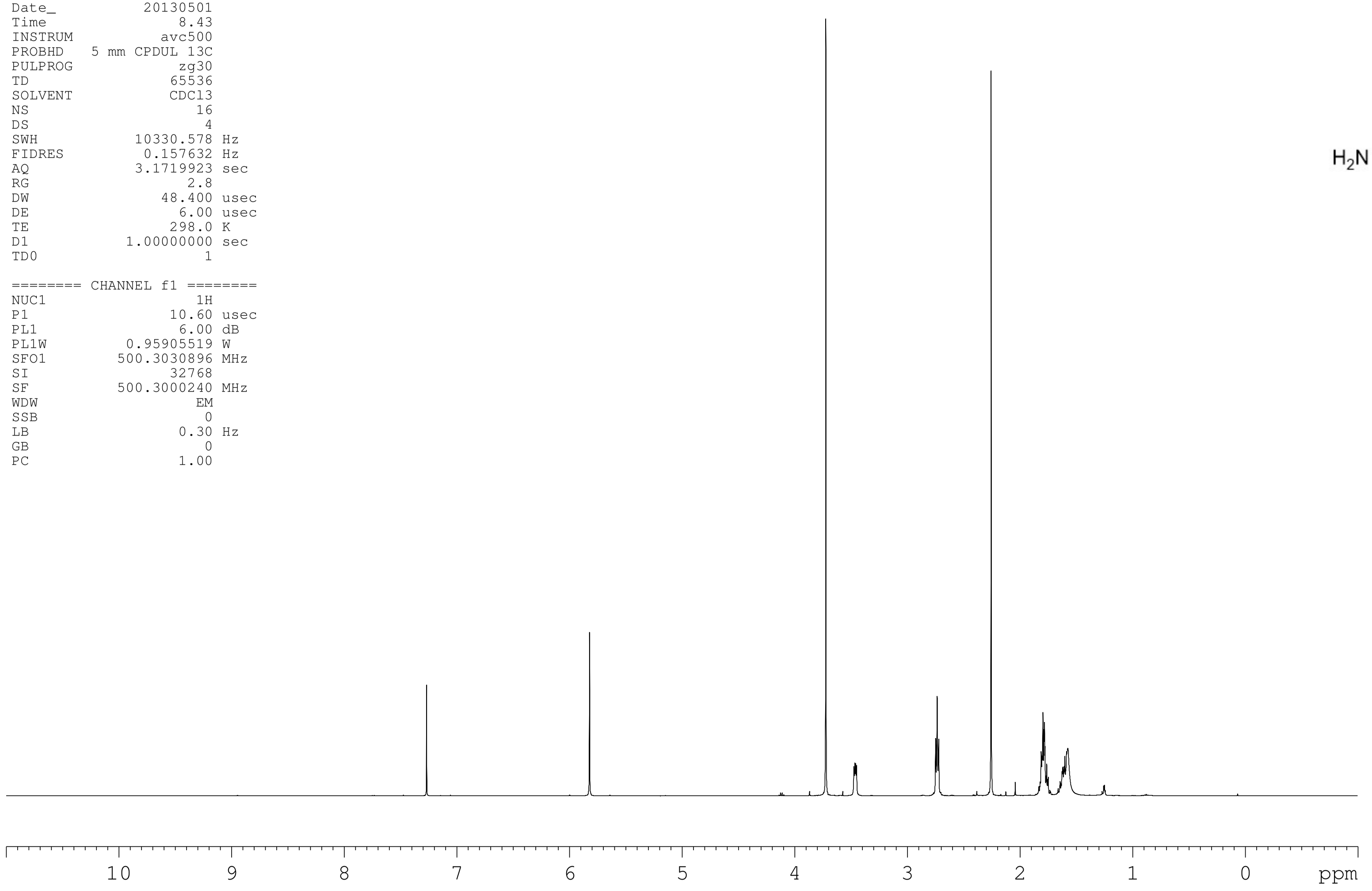

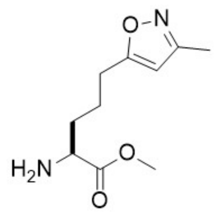

Methyl (*S*)-2-amino-5-(3-methylisoxazol-5-yl)pentanoate **44** ^{13}C NMR

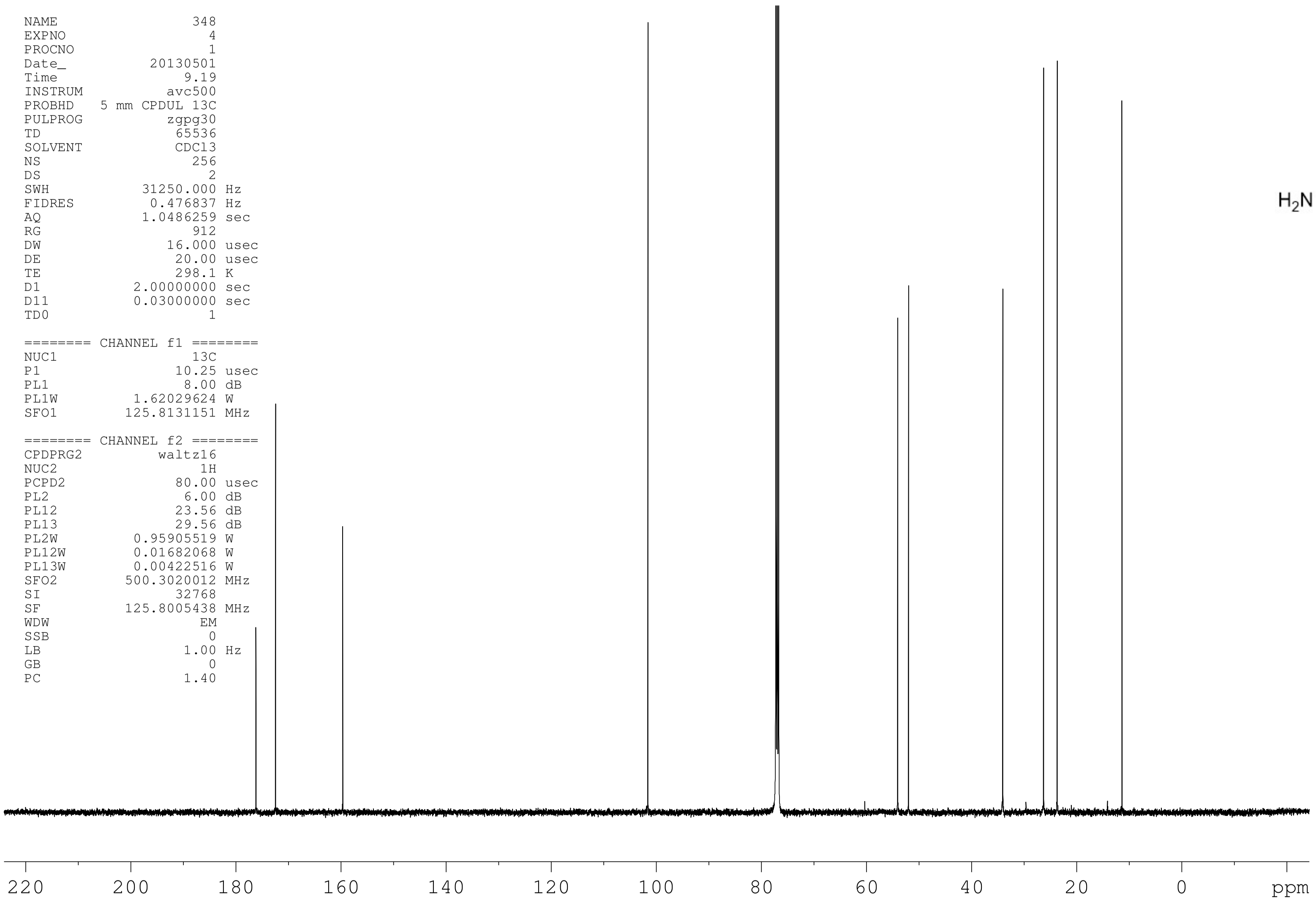

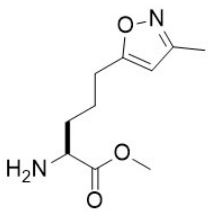


```
NAME                 348
EXPNO                  4
PROCNO                 1
Date_           20130501
Time                9.19
INSTRUM           avc500
PROBHD   5 mm CPDUL 13C
PULPROG          zgpg30
TD                 65536
SOLVENT            CDCl3
NS                   256
DS                     2
SWH            31250.000 Hz
FIDRES          0.476837 Hz
AQ             1.0486259 sec
RG                   912
DW                16.000 usec
DE                 20.00 usec
TE                 298.1 K
D1            2.00000000 sec
D11           0.03000000 sec
TD0                    1

======== CHANNEL f1 ========
NUC1                 13C
P1                 10.25 usec
PL1                 8.00 dB
PL1W          1.62029624 W
SFO1        125.8131151 MHz

======== CHANNEL f2 ========
CPDPRG2          waltz16
NUC2                  1H
PCPD2              80.00 usec
PL2                 6.00 dB
PL12               23.56 dB
PL13               29.56 dB
PL2W          0.95905519 W
PL12W         0.01682068 W
PL13W         0.00422516 W
SFO2        500.3020012 MHz
SI                 32768
SF          125.8005438 MHz
WDW                   EM
SSB                    0
LB                  1.00 Hz
GB                     0
PC                  1.40
```

Methyl (*S*)-2-amino-5-(3-methylisoxazol-5-yl)pentanoate **3** ^{1}H NMR

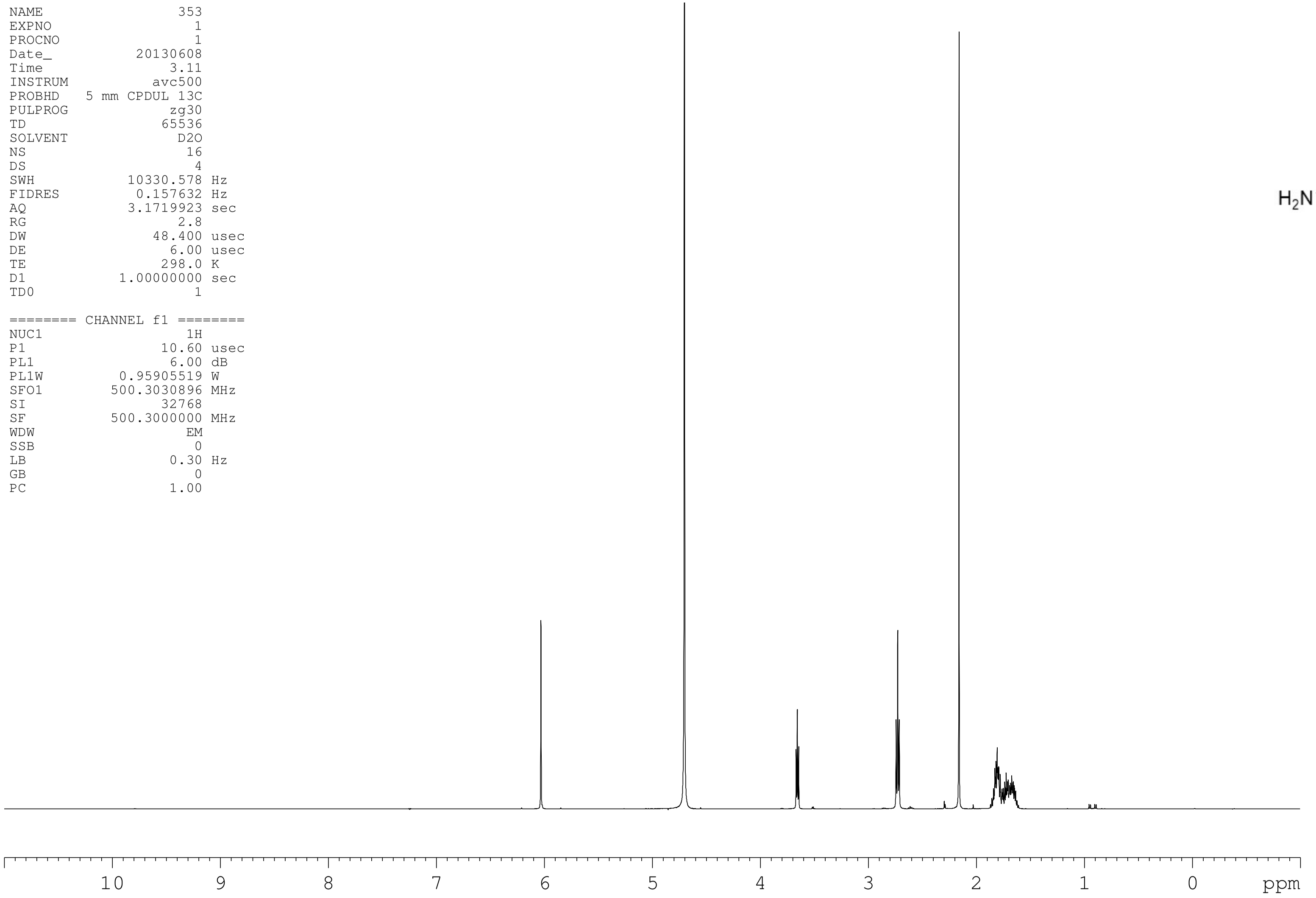

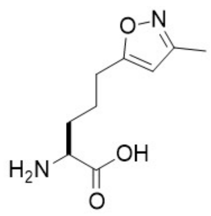

Methyl (*S*)-2-amino-5-(3-methylisoxazol-5-yl)pentanoate **3** ^{13}C NMR

```
NAME                353
EXPNO                 4
PROCNO                1
Date_          20130608
Time               3.47
INSTRUM          avc500
PROBHD   5 mm CPDUL 13C
PULPROG         zgpg30
TD                65536
SOLVENT             D2O
NS                  256
DS                    2
SWH           31250.000 Hz
FIDRES         0.476837 Hz
AQ            1.0486259 sec
RG                  912
DW               16.000 usec
DE                20.00 usec
TE                298.0 K
D1           2.00000000 sec
D11          0.03000000 sec
TD0                   1

======== CHANNEL f1 ========
NUC1                13C
P1                10.25 usec
PL1                8.00 dB
PL1W         1.62029624 W
SFO1        125.8131151 MHz

======== CHANNEL f2 ========
CPDPRG2         waltz16
NUC2                 1H
PCPD2             80.00 usec
PL2                6.00 dB
PL12              23.56 dB
PL13              29.56 dB
PL2W         0.95905519 W
PL12W        0.01682068 W
PL13W        0.00422516 W
SFO2        500.3020012 MHz
SI                32768
SF          125.8005350 MHz
WDW                  EM
SSB                   0
LB                 1.00 Hz
GB                    0
PC                 1.40
```

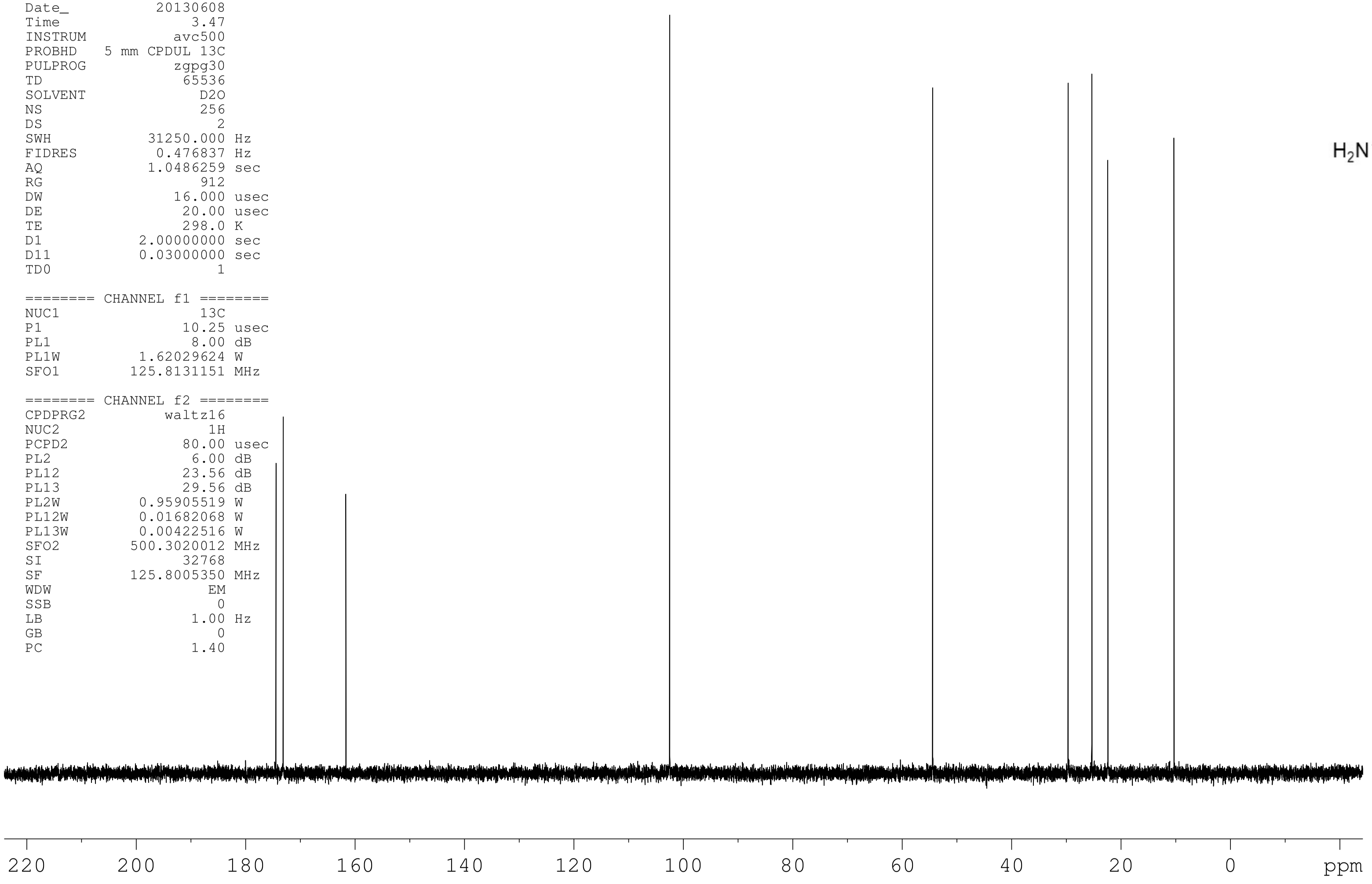

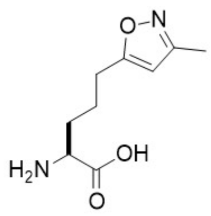

(*S*)-2-((((9*H*-Fluoren-9-yl)methoxy)carbonyl)amino)-5-(3-methylisoxazol-5-yl)pentanoic acid **45** ^{1}H NMR

NAME	386
EXPNO	1
PROCNO	1
Date_	20130817
Time	10.20
INSTRUM	avc500
PROBHD	5 mm CPDUL 13C
PULPROG	zg30
TD	65536
SOLVENT	DMSO
NS	16
DS	4
SWH	10330.578 Hz
FIDRES	0.157632 Hz
AQ	3.1719923 sec
RG	3.2
DW	48.400 usec
DE	6.00 usec
TE	298.0 K
D1	1.00000000 sec
TD0	1
======== CHANNEL f1 ========	
NUC1	1H
P1	10.60 usec
PL1	6.00 dB
PL1W	0.95905519 W
SFO1	500.3030896 MHz
SI	32768
SF	500.3000000 MHz
WDW	EM
SSB	0
LB	0.30 Hz
GB	0
PC	1.00

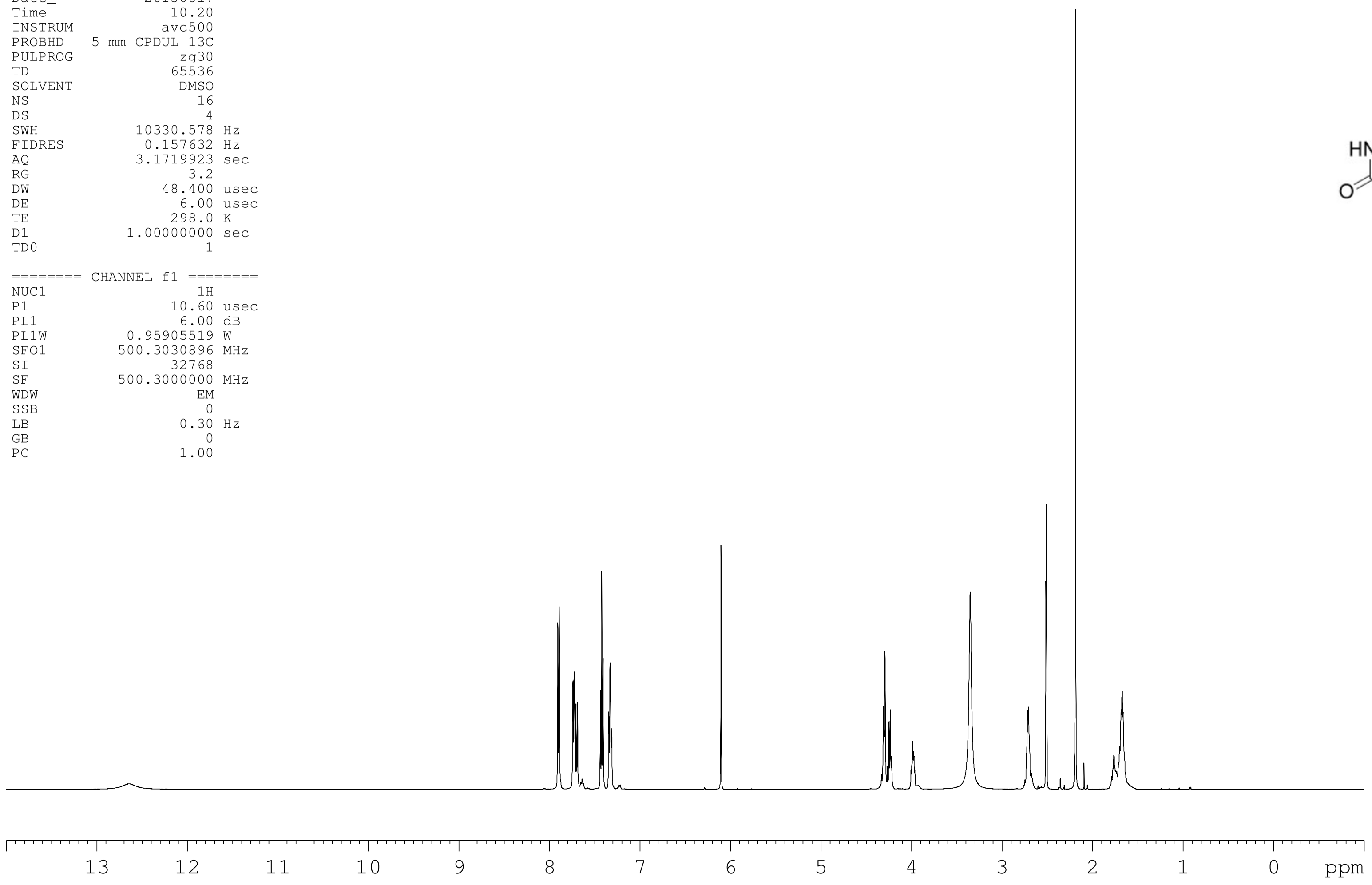

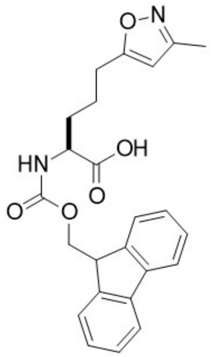

(*S*)-2-((((9*H*-Fluoren-9-yl)methoxy)carbonyl)amino)-5-(3-methylisoxazol-5-yl)pentanoic acid **45** ^{13}C NMR

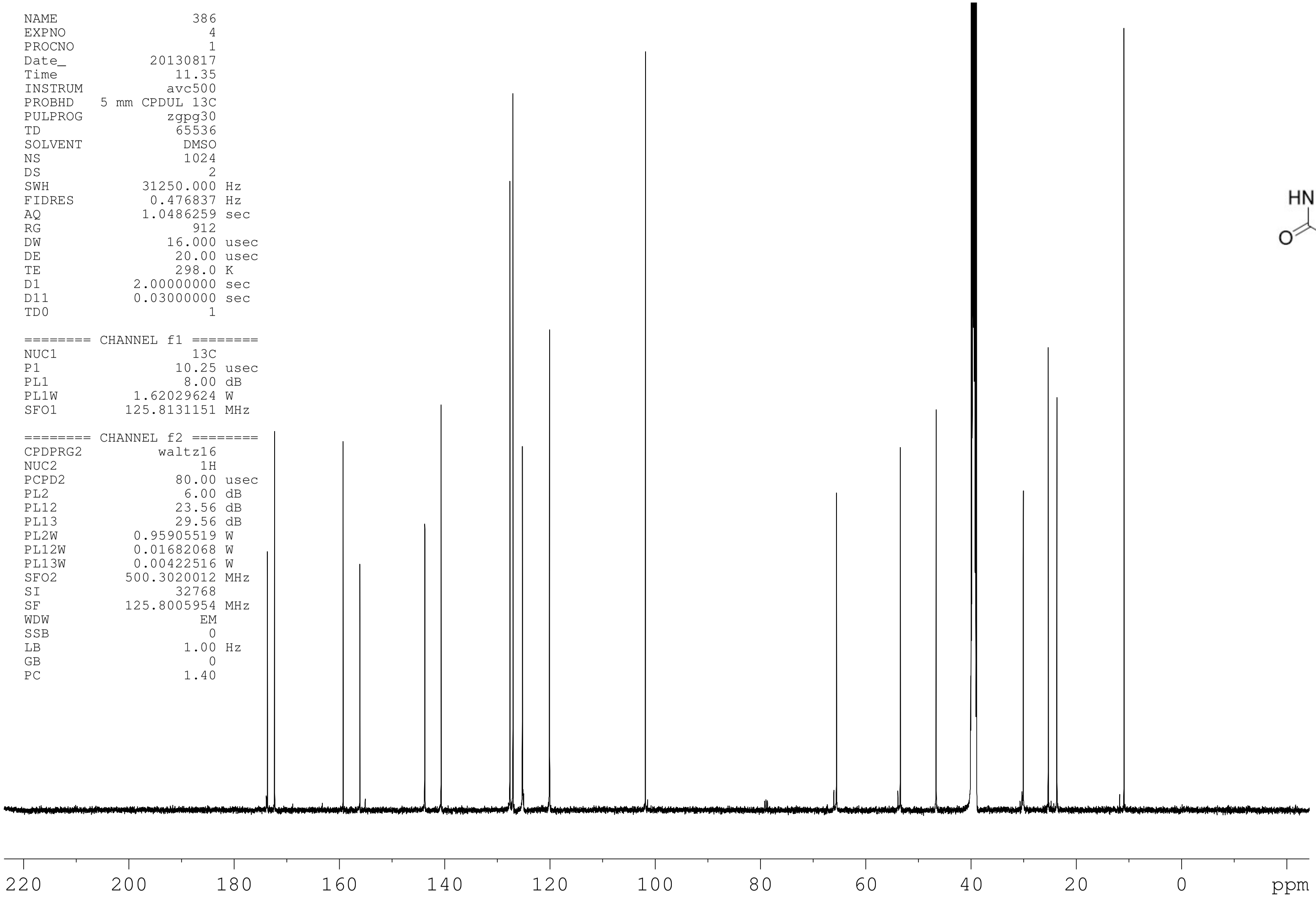

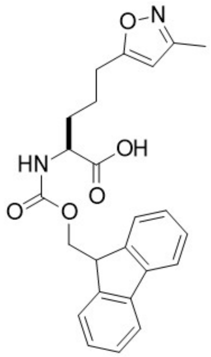

N-(*tert*-Butoxycarbonyl)-*O*-((3-methylisoxazol-5-yl)methyl)-L-serine **54** ^{1}H NMR

```
NAME                 409
EXPNO                  1
PROCNO                 1
Date_           20130930
Time               19.19
INSTRUM           avc500
PROBHD   5 mm CPDUL 13C
PULPROG             zg30
TD                 65536
SOLVENT            CDCl3
NS                    16
DS                     4
SWH           10330.578 Hz
FIDRES         0.157632 Hz
AQ            3.1719923 sec
RG                     4
DW                48.400 usec
DE                 10.00 usec
TE                 298.0 K
D1            1.00000000 sec
TD0                    1

======== CHANNEL f1 ========
SFO1         500.3030896 MHz
NUC1                  1H
P1                 15.00 usec
SI                 32768
SF           500.3000000 MHz
WDW                   EM
SSB                    0
LB                  0.30 Hz
GB                     0
PC                  1.00
```

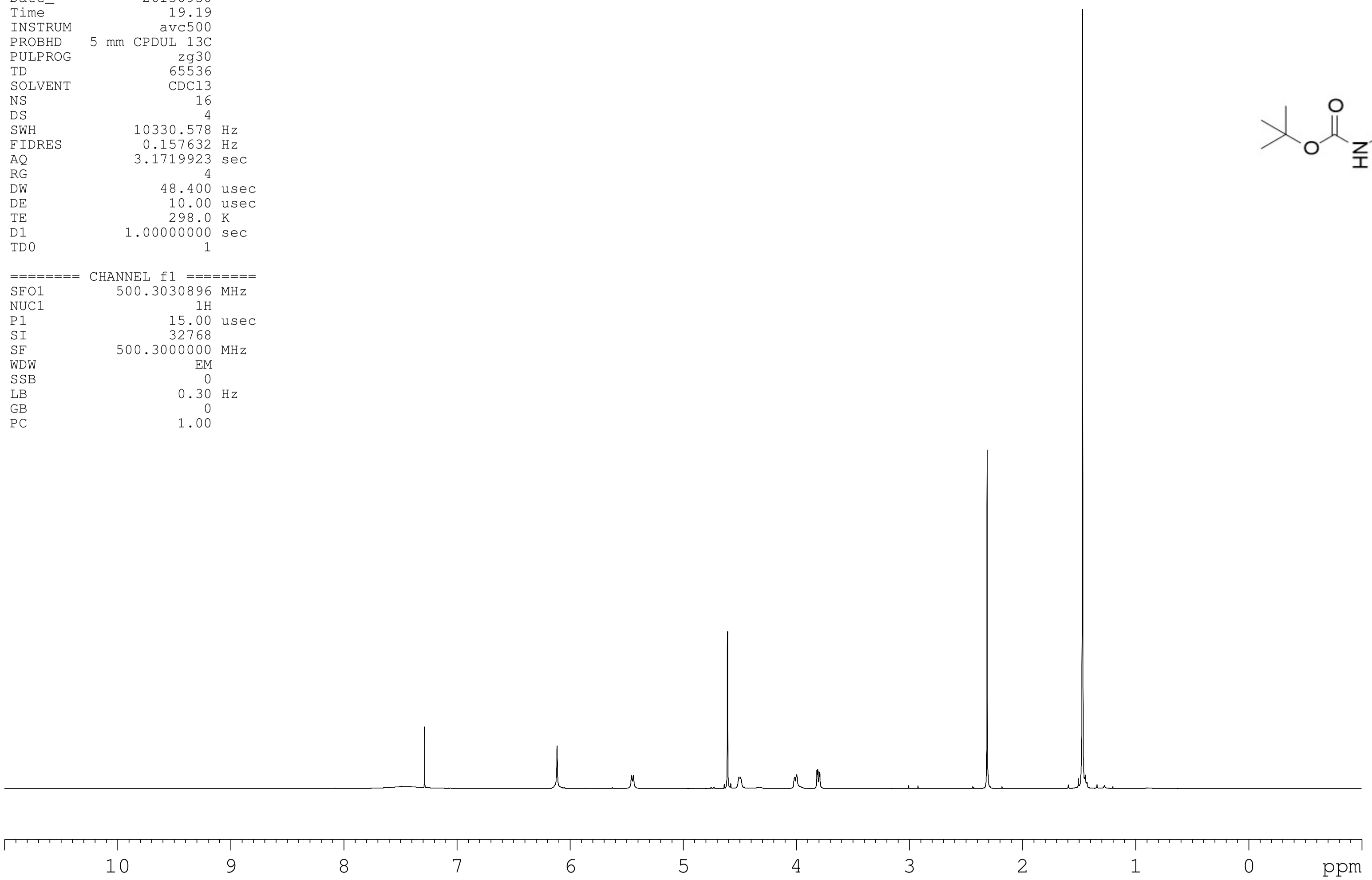

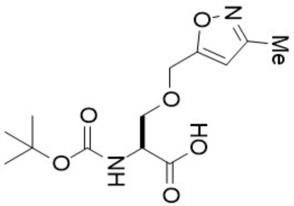

N-(*tert*-Butoxycarbonyl)-*O*-((3-methylisoxazol-5-yl)methyl)-L-serine **54** ^{13}C NMR

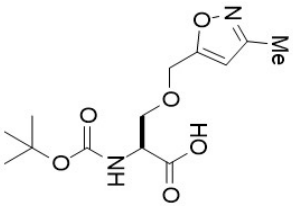

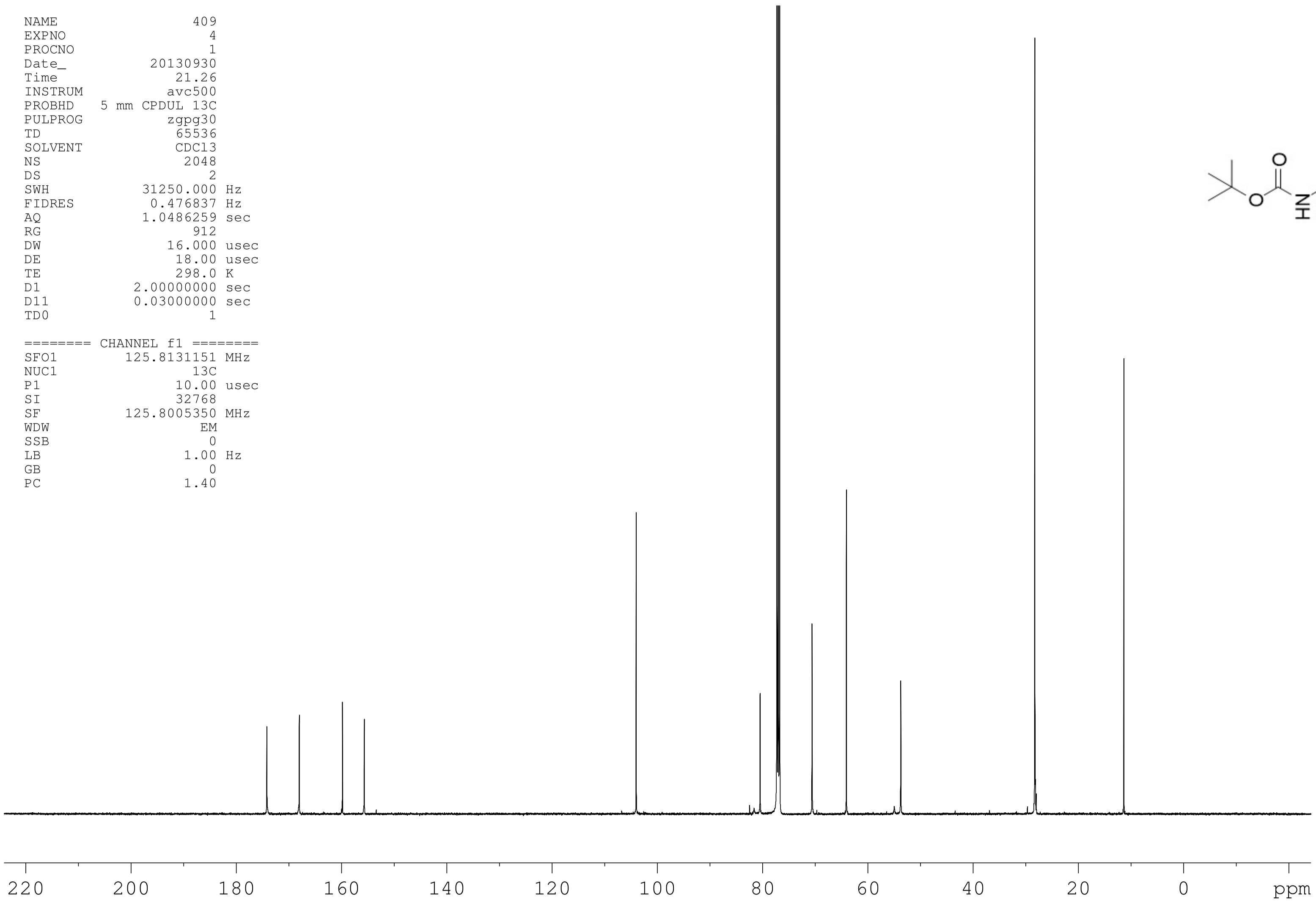


```
NAME                409
EXPNO                 4
PROCNO                1
Date_          20130930
Time              21.26
INSTRUM          avc500
PROBHD   5 mm CPDUL 13C
PULPROG         zgpg30
TD                65536
SOLVENT           CDCl3
NS                 2048
DS                    2
SWH           31250.000 Hz
FIDRES         0.476837 Hz
AQ            1.0486259 sec
RG                  912
DW               16.000 usec
DE                18.00 usec
TE                298.0 K
D1           2.00000000 sec
D11          0.03000000 sec
TD0                   1

======== CHANNEL f1 ========
SFO1        125.8131151 MHz
NUC1                13C
P1                10.00 usec
SI                32768
SF          125.8005350 MHz
WDW                  EM
SSB                   0
LB                 1.00 Hz
GB                    0
PC                 1.40
```

O-((3-Methylisoxazol-5-yl)methyl)-L-serine, trifluoroacetic acid salt **4** ^{1}H NMR

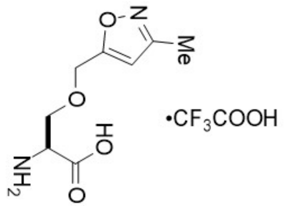


```
NAME                410
EXPNO                 1
PROCNO                1
Date_          20131005
Time              12.20
INSTRUM          avc500
PROBHD   5 mm CPDUL 13C
PULPROG            zg30
TD                65536
SOLVENT            MeOD
NS                   16
DS                    4
SWH           10330.578 Hz
FIDRES         0.157632 Hz
AQ            3.1719923 sec
RG                  4.5
DW               48.400 usec
DE                10.00 usec
TE                298.0 K
D1           1.00000000 sec
TD0                   1

======== CHANNEL f1 ========
SFO1        500.3030896 MHz
NUC1                 1H
P1                15.00 usec
SI                32768
SF          500.3000000 MHz
WDW                  EM
SSB                   0
LB                 0.30 Hz
GB                    0
PC                 1.00
```

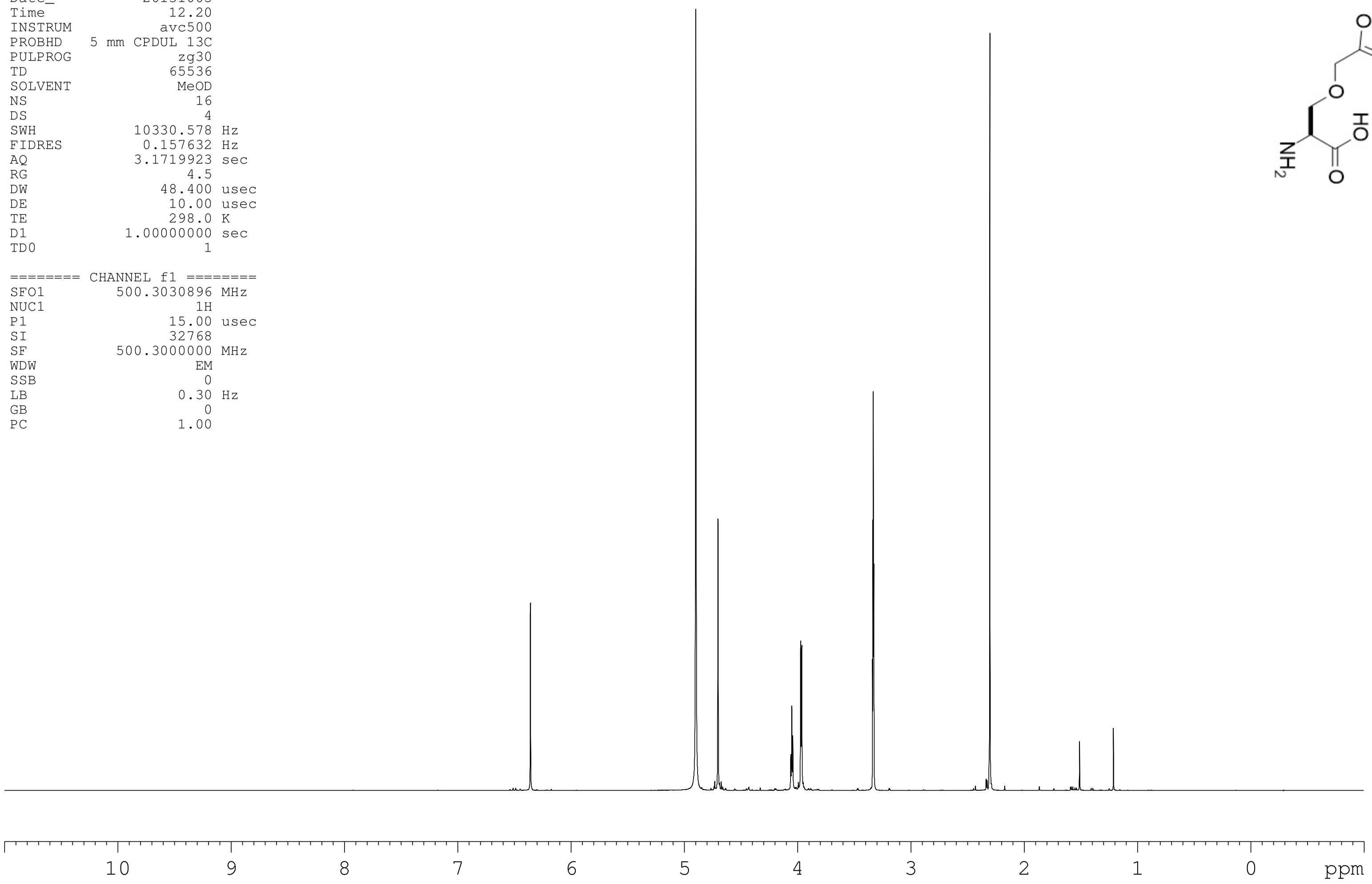

O-((3-Methylisoxazol-5-yl)methyl)-L-serine, trifluoroacetic acid salt **4** ^{13}C NMR

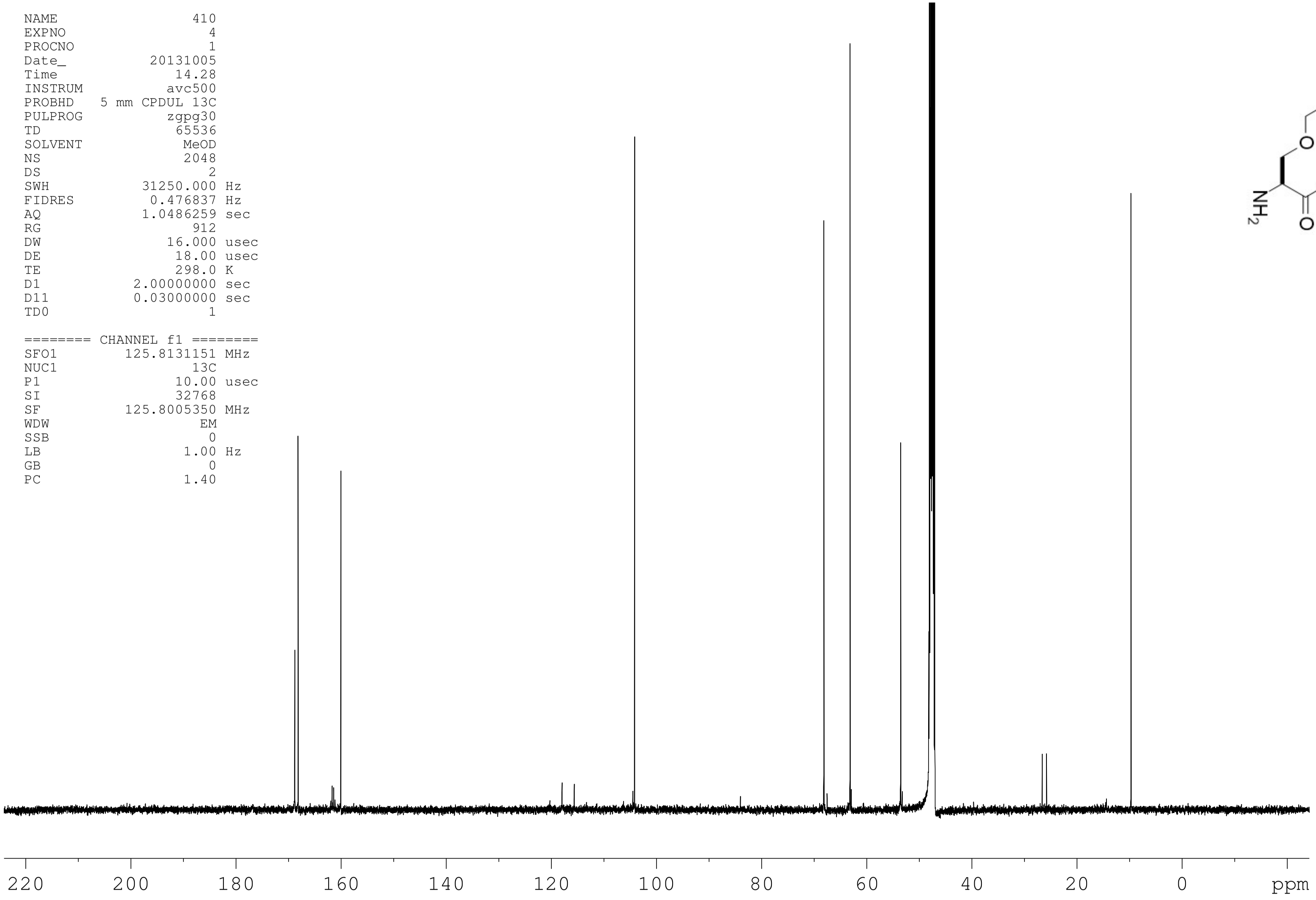

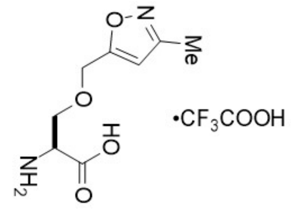

O-((3-Methylisoxazol-5-yl)methyl)-L-serine, trifluoroacetic acid salt **4** ^{19}F NMR

```
NAME           410 19F
EXPNO                1
PROCNO               1
Date_         20131007
Time             10.03
INSTRUM         avb500
PROBHD   5 mm PATXI 1H/
PULPROG       zgfhigqn
TD              131072
SOLVENT          CDCl3
NS                  16
DS                   4
SWH         113636.367 Hz
FIDRES        0.866977 Hz
AQ           0.5767668 sec
RG                2050
DW               4.400 usec
DE                6.50 usec
TE               298.0 K
D1          1.00000000 sec
D11         0.03000000 sec
D12         0.00002000 sec
TD0                  1

======== CHANNEL f1 ========
SFO1       470.4041911 MHz
NUC1               19F
P1               10.00 usec
SI               65536
SF         470.4512360 MHz
WDW                 EM
SSB                  0
LB                0.30 Hz
GB                   0
PC                1.00
```

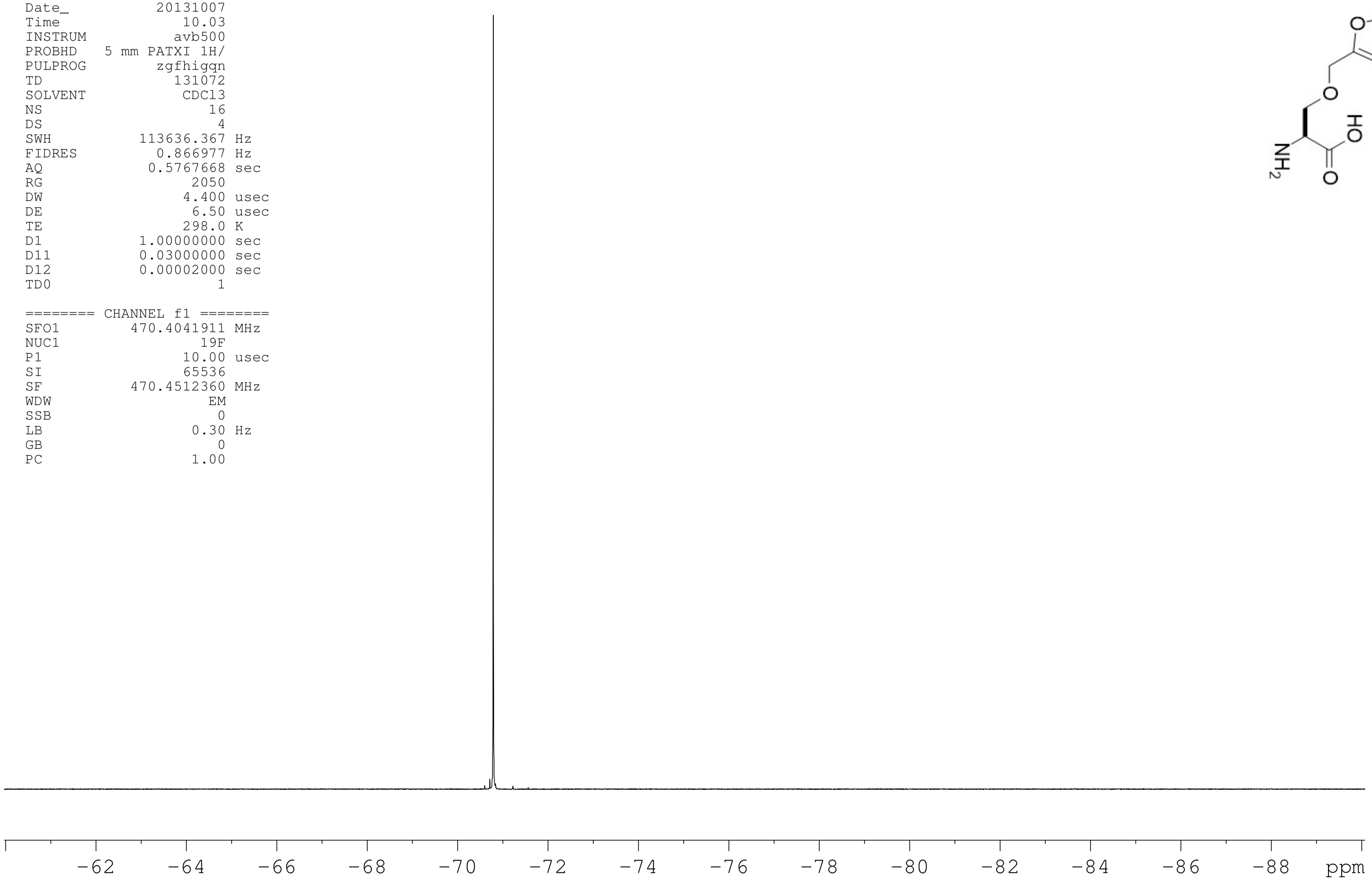

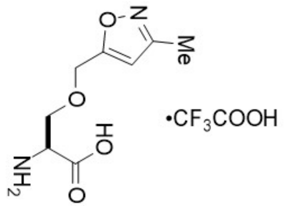

N-(((9*H*-Fluoren-9-yl)methoxy)carbonyl)-*O*-((3-methylisoxazol-5-yl)methyl)-L-serine **55** ^{1}H NMR

```
NAME        414 400 MHz
EXPNO                 1
PROCNO                1
Date_          20131013
Time               0.28
INSTRUM          avb400
PROBHD   5 mm PABBO BB/
PULPROG            zg30
TD                65536
SOLVENT           CDCl3
NS                   16
DS                    2
SWH            8012.820 Hz
FIDRES         0.122266 Hz
AQ            4.0894966 sec
RG               126.79
DW               62.400 usec
DE                 6.50 usec
TE                298.0 K
D1           1.00000000 sec
TD0                   1

======== CHANNEL f1 ========
SFO1        400.1320007 MHz
NUC1                 1H
P1                10.00 usec
SI                65536
SF          400.1300000 MHz
WDW                  EM
SSB                   0
LB                 0.30 Hz
GB                    0
PC                 1.00
```

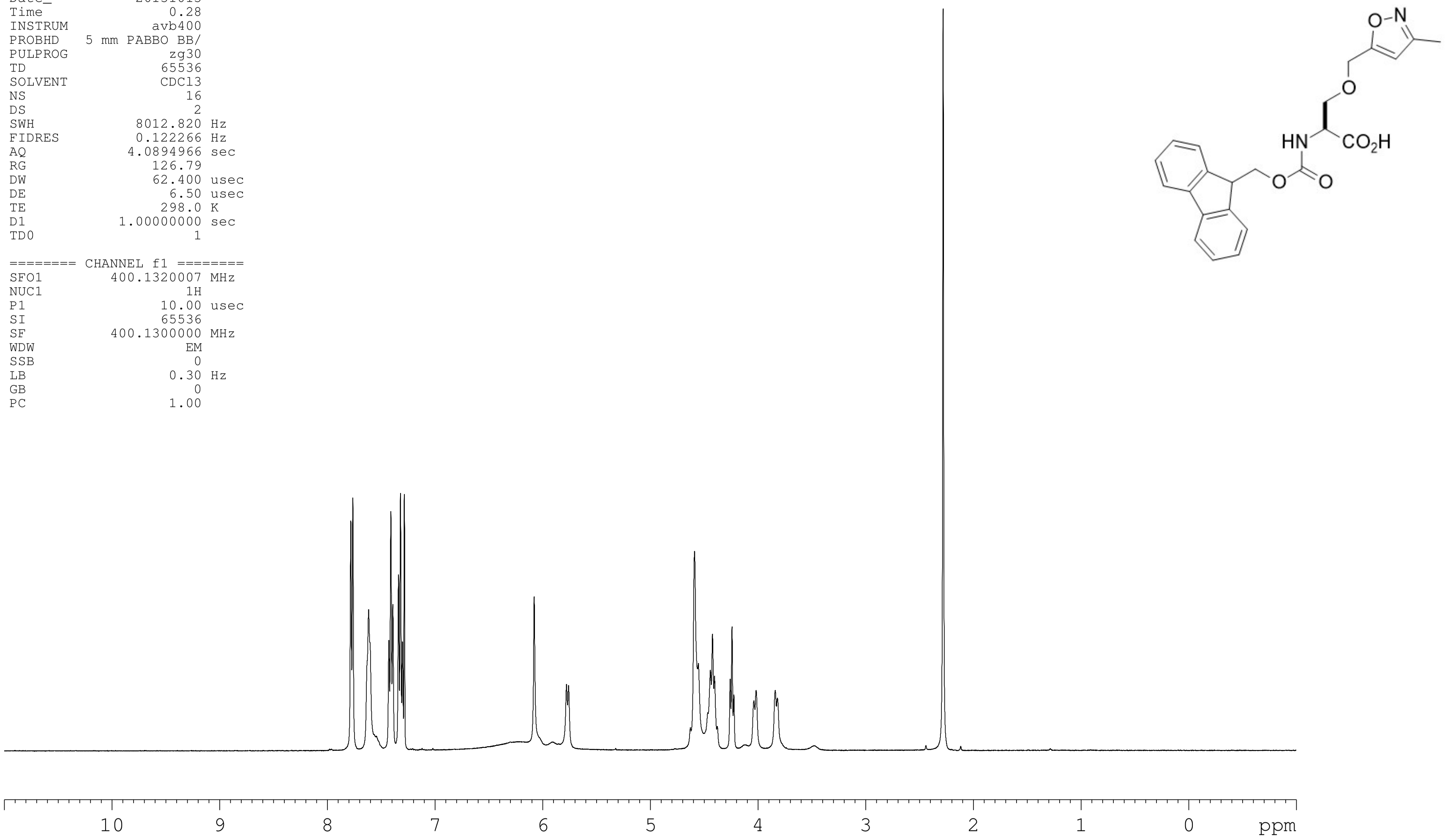

N-(((9*H*-Fluoren-9-yl)methoxy)carbonyl)-*O*-((3-methylisoxazol-5-yl)methyl)-L-serine **55** ^{13}C NMR

```
NAME        414 400 MHz
EXPNO
PROCNO
Date_
Time
INSTRUM
PROBHD    5 mm PABBO BB/
PULPROG          zgpg30
TD                65536
SOLVENT           CDCl3
NS                 2048
DS                    4
SWH           24038.461 Hz
FIDRES         0.366798 Hz
AQ            1.3631988 sec
RG               197.74
DW               20.800 usec
DE                 6.50 usec
TE                298.0 K
D1           2.00000000 sec
D11          0.03000000 sec
TD0                   1

======== CHANNEL f1 ========
SFO1        100.6228303 MHz
NUC1                13C
P1                10.00 usec
SI                32768
SF          100.6127690 MHz
WDW                  EM
SSB                   0
LB                 1.00 Hz
GB                    0
PC                 1.40
```

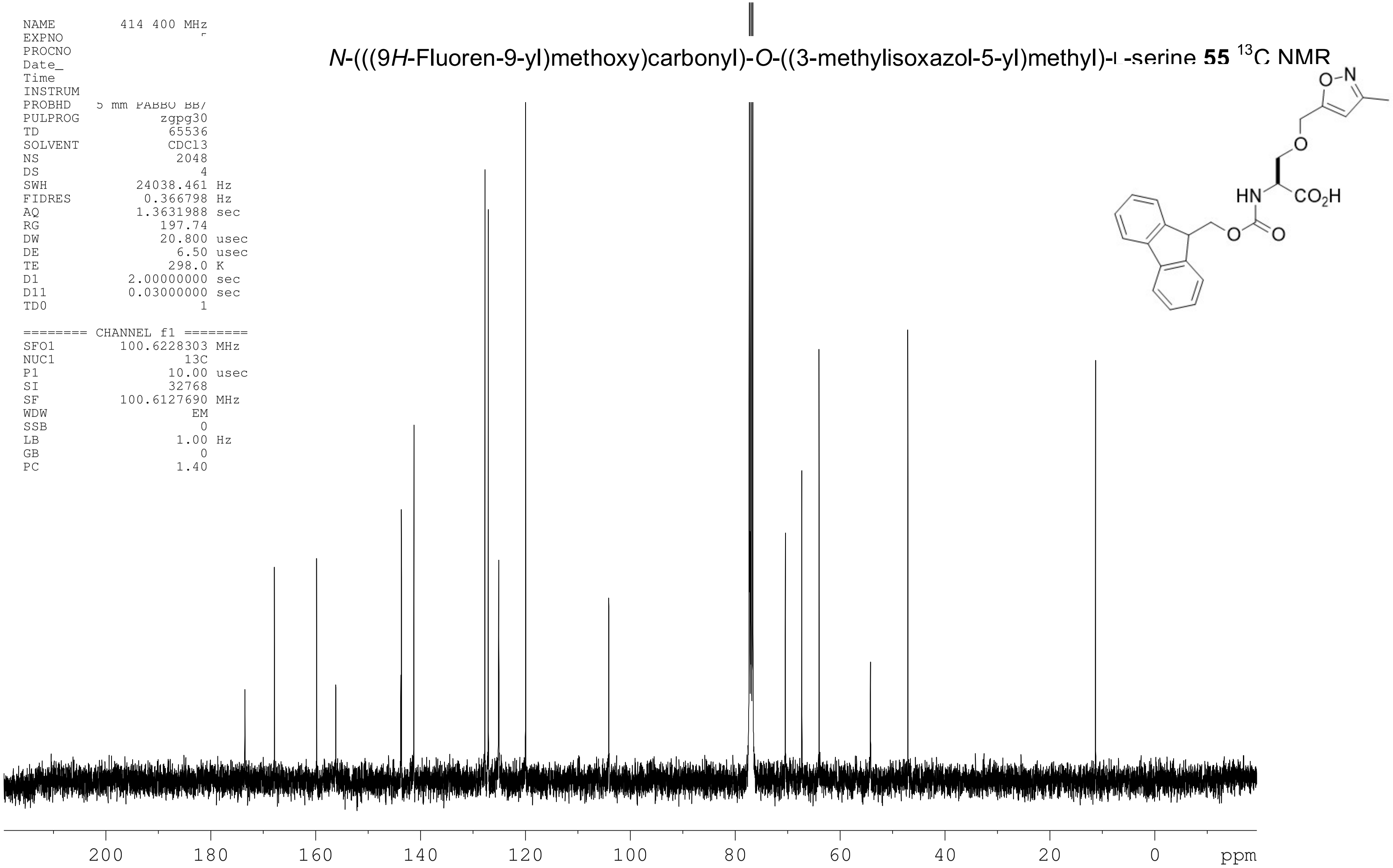

tert-Butyl *N*[2]-(*tert*-butoxycarbonyl)-*N*[5]-(3,5-dimethylisoxazol-4-yl)-L-glutaminate **58** ^{1}H NMR

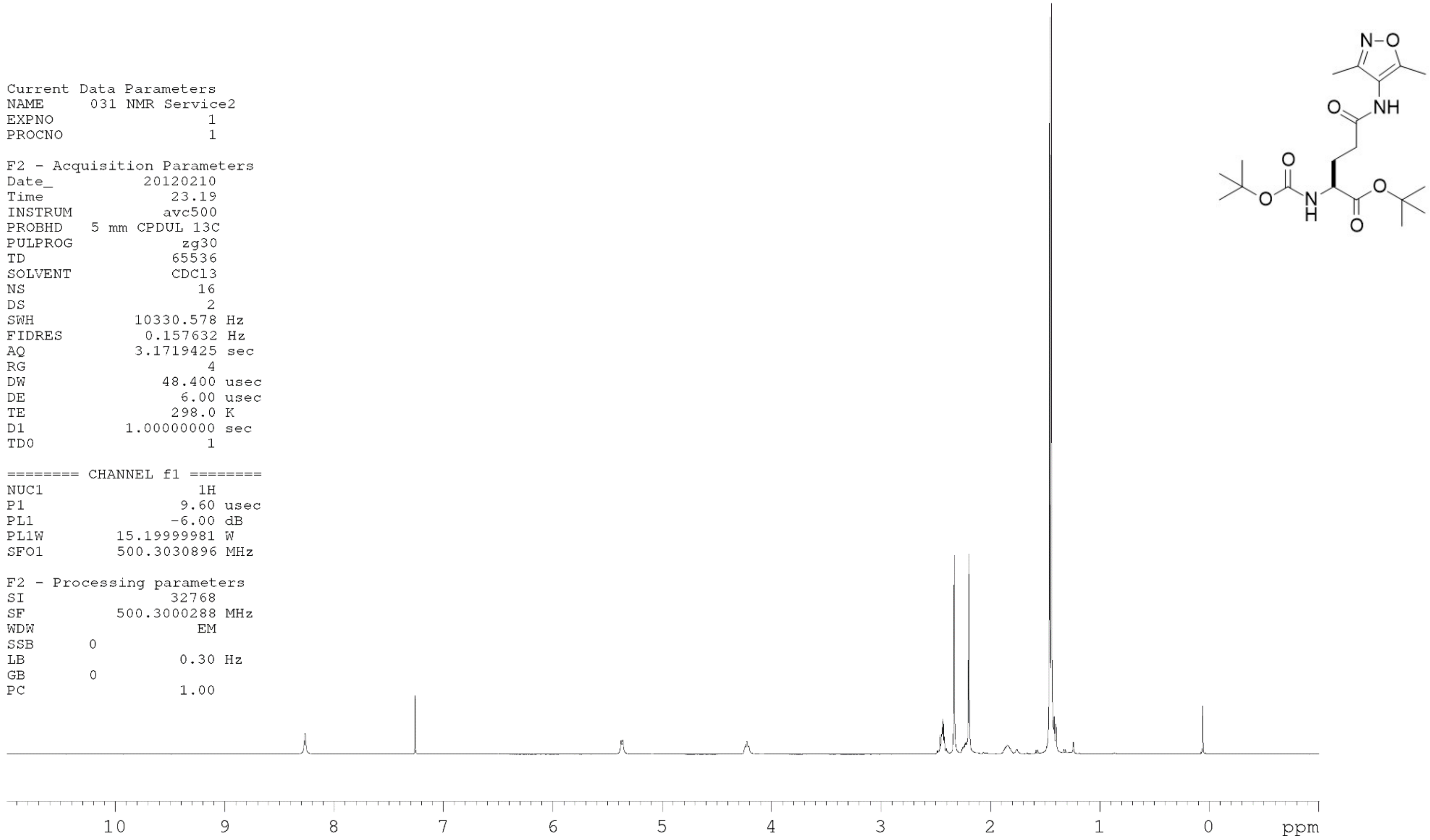

tert-Butyl N^2-(*tert*-butoxycarbonyl)-N^5-(3,5-dimethylisoxazol-4-yl)-L-glutaminate **58** ^{13}C NMR

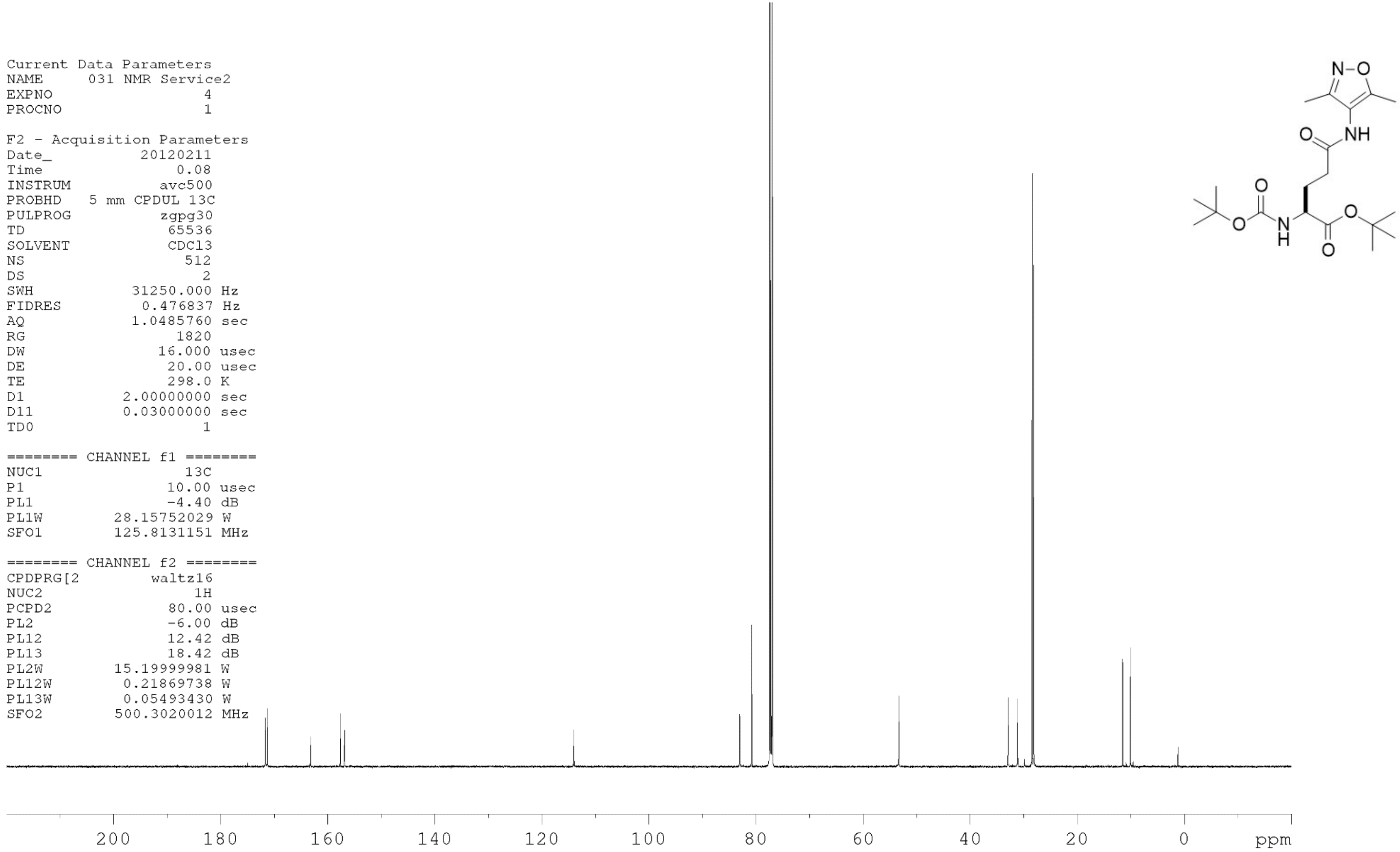

N^5-(3,5-Dimethylisoxazol-4-yl)-L-glutamine **10** ^{1}H NMR

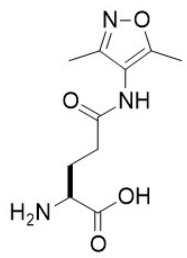

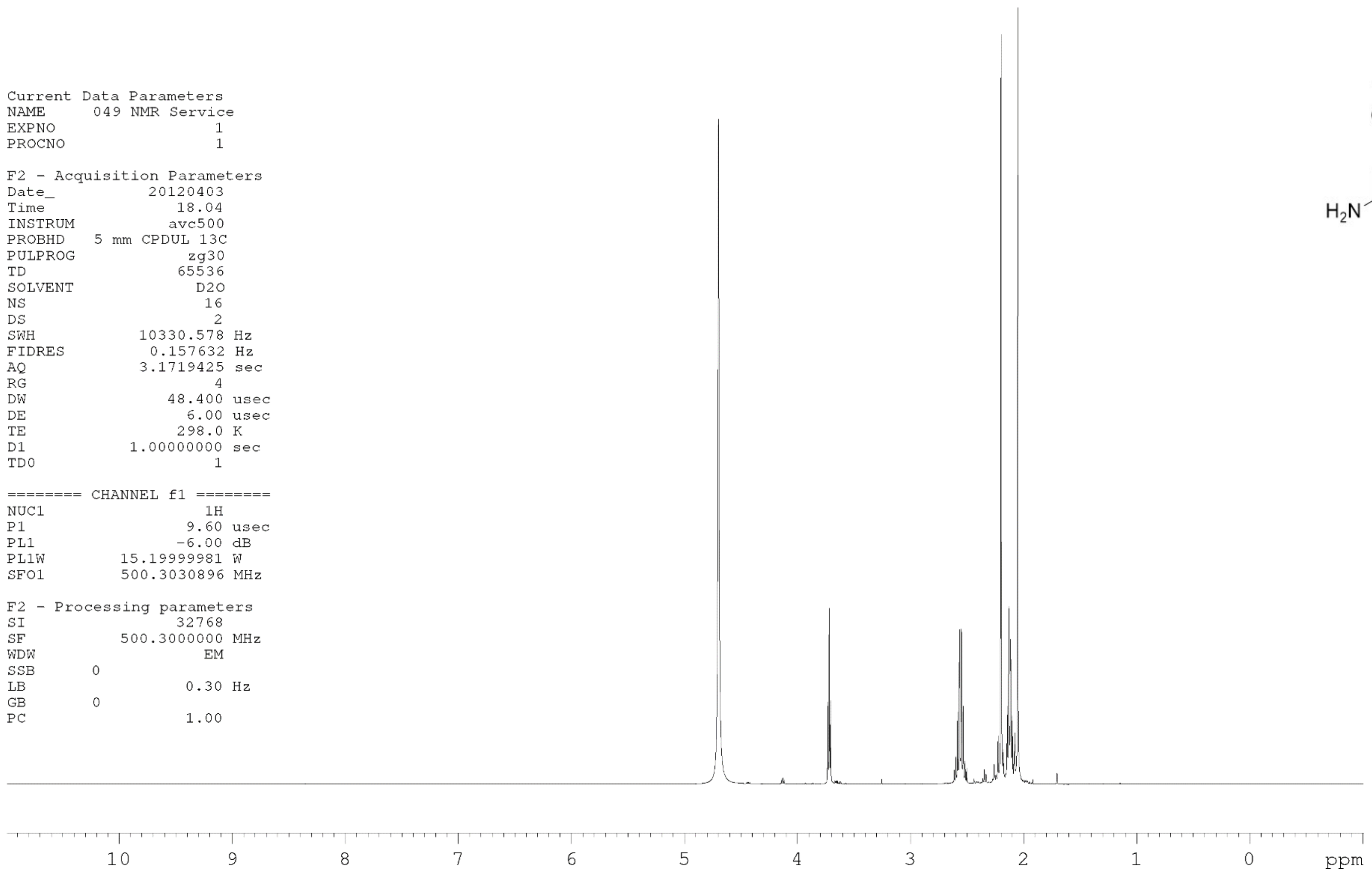

N^5-(3,5-Dimethylisoxazol-4-yl)-L-glutamine **10** ^{13}C NMR

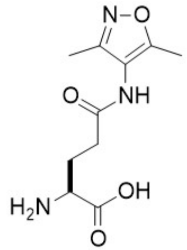

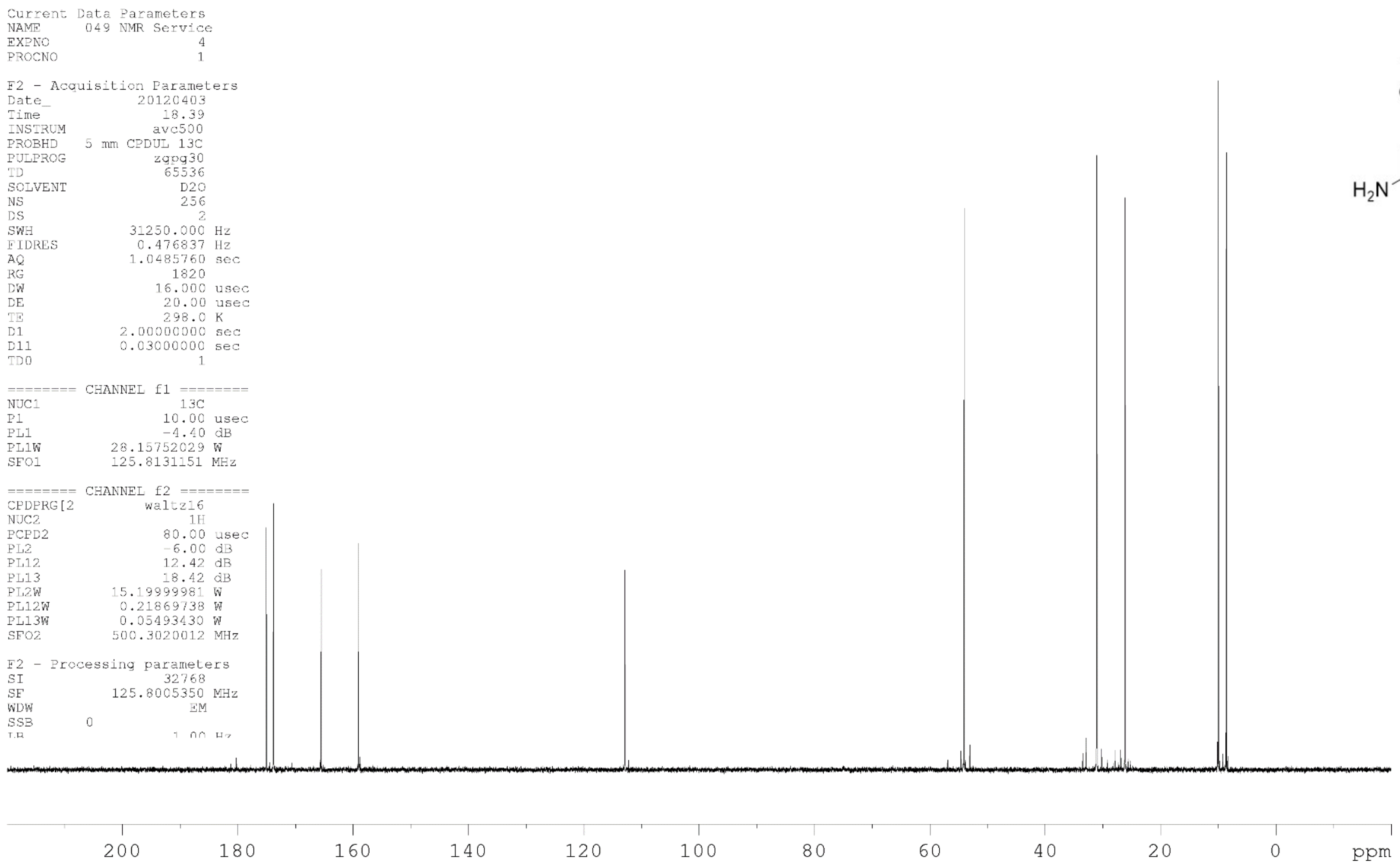

Benzyl N^2-(*tert*-butoxycarbonyl)-N^4-((3-methylisoxazol-5-yl)methyl)-L-asparaginate **64** ^{1}H NMR

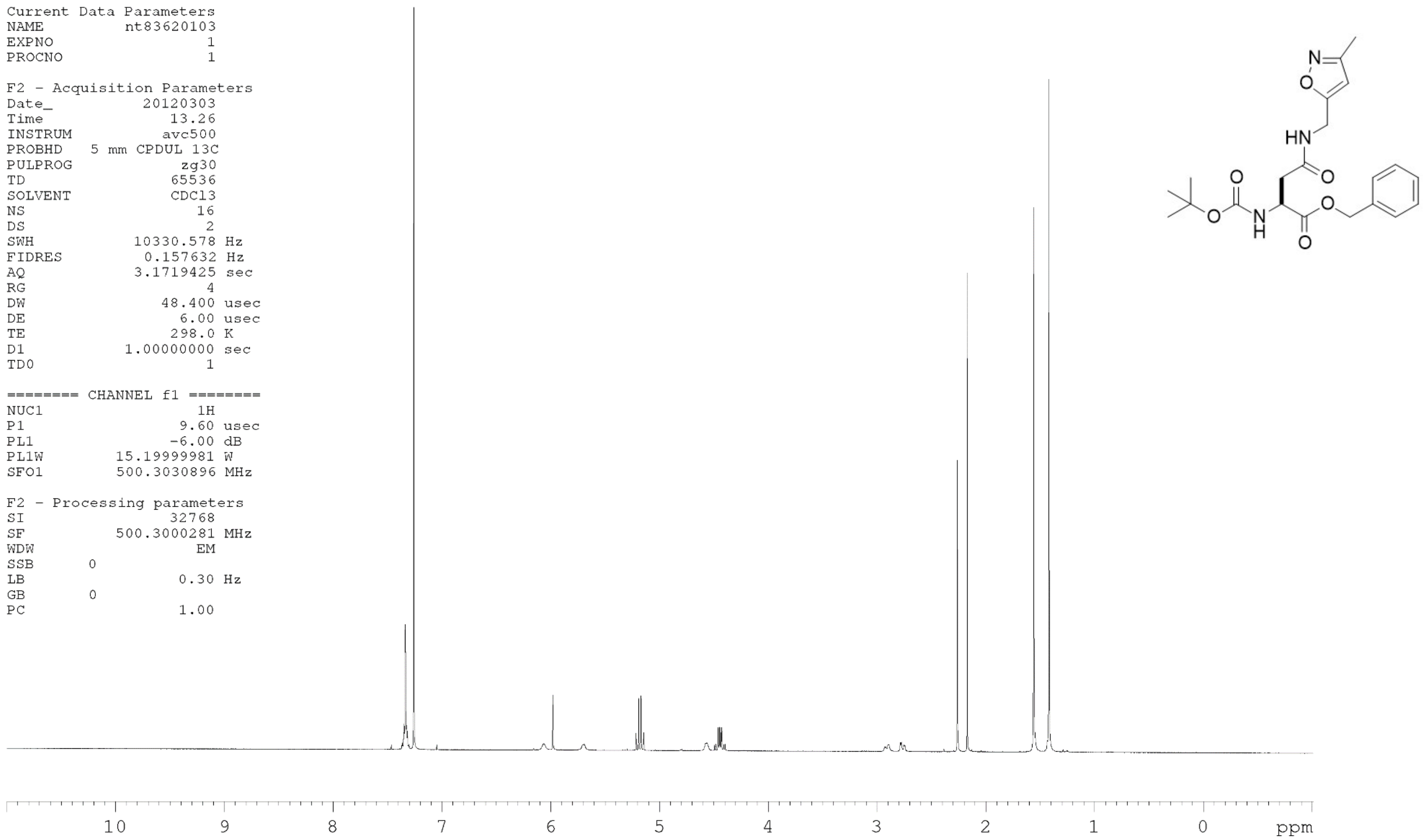

Benzyl N^2-(*tert*-butoxycarbonyl)-N^4-((3-methylisoxazol-5-yl)methyl)-L-asparaginate **64** ^{13}C NMR

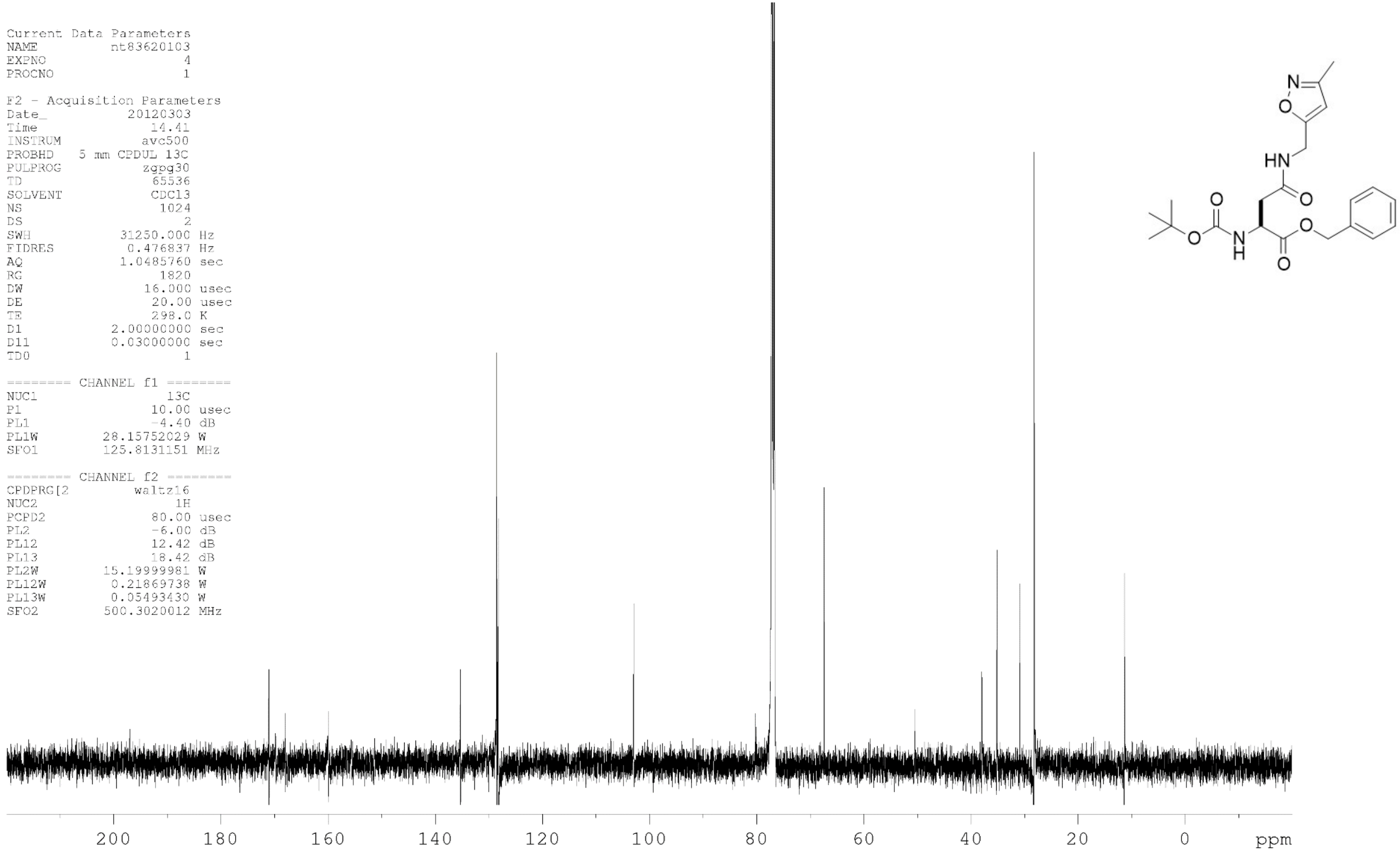

Benzyl N^2-(*tert*-butoxycarbonyl)-N^4-(3,5-dimethylisoxazol-4-yl)-L-asparaginate **65** ^{1}H NMR

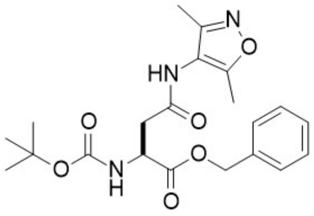

```
Current Data Parameters
NAME     024 NMR Service
EXPNO                  1
PROCNO                 1

F2 - Acquisition Parameters
Date_           20120211
Time                1.29
INSTRUM           avc500
PROBHD   5 mm CPDUL 13C
PULPROG             zg30
TD                 65536
SOLVENT            CDCl3
NS                    16
DS                     2
SWH            10330.578 Hz
FIDRES          0.157632 Hz
AQ             3.1719425 sec
RG                     4
DW                48.400 usec
DE                  6.00 usec
TE                 298.0 K
D1            1.00000000 sec
TD0                    1

======== CHANNEL f1 ========
NUC1                  1H
P1                  9.60 usec
PL1                -6.00 dB
PL1W         15.19999981 W
SFO1         500.3030896 MHz

F2 - Processing parameters
SI                 32768
SF           500.3000291 MHz
WDW                   EM
SSB      0
LB                  0.30 Hz
GB       0
PC                  1.00
```

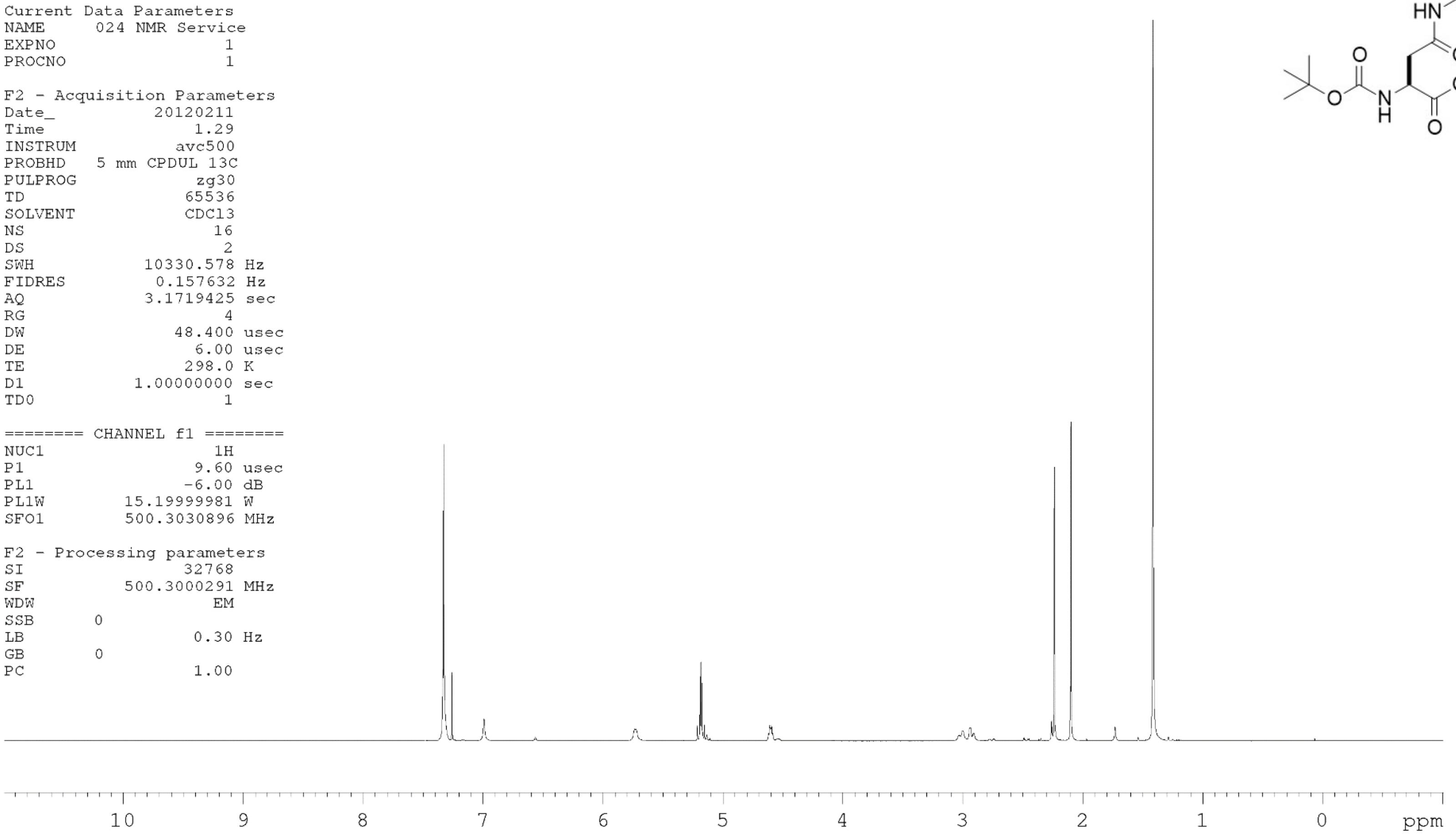

Benzyl N^2-(*tert*-butoxycarbonyl)-N^4-(3,5-dimethylisoxazol-4-yl)-L-asparaginate **65** ^{13}C NMR

```
Current Data Parameters
NAME      024 NMR Service
EXPNO                   4
PROCNO                  1

F2 - Acquisition Parameters
Date_          20120211
Time               2.44
INSTRUM          avc500
PROBHD   5 mm CPDUL 13C
PULPROG         zgpg30
TD               65536
SOLVENT          CDCl3
NS                1024
DS                   2
SWH          31250.000 Hz
FIDRES        0.476837 Hz
AQ           1.0485760 sec
RG                1820
DW              16.000 usec
DE               20.00 usec
TE               298.0 K
D1          2.00000000 sec
D11         0.03000000 sec
TD0                  1

======== CHANNEL f1 ========
NUC1               13C
P1               10.00 usec
PL1              -4.40 dB
PL1W       28.15752029 W
SFO1       125.8131151 MHz

======== CHANNEL f2 ========
CPDPRG[2        waltz16
NUC2                 1H
PCPD2            80.00 usec
PL2              -6.00 dB
PL12             12.42 dB
PL13             18.42 dB
PL2W       15.19999981 W
PL12W       0.21869738 W
PL13W       0.05493430 W
SFO2       500.3020012 MHz
```

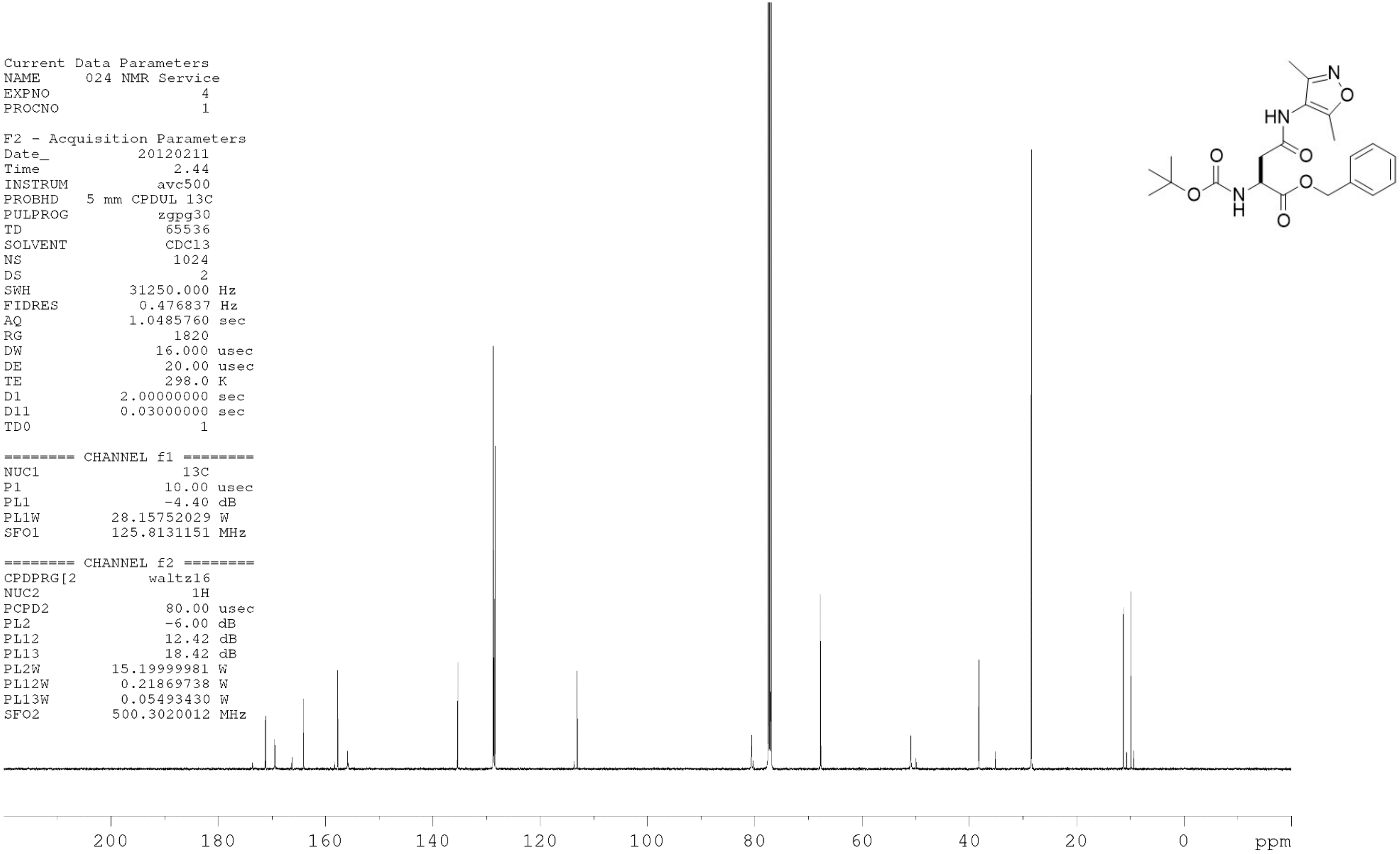

Benzyl N^2-(*tert*-butoxycarbonyl)-N^4-(5-methylisoxazol-3-yl)-L-asparaginate **66** ^{1}H NMR

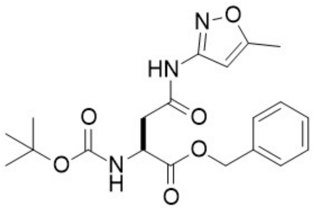

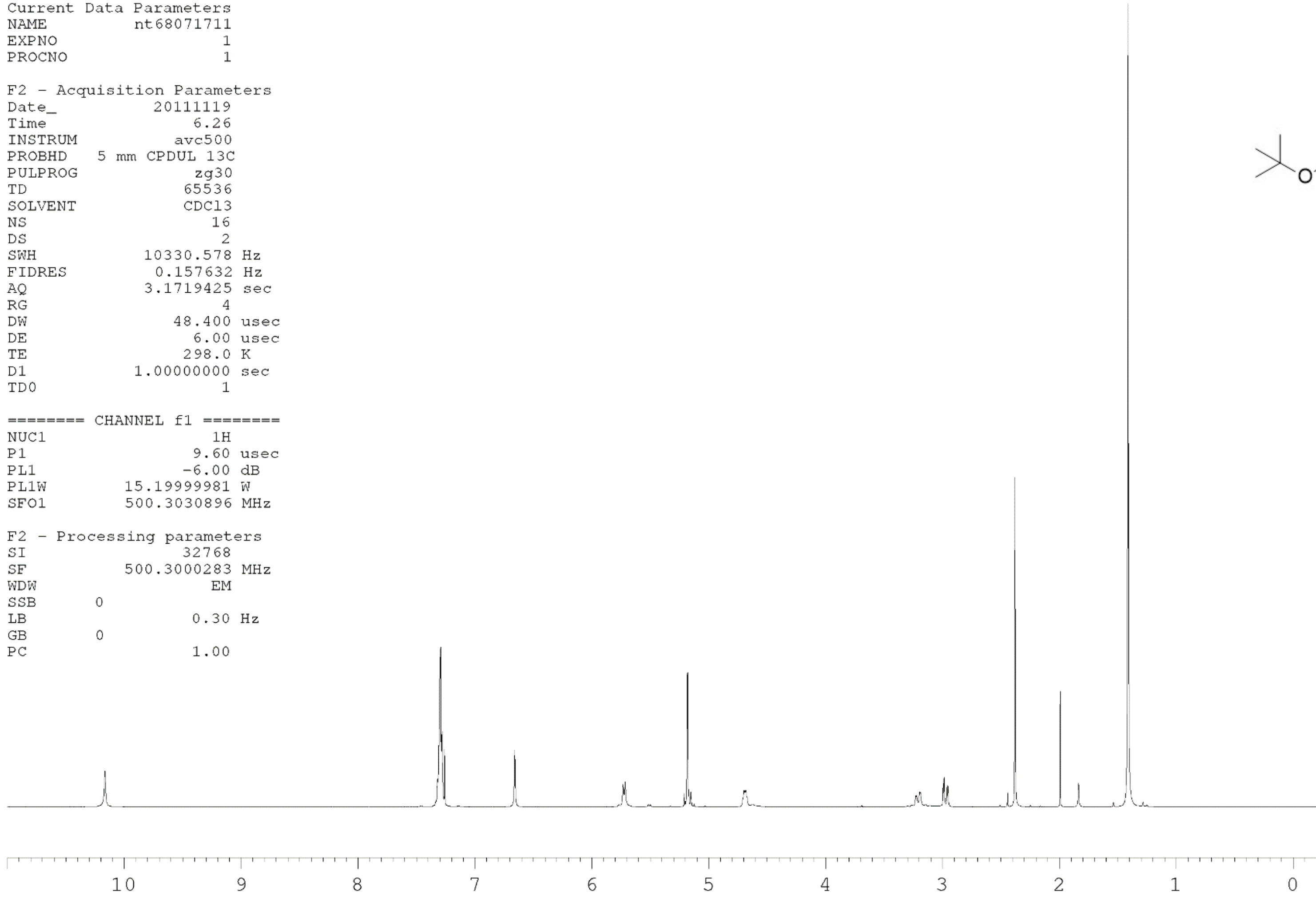


```
Current Data Parameters
NAME          nt68071711
EXPNO                  1
PROCNO                 1

F2 - Acquisition Parameters
Date_           20111119
Time                6.26
INSTRUM           avc500
PROBHD   5 mm CPDUL 13C
PULPROG             zg30
TD                 65536
SOLVENT            CDCl3
NS                    16
DS                     2
SWH          10330.578 Hz
FIDRES        0.157632 Hz
AQ           3.1719425 sec
RG                     4
DW              48.400 usec
DE                6.00 usec
TE               298.0 K
D1          1.00000000 sec
TD0                    1

======== CHANNEL f1 ========
NUC1                  1H
P1                  9.60 usec
PL1                -6.00 dB
PL1W         15.19999981 W
SFO1        500.3030896 MHz

F2 - Processing parameters
SI                 32768
SF          500.3000283 MHz
WDW                   EM
SSB      0
LB                  0.30 Hz
GB       0
PC                  1.00
```

Benzyl N^2-(*tert*-butoxycarbonyl)-N^4-(5-methylisoxazol-3-yl)-L-asparaginate **66** ^{13}C NMR

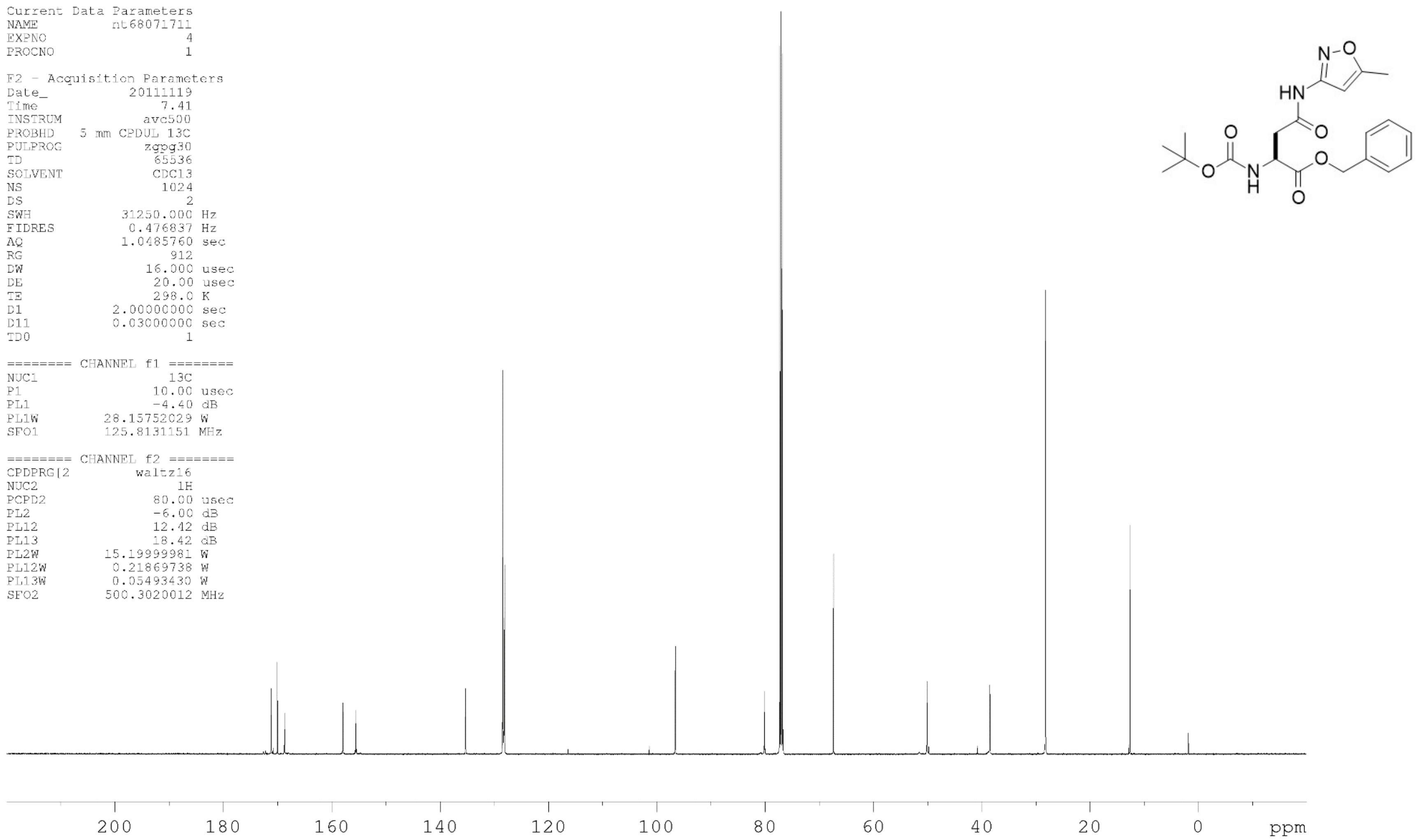

Benzyl N^2-(*tert*-butoxycarbonyl)-N^4-(3-methylisoxazol-5-yl)-L-asparaginate **67** ^{1}H NMR

```
Current Data Parameters
NAME          nt68061711
EXPNO                  1
PROCNO                 1

F2 - Acquisition Parameters
Date_           20111119
Time                4.34
INSTRUM           avc500
PROBHD   5 mm CPDUL 13C
PULPROG             zg30
TD                 65536
SOLVENT            CDCl3
NS                    16
DS                     2
SWH            10330.578 Hz
FIDRES          0.157632 Hz
AQ             3.1719425 sec
RG                     4
DW                48.400 usec
DE                  6.00 usec
TE                 298.0 K
D1            1.00000000 sec
TD0                    1

======== CHANNEL f1 ========
NUC1                  1H
P1                  9.60 usec
PL1                -6.00 dB
PL1W         15.19999981 W
SFO1         500.3030896 MHz

F2 - Processing parameters
SI                 32768
SF           500.3000286 MHz
WDW                   EM
SSB      0
LB                  0.30 Hz
GB       0
PC                  1.00
```

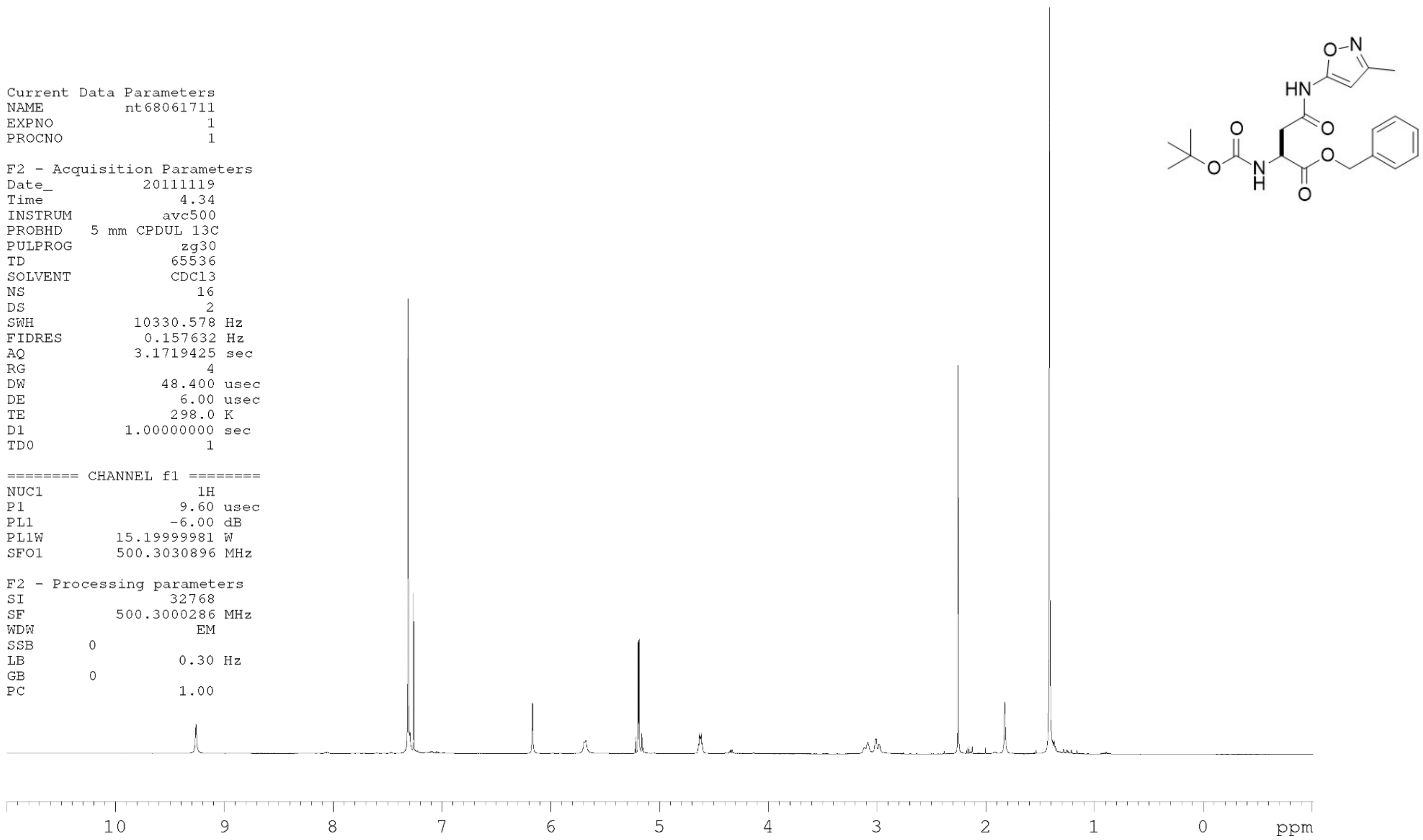

Benzyl N^2-(*tert*-butoxycarbonyl)-N^4-(3-methylisoxazol-5-yl)-L-asparaginate **67** ^{13}C NMR

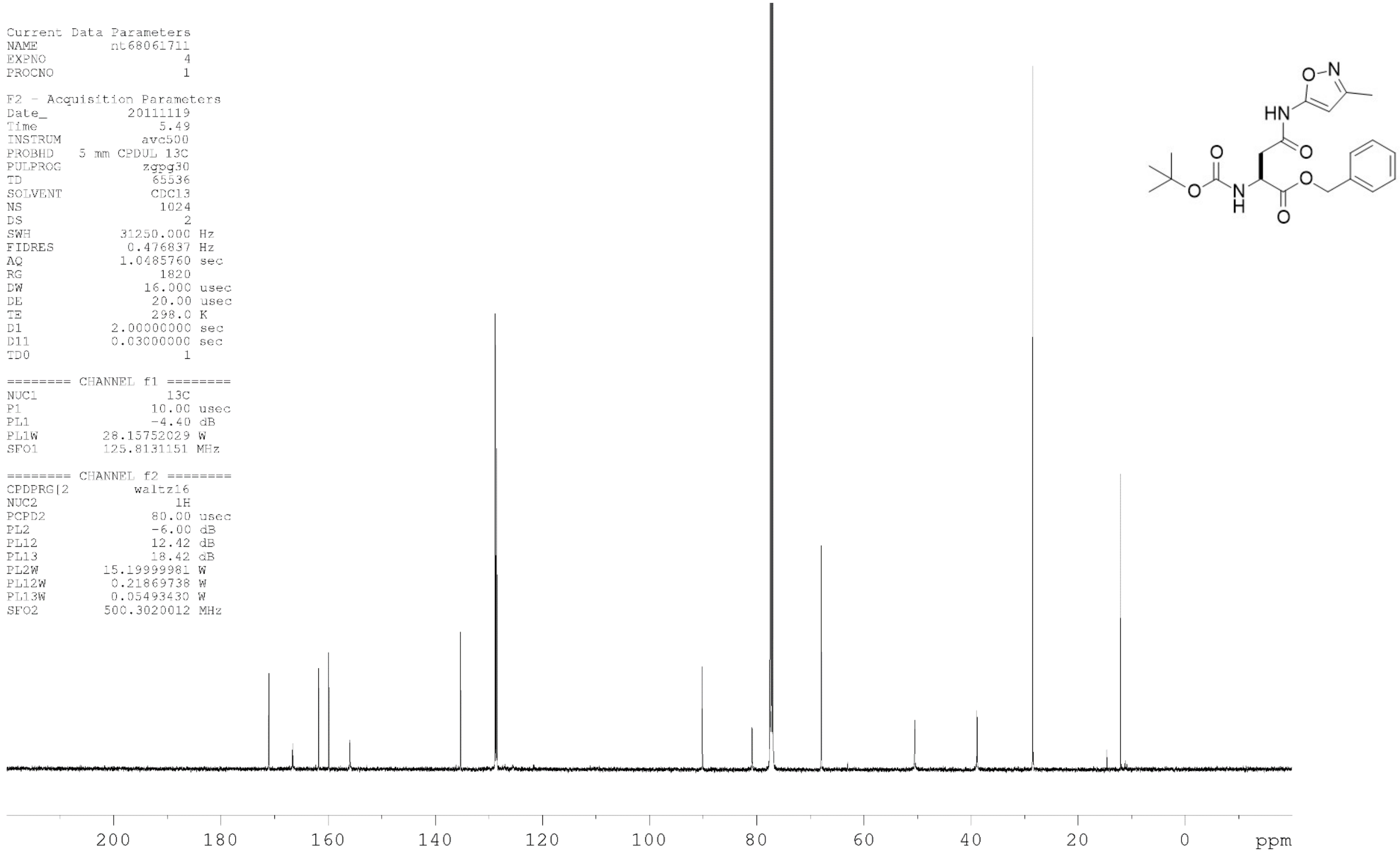

N^4-((3-Methylisoxazol-5-yl)methyl)-L-asparagine **11** ^{1}H NMR

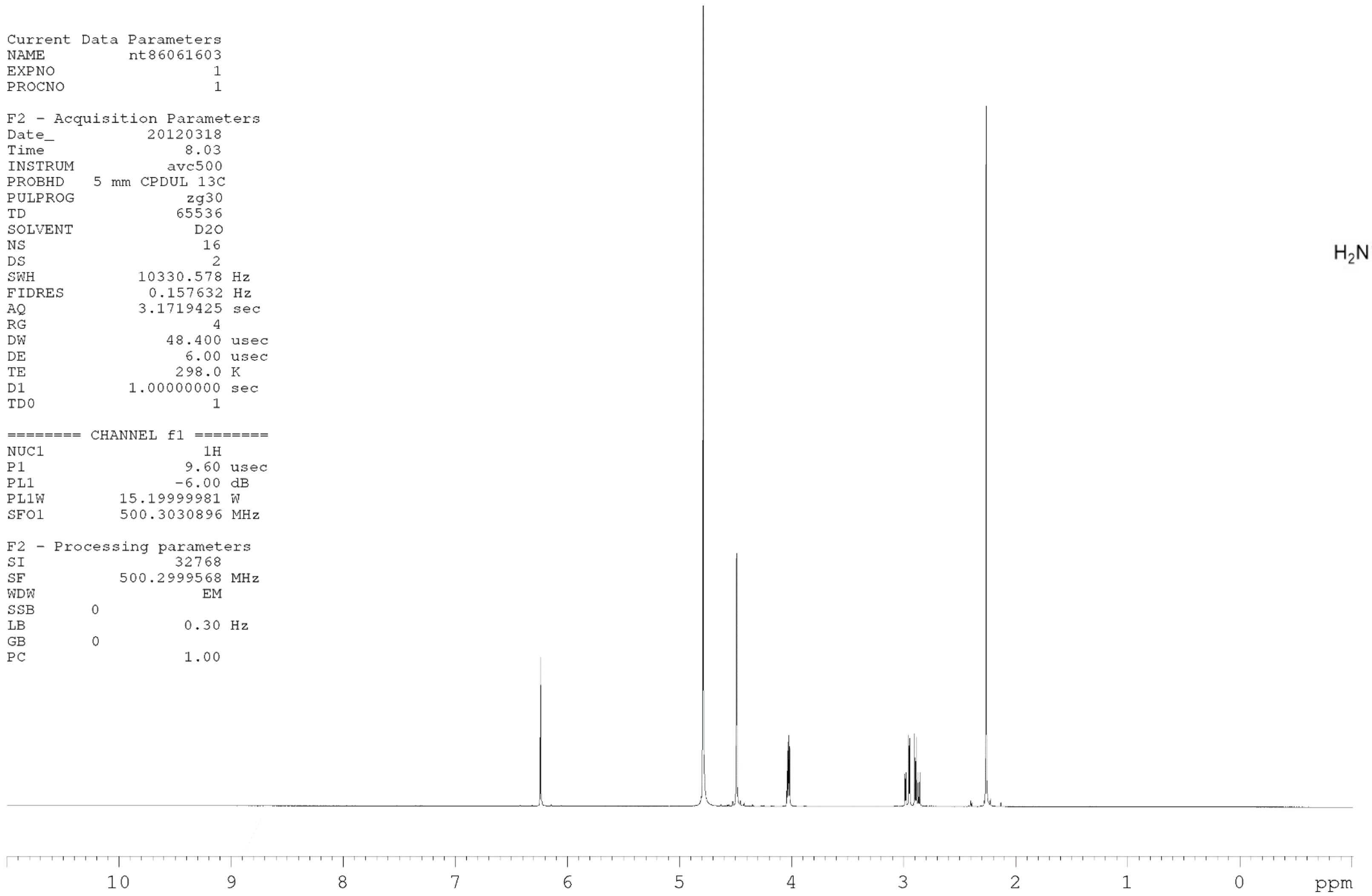

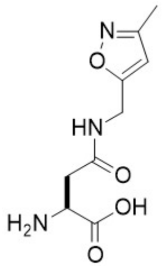

N^4-((3-Methylisoxazol-5-yl)methyl)-L-asparagine **11** ^{13}C NMR

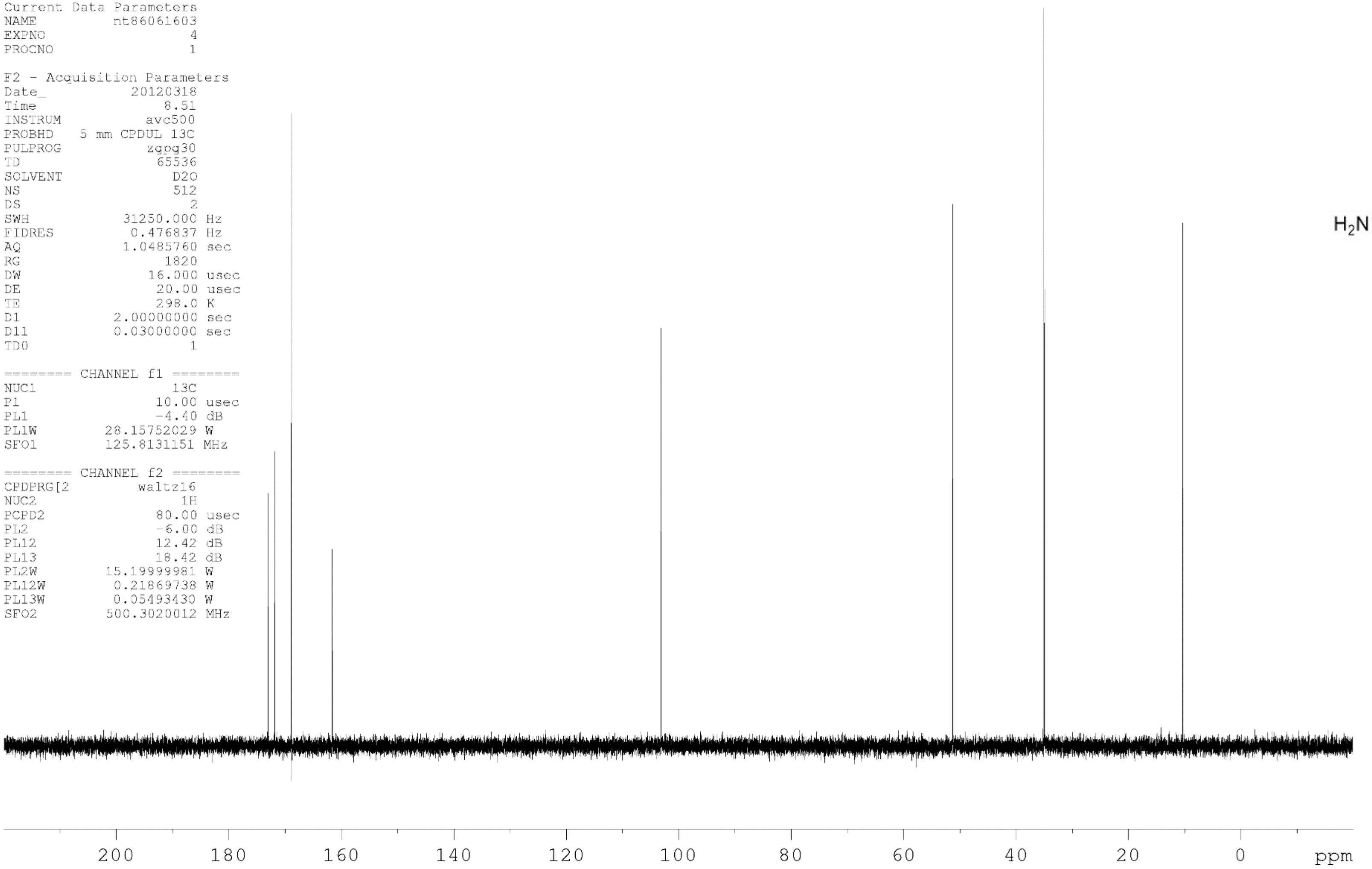

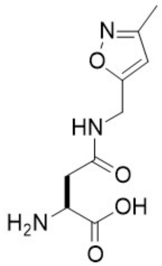

N^4-(3,5-Dimethylisoxazol-4-yl)-L-asparagine **12** ^{1}H NMR

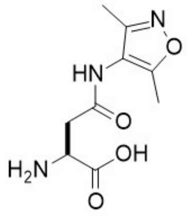

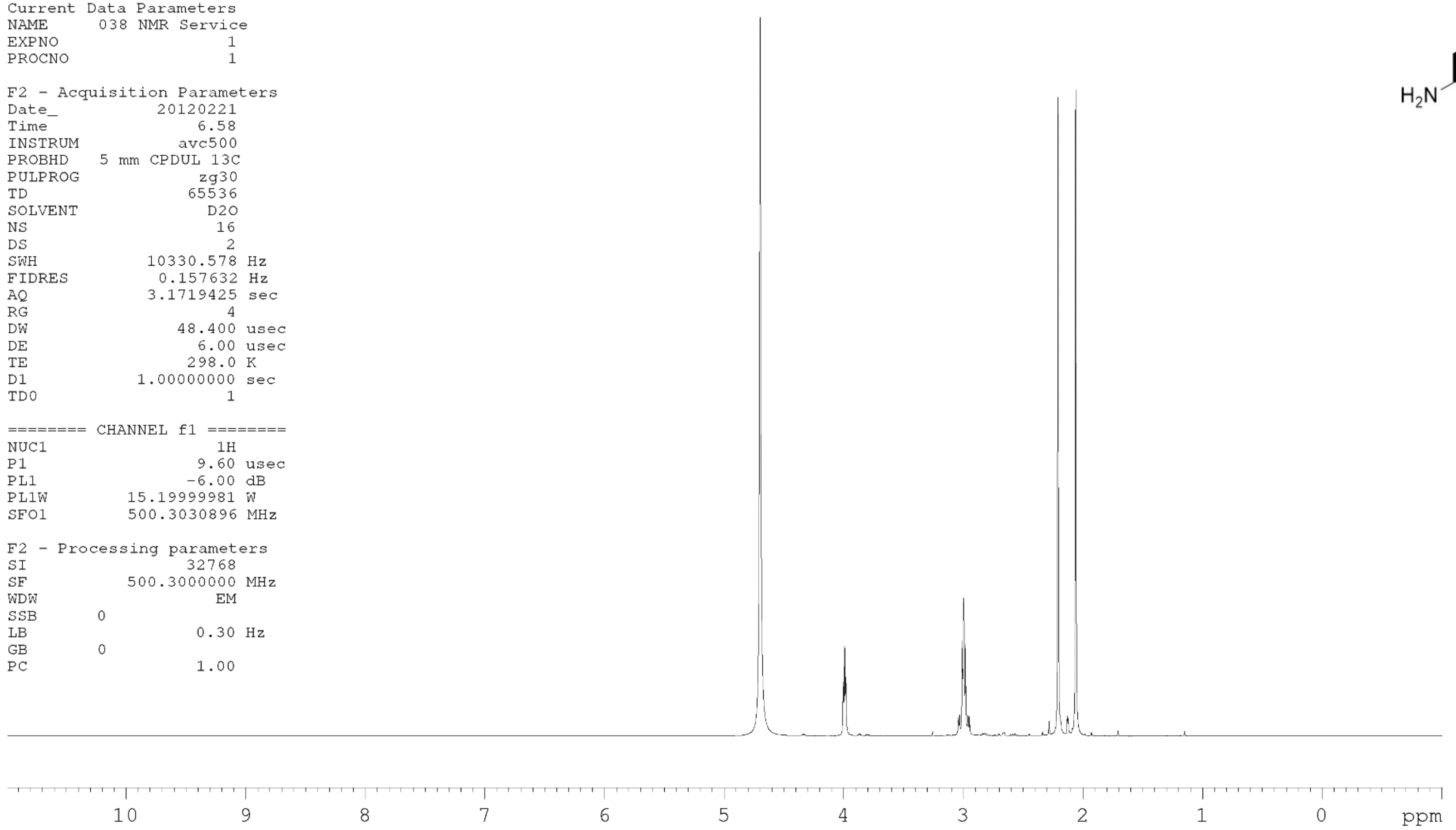

N^4-(3,5-Dimethylisoxazol-4-yl)-L-asparagine **12** ^{13}C NMR

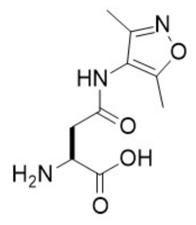

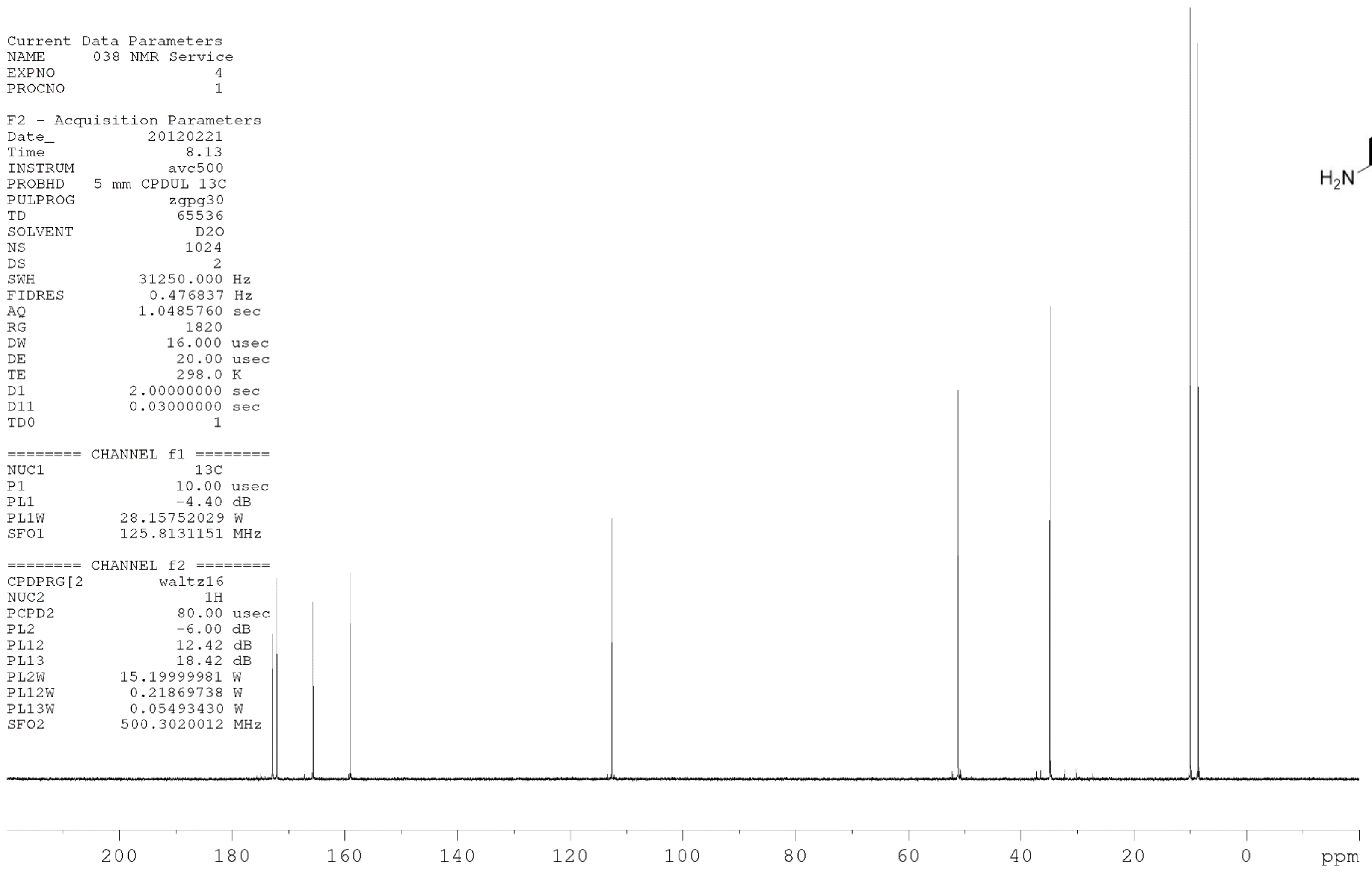

N^4-(5-Methylisoxazol-3-yl)-L-asparagine **13** ^{1}H NMR

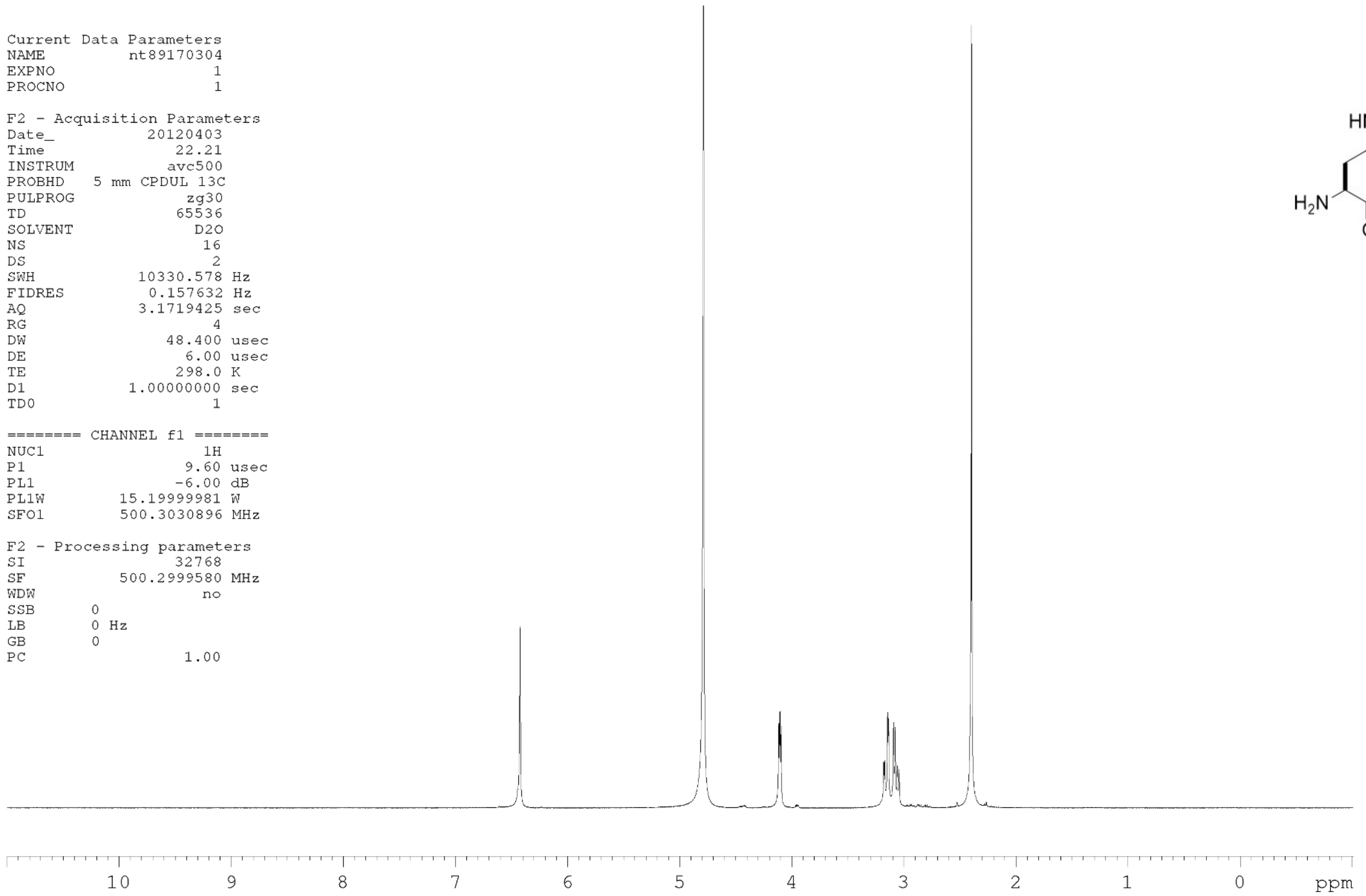

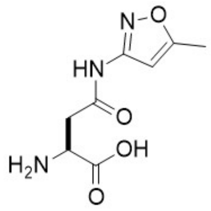

N^4-(5-Methylisoxazol-3-yl)-L-asparagine **13** ^{13}C NMR

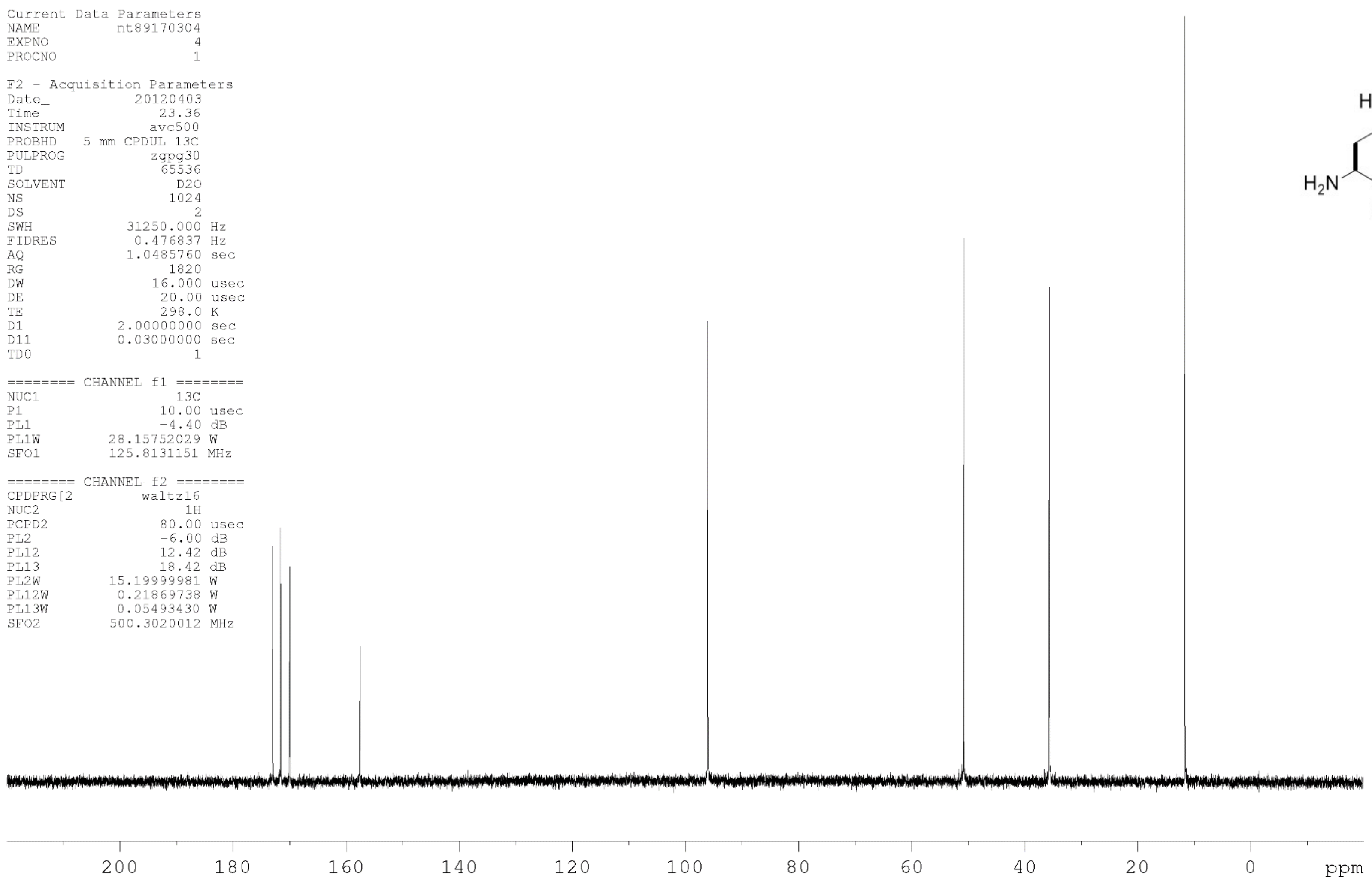

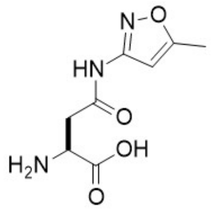

N^4-(3-Methylisoxazol-5-yl)-L-asparagine **14** ^{1}H NMR

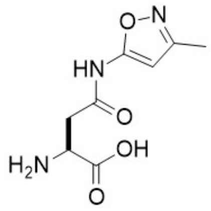

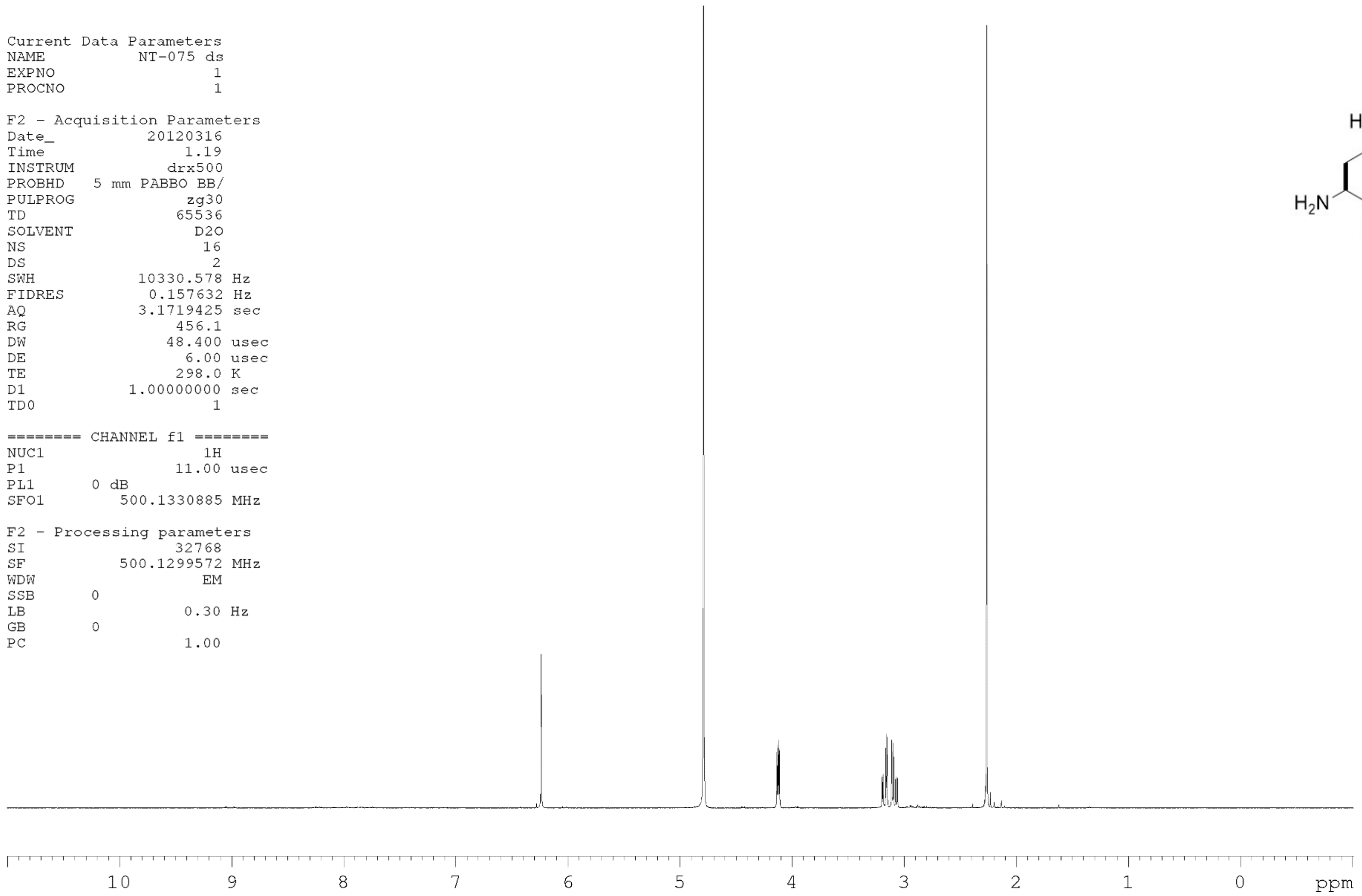

N^4-(3-Methylisoxazol-5-yl)-L-asparagine **14** ^{13}C NMR

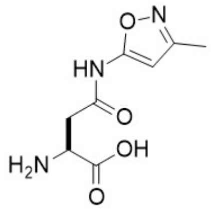


```
Current Data Parameters
NAME          NT-075 ds
EXPNO                 5
PROCNO                1

F2 - Acquisition Parameters
Date_          20120316
Time               3.05
INSTRUM          drx500
PROBHD   5 mm PABBO BB/
PULPROG         zgpg30
TD                65536
SOLVENT             D2O
NS                 1024
DS                    4
SWH           30030.029 Hz
FIDRES         0.458222 Hz
AQ            1.0911744 sec
RG               3649.1
DW               16.650 usec
DE                 6.00 usec
TE                298.0 K
D1           2.00000000 sec
d11          0.03000000 sec
DELTA        1.89999998 sec
TD0                   1

======== CHANNEL f1 ========
NUC1                13C
P1                 6.80 usec
PL1                5.00 dB
SFO1        125.7703643 MHz

======== CHANNEL f2 ========
CPDPRG[2        waltz16
NUC2                 1H
PCPD2            100.00 usec
PL2       0 dB
PL12              19.00 dB
PL13              23.00 dB
SFO2        500.1320005 MHz
```

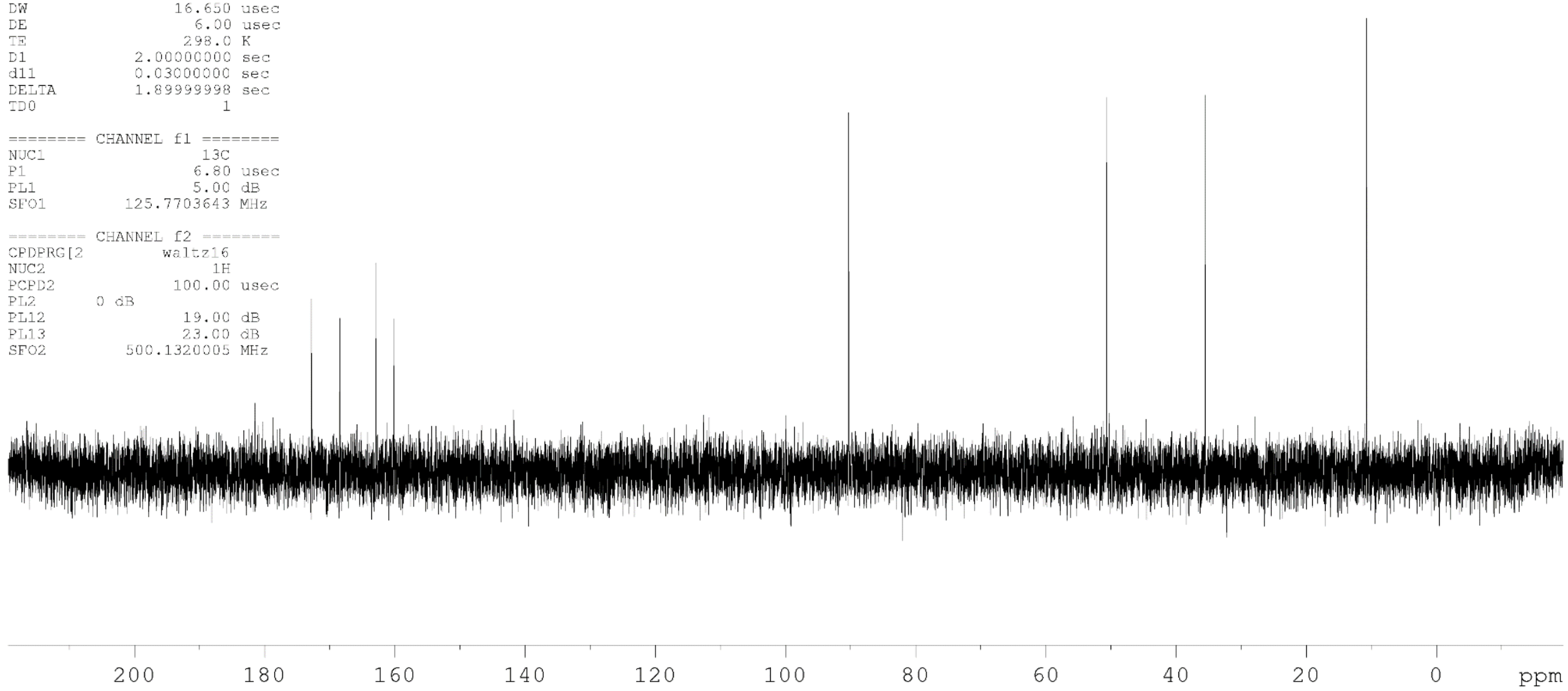

(*S*)-2-((*tert*-Butoxycarbonyl)amino)-3-(3-methylisoxazole-5-carboxamido)propanoic acid **70** ^{1}H NMR

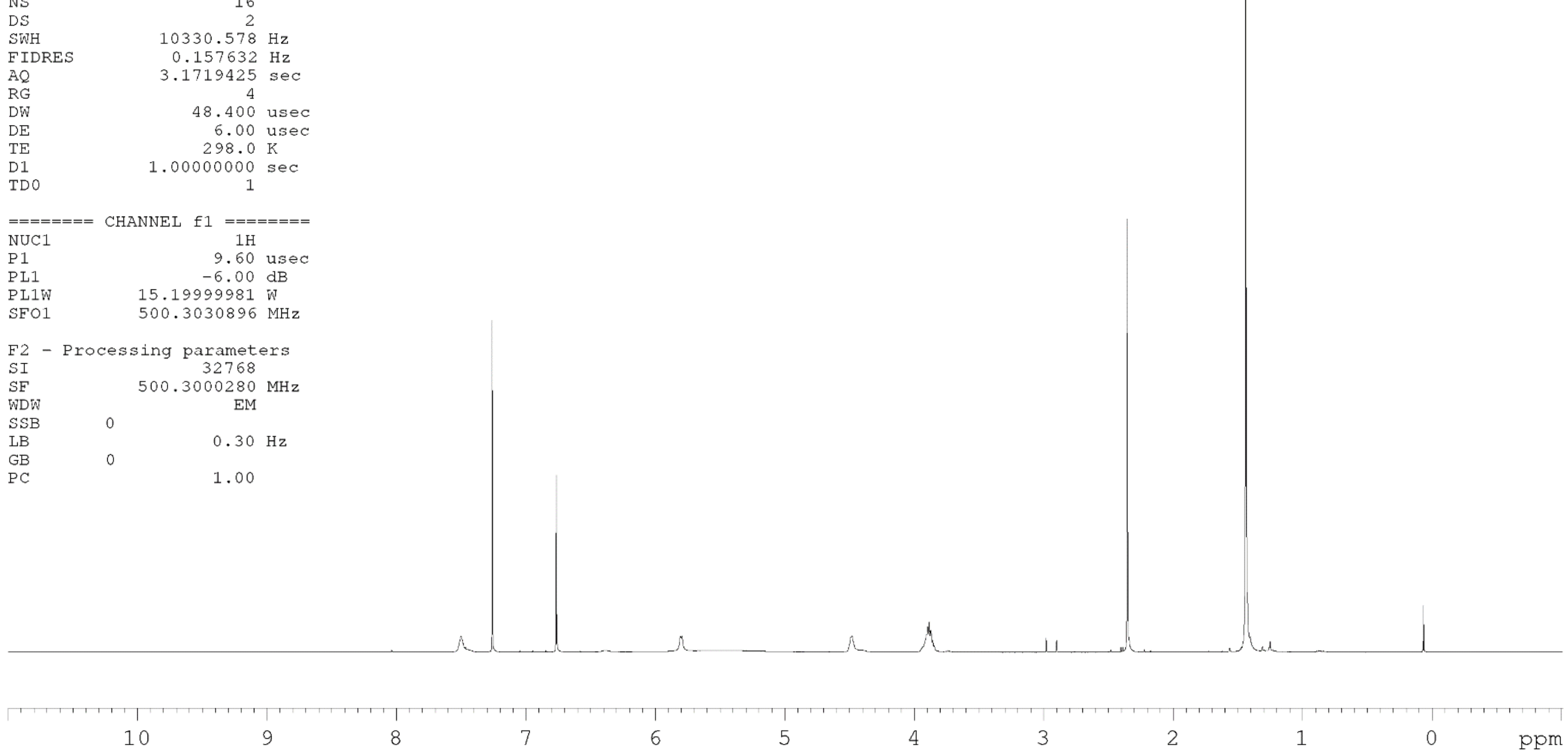

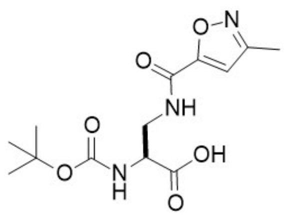

(*S*)-2-((*tert*-Butoxycarbonyl)amino)-3-(3-methylisoxazole-5-carboxamido)propanoic acid **70** ^{13}C NMR

```
Current Data Parameters
NAME          nt87942603
EXPNO                  1
PROCNO                 1

F2 - Acquisition Parameters
Date_           20120327
Time                6.16
INSTRUM           avc500
PROBHD   5 mm CPDUL 13C
PULPROG             zg30
TD                 65536
SOLVENT            CDCl3
NS                    16
DS                     2
SWH          10330.578 Hz
FIDRES        0.157632 Hz
AQ           3.1719425 sec
RG                     4
DW              48.400 usec
DE                6.00 usec
TE               298.0 K
D1         1.00000000 sec
TD0                    1

======== CHANNEL f1 ========
NUC1                  1H
P1                  9.60 usec
PL1                -6.00 dB
PL1W         15.19999981 W
SFO1        500.3030896 MHz

F2 - Processing parameters
SI                 32768
SF          500.3000280 MHz
WDW                   EM
SSB      0
LB                  0.30 Hz
GB       0
PC                  1.00
```

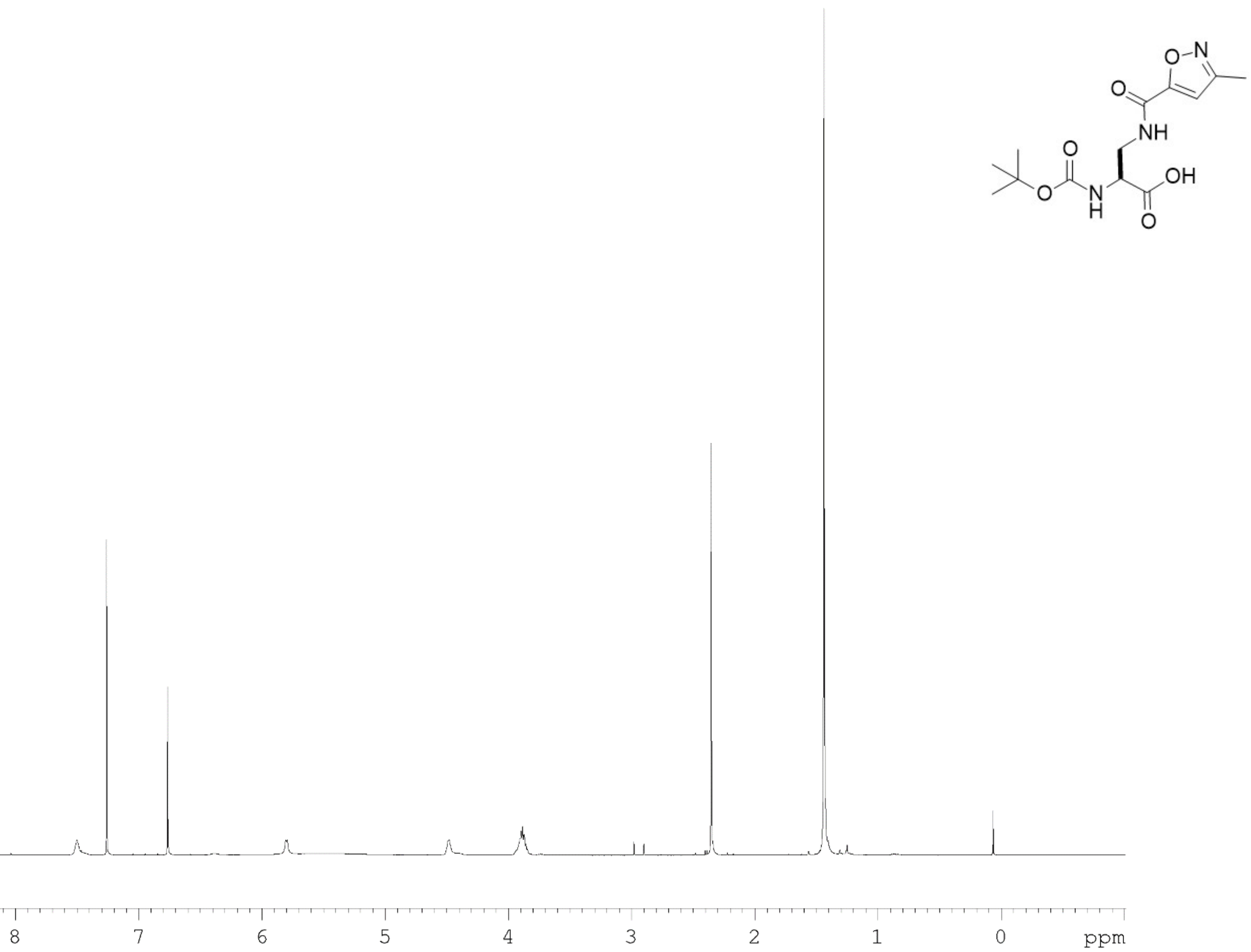

(*S*)-2-Amino-3-(3-methylisoxazole-5-carboxamido)propanoic acid **5** ^{1}H NMR

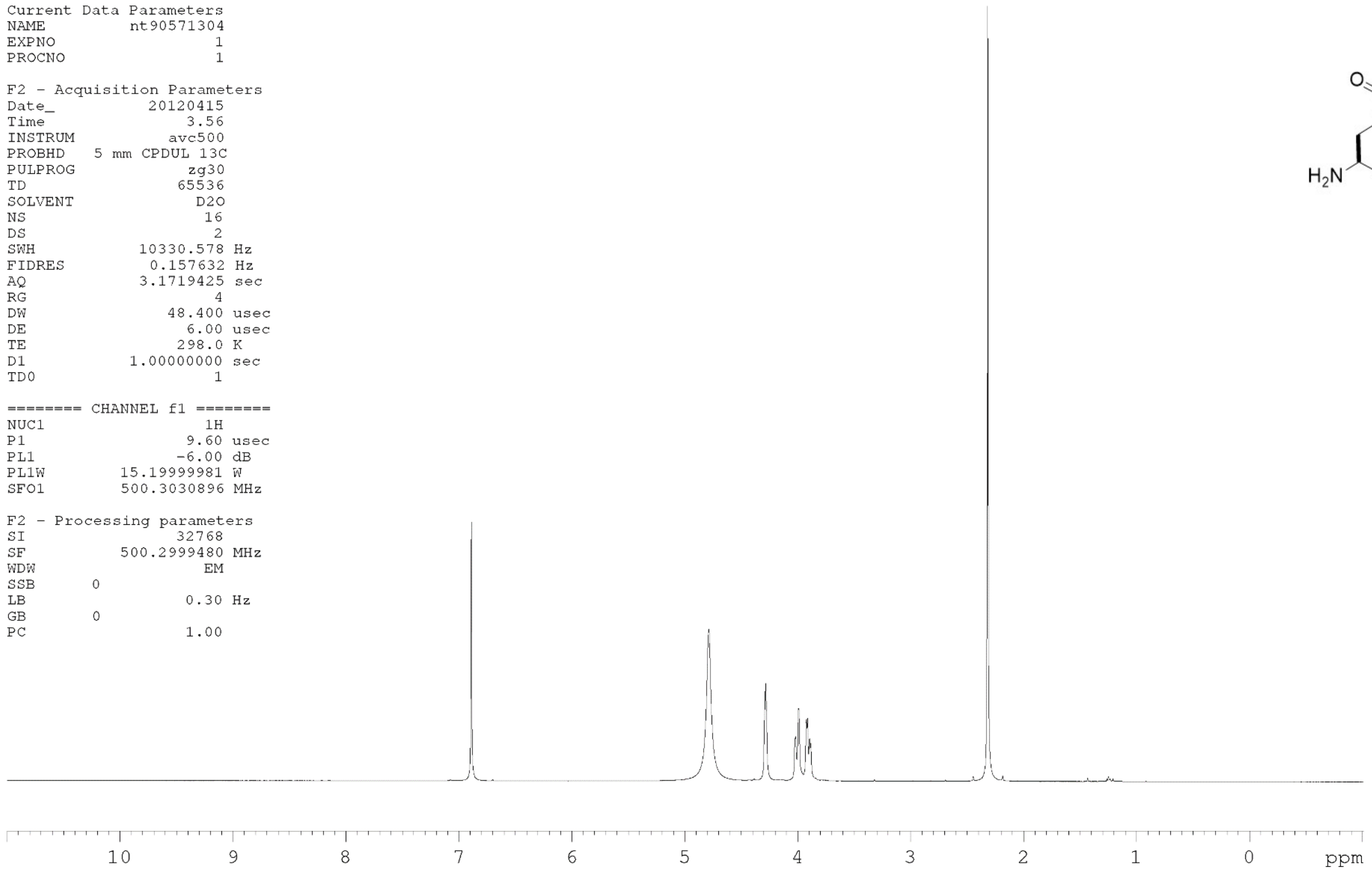

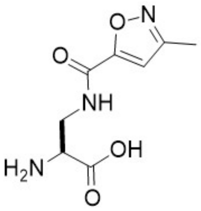

(*S*)-2-Amino-3-(3-methylisoxazole-5-carboxamido)propanoic acid **5** ^{13}C NMR

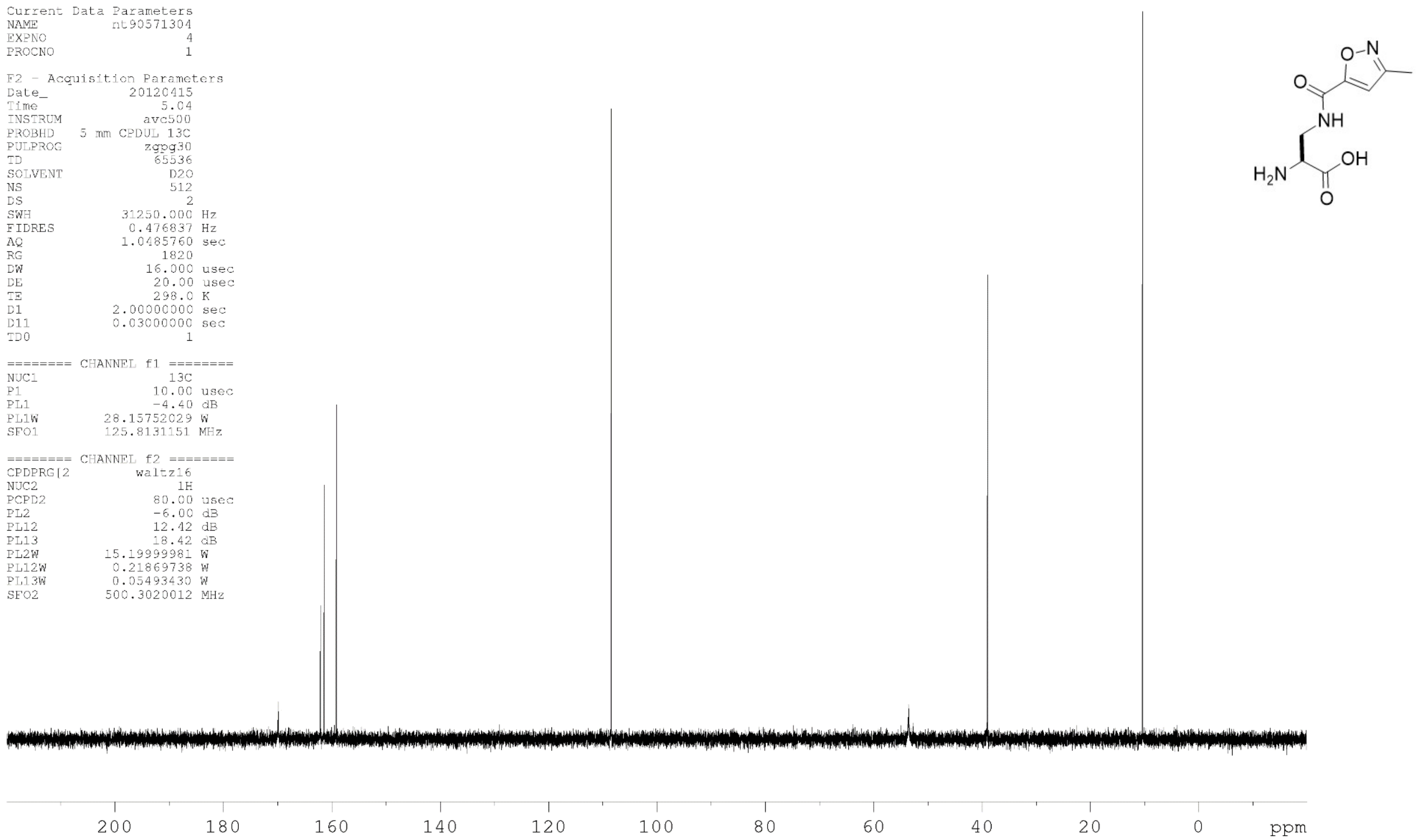

(*S*)-2-((((9*H*-Fluoren-9-yl)methoxy)carbonyl)amino)-3-(3-methylisoxazole-5-carboxamido)propanoic acid **71** ^{1}H NMR

```
NAME                263
EXPNO                 1
PROCNO                1
Date_          20121030
Time               1.19
INSTRUM          avc500
PROBHD   5 mm CPTXI 1H-
PULPROG            zg30
TD                65536
SOLVENT         Acetone
NS                   16
DS                    2
SWH           10330.578 Hz
FIDRES         0.157632 Hz
AQ            3.1719923 sec
RG                 20.2
DW               48.400 usec
DE                 6.00 usec
TE                298.0 K
D1           1.00000000 sec
TD0                   1

======== CHANNEL f1 ========
NUC1                 1H
P1                11.00 usec
PL1                0.00 dB
PL1W         3.81806731 W
SFO1        500.3030896 MHz
SI                32768
SF          500.3000000 MHz
WDW                  EM
SSB                   0
LB                 0.30 Hz
GB                    0
PC                 1.00
```

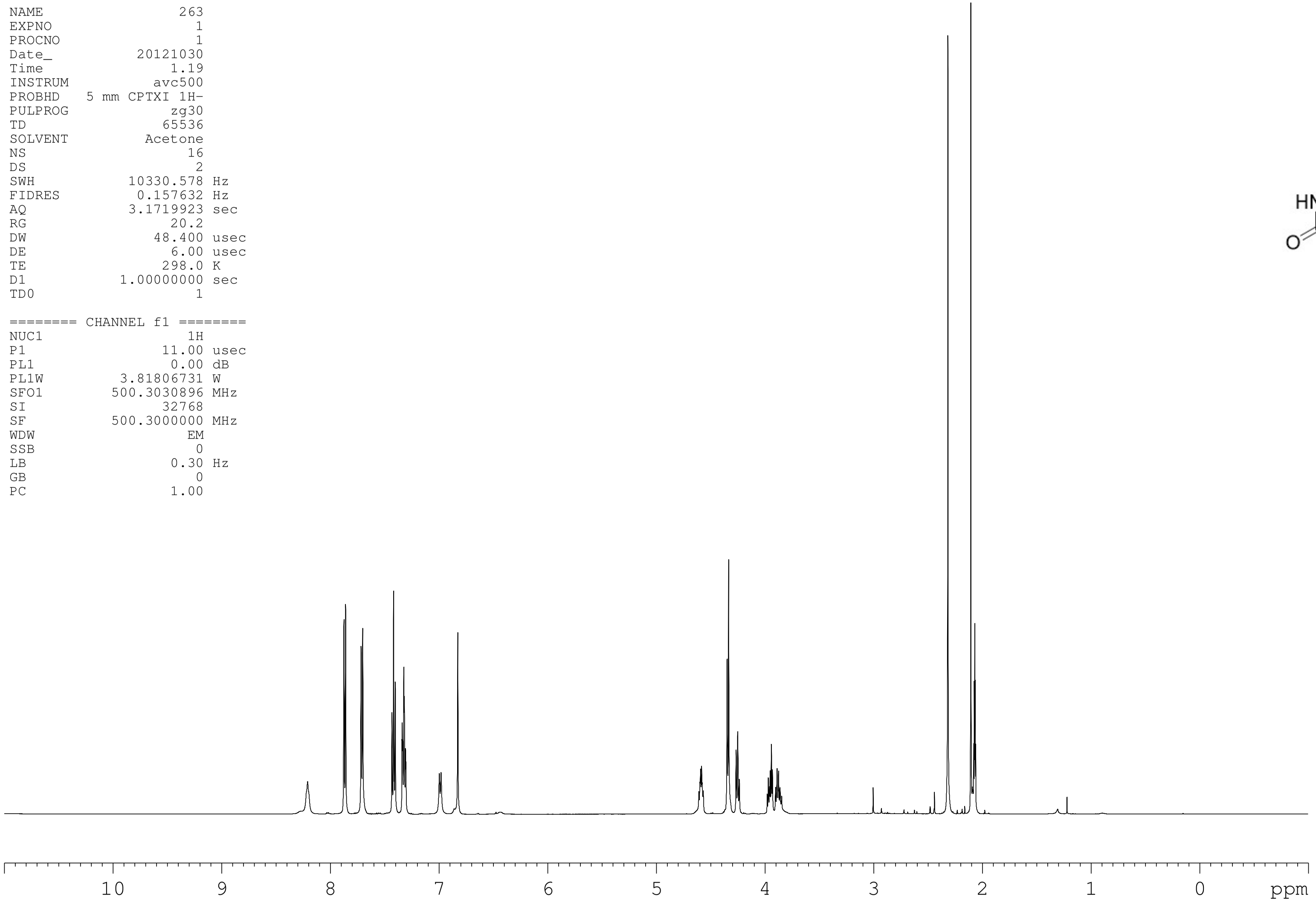

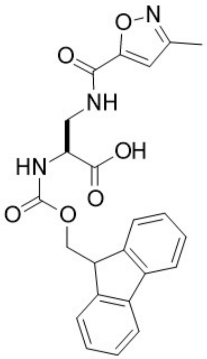

(*S*)-2-((((9*H*-Fluoren-9-yl)methoxy)carbonyl)amino)-3-(3-methylisoxazole-5-carboxamido)propanoic acid **71** ^{13}C NMR

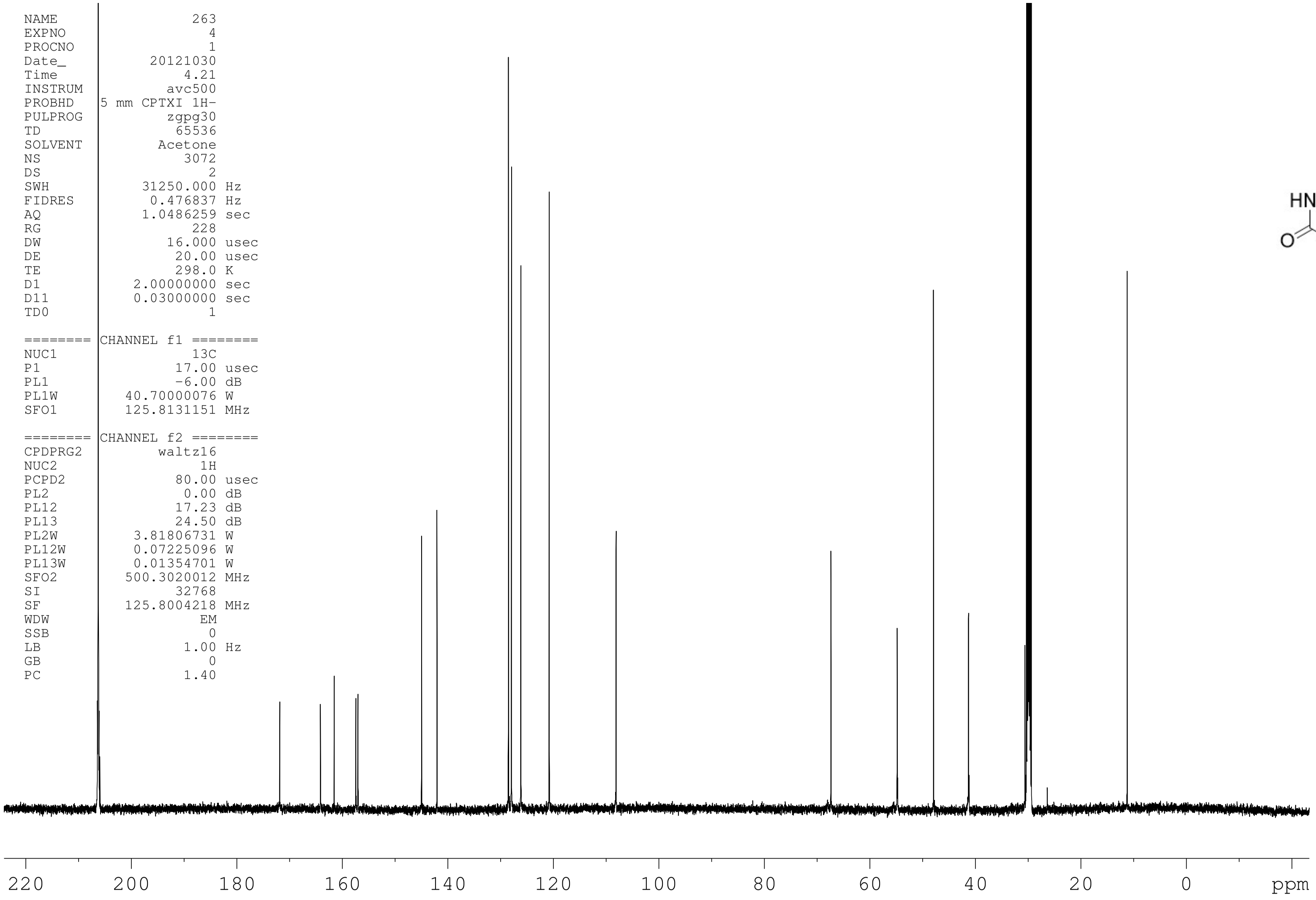

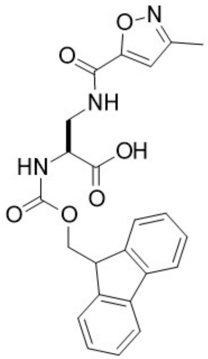

Supplement: Supplementary file 1 — Supplementary [file ANIE-55-8353-s001.pdf]
